# Phosphonamidates Integrating Sterically Hindered Phenols with Membrane-Active Cations: A Redox-Activated Approach to Antimicrobial Agents

**DOI:** 10.3390/ijms27104524

**Published:** 2026-05-18

**Authors:** Elmira Gibadullina, Adel Shakirov, Margarita Neganova, Yulia Aleksandrova, Alexandra Voloshina, Anna Lyubina, Anastasiya Sapunova, Anna Strelnik, Kamil Ivshin, Assel Shuragaziyeva, Altynkul Toibazarova, Banu Diyarova, Anipa Tapalova, Nurbol Appazov, Alexander Burilov

**Affiliations:** 1 Arbuzov Institute of Organic and Physical Chemistry, FRC Kazan Scientific Center, Russian Academy of Sciences, Akad. Arbuzov St. 8, Kazan 420088, Russia; shakirov-adel@inbox.ru (A.S.); microbi@iopc.ru (A.V.); burilov_2004@mail.ru (A.B.); 2 Laboratory of Engineering Profile “Physical and Chemical Methods of Analysis”, Korkyt Ata Kyzylorda University, 29A, Aiteke Bi Street, Kyzylorda 120014, Kazakhstan; neganovam@ipac.ac.ru (M.N.); yulia.aleks.97@mail.ru (Y.A.); nurasar.82@korkyt.kz (N.A.); 3 A.N. Nesmeyanov Institute of Organoelement Compounds of Russian Academy of Sciences, Vavilova St. 28, Bld. 1., Moscow 119334, Russia; 4 Department of Chemistry, Institute of Natural Science, Kazakh National Women’s Teacher Training University, 144/8 Gogol Street, Almaty 050000, Kazakhstan

**Keywords:** antimicrobial activity, phosphonamidates, sterically hindered phenols, quinone methide, redox activation, bacterial resistance, MRSA, cytotoxicity profile, quaternary ammonium salts, structure-activity relationship

## Abstract

A strategy to create highly effective antimicrobial agents was proposed based on the conjugation of three functional components: a cationic quaternary ammonium salt (QAS) that exerts a membrane-disrupting effect and promotes selective accumulation on bacterial surfaces; a phosphonamidate linker for controlled activation; and a sterically hindered phenol (SHP) fragment as a potential redox component. This approach enabled the preparation of 40 target phosphonamidate–SHP/QAS hybrids in high yields (88–98%). Evaluation of their antimicrobial activity against major pathogens and methicillin-resistant *Staphylococcus aureus* (MRSA) revealed high potency against Gram-positive bacteria. The lead compounds achieved minimum inhibitory concentration (MIC) values of 0.7–2.8 μM, which is up to 10 times lower than that of the reference drug, norfloxacin. Mechanistic studies confirmed that these hybrids disrupt the bacterial membrane. In addition, an increase in intracellular ROS levels was observed for the most active compound. The SHP/QAS hybrids retained high activity against *S. aureus* ATCC 209P after 17 passages and showed low cytotoxicity (SI = 62–92) and negligible hemolysis. These properties indicate that this approach may offer a useful strategy for developing antibacterial agents with a potentially lower risk of inducing conventional resistance mechanisms.

## 1. Introduction

The escalating global problem of antimicrobial resistance (AMR) poses a critical threat to public health, necessitating the development of new therapeutic strategies capable of circumventing the complex adaptation mechanisms of pathogens [1,2]. These mechanisms include evolved resistance strategies such as enzymatic drug modification, efflux pump regulation, or target site mutations that counteract inhibitors of essential processes (e.g., translation, transcription, and replication) [3,4,5,6]. Unlike the direct inhibition of individual enzymes, a strategy aimed at disrupting fundamental cellular processes potentially allows one to “bypass” many of these classic resistance mechanisms. A promising direction in this context is the deliberate disruption of redox homeostasis, whose vulnerability is confirmed by its role in bacterial survival and involvement in the mechanism of action of antimicrobial agents [7,8,9,10,11]. The accumulation of reactive oxygen species (ROS) acts as both a direct consequence and a key tool of such disruption, as the fundamental survival system of bacteria itself becomes the target. Based on derivatives of 2,6-di-*tert*-butyl-4-methylphenol (BHT), a traditional sterically hindered phenol (SHP) distinguished by its distinct dual redox activity (anti-/pro-oxidant), our team is creating a platform of multifunctional “hybrid” molecules [12,13,14,15,16,17]. SHP is being used to synthesize novel physiologically active chemicals with regulated redox switching, which offer selective cytotoxic activation in diseased foci and antioxidant protection in healthy settings. Although the antimicrobial activity of BHT has been known since the 1980s [18,19,20], the molecular basis of this phenomenon remains incompletely understood. Current data suggest that the key role may belong to BHT’s pro-oxidant activity, specifically its ability to generate ROS and electrophilic metabolites [21,22,23]. This duality highlights that the biological activity and toxicity of BHT are a direct consequence of its redox and alkylating properties. It should be noted that the ability of BHT derivatives to transform into highly reactive quinone methides (QMs), which act as powerful Michael acceptors, covalently modifying critical nucleophilic centers in the cell, represents a promising but complex pathway for direct redox targeting. The main challenges here remain controlling the specificity of QM activation and minimizing unwanted effects. To address these challenges, we previously proposed a strategy of controlled in vivo activation, where the introduction of electron-accepting phosphoryl groups into the benzyl position of the SHP plays a key role, facilitating the formation of QMs and their anion radicals [16,17] in the synthesis of antitumor compounds. In previous studies, hybrid molecules combining an SHP core with cationic quaternary ammonium salt motifs (QAS) via an alkylamine linker (aminophosphonates and their phosphorus-free analogs) have demonstrated high activity against Gram-positive and Gram-negative pathogens, as well as selectivity toward bacterial cells [13]. It is important to note that phosphorus-free analogs of these hybrids served as key components of the first cationic nanocarriers for intranasal delivery, which in vivo demonstrated the ability to cross the blood–brain barrier in vivo, to inhibit brain cholinesterase, and to reduce Alzheimer’s disease symptoms, confirming their unique combination of enzymatic inhibition with antioxidant protection [12,24]. As bacterial resistance continues to grow and the discovery of new antibiotics stagnates, key bioavailability challenges—including the barrier function of Gram-positive and Gram-negative bacterial membranes and the activity of efflux pumps—critically limit the effectiveness of most antimicrobial candidates.

**Figure 1:**
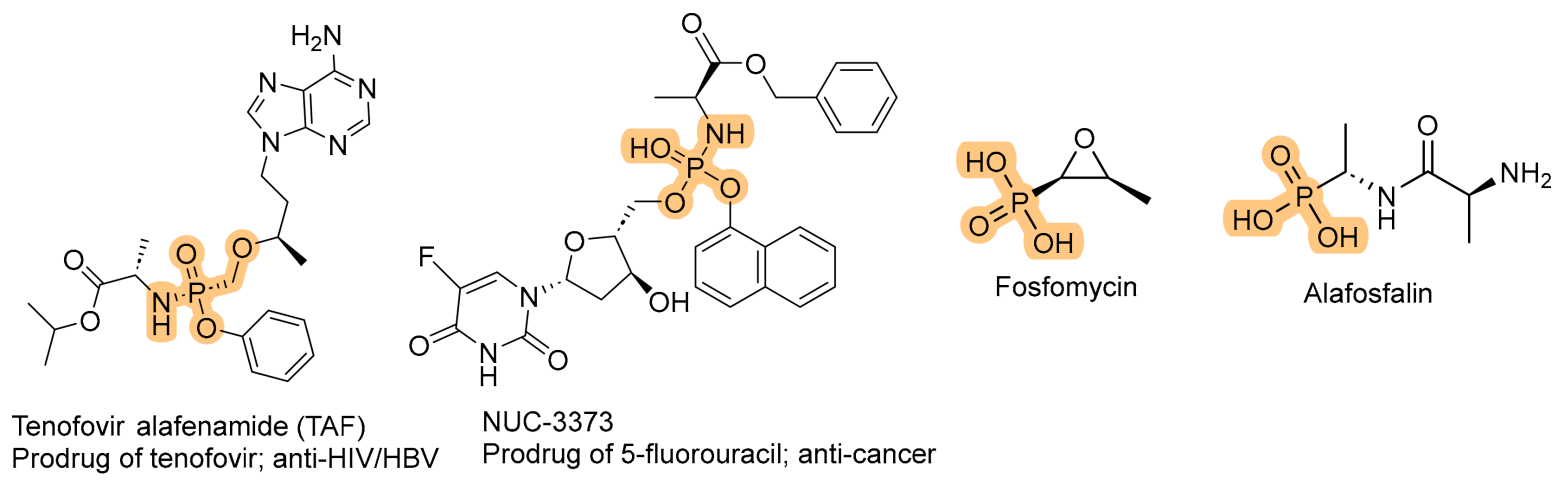
Phosphonamidate prodrug and phosphorus-based antibacterial agents in clinical practice.

Phosphonamidate prodrugs, which have proven effective in antiviral (tenofovir alafenamide) and anticancer therapy (5-fluorouracil-NUC-3373), offer a strategic solution due to an improved lipophilicity/solubility balance, and intracellular cleavage of the P–N bond by phosphoramidases ensures selective activation [25,26,27,28,29,30,31]. However, in the field of antibacterial agents, such systems remain poorly understood despite their potential to overcome membrane barriers. Representative examples of both classes are shown in Figure 1.

**Figure 2:**
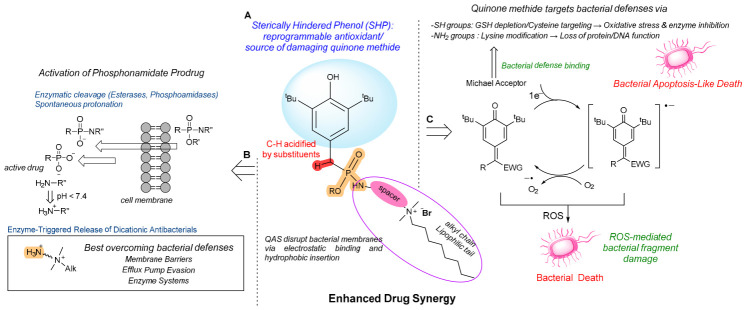
(**A**) General design strategy for antibacterial agents based on phosphonamidates SHP/QAS synergy; (**B**) Bacterial enzyme-mediated activation via P–N bond cleavage; (**C**) Redox-switchable QM generators: ROS/Electrophile cooperation in bacterial death.

Thus, building on the successes with SHP/QAS hybrids and understanding the potential of redox targeting, we modified the structure of the hybrids by including a phosphonamidate linker. The overall design is presented in Figure 2.

This approach combines three key mechanisms:

- a potential prodrug mechanism, where hydrolysis of the P–N amide bond enables the release of the active component in vivo by phosphoramidase enzymes.
- regulated activation of QMs, facilitated by the electron-withdrawing effect of the phosphoryl group, which increases the acidity of the benzylic C–H bond. This pathway induces oxidative stress and covalent alkylation of critical targets, ultimately leading to apoptosis-like cell death in bacteria.
- the release of functionalized aminoalkyl quaternary ammonium fragments, which form a highly active multicationic structure under physiological conditions via protonation of the amino group.

The formation of an additional charging cation in situ provides a significant advantage. This allows for an enhancement of the membrane-disrupting effect, which will lead to the minimization of the influence of efflux pumps. This hypothesis is supported by numerous studies indicating that the presence of a charged cationic moiety is key to the development of new antimicrobial drugs. Additionally, studies [32,33,34] have shown that polycationic compounds exhibit improved MIC values, particularly against Gram-negative bacteria [35,36].

Therefore, our proposed design of phosphonamidate–SHP/QAS hybrids is aimed at enhancing antimicrobial activity and potentially reducing the risk of resistance development. The design combines a membrane-disrupting cationic motif with a redox-active SHP fragment that can generate ROS and electrophilic QMs.

In this context, a series of novel phosphonamidates was synthesized and characterized for the first time, and their antimicrobial activity was evaluated against a wide panel of clinically significant pathogens, including multidrug-resistant strains such as MRSA. The rate and extent of resistance development in *S. aureus* (*Sa*) were studied for the most active compounds. Furthermore, their effect on the permeability and integrity of bacterial cytoplasmic membranes was demonstrated. For the identified hit compound, an increase in intracellular ROS levels was observed. This work encompasses the synthesis and study of new compounds in which cooperative effects are realized through the combination of an SHP fragment, a phosphonamidate linker, and a quaternary ammonium salt motif. We believe that this approach may help reduce the potential for resistance development.

## 2. Results

### 2.1. Chemistry

**Scheme 1:**
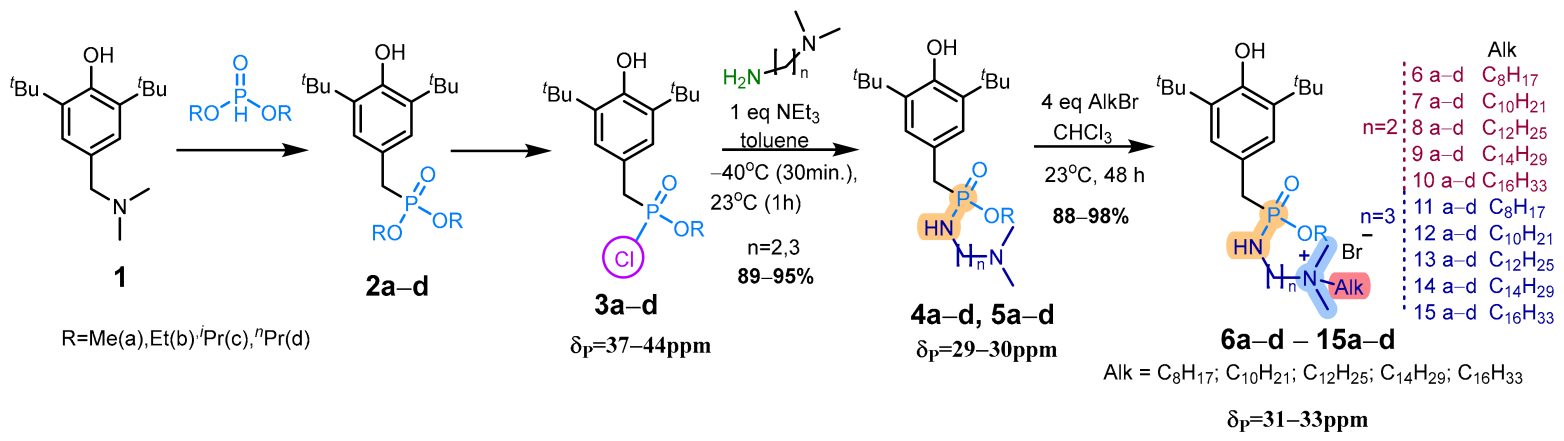
The synthesis of phosphonamidate–SHP/QAS hybrids.

A significant consideration in the synthesis of new organic compounds with practically useful properties is the availability of synthetic reagents. A convenient starting reagent for the synthesis of compounds containing phenolic and phosphoryl fragments is commercially available 2,6-di-*tert*-butyl-4-(dimethylaminomethyl)phenol, and methods for obtaining alkyl(3,5-di-*tert*-butyl-4-hydroxybenzyl)phosphonates based on it are well-developed [37]. The key intermediates in the synthesis of the target hybrids in this work were the *O*-alkyl-3,5-di-*tert*-butyl-4-hydroxybenzylphosphonochloridates **3a**–**d**, developed by our group, which were synthesized by the interaction of benzylphosphonates **2a**–**d** with a 35% excess of PCl_5_ in toluene in 85–92% [38]. Optimization of the temperature regime for the reaction of phosphonochloridates **3a**–**d** with amines containing terminal dimethylamine groups (*N*,*N*-dimethylethylenediamine, *N*,*N*-dimethylpropylene-1,3-diamine) revealed the critical importance of this parameter. When the process was carried out at 60 °C or 23 °C in the presence of triethylamine, in addition to the target product signal of the target product (δp in the 30 ppm region), additional signals were observed at δp 19 ppm (pyrophosphonate) and 27 ppm (salt of alkylphosphonate). These competing processes were suppressed by lowering the temperature to −40 °C, which ensured the formation of phosphonamidate precursors **4a**–**d**, **5a**–**d** in 89–95%, as illustrated in Scheme 1.

**Figure 3:**
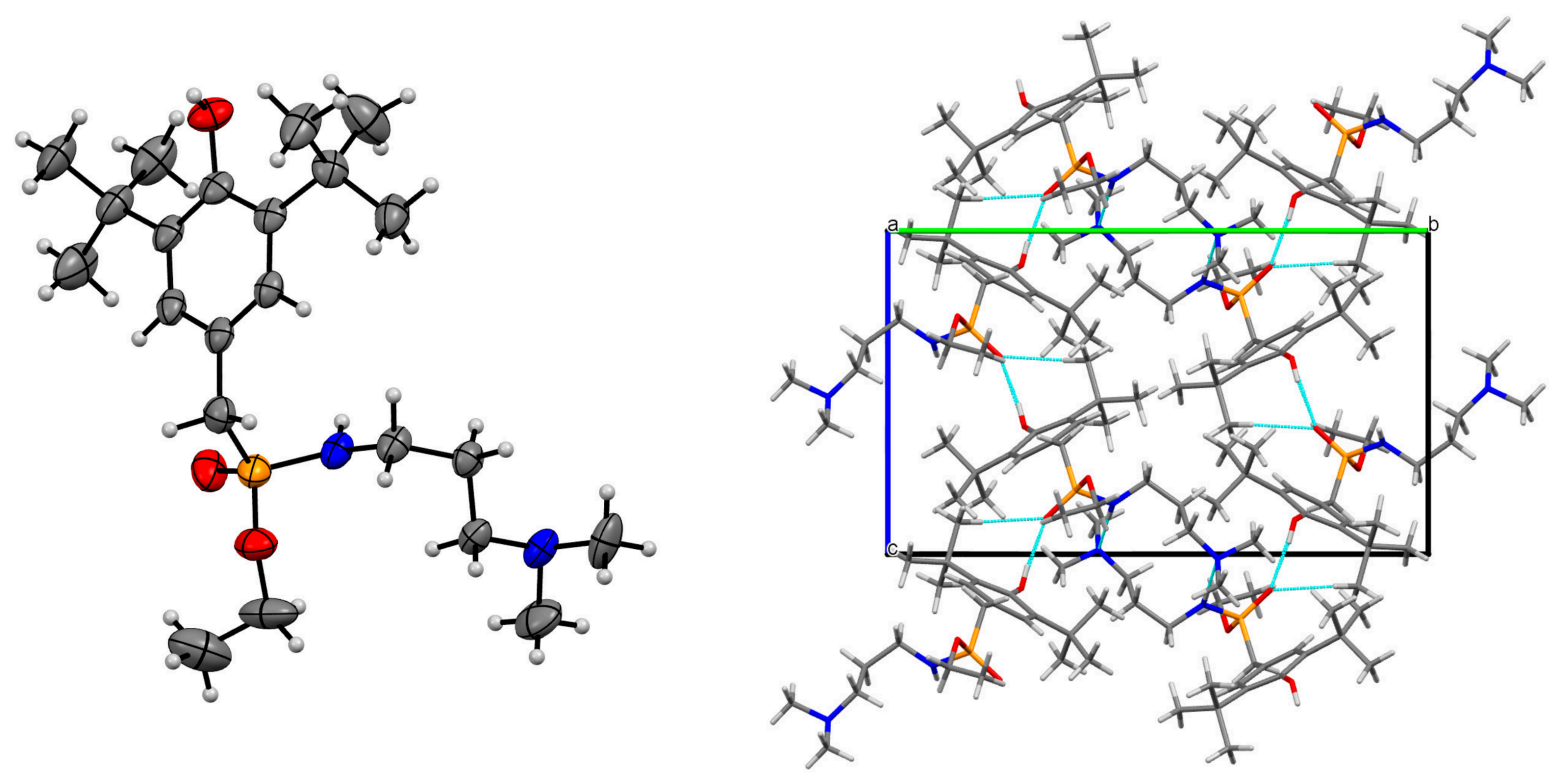
Molecular structure and fragments of crystal packing in of compound **5b**. Colour code: blue—nitrogen, red—oxygen, orange—phosphorus. Labels a, b, c denotes the unit cell axes. Ellipsoids are shown with 50% probability.

The product yield depended on the alkyl substituent at the phosphorus atom, reaching a minimum for methyl derivatives, while the length of the spacer between the nitrogen atoms had no significant effect. The synthesized phosphonamidates **4a**–**d**, **5a**–**d** (8 compounds) were characterized by ^1^H, ^13^C, ^31^P NMR spectroscopy, IR spectroscopy, and mass spectrometry (ESI-TOF). Single crystals of compound **5b**, grown from acetone recrystallization, were analyzed via high-resolution X-ray diffraction at 150(2) K. The compound crystallizes in the monoclinic space group *P*2_1_/*n*, confirming a racemic assembly despite molecular chirality at the tetrahedral phosphorus center (due to four distinct substituents: phenolic, phosphoryl, amino, and alkoxy groups). As shown in Figure 3, the structure of **5b** features N–H⋯N-bonded dimers that assemble into supramolecular layers stabilized by bifurcated O⋯H–O and O⋯H–C interactions between phosphoryl oxygen and phenolic fragments, with additional reinforcement from H⋯H contacts (see Supplementary Materials pp. S27–S28, Figure S1, Table S1 for a more detailed discussion).

Spectral data (a doublet at 3.03 ppm for the -CH_2_- group with *J*_PH_ = 20.3 Hz; the absence of diastereomeric signals; a single signal in the 30 ppm region in the ^31^P NMR spectrum) combined with the results of X-ray diffraction analysis of compound **5b** confirm the formation of racemic mixtures of the obtained compounds **4a**–**d**, **5a**–**d**.

Subsequent quaternization with alkyl bromides (4 eq., CHCl_3_, 48 h) yielded 40 target compounds **6a**–**d**–**15a**–**d** in 88–98%. Chloroform was selected as the solvent because it gave higher yields than acetonitrile or toluene in preliminary experiments. The overall synthetic route is summarized in Scheme 1. It is important to note that the excess alkylating agent is effectively removed by washing with hexane without chromatographic purification. The synthesized series of phosphonamidate was characterized based on ^1^H, ^13^C, ^31^P NMR spectroscopy, IR spectroscopy, and mass spectrometry (ESI-TOF) data. Thus, optimal conditions for the synthesis of 8 phosphonamidates and 40 ammonium salts based on them, containing an SHP fragment, have been developed.

### 2.2. Biological Evaluation

A comprehensive range of antibacterial properties was evaluated for the synthesized phosphonamidate–SHP/QAS hybrids **6a**–**d**–**15a**–**d** to determine their antimicrobial potential and elucidate mechanisms of action. Specifically, we studied direct antibacterial activity against clinical isolates including methicillin-resistant *Staphylococcus aureus* (MRSA) through MIC/MBC determinations; cytotoxicity profiles via hemolytic activity (HC_50_) and mammalian cell viability (Chang Liver, IC_50_) to establish selectivity indices; membrane integrity disruption using propidium iodide uptake assays; intracellular ROS generation measured with CellROX^®^ Deep Red fluorescence for hit compound; and resistance development potential through serial passage experiments.

This approach provides a comprehensive characterization of the antimicrobial potential of phosphonamidate–SHP/QAS hybrids, combining screening tests (MIC) with mechanistic analysis for the rational design of new antimicrobial drugs.

#### 2.2.1. In Vitro Antimicrobial Activity

The synthesized phosphonamidate–SHP/QAS hybrids were tested for in vitro inhibitory activity against standard pathogenic strains, namely: Gram-positive bacteria—*Staphylococcus aureus* ATCC 6538 P FDA 209P (Sa), *Bacillus cereus* ATCC 10702 NCTC 8035 (Bc), *Enterococcus faecalis* ATCC 29212 (Ef); Gram-negative bacteria—*Escherichia coli* ATCC 25922 (Ec), *Pseudomonas aeruginosa* ATCC 9027 (Pa) and fungi: *Trichophyton mentagrophytes var. gypseum* 1773 (Tm) and *Candida albicans* ATCC 10231 (Ca). Bacteriostatic and fungistatic activity was studied using the serial dilution method in liquid nutrient medium according to a previously described procedure [39] by determining the minimum inhibitory concentration (MIC), which causes the suppression of the growth and reproduction of the test microorganism. Bactericidal and fungicidal activity, causing the death of microbial cells, was determined using the methods from [40]. The tests were conducted in triplicate and repeated three times. Table 1 presents the antimicrobial activity data for the synthesized phosphonamidates **6a**–**d**–**15a**–**d**. The compounds exhibited activity (bacteriostatic, fungistatic, bactericidal, and fungicidal) within a concentration range of 0.70–412 μM. They demonstrated pronounced antibacterial efficacy against Gram-positive microorganisms. The MIC values for the most active compounds against *S. aureus* ranged from 0.7 to 6.3 μM. Notably, compounds **12d** and **13b**–**d** (MIC = 0.7–0.8 μM) were up to 10 times more potent than the reference drug norfloxacin. Against the Gram-negative bacterium *E. coli*, compounds **8c** and **13b**–**d** showed moderate activity, with MIC values of 11.6–11.8 μM. Furthermore, compounds **8a**–**c**, **9a**, **13c**,**d** exhibited fungicidal activity, with MIC values ranging from 11.6 to 24.6 μM, which was slightly lower than that of the reference drug ketoconazole (MIC = 7.3 μM).

Based on the antimicrobial screening results, which revealed that the synthesized phosphonamidate–SHP/QAS hybrids are active against Gram-positive bacteria, their efficacy against clinically significant MRSA strains was evaluated. The MRSA-1 and MRSA-2 strains used in this study were isolated from patients with chronic tonsillitis and sinusitis, respectively, at the bacteriological laboratory of the Republican Clinical Hospital (Kazan, Russia). The study focused on compounds that exhibited an MIC of ≤6.3 μM against the reference strain *S. aureus* ATCC 209P. The antibacterial activity (MIC/MBC) of the most active phosphonamidates against these clinical MRSA isolates is presented in Table 2.

The obtained MIC/MBC values indicate that compounds **8c** and **12d**, **13c**,**d** showed the highest activity against MRSA isolates, with MIC/MBC ranges of 0.7–2.8 μM and 0.7–11.8 μM, respectively. The most effective compound was **13c**, with MIC/MBC values of 0.7/1.3 μM against *S. aureus* and 2.8/2.8 μM against the MRSA strains, demonstrating superior overall activity including potent bactericidal action against MRSA-2 and pronounced effects against *E. coli* (11.8/11.8 μM) and *C. albicans* (11.6/23.1 μM). The least effective compounds (**15a**,**b**,**d**; **6a**–**c**; **10a**–**d**; **11a**,**c**,**d**) had MIC values >25 μM. A comparison with reference antibiotics revealed that the most active compounds were hundreds of times more potent than ciprofloxacin against the MRSA-1 isolate.

#### 2.2.2. SAR Study of Antimicrobial Phosphonamidate–SHP/QAS Hybrids

**Figure 4:**
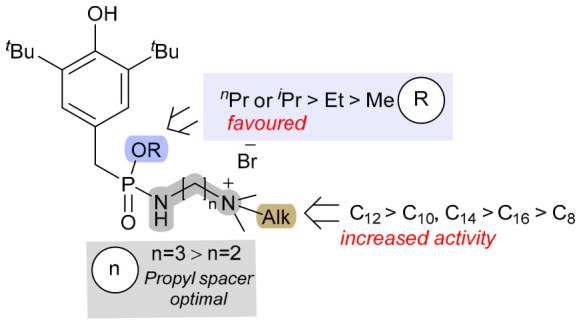
SAR phosphonamidate–SHP/QAS hybrids.

The study of in vitro antimicrobial activity of 40 synthesized phosphonamidate–SHP/QAS hybrids revealed distinct structure-activity relationships (SAR). Figure 4 presents the main structure-activity relationships (SAR) for this class of compounds, demonstrating the influence of alkyl chain length and the nature of substituents at the phosphorus atom on antimicrobial efficacy.

The obtained compounds demonstrated pronounced and selective activity against Gram-positive bacteria, showing the greatest effectiveness against *S. aureus* ATCC 209P and its methicillin-resistant isolate (MRSA). The most active are derivatives with decyl (C_10_), dodecyl (C_12_), and tetradecyl (C_14_) substituents, with MIC values ranging from 0.7 to 6.3 μM, which are superior to the comparison drug norfloxacin (MIC = 7.5 μM). Activity against *E. coli* was lower (MIC = 11.6 μM). Although this is lower than the activity of the same compounds against *S. aureus*, it is significant because it demonstrates the retention of antibacterial effect even despite conditions of limited permeability of the Gram-negative bacterial outer membrane and is consistent with our hypothesis that the compounds can overcome the barriers of the complex cell wall. The observed effect is likely related to the formation of a multicationic structure. SAR analysis showed that optimal activity was achieved when isopropyl/propyl substituents on the phosphorus atom in SHP/QAS hybrids were combined with long-chain alkyl fragments (C_10_–C_14_) on the nitrogen atom.

#### 2.2.3. Cytotoxicity of Phosphonamidate Hybrids on Erythrocytes and Chang Liver Cells and Their Selectivity Index Against *S. aureus* ATCC 209P 

The safety assessment of the synthesized compounds included a study of their cytotoxic effect on two experimental models: mammalian blood erythrocytes (hemolytic activity) and a human Chang Liver (HeLa) cell line. These models allow for the assessment of potential toxic effects on the blood and the main metabolizing organ—the liver. Data on hemolytic and cytotoxic activity are presented as HC_50_ values (the concentration of the test compound that causes 50% hemolysis of erythrocytes in the experiment) and IC_50_ values (the concentration of the test compound that causes the death of 50% of cells in the experimental population) in Table 3. Compound **7b** (HC_50_ = 11 μM) showed the highest hemotoxicity, while the other compounds exhibited moderate to low toxicity, with HC_50_ values ranging from 26 to 276 μM. Cytotoxicity against liver cells (IC_50_) ranged from 35 to 71 μM, which is significantly higher than the MIC/MBC values for microbial cells, indicating a favorable selectivity index. The comparison drug CTAB proved to be much more toxic to erythrocytes and liver cells.

The key safety assessment parameter was the selectivity index (SI), calculated as the ratio between the HC_50_ value for erythrocytes (IC_50_ for eukaryotic cells) and the MIC value for bacterial cells of *S. aureus* (see Table 3). The compounds **13b** (SI = 78/78), **13c** (SI = 62/90), and **12d** (SI = 86/87) showed the highest selectivity against *S. aureus* ATCC 209P. Compounds **15d** (SI = 70) and **8a** (SI = 92) showed increased selectivity toward blood cells.

The obtained results demonstrate that the synthesized phosphonamidates exhibit pronounced selectivity of action in favor of antimicrobial activity, which confirms their promise for further study as potential antimicrobial agents. The selectivity index values significantly exceed the minimum threshold values, indicating a favorable safety profile.

#### 2.2.4. Study of Drug Resistance of Phosphonamidates SHP\QAS—**8c**, **12c–d**, **13a**–**d**, **14c**, **15c**

**Figure 5:**
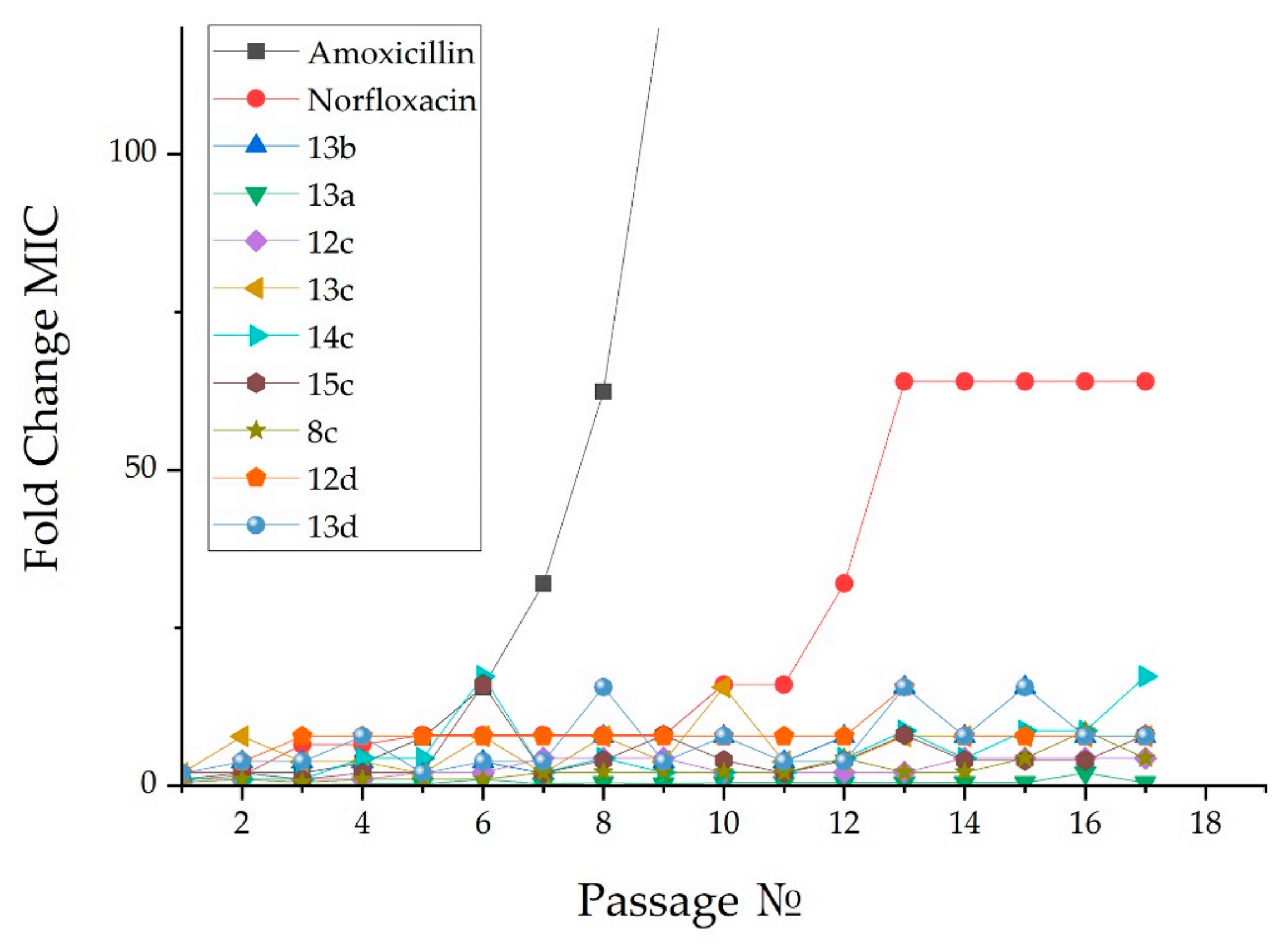
Bacterial resistance studies of compounds **8c**, **12c–d**, **13a**–**d**, **14c**, **15c**, **norfloxacin**, **amoxicillin** against *S. aureus* ATCC 209P.

Drug resistance testing was performed according to the method of Lin S. et al. [41]. Known antibacterial drugs from various classes were used as reference drugs: norfloxacin and amoxicillin. The MIC of the compounds under study and reference drugs were determined by the serial microdilution method in 96-well plates. The MIC results were obtained after 24 h of incubation. To further determine the corresponding MIC, a bacterial suspension of *S. aureus* ATCC 209P was used, obtained in the presence of 0.5× MIC for each substance. Similar measurements were taken over 17 passes. The experiment was conducted in three replicates. The results of the resistance studies for the investigated compounds **8c**, **12c**–**d**, **13a**–**d**, **14c**, **15c** in comparison with the reference antibiotics norfloxacin and amoxicillin against *S. aureus* ATCC 209P are presented in Figure 5.

Figure 5 shows that even after 17 passages and incubation in the presence of the compounds under study, stable resistant bacterial populations did not emerge. The minimum inhibitory concentration for most compounds increased by more than 2 times in the last few passages (15–17) alone. At the same time, the MICs of the reference comparator drugs significantly increased, starting from passage 8.

Thus, these results indicate that exposure to the compounds under investigation does not lead to the development of resistance in *S. aureus* ATCC 209P during long-term in vitro experiments.

#### 2.2.5. The Effect of Phosphonamidate–SHP\QAS Hybrids on the Bacterial Cell Wall

**Figure 6:**
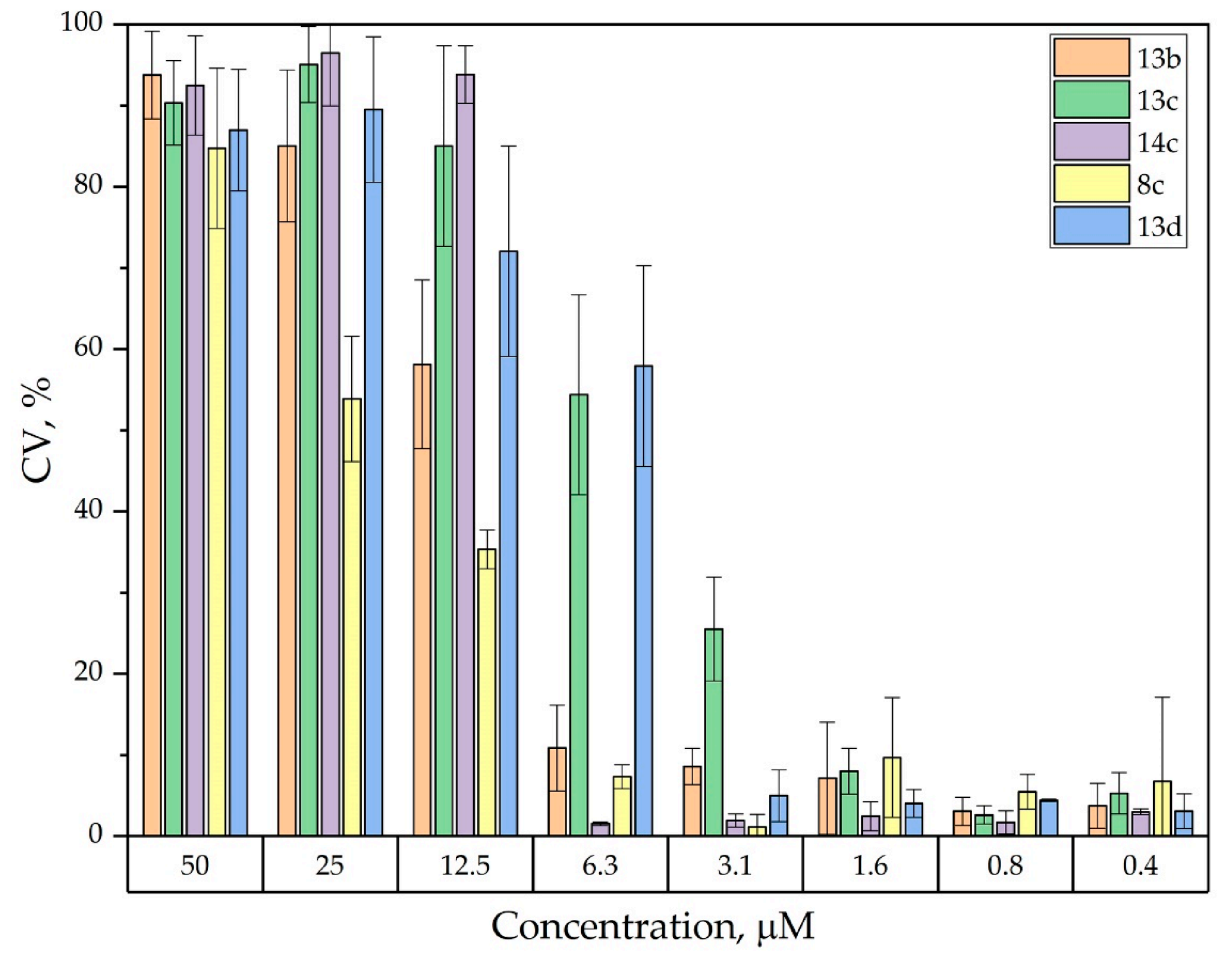
Change in the degree of damage to the cell wall of *S. aureus* determined by the percentage of crystal violet (CV) absorption in the presence of different concentrations of test compounds.

The search for new antimicrobial agents often targets bacterial surface structures (the cell wall and cytoplasmic membrane) because these components are crucial for cell integrity. This makes them attractive targets for antibacterial drug development, particularly in the context of growing antibiotic resistance. To assess the mechanism of action of the lead compounds **8c**, **13b**–**d**, and **14c**, their effect on the *S. aureus* cell wall was investigated using a colorimetric method with crystal violet (CV). This dye forms strong complexes with the negatively charged components of peptidoglycan and teichoic acids. Damage to the cell wall leads to the release of CV into the supernatant, which is detected spectrophotometrically [42]. Studies within the concentration range (including MIC and MBC) showed that none of the tested compounds caused significant damage to the cell wall at these concentrations. As shown in Figure 6, even at the maximum concentrations tested, none of the investigated compounds induced statistically significant release of crystal violet from *S. aureus* cells. The most pronounced, but still weak, effect was observed for compound **13c** at a concentration of 2× MBC.

#### 2.2.6. Membrane Integrity Assessment of Phosphonamidate Hybrids **8c**, **13b**–**d**, and **14c** on *S. aureus* Using Propidium Iodide

Determining the integrity of the cytoplasmic membrane is a key step in studying the mechanism of action of antimicrobial compounds. For this purpose, the fluorescent method using propidium iodide (PI) is widely used. As a high molecular weight probe, it does not penetrate cells with intact membranes. The binding of PI to DNA and the appearance of red fluorescence indicate a disruption of membrane permeability [43].

**Figure 7:**
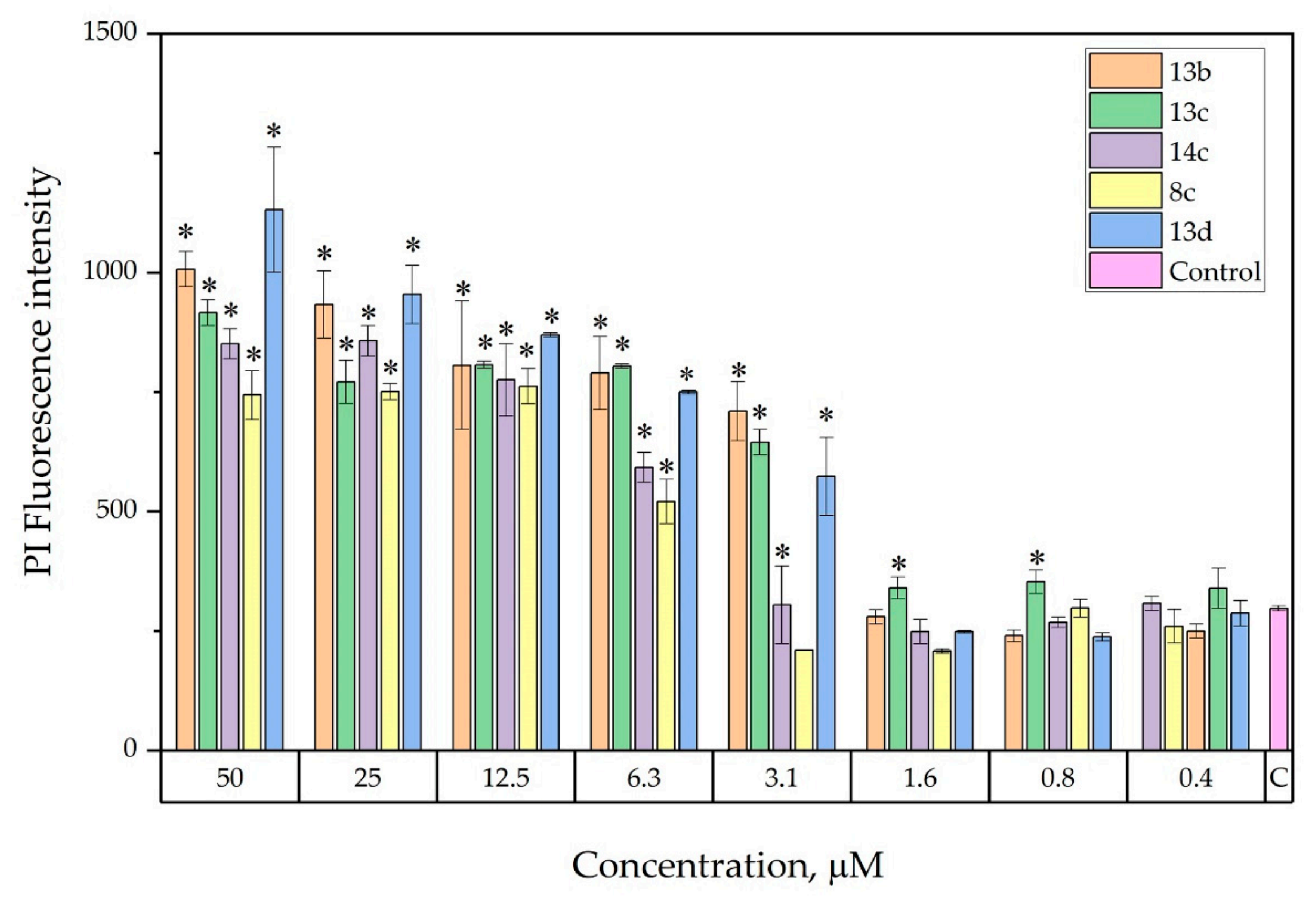
Membrane permeabilization *S. aureus* assay determined using PI dye. Data are presented as mean ± SD of three independent experiments. * Values indicate *p* < 0.05 compared to control.

Figure 7 illustrates the study of the effect of compounds **8c**, **13b**–**d** and **14c** on the *S. aureus* membrane. The strongest effect is characteristic of **13c**, which causes a significant increase in PI fluorescence even at a concentration close to the MIC (0.8 μM). The remaining compounds showed a significant membrane-tropic effect at higher concentrations (2× MIC and above).

The obtained data are consistent with the expected mechanism of action, which is due to the presence of cationic fragments of quaternary ammonium compounds in the molecular structure. Thus, while cell wall damage is absent even at MIC/MBC, disruption of cytoplasmic membrane permeability is observed at lower concentrations (≥0.8 μM), indicating a membrane-directed mechanism of action for the tested compounds.

#### 2.2.7. Analysis of ROS Generation for Active Compound **13c**

**Figure 8:**
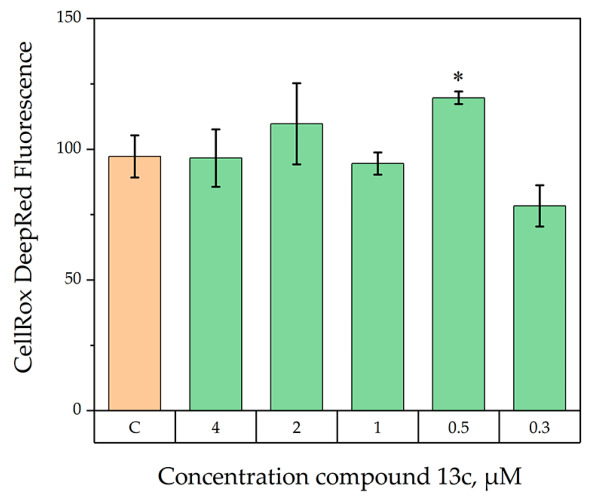
Generation of ROS by compound **13c** in *S. aureus* cells. Data are presented as mean ± SD of three independent experiments. * Values indicate *p* < 0.05 compared to control.

The ROS generation by SHP/QAS hybrids was studied assess their potential to induce oxidative stress as a possible mechanism of antimicrobial action. Compound **13c** was used as an example to investigate the ability to generate ROS because it was the most active representative of the series based on cytotoxicity, selectivity, and membrane-tropic effect results. This allowed us to correlate its leading antimicrobial activity with a potential oxidative mechanism of action. To determine ROS, the stationary fluorescence method was used with the fluorochrome CellROX^®^ Deep Red (Thermo Fisher Scientific, Waltham, MA, USA), which fluoresces in the red region of the spectrum and is used as an indicator of ROS formation in cells. The results are presented in Figure 8.

Compound **13c** generates ROS at concentrations near the MIC (0.5 μM), resulting in significantly higher fluorescence intensity for CellROX^®^ Deep Red compared to the control. When the concentration is increased to MBC or higher, the production of reactive oxygen species is nearly identical to that of the control. At concentrations above the MIC, bacterial viability is inhibited, and both ROS generation and membrane permeability disruption are observed.

## 3. Discussion

The key idea of the work is the combination of three pharmacophoric motifs: a cationic quaternary ammonium center (QAC) with hydrophobic C_8_–C_16_ alkyl substituents, providing membrane-tropic activity and selective accumulation on bacterial surfaces; a phosphonamidate linker acting as a platform for controlled activation; and an SHP with the function of a redox component. Among the 40 newly synthesized phosphonamidate hybrids (SHP/QAS **6a**–**d**–**15a**–**d**), several compounds were discovered that exhibit high submicromolar activity (MIC/MBC = 0.7/1.5 μM) against the Gram-positive bacterium *S. aureus* ATCC 209P and its methicillin-resistant strain MRSA. In addition, these compounds show low cytotoxicity toward blood erythrocytes and liver cells, along with high selectivity (SI > 60). The hybrids retained high activity against MRSA after 17 passages, indicating delayed resistance development. It should be noted that the hit compounds also showed good MIC values of 11.6 μM against the Gram-negative bacterium *E. coli*.

The synthesized compounds have the main structural characteristics of cationic amphiphiles, including a persistently charged quaternary ammonium center and a lengthy hydrophobic alkyl chain. It is well established that such compounds exert their antibacterial activity primarily through non-specific membrane disruption. Accordingly, the presence of the quaternary ammonium cationic center in the phosphonamidate derivatives was shown to destabilize the cytoplasmic membrane in the PI uptake assay. Compound **13c** caused a statistically significant increase in fluorescence even at 1× MIC (0.7 μM), while compounds **8c**, **12c**, **13b**,**d**, and **14c** exhibited a pronounced effect at 2× MIC. The PI uptake data thus indicate that membrane permeabilisation occurs, supporting the interpretation that the loss of barrier function (characteristic of cationic amphiphiles) explains the high antibacterial activity of the hybrids against *S. aureus*, including MRSA.

For the lead compound **13c**, an increase in intracellular ROS levels was observed. This increase could arise from the membrane-disrupting action of the QAC fragment [44,45] or from the pro-oxidant activity of the SHP fragment. The latter cannot be excluded, given the well-documented ability of sterically hindered phenols to participate in redox processes and generate reactive oxygen species [17,46]. However, the precise contribution of the SHP fragment to the antibacterial effect remains to be established, and further mechanistic studies are required to elucidate its role.

**Figure 9:**
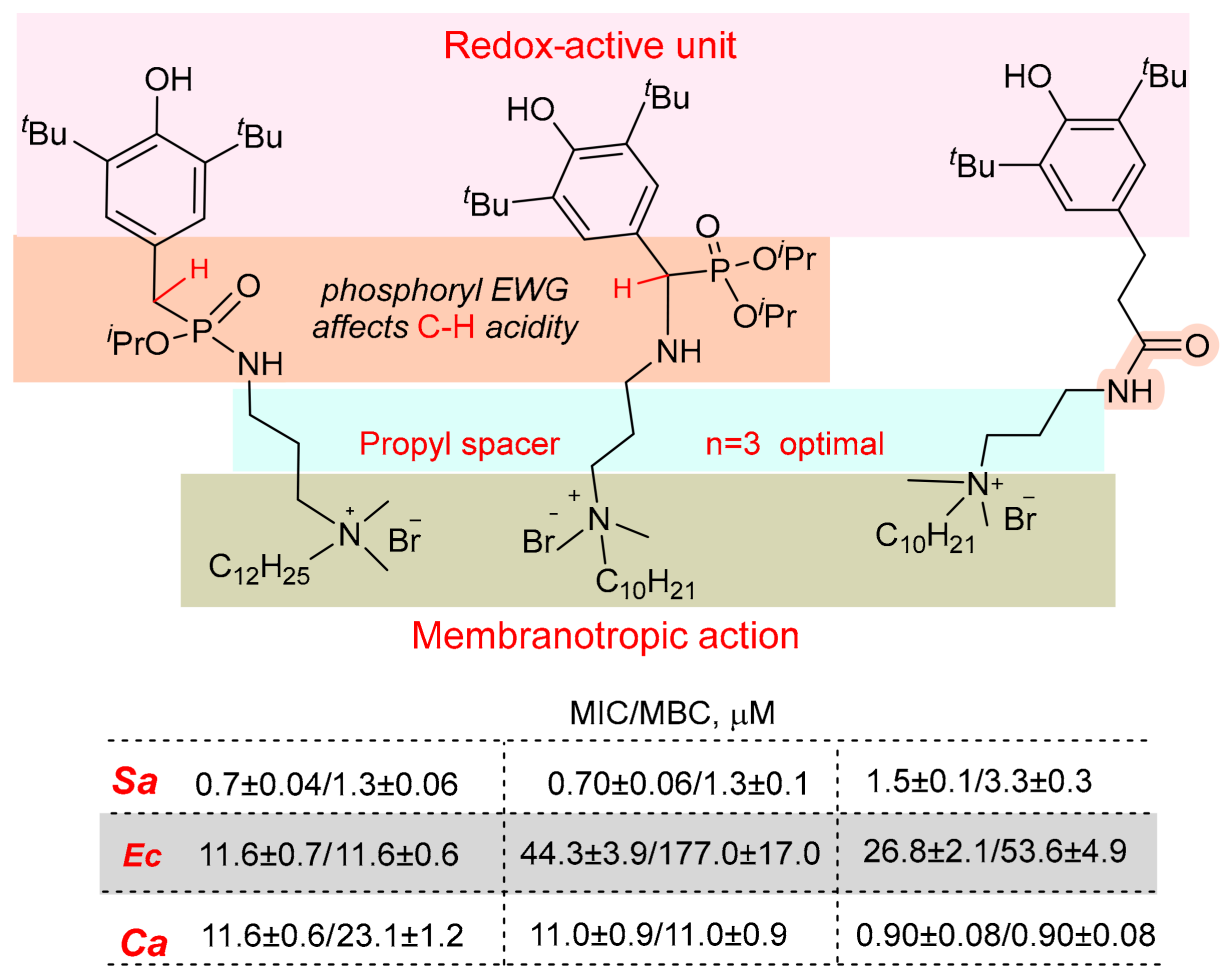
SAR hybrids SHP\QAS.

The introduction of a phosphonamidate linker into the SHP/QAS scaffold offers potential benefits. This linker is expected to enable the selective release of active components, which may enhance specificity. A comparison of the phosphonamidate hybrids with previously synthesized analogs (aminophosphonates and phosphorus-free analogs), whose structures are shown in Figure 9, suggests that the phosphorus-containing SHP fragments are important for activity.

Phosphonoamidates and aminophosphonates exhibit relatively high activity against Gram-positive bacteria (MIC = 0.7 μM), while the MIC for phosphorus-free analogs is twice as high. Phosphonamidates showed an advantage against the Gram-negative bacterium *E. coli* (MIC = 11.6 μM), which is 2- and 4-fold higher than for aminophosphonates and phosphorus-free analogs, respectively, indicating the contribution of the amidophosphonate linker to overcoming Gram-negative barriers. These data suggest that the electron-withdrawing phosphoryl group may influence the redox properties of the phenolic fragment, potentially leading to the formation of superoxide anion and reactive quinone methides and ROS [16,17,46]. This mechanism could complement the main membrane-disrupting action of the QAC fragment. The twofold increase in activity of phosphorus-containing analogs (Figure 9) compared to their phosphorus-free counterparts is consistent with this hypothesis. Moderate antifungal activity likely depends more on lipophilicity and membrane tropism.

The observed increase in ROS levels for compound **13c** is of interest because oxidative stress is known to effectively kill persister cells [47,48,49,50]. This property, together with the membrane-tropic action of the cationic fragment, suggests that the hybrid compounds could be further explored for the treatment of chronic and recurrent infections.

In conclusion, phosphonamidate-linked hybrids combining sterically hindered phenols with membrane-active cations represent a promising class of antibacterial agents. The lead compounds **8c**, **13b–d**, and **14c** demonstrated potent activity against *S. aureus* ATCC 209P, low cytotoxicity toward Chang Liver cells and erythrocytes, and a favorable selectivity index. Importantly, the stability of MIC values over multiple passages indicates a low risk of resistance development, which may be related to the membrane-disrupting activity of the cationic fragment.

## 4. Materials and Methods

### 4.1. Chemicals

#### 4.1.1. Materials

The reagents and solvents used for all activities presented in the research were purchased from local suppliers. Dialkyl (3,5-di-*tert*-butyl-4-hydroxybenzyl)phosphonate **2a**–**d** and *O*-alkyl (3,5-di-*tert*-butyl-4-hydroxybenzyl)phosphonochloridate **3a**–**d** was synthesized according to the literature [37,38].

#### 4.1.2. General Procedures for Compounds’ Identification

The ^1^H- and ^13^C-NMR spectra were recorded on a Bruker AVANCE 400 spectrometer (Bruker BioSpin, Rheinstetten, Germany) operating at 400 MHz (for 1H NMR), 101 MHz (for ^13^C NMR) and 162 MHz (for ^31^P NMR); Brucker spectrometers AVANCEIII-500 (Bruker Corporation, Rheinstetten, Germany) operating at 500 MHz (for ^1^H NMR) and 126 MHz (for ^13^C NMR); Brucker spectrometers AVANCEIII-600 (Bruker Corporation, Rheinstetten, Germany) operating at 600.13 MHz (for ^1^H NMR), 150.19 MHz (for ^13^C NMR) and 242.94 MHz (for ^31^P NMR). Chemical shifts are given relative to the residual signals of the deuterated solvent (CDCl3 δ(^1^H) = 7.26 ppm and δ(^13^C) = 77.0 ppm; d6-acetone δ(^1^H) = 2.09 ppm and δ(^13^C) = 30.6 ppm, 206.2 ppm). IR spectra were recorded on an IR Fourier spectrometer Tensor 37 (Bruker Optik GmbH, Ettlingen, Germany) in the 400–3600 cm^−1^ range in Vaseline or as film on a KBr plate. Electrospray ionization mass spectra (ESI-MS) were obtained on an AmazonX ion trap mass spectrometer (Bruker Daltonik GmbH, Bremen, Germany). Elemental analysis was performed on a CHNS-O Elemental Analyser EuroEA3028-HT-OM (EuroVector S.p.A., Milan, Italy). The melting points were determined on JK-MAM-4 Melting-point Apparatus with Microscope (SGW-X4 JINGKE SCIENTIFIC INSTRUMENT Co., Shanghai, China). The progress of reactions and the purity of products were monitored by TLC on Sorbfil UV-254 plates (Sorbpolimer, Krasnodar, Russia); the chromatograms were developed under UV light. The purity of the synthesized compounds was assessed using elemental analysis (>95%), NMR spectroscopy (^1^H, ^13^C, ^31^P), and mass spectrometry (MALDI). No significant impurities were detected.

##### X-Ray Diffraction

The high-resolution X-ray diffraction data for the single crystals were collected on a Bruker AXS D8 Quest diffractometer at 150(2) K using Mo Kα radiation (λ = 0.71073 Å). Data collection was performed according to recommended strategies employing an ω/φ-scan mode. The programs used: APEX3 for data collection, SAINT for data reduction, SADABS and TWINABS for multi-scan absorption correction, SHELXT for structure solution, SHELXL for structure refinement by full-matrix least-squares against F2 [51,52]. CCDC 2474807 contains the supplementary crystallographic data for this paper. Crystallographic data for structures reported in this paper have been deposited with the Cambridge Crystallographic Data Center.

Crystal data for **5b** C_22_H_41_N_2_O_3_P (M = 412.54 g mol^−1^), monoclinic, space group P21/n at 150(2) K: a = 11.3463(12) Å, b = 19.353(2) Å and c = 11.6092(12) Å, β = 95.019(3)°, V = 2539.4(5) Å3, Z = 4, dcalc = 1.076 g cm^−3^, μ(MoKα)) = 0.130 mm^−1^, F(000) = 904. A total of 75,845 reflections were collected (12,302 independent reflections and 7917 independent reflections with I ≥ 2(σ)), GOOF 1.018, final R indices [I ≥ 2(σ)]: R1 = 0.0844, wR2 = 0.1601, R indices (all data): R1 = 0.1414, wR2 = 0.1916. 

#### 4.1.3. General Procedure for the Synthesis of Phosphonamidate Hybrids SHP/QAS **6a**–**d**–**15a**–**d**

**Synthesis of Phosphonamidate Precursors 4a–d and 5a**–**d**. The corresponding *N*,*N*-dimethylethylenediamine or *N*,*N*-dimethylpropylenediamine (1.0 mmol) and triethylamine (1.0 mmol) were dissolved in 2 mL of absolute toluene. The reaction flask was cooled in a Dewar vessel (in bowl shape, KGW-Isotherm) using liquid nitrogen and kept at −40 °C for 10–15 min. Then, a solution of the alkyl (3,5-di-*tert*-butyl-4-hydroxybenzyl)phosphonochloridate (1.0 mmol) **3a**–**d** in 2 mL of absolute toluene was added dropwise. The mixture was stirred magnetically for 2 h without cooling, allowing it to reach 23 °C. After the precipitate of triethylamine hydrochloride was filtered off, the filtrate was concentrated on a rotary evaporator. The resulting solid was washed with distilled water containing diethylamine (10%) to remove hydrolysis and pyrophosphonate byproducts, followed by hexane, and dried under vacuum (0.06 mm Hg) at 40 °C to constant weight. The intermediate phosphonamidates were obtained as light-colored powders in high yields.

**Quaternary Ammonization (Alkylation)**. To a 5 mL chloroform solution, the phosphonamidate (1.0 mmol) and corresponding bromoalkanes (4.0 mmol) were added. The reaction mixture was stirred for 48 h at room temperature (23 °C), and the reaction progress was monitored by TLC. After the chloroform was removed on a rotary evaporator, the resulting crude product was washed with hexane (3 × 5 mL) and dried under vacuum (0.06 mm Hg) at 40 °C to constant weight. The resulting oil-like products **6a**–**d**–**15a**–**d** were light yellow or brown in high yields.

Synthetic procedures, compound characterization data (Supplementary Materials pp. S2–S26) and the ^1^H-, ^31^P-, ^13^C-NMR spectra of compounds **4a**–**d**–**15a**–**d** are included in the Supplementary Materials (pp. S29–S55, Figures S2–S55).

**Stability assessment**. The stability of compounds **8c**, **12c**, and **13c** under the biological assay conditions was assessed by ^31^P NMR spectroscopy following a published procedure [53,54]. Each compound (5.0 mg) was dissolved in DMSO-d_6_ (0.050 mL) and D_2_O (0.15 mL). After recording an initial spectrum at 37 °C, the appropriate medium (0.30 mL) was added: Mueller-Hinton broth for **12c** and **13c**; cell culture medium for **12c**; and serum albumin solution for **8c**. The mixtures were incubated at 37 °C, and ^31^P NMR spectra were recorded after 1, 12, and 24 h. No degradation was detected at any time point. The corresponding ^31^P NMR spectra are provided in the Supplementary Materials (pp. S56–S57, Figures S56–S59).

### 4.2. Biology

#### 4.2.1. Cells and Materials

For the experiments, we used cell culture Gram-positive bacteria: *Staphylococcus aureus* ATCC 6538 P FDA 209P (*Sa*), *Bacillus cereus* ATCC 10702 NCTC 8035 (*Bc*); *Enterococcus faecalis* ATCC 29212 (*Ef*); Gram-negative bacteria: *Escherichia coli* ATCC 25922 (*Ec*), *Pseudomonas aeruginosa* ATCC 9027 (*Pa*) and fungi: *Trichophyton mentagrophytes var. gypseum* 1773 (*Tm*) и *Candida albicans* ATCC 10231 (*Ca*) from the State Collection of Pathogenic Microorganisms and Cell Cultures “GKPM-Obolensk”. Methicillin-resistant *S. aureus* (MRSA) strains were isolated from patients with chronic tonsillitis (MRSA-1) and sinusitis (MRSA-2) in the bacteriological laboratory of the Republican Clinical Hospital (Kazan, Russia). For the experiments, we used cell culture human liver cells Chang Liver (HeLa)—HeLa-similar cell line from the collection and the Research Institute of Virology of the Russian Academy of Medical Sciences (Moscow). Blood samples for hemolysis and agglutination analysis were collected from the tail tips of outbred Wistar rats (males, 3–4 months old, body weight 300–350 g) in accordance with the protocol of the Ethics Committee of the Federal Research Center “Kazan Scientific Center of the Russian Academy of Sciences” No. 24/1 dated 4 October 2024.

Preparation of bacterial culture. Bacterial (fungal) suspensions for the following methods were prepared as follows: overnight cultures of test microbes were grown in Mueller-Hinton broth (for bacteria) and Sabouraud medium (for fungi) until the mid-exponential growth phase was reached, then centrifuged. The pellet was washed twice with 0.01 M phosphate-buffered saline (PBS, pH 7.2) and centrifuged at 5000 rpm for 10 min. Cells were resuspended in PBS to achieve a final concentration of 2 × 10^8^ CFU/mL (for bacteria) and 2 × 10^3^ CFU/mL. The bacterial (fungal) suspension was then mixed with solutions of test compounds (prepared in PBS) and incubated at 37 °C for 30 min.

#### 4.2.2. Antimicrobial Activity

The antimicrobial activity of the tested compounds was determined using the serial micro-dilution method in 96-well plates. Dilutions were prepared in Mueller-Hinton broth for bacterial cultivation and in Sabouraud broth for fungal pathogens. The bacterial concentration in the experiment was 3.0 × 10^5^ CFU/mL, and the fungal concentration was 2.0 × 10^2–3^ CFU/mL. The results were recorded every 24 h for 5 days. Bacterial and fungal cultures were incubated at 37 °C and 25 °C, respectively. The experiment was repeated three times. For better solubility of the substances in the nutrient medium, 5% DMSO was added—the tested strains did not lose viability at this concentration. To determine the minimum bactericidal and fungicidal concentrations (MBC, MFC), an aliquot of the test microorganism suspension was transferred onto agar-based nutrient medium and incubated at 37 °C or 25 °C, respectively. The MIC or MFC represents the lowest concentrations at which no microbial colonies were found, indicating that they died with an efficacy greater than 99.9% [39,40].

#### 4.2.3. Hemolytic Activity

The hemolytic activity of the systems under investigation was evaluated by comparing the optical density of a solution containing the compound under study with the optical density of blood at 100% hemolysis. Rat erythrocytes were used as the research object. Erythrocytes collected with EDTA were washed three times with physiological saline (0.9% NaCl), centrifuged for 10 min at 800 rpm, and resuspended in physiological saline (0.9% NaCl) to a concentration of 10%. The tested concentrations were prepared in physiological saline (0.9% NaCl). The compounds at the appropriate dilution (450 μL) were added to 50 μL of a 10% erythrocyte suspension. The samples were incubated for 1 h at 37 °C and centrifuged for 10 min at 2000 rpm. Hemoglobin release was monitored by measuring the optical density of the supernatant on an Invitrologic (Novosibirsk, Russia) microplate reader at λ = 540 nm. A control sample corresponding to zero hemolysis (blank) was prepared by adding 50 μL of a 10% erythrocyte suspension to 450 μL of physiological saline solution (0.9% NaCl). A control sample corresponding to 100% hemolysis was prepared by adding 50 μL of a 10% erythrocyte suspension to 450 μL of distilled water.

#### 4.2.4. MTT Assay

The cytotoxic effect on cells was determined using the colorimetric method of cell proliferation—the MTT test. NADP-H-dependent cellular oxidoreductase enzymes can, under certain conditions, reflect the number of viable cells. These enzymes are able to reduce the tetrazolium dye (MTT)—3-(4,5-dimethylthiazol-2-yl)-2,5-diphenyl-tetrazolium bromide to insoluble blue-violet formazan, which crystallizes inside the cell. The amount of formazan formed is proportional to the number of cells with active metabolism. Cells were seeded on a 96-well Nunc plate at a concentration of 5 × 10^3^ cells per well in a volume of 100 μL of medium and cultured in a CO_2_ incubator at 37 °C until a monolayer was formed. Then the nutrient medium was removed and 100 μL of solutions of the test drug in the given dilutions were added to the wells, which were prepared directly in the nutrient medium with the addition of 5% DMSO to improve solubility. After 48 h of incubation of the cells with the tested compounds, the nutrient medium was removed from the plates and 100 μL of the nutrient medium without serum with MTT at a concentration of 0.5 mg/mL was added and incubated for 4 h at 37 °C. Formazan crystals were added to 100 μL of DMSO in each well. Optical density was recorded at 540 nm on an Invitrologic microplate reader (Novosibirsk, Russia). The experiments for all compounds were repeated three times.

#### 4.2.5. Drug Resistance Study

The development of drug resistance was investigated using the method described by Lin et al. [41]. The reference antibacterial drugs Norfloxacin and Amoxicillin were used as controls. The minimum inhibitory concentration (MIC) of the test compound and reference drugs was determined as described above. MIC readings were taken after 24 h of incubation. A bacterial suspension of *S. aureus* ATCC 209P, prepared in the presence of 0.5× MIC for each compound, was used for subsequent MIC determination. This process was repeated over 17 passages. The experiment was performed in triplicate.

#### 4.2.6. Evaluation of Bacterial Membrane Permeability

To study the membrane permeability of *S. aureus* 209 P bacteria, high-molecular fluorescent dye propidium iodide (PI, Sigma-Aldrich, St. Louis, MO, USA) was added to the test and control samples after incubation. The samples were then kept at 37 °C for 20 min in the dark [55]. The final concentration of PI in the samples was 1.5 μM. The results were evaluated using a Hitachi F-7100 fluorescence spectrophotometer (Tokyo, Japan) at an excitation wavelength of 544 nm and an emission wavelength of 620 nm. Bacterial cells incubated in the absence of the test compounds were used as a control.

#### 4.2.7. Intracellular ROS Generation

The intracellular production of reactive oxygen species (ROS) was assessed using 2′,7′-dichlorodihydrofluorescein diacetate (H_2_DCFDA). H_2_DCFDA is a chemically reduced, non-fluorescent form of fluorescein used as an ROS indicator in cells. Upon cleavage of acetate groups by intracellular esterases and subsequent oxidation, the non-fluorescent H_2_DCFDA is converted into 2′,7′-dichlorofluorescein (DCF), which exhibits strong green fluorescence. To detect ROS, an overnight culture of *S. aureus* was grown to mid-logarithmic phase, centrifuged at 5000 rpm for 10 min, and washed with physiological saline. The bacterial suspension was adjusted to 10^8^ CFU/mL and incubated with the test compounds for 30 min at 37 °C. Subsequently, H_2_DCFDA dye was added to a final concentration of 25 μM in the culture medium, followed by an additional 30 min incubation at 37 °C. ROS production in the cells was immediately measured using a Hitachi F-7100 fluorescence spectrophotometer (Japan) with an excitation wavelength of 495/529 nm and an emission wavelength of 485/535 nm [56].

#### 4.2.8. Statistical Analysis

Statistical analysis of the data was performed using Past 4.17 and Microsoft Office Excel 2016. The significance of differences in mean values was determined using the Mann–Whitney U test (*p* ≤ 0.05), taking into account the Bonferroni correction. The IC_50_ values were calculated using the online calculator MLA—Quest Graph™ IC_50_ Calculator AAT Bioquest, Inc. (AAT Bioquest, Inc., Pleasanton, CA, USA), 13 March 2025. The corresponding dose–response curves are provided in the Supplementary Materials (Table S2, p. S58).

## 5. Conclusions

The results of a study on the antimicrobial activity of a series of 40 phosphonamidates containing SHP fragments and cationic quaternary ammonium groups suggest that they exhibit enhanced antimicrobial action against Gram-positive bacteria *S. aureus*, including strains MRSA. The most active in this series were the SHP/QAS phosphonamidates with isopropyl and propyl substituents at the phosphorus atom, a propylene spacer, and hydrophobic long-chain alkyl groups C_10_–C_14_, which had MIC values in the range of 0.7–6.3 μM. For the Gram-negative pathogen *E. coli*, the MIC values were 11.6 μM, while the antifungal activity against *C. albicans* was moderate. The combined findings suggest that these compounds are promising candidates for further development. Mechanistic studies demonstrated membrane breakdown, along with an increase in ROS levels. After 17 passes, the hybrids remained active, demonstrating that resistance in *S. aureus* develops slowly. We hypothesize that the combination of membrane damage and potential oxidative stress contributes to the high efficacy and slow onset of resistance.

## Figures and Tables

**Table 1:** The minimum inhibitory concentration (MIC) and bactericidal/fungicidal (MBC/MFC) of the investigated compounds **6a**–**d**–**15a**–**d** against tested microbial strains.

R	n	Compounds	MIC/MBC (MFC)—μM						
			Gram-Positive			Gram-Negative		Fungi	
			*Staphylococcus aureus*	*Bacillus cereus*	*Enterococcus faecalis*	*Escherichia coli*	*Pseudomonas aeruginosa*	*Trichophyton mentagrophytes*	*Candida albicans*
**Me**	**2**	**6a**	13.5 ± 0.6/ 13.5 ± 0.6	27.0 ± 1.3/ 54.2 ± 2.5	54.2 ± 2.3/ 54.2 ± 2.4	216.3 ± 9/ 216.3 ± 10	-	-	-
		**7a**	3.1 ± 0.1/ 3.1 ± 0.1	12.9 ± 0.7/ 12.9 ± 0.6	12.9 ± 0.7/ 12.9 ± 0.6	51.7 ± 2.6/ 51.7 ± 2.5	206.3 ± 10/ 206.3 ± 11	-	206.3 ± 11/ 412.5 ± 20
		**8a**	3.0 ± 0.1/ 3.0 ± 0.1	3.0 ± 0.1/ 3.0 ± 0.1	1.4 ± 0.07/ 3 ± 0.1	24.6 ± 1.3/ 24.6 ± 1.4	98.6 ± 5.4/ 197.2 ± 10	197.2 ± 9/ 197.2 ± 11	24.6 ± 1.3/ 24.6 ± 1.2
		**9a**	**1.4 ± 0.07/** **1.4 ± 0.08**	**2.9 ± 0.1/** **2.9 ± 0.1**	**1.4 ± 0.07/** **1.4 ± 0.08**	24.3 ± 2.3/ 24.3 ± 2.5	-	-	23.6 ± 1.3/ 23.6 ± 1.4
		**10a**	22.6 ± 1.3/ 22.6 ± 1.4	181.2 ± 11/ 362.3 ± 18	181.2 ± 11/ 362.3 ± 20	-	-	-	-
	**3**	**11a**	26.4 ± 1.3/ 26.4 ± 1.4	52.9 ± 2.6/ 52.9 ± 2.5	52.9 ± 2.3/ 105.6 ± 5.4	211.1 ± 10/ 211.1 ± 9	-	-	-
		**12a**	3.1 ± 0.1/ 3.1 ± 0.1	6.3 ± 0.3/ 6.3 ± 0.3	6.3 ± 0.2/ 6.3 ± 0.2	25.2 ± 1.2/ 50.5 ± 2.6	201.6 ± 10/ 201.6 ± 11	201.6 ± 10/ 201.6 ± 11	201.6 ± 11/ 201.6 ± 10
		**13a**	6.3 ± 0.3/ 6.3 ± 0.3	6.3 ± 0.3/ 6.3 ± 0.2	3.1 ± 0.1/ 6.3 ± 0.3	50.6 ± 2.4/ 50.6 ± 2.5	201.9 ± 11/ 403.9 ± 19	201.9 ± 10/ 201.9 ± 11	50.6 ± 2.4/ 101.0 ± 5.5
		**14a**	**2.8 ± 0.1/** **2.8 ± 0.1**	5.8 ± 0.2/ 5.8 ± 0.2	5.8 ± 0.1/ 5.8 ± 0.3	46.3 ± 2.3/ 46.3 ± 2.2	-	-	46.3 ± 2.2/ 92.5 ± 5.3
		**15a**	11.1 ± 0.6/ 11.1 ± 0.5	11.1 ± 0.6/ 22.2 ± 1.2	11.1 ± 0.7/ 11.1 ± 0.6	177.6 ± 9/ 177.6 ± 11	-	-	355.1 ± 18/ 355.1 ± 20
**Et**	**2**	**6b**	26.4 ± 1.2/ 52.9 ± 2.4	26.4 ± 1.3/ 52.9 ± 2.5	26.4 ± 1.4/ 52.9 ± 2.5	211.1 ± 10/ 211.1 ± 9	-	-	-
		**7b**	**1.5 ± 0.08/** 3.1 ± 0.1	3.1 ± 0.1/ 6.3 ± 0.3	3.1 ± 0.1/ 3.1 ± 0.1	50.5 ± 2.6/ 50.5 ± 2.5	100.8 ± 5.1/ 100.8 ± 5.2	-	100.8 ± 5.4 /201.6 ± 11
		**8b**	**1.4 ± 0.07/** 2.9 ± 0.1	6.0 ± 0.5/ 12.0 ± 0.6	1.4 ± 0.06/ 2.9 ± 0.1	24.1 ± 1.2/ 24.1 ± 1.4	192.9 ± 11/ 385.8 ± 21	-	24.1 ± 1.3/ 24.1 ± 1.2
		**9b**	2.9 ± 0.1/ 2.9 ± 0.1	2.9 ± 0.1/ 5.9 ± 0.2	5.9 ± 0.3/ 5.9 ± 0.2			-	-
		**10b**	11.1 ± 0.7/ 11.1 ± 0.6	88.8 ± 5.3/ 355.1 ± 18	44.5 ± 2.3/ 44.5 ± 2.5	-	-	-	-
	**3**	**11b**	12.9 ± 0.6/ 51.7 ± 2.5	25.7 ± 1.3/ 51.7 ± 2.4	51.7 ± 2.6/ 51.7 ± 2.5	103.1 ± 5.2/ 103.1 ± 5.1	-	-	-
		**12b**	3.0 ± 0.5/ 6.2 ± 0.2	3.0 ± 0.5/ 12.3 ± 0.6	3.0 ± 0.5 /6.2 ± 0.2	49.4 ± 2.4 /49.4 ± 2.6	98.6 ± 5.3 /197.2 ± 10	98.6 ± 5.3/ 197.2 ± 11	98.6 ± 5.5/ 197.2 ± 9
		**13b**	**0.8 ± 0.1/** **1.5 ± 0.07**	**2.9 ± 0.1/** 5.9 ± 0.2	**1.5 ± 0.07/** **2.9 ± 0.1**	**11.8 ± 0.7/** **11.8 ± 0.6**	189 ± 10/ 189 ± 11	250 ± 19/ 250 ± 20	23.6 ± 1.3/ 23.6 ± 1.4
		**14b**	**2.8 ± 0.1/** 5.7 ± 0.3	11.3 ± 0.6/ 22.6 ± 1.2	**2.8 ± 0.1/** 2.8 ± 0.1	45.4 ± 2.4/ 45.4 ± 2.5	-	-	45.4 ± 2.3/ 45.4 ± 2.4
		**15b**	43.6 ± 2.3/ 43.6 ± 2.4	43.6 ± 2.6/ 43.6 ± 2.5	43.6 ± 2.4/ 43.6 ± 2.6	-	-	-	-
***^i^*** **Pr**	**2**	**6c**	25.7 ± 1.4/ 25.7 ± 1.3	103.1 ± 5.3/ 103.1 ± 5.1	103.1 ± 5.1/103.1 ± 5.1	412.5 ± 19/ 412.5 ± 21	-	-	-
		**7c**	2.9 ± 0.1/ 2.9 ± 0.1	5.9 ± 0.2/ 5.9 ± 0.3	5.9 ± 0.3/ 5.9 ± 0.2	23.5 ± 1.4/ 23.5 ± 1.3	377 ± 18/ 377 ± 19	-	189 ± 10/ 377 ± 20
		**8c**	**1.5 ± 0.07/** **1.5 ± 0.06**	**1.5 ± 0.06/** **1.5 ± 0.08**	**1.5 ± 0.1/** **1.5 ± 0.06**	**11.8 ± 0.7/** **11.8 ± 0.6**	378 ± 20/ 378 ± 19	-	47.3 ± 2.3/ 94.6 ± 5.4
		**9c**	**2.8 ± 0.1/** **2.8 ± 0.1**	5.7 ± 0.3/ 5.7 ± 0.2	5.7 ± 0.3/ 5.7 ± 0.3	90.6 ± 5.3/ 90.6 ± 5.2	-	-	90.6 ± 5.4/ 90.6 ± 5.3
		**10c**	10.9 ± 0.7/ 10.9 ± 0.6	21.7 ± 1.2/ 43.6 ± 2.5	43.6 ± 2.3/ 43.6 ± 2.2	-	-	-	-
	**3**	**11c**	12.6 ± 0.6/ 12.6 ± 0.7	12.6 ± 0.5/ 25.2 ± 1.4	25.2 ± 1.2/ 25.2 ± 1.3	100.8 ± 5.3/100.8 ± 5.4	-	-	-
		**12c**	**1.4 ± 0.07/** **1.4 ± 0.06**	**1.4 ± 0.07/** **1.4 ± 0.05**	**2.9 ± 0.1/** **2.9 ± 0.1**	24.1 ± 1.4/ 24.1 ± 1.3	96.5 ± 5.3/ 96.5 ± 5.2	-	48.3 ± 2.4/ 96.5 ± 5.3
		**13c**	**0.7 ± 0.04/** **1.3 ± 0.06**	5.8 ± 0.3/ 5.8 ± 0.2	**1.3 ± 0.07/** **2.8 ± 0.1**	**11.6 ± 0.7/** **11.6 ± 0.6**	370.4 ± 18/ 370.4 ± 19	-	**11.6 ± 0.6/** 23.1 ± 1.2
		**14c**	**1.3 ± 0.06/** **1.3 ± 0.06**	**2.7 ± 0.1/** **2.7 ± 0.1**	**1.3 ± 0.05/** **1.3 ± 0.06**	44.5 ± 2.3/ 44.5 ± 2.2	-	-	44.5 ± 2.4/ 88.9 ± 5.3
		**15c**	5.3 ± 0.2/ 5.3 ± 0.2	42.8 ± 2.3/ 42.8 ± 2.5	10.7 ± 0.7/ 10.7 ± 0.8	-	-	-	-
***^n^*** **Pr**	**2**	**6d**	3.1 ± 0.1/ 3.1 ± 0.1	6.4 ± 0.4/ 6.4 ± 0.3	6.4 ± 0.3/ 6.4 ± 0.2	103.1 ± 5.3/ 206.3 ± 9	412.5 ± 21/ 412.5 ± 19	-	412.5 ± 20/ 412.5 ± 19
		**7d**	**2.8 ± 0.1/** **2.8 ± 0.1**	5.7 ± 0.3/ 5.7 ± 0.2	**2.8 ± 0.1/** **2.8 ± 0.1**	90.6 ± 5.5/ 90.6 ± 5.3	-	362.3 ± 19/ 362.3 ± 20	90.6 ± 5.3/ 90.6 ± 5.4
		**10d**	10.9 ± 0.5/ 10.9 ± 0.6	21.7 ± 1.2/ 21.7 ± 1.3	10.9 ± 0.7/ 10.9 ± 0.6	10.9 ± 0.5/ 10.9 ± 0.6	21.7 ± 1.2/ 21.7 ± 1.3	10.9 ± 0.7/ 10.9 ± 0.6	10.9 ± 0.5/ 10.9 ± 0.6
	**3**	**11d**	12.6 ± 0.7/ 12.6 ± 0.5	25.2 ± 1.2/ 25.2 ± 1.3	50.5 ± 2.4/ 50.5 ± 2.2	403.2 ± 20/ 403.2 ± 21	-	-	-
		**12d**	**0.8 ± 0.04/** **1.5 ± 0.07**	**2.9 ± 0.1/** **2.9 ± 0.1**	**1.5 ± 0.05/** **1.5 ± 0.06**	24.1 ± 1.2/ 24.1 ± 1.3	48.3 ± 2.3/ 48.3 ± 2.24	386 ± 21/ 386 ± 19	48.3 ± 2.3/ 48.3 ± 2.5
		**13d**	**0.7 ± 0.04/** **0. 7 ± 0.03**	**2.8 ± 0.1/** **2.8 ± 0.1**	**2.8 ± 0.1/** **2.8 ± 0.1**	**11.6 ± 0.6/** 23.1 ± 1.3	370 ± 20/ 370 ± 19	-	**11.6 ± 0.7/** **11.6 ± 0.6**
		**14d**	**2.7 ± 0.1/** **2.7 ± 0.1**	11.1 ± 0.7/ 11.1 ± 0.6	**2.7 ± 0.1/** **2.7 ± 0.1**	355.1 ± 18/ 355.1 ± 19	-	-	88.8 ± 5.4/ 177.6 ± 11
		**15d**	10.7 ± 0.7/ 21.3 ± 1.4	21.3 ± 1.4/ 21.3 ± 1.3	5.3 ± 0.3/ 5.3 ± 0.3	-	-	-	-
**NF**			7.5 ± 0.2/ 7.5 ± 0.2	24.5 ± 0.5/ 24.5 ± 0.6	12.2 ± 0.3/ 12.2 ± 0.2	4.7 ± 0.1/ 24.5 ± 0.6	12.2 ± 0.4/ 48.9 ± 1.3		
**KK**								7.3 ± 0.3/ 7.3 ± 0.2	7.3 ± 0.3/ 7.3 ± 0.4

Average of three values measured; ± standard deviation (SD); - means non-active (MIC > 500 μM); NF—Norfloxacin; KK—Ketoconazole. Bold values correspond to the highest activity (MIC/MBC (MFC)): 0.7–2.8 μM for Gram-positive bacteria, 11.6 μM for *E. coli* and *C. albicans*.

**Table 2:** The minimum inhibitory (MIC) and bactericidal (MBC) concentrations of phosphonamidate–SHP/QAS hybrids against clinical isolates methicillin-resistant *Staphylococcus aureus* (MRSA).

Compounds	MIC/MBC—μM		
	*Staphylococcus aureus*	MRSA-1	MRSA-2
**7a**	3.1 ± 0.1/3.1 ± 0.1	6.4 ± 0.3/6.4 ± 0.2	12.9 ± 0.7/51.7 ± 2.4
**8a**	3.0 ± 0.1/3 ± 0.1	1.4 ± 0.07/1.4 ± 0.06	12.3 ± 0.5/49.4 ± 2.2
**9a**	1.4 ± 0.07/1.4 ± 0.08	1.4 ± 0.07/5.9 ± 0.3	47.3 ± 2.5/47.3 ± 2.3
**12a**	3.1 ± 0.1/3.1 ± 0.1	6.3 ± 0.2/12.6 ± 0.7	12.6 ± 0.7/25.2 ± 1.1
**13a**	6.3 ± 0.3/6.3 ± 0.3	1.5 ± 0.1/1.5 ± 0.1	25.2 ± 1.2/25.2 ± 1.3
**14a**	2.8 ± 0.1/2.8 ± 0.1	2.8 ± 0.1/5.8 ± 0.2	23.1 ± 1.2/23.1 ± 1.3
**7b**	1.5 ± 0.08/3.1 ± 0.1	6.3 ± 0.2/12.6 ± 0.6	3.1 ± 0.1/3.1 ± 0.1
**8b**	1.4 ± 0.07/2.9 ± 0.1	1.4 ± 0.07/2.9 ± 0.1	24.1 ± 1.2/24.1 ± 1.1
**9b**	2.9 ± 0.1/2.9 ± 0.1	1.4 ± 0.07/1.4 ± 0.06	47.3 ± 2.3/188.8 ± 11
**12b**	3.0 ± 0.5/6.2 ± 0.2	3.0 ± 0.5/6.2 ± 0.2	24.6 ± 1.2/24.6 ± 1.3
**13b**	0.8 ± 0.1/1.5 ± 0.07	1.5 ± 0.07/2.9 ± 0.1	23.6 ± 1.3/23.6 ± 1.2
**14b**	2.8 ± 0.1/5.7 ± 0.3	11.3 ± 0.7/11.3 ± 0.6	22.6 ± 1.2/22.6 ± 1.4
**7c**	2.9 ± 0.1/2.9 ± 0.1	5.9 ± 0.2/5.9 ± 0.3	11.8 ± 0.7/11.8 ± 0.7
**8c**	**1.5 ± 0.07/1.5 ± 0.06**	**1.4 ± 0.07/1.4 ± 0.06**	**2.9 ± 0.1/**11.8 ± 0.7
**9c**	2.8 ± 0.1/2.8 ± 0.1	5.7 ± 0.2/5.7 ± 0.3	90.6 ± 5.1/181.2 ± 9
**13c**	**0.7 ± 0.04/1.3 ± 0.06**	**2.8 ± 0.1/2.8 ± 0.1**	**2.8 ± 0.1/2.8 ± 0.1**
**14c**	1.3 ± 0.06/1.3 ± 0.06	5.5 ± 0.2/5.5 ± 0.3	11.1 ± 0.7/22.2 ± 1.4
**15c**	5.3 ± 0.2/5.3 ± 0.2	42.8 ± 2.3/170.8 ± 9	83.4 ± 5.2/170.8 ± 10
**6d**	3.1 ± 0.1/3.1 ± 0.1	12.9 ± 0.6/12.9 ± 0.7	25.7 ± 1.3/103.1 ± 5.3
**7d**	2.8 ± 0.1/2.8 ± 0.1	1.9 ± 0.1/5.7 ± 0.2	1.3 ± 0.05/2.8 ± 0.1
**12d**	**0.8 ± 0.04/1.5 ± 0.07**	**0.8 ± 0.04/1.4 ± 0.06**	**2.9 ± 0.1/2.9 ± 0.1**
**13d**	**0.7 ± 0.04/0. 7 ± 0.03**	**1.3 ± 0.07/2.8 ± 0.1**	5.8 ± 0.2/11.6 ± 0.6
**14d**	2.7 ± 0.1/2.7 ± 0.1	2.7 ± 0.1/5.5 ± 0.3	5.5 ± 0.2/22.2 ± 1.2
**CF**	1.5 ± 0.04/1.5 ± 0.03	377.2 ± 9/754.5 ± 19	2.7 ± 0.07/2.7 ± 0.06
**AC**	1.4 ± 0.04/2.5 ± 0.07	85.7 ± 2.1/85.7 ± 2.3	85.7 ± 2.4/85.7 ± 2.5

Average of three values measured; ± standard deviation (SD); - means non-active (MIC > 500 μM); CF—Norfloxacin; AC—Amoxicillin. Bold values indicate the most active compounds: MIC/MBC in the range of 0.7–2.9 μM.

**Table 3:** Evaluation of the cytotoxic effect of phosphonamidates on blood erythrocytes and Chang liver (M-HeLa) cells and their selectivity (SI) against *S. aureus* 209 P (Sa).

Compounds	HC_50_, μM	SI HC_50_/MIC*_Sa_*	IC_50_, μM	SI IC_50_/MIC*_Sa_*
**7a**	41.3 ± 3.7	13	68.8 ± 5.4	22
**8a**	**276.0 ± 17**	**92**	61.2 ± 5.1	20
**9a**	55.9 ± 4.3	41	62.7 ± 4.9	46
**12a**	153.2 ± 1.2	50	57.9 ± 4.9	19
**13a**	86.4 ± 7.8	14	52.9 ± 3.9	9
**14a**	81.4 ± 6.4	29	67.0 ± 5.5	24
**7b**	11.3 ± 0.9	8	57.9 ± 4.4	40
**8b**	69.4 ± 5.5	50	74.1 ± 6.5	53
**9b**	44.4 ± 3.8	16	60.1 ± 4.7	21
**12b**	107.3 ± 8.4	36	58.8 ± 3.8	20
**13b**	**58.9 ± 4.7**	**78**	**59.1 ± 4.5**	**78**
**14b**	50.7 ± 3.6	18	57.0 ± 3.7	21
**7c**	177.3 ± 1.5	59	66.4 ± 5.3	22
**8c**	60.4±4.8	44	69.9 ± 3.5	51
**9c**	56.7 ± 3.9	21	71.0 ± 6.7	26
**12c**	64.8 ± 5.2	47	65.0 ± 5.3	47
**13c**	**45.9 ± 3.7**	**62**	**66.4 ± 3.5**	**90**
**14c**	38.5 ± 2.8	30	36.6 ± 2.4	29
**15c**	41.0 ± 2.9	8	55.1 ± 4.7	10
**7d**	39.1 ± 2.1	14	35.1 ± 1.8	13
**12d**	**66.4 ± 5.4**	**86**	**67.4 ± 4.6**	**87**
**13d**	26.6 ± 1.9	36	50.0 ± 3.3	68
**14d**	32.7 ± 2.7	12	67.0 ± 5.6	25
**15d**	**219.5 ± 21**	**70**	69.0 ± 4.2	22
**CTAB**	7.1 ± 0.6	3.5 ± 0.6	14.3 ± 1.1	7.0

CTAB—Cetyltrimethylammonium bromide. The experiments were repeated for three times. The results are expressed as the mean ± standard deviation (SD). Bold values indicate the most favourable safety parameters: high selectivity index (SI) and high HC_50_ or IC_50_ values.

## Data Availability

The original contributions presented in this study are included in the article/supplementary material. Further inquiries can be directed to the corresponding authors.

## Supplementary Materials

The following supporting information can be downloaded at: https://www.mdpi.com/article/10.3390/ijms27104524/s1.
