# Supplementary material for: Phosphonamidates Integrating Sterically Hindered Phenols with Membrane-Active Cations: A Redox-Activated Approach to Antimicrobial Agents
Source: Int J Mol Sci. 2026 May 18;27(10):4524. doi: 10.3390/ijms27104524 (PMC13207327; doi:10.3390/ijms27104524)
Supplement: Supplementary file 1 [file ijms-27-04524-s001.zip › ijms-4282114 - Supplementary Materials.pdf]

Supporting Information

Phosphonamidates Integrating Sterically Hindered Phenols with Membrane-Active Cations: A Redox-Activated Approach to Antimicrobial Agents

Elmira Gibadullina [1,2,*], Adel Shakirov [1], Margarita Neganova [2,3], Yulia Aleksandrova [2,3], Alexandra Voloshina [1], Anna Lyubina [1], Anastasiya Sapunova [1], Anna Strelnik [1], Kamil Ivshin [1], Assel Shuragaziyeva [2], Altynkul Toibazarova [2], Banu Diyarova [4], Anipa Tapalova [2,*], Nurbol Appazov [2] and Alexander Burilov [1]

[1] Arbuzov Institute of Organic and Physical Chemistry, FRC Kazan Scientific Center, Russian Academy of Sciences, Akad. Arbuzov St. 8, Kazan 420088, Russia; shakirov-adel@inbox.ru (A.S.); microbi@iopc.ru (A.V.); burilov_2004@mail.ru (A.B.)

[2] Laboratory of Engineering Profile "Physical and Chemical Methods of Analysis", Korkyt Ata Kyzylorda University, 29A, Aiteke Bi Street, Kyzylorda 120014, Kazakhstan; nurasar.82@korkyt.kz (N.A.)

[3] A.N. Nesmeyanov Institute of Organoelement Compounds of Russian Academy of Sciences, Vavilova St. 28, Bld. 1., Moscow 119334, Russia; neganovam@ipac.ac.ru (M.N.); yulia.aleks.97@mail.ru (Y.A.)

[4] Department of Chemistry, Institute of Natural Science, Kazakh National Women's Teacher Training University, 144/8 Gogol Street, Almaty 050000, Kazakhstan

* Correspondence: elmirak@iopc.ru (E.G.); anipa52@mail.ru (A.T.); Tel.: +7-843-272-7324 (E.G.)

Contents

Compound characterization data S2

The X-ray diffraction data S27

NMR spectra of compounds S29

Stability of compounds 8c, 12c, and 13c in biological media S56

Cytotoxicity data S58

References S599

Compound characterization data

The synthesis of phosphonamidate–SHP/QAS hybrids is summarized in Scheme S1.

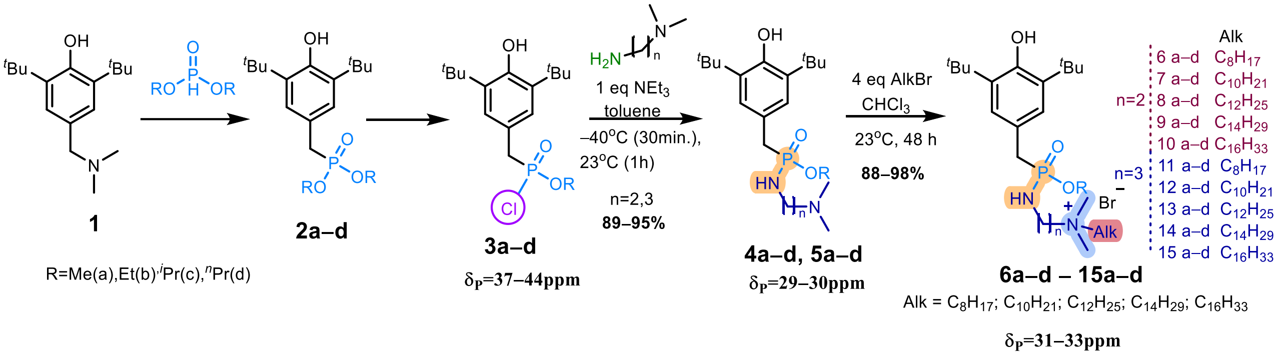

Scheme S1. The synthesis of phosphonamidate–SHP/QAS hybrids

The ^{1}H- and ^{13}C-NMR spectra were recorded on a Bruker AVANCE 400 spectrometer (Bruker BioSpin, Rheinstetten, Germany) operating at 400 MHz (for ^{1}H NMR), 101 MHz (for ^{13}C NMR) and 162 MHz (for ^{31}P NMR); Brucker spectrometers AVANCEIII-500 (Bruker Corporation, Rheinstetten, Germany) operating at 500 MHz (for ^{1}H NMR) and 126 MHz (for ^{13}C NMR); Bruker spectrometers AVANCEIII-600 (Bruker Corporation, Rheinstetten, Germany) operating at 600.13 MHz (for ^{1}H NMR), 150.19 MHz (for ^{13}C NMR) and 242.94 MHz (for ^{31}P NMR). Chemical shifts are given relative to the residual signals of the deuterated solvent ($CDCl_3$ δ(1H) = 7.26 ppm and δ(^{13}C) = 77.0 ppm; d_6-acetone δ(^{1}H) = 2.09 ppm and δ(^{13}C) = 30.6 ppm, 206.2 ppm).

IR spectra were recorded on IR Fourier spectrometer Tensor 37 (Bruker Optik GmbH, Germany) in the 400–3600 cm^{-1} range in nujol or as a thin film.

Electrospray ionization mass spectra (ESI-MS) were obtained on an AmazonX ion trap mass spectrometer (Bruker Daltonik GmbH, Bremen, Germany). The measurements were performed in positive ion mode over a mass range of *m*/*z* 100–2800. The capillary voltage was set to –4500 V. Nitrogen was used as the drying gas at a temperature of 250 °C and a flow rate of 8 L/min. A methanol/water mixture (70:30) was used as the eluent at a flow rate of 0.2 mL/min, delivered by an Agilent 1260 chromatograph (USA). The compounds were dissolved in methanol at a concentration of 10^{-6} g/L. The injection volume was 20 μL. Data processing was performed using DataAnalysis 4.0 software (Bruker Daltonik GmbH, Bremen, Germany).

Elemental analysis was performed on a CHNS-O Elemental Analyser EuroEA3028-HT-OM (EuroVector S.p.A., Milan, Italy). The melting points were determined on JK-MAM-4 Meltingpoint Apparatus with Microscope (SGW-X4 JINGKE SCIENTIFIC INSTRUMENT CO, Shanghai, China).

The progress of reactions and the purity of products were monitored by TLC on Sorbfil UV-254 plates (Sorbpolimer, Krasnodar, Russia); the chromatograms were developed under UV light.

The reagents and solvents used for all activities presented in the research were purchased from local suppliers. Dialkyl (3,5-di-*tert*-butyl-4-hydroxybenzyl)phosphonate **2a-d** and *O*-alkyl (3,5-di-*tert*-butyl-4-hydroxybenzyl)phosphonochloridate **3a-d** was synthesized according to the literature [1, 2].

General Procedure for the Synthesis of Phosphonamidate Hybrids SHP/QAS 6a-d – 15a-d

Synthesis of Phosphonamidate Precursors 4a-d and 5a-d. To a 2 mL abs.toluene of solution with the corresponding *N,N*-dimethylethylenediamine or *N,N*-dimethylpropylenediamine (1.0 mmol) and triethylamine (1.0 mmol), cooled in a liquid nitrogen bath to –40°C at this temperature for 10-15 min and 2 mL abs.toluene with alkyl (3,5-di-*tert*-butyl-4-hydroxybenzyl)phosphonochloridate (1.0 mmol) **3a-d** in was added dropwise. The mixture was stirred magnetically for 2 h without cooling, allowing it to equilibrate to 23°C. After the precipitate of triethylamine hydrochloride were filtered off, the filtrate was concentrated on a rotary evaporator. The resulting solid were washed with distilled water containing diethylamine (10%) to remove hydrolysis and pyrophosphonate byproducts, followed by hexane, and dried under vacuum (0.06 mm Hg) at 40 °C to constant weight. The intermediate phosphonamidates were obtained as light-colored powders in high yields.

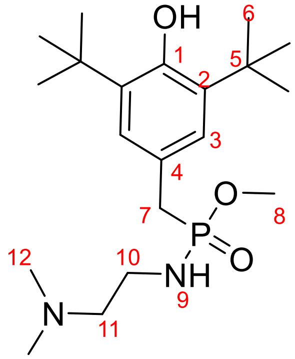

Methyl-*P*-(3,5-di-*tert*-butyl-4-hydroxyphenyl)-*N*-(2-(dimethylamino)propyl)phosphonamidate 4a: gray-white solid, yield 0.08 g (85%), M.p.: 88–89°C. IR (nujol), *ν*, cm $^{-1}$: 1061 (P–O–C_{alk}), 1353 (P=O), 1624 (C=C_{Ar}), 3772 (OH). ^{1}H NMR (600 MHz, $CDCl_3$, δ, ppm., *J*/Hz): 1.45 s (18H, CH_3), 2.18 s (6H, $N(CH_3)_2$), 2.35 m (2H, C$\underline{H}_2$$CH_2$NH), 2.92 br m (2H, CH_2C$\underline{H}_2$NH), 3.06 d (2H, CH_2, J_{PH} 20), 3.45 br s (1H, NH), 3.66 m (3H, OCH_3), 5.2 s (1H, OH), 7.1 s (2H, CH_{Ar}). ^{31}P NMR (243 MHz, $CDCl_3$), δ, ppm: 31.4. Elemental analysis. Found, %: C, 62.64; H 9.31; N 7.45; P 7.98. $C_{20}H_{37}N_2O_3P$. Calculated, %:C, 62.48; H, 9.70; N, 7.29; P, 8.06. MS (ESI-TOF), *m/z* (*rel. int.*, %): Found, 385.1 $[M+H]^+$.

Ethyl-*P*-(3,5-di-*tert*-butyl-4-hydroxyphenyl)-*N*-(2-(dimethylamino)ethyl)phosphonamidate 4b: off-white solid, yield 0.11 g (92%), M.p.: 88-90°C. IR (nujol), *ν*, cm $^{-1}$: 1079 ($P–O–C_{alk}$), 1323 (P=O), 1601 ($C=C_{Ar}$), 3628 (OH). ^{1}H NMR (600 MHz, $CDCl_3$, δ, ppm., *J*/Hz): 1.27 t (3H, $OCH_2\text{-}C\underline{H}_3$, J_{HH} 6.8), 1.41 s (18H, CH_3), 2.13 s (6H, $N(CH_3)_2$), 2.26 m (2H, CH_2CH_2NH), 2.87 m (2H, CH_2CH_2NH), 3.03 d (2H, CH_2, J_{PH} 20), 3.13 br s (1H, NH), 3.96-4.05 m (2H, $OC\underline{H}_2\text{-}CH_3$), 5.13 s (1H, OH), 7.10 s (2H, CH_{Ar}). ^{13}C NMR (151 MHz, $CDCl_3$, δ, ppm, *J*/Hz): 16.4 (C^9), 30.3 (C^6), 34.2 (C^5), 35.6 (C^7, J_{PC} 126.0), 38.1 (C^{11}), 44.9 (C^{14}), 59.7 (C^{12}), 59.9 (C^8), 123.1 (C^4), 126.1 (C^3), 136.0 (C^2), 152.5 (C^1). ^{31}P NMR (243 MHz, $CDCl_3$, δ, ppm, *J*/Hz): 30.7. Elemental analysis. Found, %: C, 63.21; H 10.07; N 7.15; P 7.41. $C_{21}H_{39}N_2O_3P$. Calculated, %:C, 63.29; H, 9.86; N, 7.03; P, 7.77. MS (ESI-TOF), *m/z* (*rel. int.*, %): Found, 400.1 $[M+H]^+$.

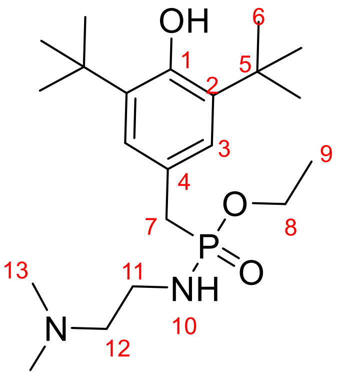

***iso*-Propyl-*P*-(3,5-di-*tert*-butyl-4-hydroxyphenyl)-*N*-(2-(dimethylamino)ethyl) phosphonamidate 4c:** gray-white solid, yield 0.095 g (95%), M.p.: 92–93°C. IR (nujol), *ν*, cm $^{-1}$: 1119 ($P–O–C_{alk}$), 1418 (P=O), 1589 ($C=C_{Ar}$), 36332 (OH). ^{1}H NMR (400 MHz, $CDCl_3$, δ, ppm., *J*/Hz): 1.27 dd (6H, $OCH\text{-}(C\underline{H}_3)_2$, J_{HH} 6.2), 1.42 s (18H, CH_3), 2.14 s (6H, $N(CH_3)_2$), 2.29 t (2H, $C\underline{H}_2CH_2NH$, J_{HH} 6.0), 2.88 m (2H, $C\underline{H}_2CH_2N$), 3.01 d (2H, CH_2, J_{PH} 20.0), 3.15 br s (1H, NH), 3.96-4.05 s (1H, $OC\underline{H}\text{-}(CH_3)_2$), 5.14 s (1H, OH), 7.09 s (2H, CH_{Ar}). ^{13}C NMR (101 MHz, $CDCl_3$, δ, ppm, *J*/Hz): 24.1-24.3 (C^9), 30.3 (C^6), 34.2 (C^5), 35.1-36.3 (C^7, J_{PC} 126.0), 38.1 (C^{11}), 44.9 (C^{14}), 59.9 (C^{12}), 68.0 (C^8), 123.1 (C^4), 126.3 (C^3), 136.0 (C^2), 152.5 (C^1). ^{31}P NMR (243 MHz, $CDCl_3$, δ, ppm, *J*/Hz): 29.6. Elemental analysis. Found, %: C, 64.01; H 10.09; N 6.30; P 7.37. $C_{22}H_{41}N_2O_3P$. Calculated, %:C, 64.05; H, 10.02; N, 6.79; P, 7.51. MS (ESI-TOF), *m/z* (*rel. int.*, %): Found, 413.15 $[M+H]^+$.

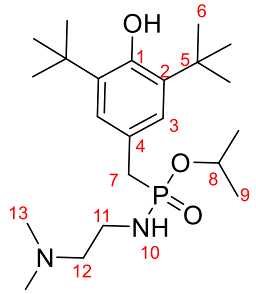

Propyl-*P*-(3,5-di-*tert*-butyl-4-hydroxyphenyl)-*N*-(2-(dimethylamino)ethyl)phosphonamidate 4d: gray-white solid, yield 0.12 g (94%), M.p.: 91-93°C. IR (nujol), *ν*, cm $^{-1}$: 1078 ($P–O–C_{alk}$), 1121 (C–O–C), 1304 (P=O), 1595 ($C=C_{Ar}$), 3628 (OH). ^{1}H NMR (500 MHz, $CDCl_3$, δ, ppm., *J*/Hz): 0.85 m (3H, $OCH_2CH_2C\underline{H}_3$), 1.33 s (18H, CH_3), 1.53 m (2H, $OCH_2C\underline{H}_2CH_3$), 2.09 s (6H, $N(CH_3)_2$), 2.26 m (2H, $C\underline{H}_2CH_2NH$), 2.84 br m (2H, $CH_2C\underline{H}_2NH$), 2.99 d (2H, CH_2, J_{PH}

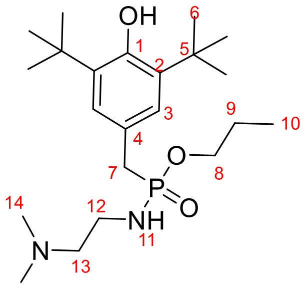

20.0), 3.52 br s (1H, NH), 3.85 br m (2H, OC$\underline{H}_2$CH$_2$CH$_3$), 5.26 br s (1H, OH), 7.03 s (2H, CH$_{Ar}$). ^{13}C NMR (126 MHz, CDCl$_3$, δ, ppm, *J*/Hz): 9.6 (C^{10}), 23.2 (C^9), 29.8 (C^6), 33.7 (C^5), 35.2 (C^7, J_{PC} 126.2), 37.2 (C^{12}), 44.0 (C^{14}), 59.2 (C^{13}), 64.6 (C^8), 122.5 (C^4), 125.7 (C^3), 135.6 (C^2), 152.0 (C^1). ^{31}P NMR(243 MHz, CDCl$_3$, δ, ppm, *J*/Hz): 31.2. Elemental analysis. Found, %: C, 64.18; H 10.14; N 6.56; P 7.35. $C_{22}H_{41}N_2O_3P$. Calculated, %:C, 64.05; H, 10.02; N, 6.79; P, 7.51. MS (ESI-TOF), *m/z* (*rel. int.*, %): Found, 413.5 [M+H]$^+$.

Methyl-*P*-(3,5-di-*tert*-butyl-4-hydroxyphenyl)-*N*-(3-(dimethylamino)propyl)phosphonamidate 5a: gray-white solid, yield 0.1 g (90%), M.p.:

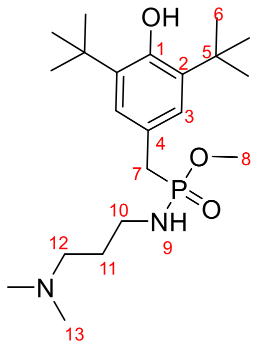

86-87°C. IR (nujol), ν, cm^{-1}: 1053 (P–O–C$_{alk}$), 1304 (P=O), 1598 (C= C$_{Ar}$), 3678 (OH). ^{1}H NMR (400 MHz, CDCl$_3$, δ, ppm., *J*/Hz): 1.41 s (18H, CH$_3$), 1.53 quint (2H, CH$_2$C$\underline{H}_2$CH$_2$NH, J_{HH} 8.0), 2.11 s (6H, N(CH$_3$)$_2$), 2.28 t (2H, C$\underline{H}_2$CH$_2$CH$_2$NH, J_{HH} 6.4), 2.91 m (2H, CH$_2$CH$_2$C$\underline{H}_2$NH), 3.00 d (2H, CH$_2$, J_{PH} 19.9), 3.32 br s (1H, NH), 3.62 d (3H, OCH$_3$, J_{PH} 10.9), 5.11 s (1H, OH), 7.06 s (2H, CH$_{Ar}$, J_{PH} 2.4). ^{13}C NMR(101 MHz, CDCl$_3$, δ, ppm, *J*/Hz): 30.3 (C^{11}), 30.3 (C^6), 34.8 (C^5), 35.3 (C^7, J_{PC} 125.2), 37.8 (C^{12}), 44.6 (C^{13}), 50.6 (C^8), 59.7(C^{10}), 122.8 (C^4), 126.2 (C^3), 136.3 (C^2), 152.6 (C^1). ^{31}P NMR(243 MHz, CDCl$_3$, δ, ppm, *J*/Hz): 30.7. Elemental analysis. Found, %: C, 62.61; H 9.14; N 7.12; P 7.213. $C_{21}H_{39}N_2O_3P$. Calculated, %:C, 62.39; H, 9.86; N, 7.03; P, 7.77. MS (ESI-TOF), *m/z* (*rel. int.*, %): Found, 400.3 [M+H]$^+$.

Ethyl-*P*-(3,5-di-*tert*-butyl-4-hydroxyphenyl)-*N*-(3-(dimethylamino)propyl)phosphonamidate 5b: off-white solid, yield 0.08 g (87%)g, M.p.:

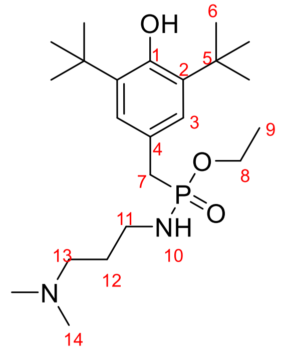

89-91°C. IR (nujol), *ν*, cm $^{-1}$: 1074 (P–O–C$_{alk}$), 1257 (P=O), 1566 (C= C$_{Ar}$), 3711 (OH). ^{1}H NMR(400 MHz, CDCl$_3$, δ, ppm., *J*/Hz): 1.29 t (3H, OCH$_2$-C$\underline{H}_3$, J_{HH} 7.1), 1.44 s (18H, CH$_3$), 1.55 m (2H, CH$_2$C$\underline{H}_2$CH$_2$NH), 2.14 s (6H, N(CH$_3$)$_2$), 2.28 t (2H, CH$_2$CH$_2$CH$_2$NH, J_{HH} 7.1), 2.91 m (2H, CH$_2$CH$_2$CH$_2$NH), 3.06 d (2H, CH$_2$, J_{PH} 19.9), 3.23 br s (1H, NH), 3.95-4.11 m (2H, OC$\underline{H}_2$-CH$_3$), 5.13 s (1H, OH), 7.10 d (2H, CH$_{Ar}$, J_{PH} 2.3). ^{13}C NMR(101 MHz, CDCl$_3$, δ, ppm, *J*/Hz): 16.5 (C^9), 28.9 (C^{12}), 30.3 (C^6), 34.2 (C^5), 35.5 (C^7, J_{PC} 125.2), 40.3 (C^{11}), 45.4 (C^{15}), 58.0 (C^{13}), 59.6 (C^8), 123.1 (C^4), 126.3 (C^3), 135.9 (C^2), 152.5 (C^1). ^{31}P NMR (243 MHz, CDCl$_3$, δ, ppm, *J*/Hz): 30.4. Elemental analysis. Found, %: C, 63.94; H 10.17; N 6.49; P 7.27. $C_{22}H_{41}N_2O_3P$. Calculated, %:C, 64.05; H, 10.02; N, 6.79; P, 7.51. MS (ESI-TOF), *m/z* (*rel. int.*, %): Found, 413.1 [M+H]$^+$.

iso-Propyl-P-(3,5-di-tert-butyl-4-hydroxyphenyl)-N-(3-(dimethylamino)propyl)phosphonamidate 5c: gray-white solid, yield 0.11 g (91%), M.p.: 97-99°C. IR (nujol), ν, cm $^{-1}$: 1078 (P–O–C_{alk}), 1304 (P=O), 1595 (C=Carom), 3628 (OH). ^{1}H NMR (400 MHz, $CDCl_3$, δ, ppm., *J*/Hz): 1.32 dd (6H, OCH-(C<u>H</u>$_3$)$_2$, J_{HH} 9.7, 6.2), 1.43 s (18H, CH_3), 1.55 m (2H, CH_2C<u>H</u>$_2$$CH_2$NH), 2.14 s (6H, N$(CH_3)_2$), 2.28 t (2H, $CH_2CH_2CH_2$NH, J_{HH} 8.0), 2.93 m (2H, CH_2CH_2C<u>H</u>$_2$NH), 3.02 d (2H, CH_2, J_{PH} 20.0), 3.24 br s (1H, NH), 4.64-4.72 m (1H, OC<u>H</u>$(CH_3)_2$), 5.11 s (1H, OH), 7.11 d (2H, $CH_{Ar,}$ J_{PH} 2.5). ^{13}C NMR (101 MHz, $CDCl_3$, δ, ppm, *J*/Hz): 24.2 (C^9), 28.8 (C^{12}), 30.3 (C^6), 34.2 (C^5), 34.8 (C^7, J_{PC} 126.2), 40.4 (C^{11}), 45.3 (C^{15}), 58.0 (C^{13}), 68.3 (C^8), 123.4 (C^4), 126.3 (C^3), 135.8 (C^2), 152.4 (C^1). ^{31}P NMR (243 MHz, $CDCl_3$, δ, ppm, *J*/Hz): 30.4. Elemental analysis. Found, %: C, 64.97; H 10.21; N 6.39; P 7.31. $C_{23}H_{43}N_2O_3P$. Calculated, %:C, 64.76; H, 10.16; N, 6.57; P, 7.26. MS (ESI-TOF), *m/z* (*rel. int.*, %): Found, 427.34 $[M+H]^+$.

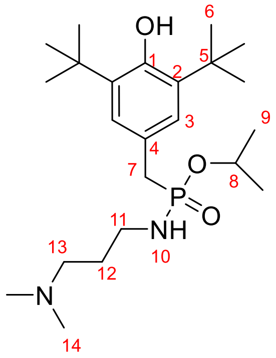

Propyl-P-(3,5-di-tert-butyl-4-hydroxyphenyl)-N-(3-(dimethylamino)propyl)phosphonamidate 5d: gray-white solid, yield 0.08 g (87%), M.p.: 95-96°C. ^{1}H NMR (500 MHz, $CDCl_3$, δ, ppm., *J*/Hz): 0.89 dt (3H, OCH_2CH_2C<u>H</u>$_3$, J_{HH} 7.4, J_{PH} 1.7), 1.40 s (18H, CH_3), 1.51 m (2H, OCH_2C<u>H</u>$_2$$CH_3$), 1.62 m (2H, CH_2C<u>H</u>$_2$$CH_2$NH), 2.11 s (6H, N$(CH_3)_2$), 2.27 t (2H, C<u>H</u>$_2$$CH_2CH_2$NH, J_{HH} 7.5), 2.89 m (2H, CH_2CH_2C<u>H</u>$_2$NH), 3.01 d (2H, $CH_{2,}$ J_{PH} 19.9), 3.31 br s (1H, NH), 3.80–3.94 m (2H, OC<u>H</u>$_2$$CH_2CH_3$), 5.17 s (1H, OH), 7.06 d (2H, $CH_{Ar,}$ J_{PH} 2.5). ^{13}C NMR (126 MHz, $CDCl_3$, δ, ppm, *J*/Hz): 9.67 (C^{10}), 23.3 (C^9), 28.2 (C^{12}), 29.7 (C^6), 33.7 (C^5), 34.9 (C^7, J_{PC} 126.0), 39.5 (C^{11}), 44.6 (C^{14}), 57.2 (C^{13}), 64.6 (C^8), 122.6 (C^4), 125.7 (C^3), 135.5 (C^2), 152.7 (C^1). ^{31}P NMR (243 MHz, $CDCl_3$, δ, ppm, *J*/Hz): 31.2. Elemental analysis. Found, %: C, 64.40; H 9.98; N 6.29; P 7.12. $C_{23}H_{43}N_2O_3P$. Calculated, %:C, 64.76; H, 10.16; N, 6.57; P, 7.26. MS (ESI-TOF), *m/z* (*rel. int.*, %): Found, 428.3 $[M+H]^+$.

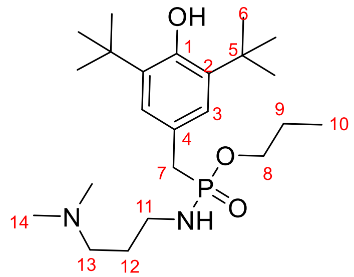

General Procedure for Quaternary Ammonization (Alkylation) f Phosphonamidates 6a-d – 15a-d. To a 5 mL chloroform solution the phosphonamidate (1.0 mmol) and corresponding bromoalkanes (4.0 mmol) were added. The reaction mixture was stirred for 48 h at room temperature (23°C), and the reaction progress was monitored by TLC. After the chloroform was removed on a rotary evaporator. The resulting crude product was washed with hexane (3 × 5 mL) and dried under vacuum

(0.06 mm Hg) at 40 °C to constant weight. The resulting products **6a-d** – **15a-d** were obtained as light yellow to brown oils in high yields.

***N*-(2-(((3,5-di-*tert*-butyl-4-hydroxybenzyl)(methoxy)phosphoryl)amino)ethyl)-*N,N*-dimethyloctan-1-aminium bromide 6a**: Yield 0.11g (87%). ^{1}H NMR(500 MHz, $CDCl_3$, δ, ppm., *J*/Hz): 0.74 br s (3H, CH_3), 1.20 br m (10H, CH_2), 1.30 s (18H, CH_3), 1.55 br m (2H, CH_2), 2.95 – 3.06 br m (2H, CH_2CH_2NH and 2H, PCH_2), 3.24 br s (6H, $N(CH_3)_2$), 3.40 br m (4H, CH_2), 3.51 br d (3H, OCH_3, J_{PH} 11), 4.90 br s (1H, OH), 7.00 s (2H, CH_{Ar}). ^{13}C NMR (126 MHz, $CDCl_3$, δ, ppm, *J*/Hz): 13.9 (C^{19}), 22.4 (C^{18}), 22.6 (C^{13}), 26.1 (C^{14}), 29.2 ($C^{15,16}$), 30.3 (C^6), 31.4 (C^{17}), 34.1 (C^5), 34.7 (C^7), 5.1 (C^9), 51.4 (C^{11}), 51.6 (C^8), 64.4 (C^{12}), 65.3 (C^{10}), 122.2 (C^4), 126.5 (C^3), 136.4 (C^2), 152.8 (C^1). ^{31}P NMR (243 MHz, $CDCl_3$, δ, ppm, *J*/Hz): 33.2. Elemental analysis. Found, %: C, 58.42; H, 9.52; Br, 13.93; N, 4.63; P, 5.48. $C_{28}H_{54}N_2O_3PBr$. Calculated, %:C, 58.22; H, 9.42; Br, 13.83; N, 4.83; P, 5.36. MS (ESI-TOF), *m*/*z* (*rel. int.*, %): Found, 498.4 $[M–Br]^+$. Calculated for the bromide salt: 578.2 for $[M+H]^+$.

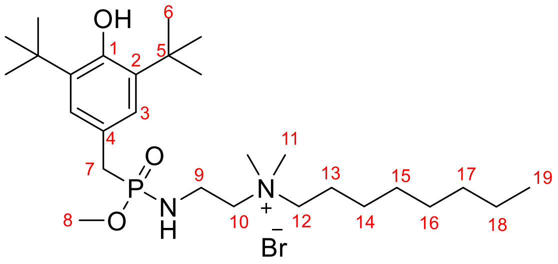

***N*-(2-(((3,5-di-*tert*-butyl-4-hydroxybenzyl)(ethoxy)phosphoryl)amino)ethyl)-*N,N*-dimethyloctan-1-aminium bromide 6b:** Yield 0.12 g (91%). IR, ν, cm $^{-1}$: 924 (P-N), 1014 (P–O–C_{alk}), 1321 (P=O), 1391-1467 (C_8H_{17}), 1621 (C=C_{Ar}), 3633 (OH). ^{1}H NMR(500 MHz, $CDCl_3$, δ, ppm., *J*/Hz): 0.87 br s (3H, CH_3), 1.17 br s (10H, CH_2), 1.24 br m (3H, CH_3), 1.44 s (18H, CH_3), 1.67 br m (2H, CH_2), 3.03 br m (2H, CH_2CH_2NH), 3.08 br m (2H, CH_2), 3.28 s (6H, $N(CH_3)_2$,), 3.41 br m (2H, CH_2), 3.76 br m (2H, CH_2), 3.90 br m (2H, OCH_2), 4.85 br s (1H, NH), 7.14 s (2H, CH_{Ar}). ^{13}C NMR(126 MHz, $CDCl_3$, δ, ppm, *J*/Hz): 14.5 (C^{21}), 16.9 (C^9), 23.1 (C^{20}), 23.4 (C^{15}), 26.7 (C^{16}), 29.8 (C^{17-18}), 30.9 (C^6), 32.3 (C^{19}), 34.2 (C^5), 34.8 (C^7, J_{PC} 123.5), 43.9 (C^{11}), 52.1 (C^{13}), 61.4 (C^8), 65.5 (C^{14}), 66.4 (C^{12}), 123.0 (C^4), 127.2 (C^3), 136.6 (C^2), 153.2 (C^1). ^{31}P NMR(243 MHz, $CDCl_3$, δ, ppm, *J*/Hz): 32.5. Elemental analysis. Found, %: C, 59.12; H, 9.79; Br, 13.21; N, 4.84; P, 5.45. $C_{29}H_{56}N_2O_3PBr$. Calculated, %:C, 58.87; H, 9.54; Br, 13.51; N, 4.73; P, 5.24. MS (ESI-TOF), *m*/*z* (*rel. int.*, %): Found, 512.7 $[M–Br]^+$. Calculated for the bromide salt: 592.6 $[M+H]^+$.

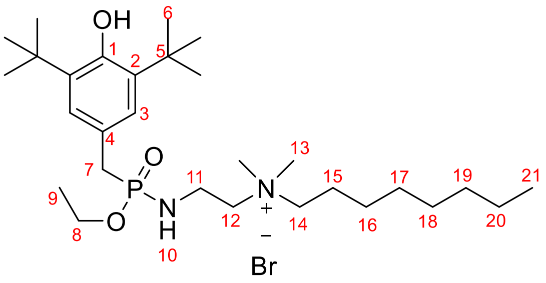

N-(2-(((3,5-di-*tert*-butyl-4-hydroxybenzyl)(*iso*-propoxy)phosphoryl)amino)ethyl)-*N*,*N*-dimethyloctan-1-aminium bromide 6c: Yield 0.09 g(80%). IR, ν, cm $^{-1}$: 998 (P-N), 1257 (P–O–C_{alk}), 1377 (P=O), 1629 (C=C_{Ar}), 3611 (OH).

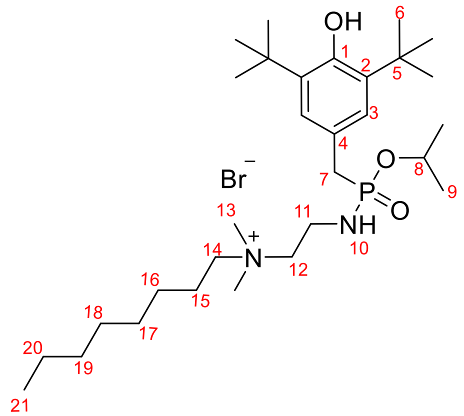

^{1}H NMR (500 MHz, $CDCl_3$, δ, ppm., *J*/Hz): 0.86 br t (3H, CH_3, J_{HH} 7.3), 1.15 d (3H, CH_3, J_{PH} 5.0), 1.25 br m (10H, CH_2), 1.33 br m (3H, CH_3), 1.40 s (18H, CH_3), 1.68 br m (2H, CH_2), 2.72 br m (2H, CH_2CH_2NH), 3.04 d (2H, CH_2, J_{PH} 19.4), 3.24 br s (6H, $N(CH_3)_2$), 3.37 m (4H, CH_2), 3.63 br s (1H, NH), 4.52 br m (1H, CH), 7.13 s (2H, CH_{Ar}). ^{13}C NMR (126 MHz, $CDCl_3$, δ, ppm, *J*/Hz): 14.6 (C^{21}), 23.1 (C^{20}), 24.6 (C^9), 26.7 (C^{15}), 29.6 (C^{16}), 29.8 (C^{17-18}), 30.9 (C^6), 32.3 (C^{19}), 34.8 (C^5), 35.5 (C^7, J_{PC} 124.7), 44.0 (C^{11}), 51.9 (C^{13}), 65.4 (C^{12}), 66.2 (C^{14}), 69.8 (C^8), 123.3 (C^4), 127.2 (C^3), 136.6 (C^2), 153.1 (C^1). ^{31}P NMR(243 MHz, $CDCl_3$, δ, ppm, *J*/Hz): 31.2. Elemental analysis. Found, %: C, 59.77; H, 10.01; Br, 12.81; N, 4.72; P, 5.47. $C_{30}H_{58}N_2O_3PBr$. Calculated, %:C, 59.49; H, 9.65; Br, 13.19; N, 4.63; P, 5.11. MS (ESI-TOF), *m/z* (*rel. int.*, %): Found, 525.3 [M–Br]$^+$. Calculated for the bromide salt: 605.6 [M+H]$^+$.

N-(2-(((3,5-di-*tert*-butyl-4-hydroxybenzyl)(propooxy)phosphoryl)amino)ethyl)-*N*,*N*-dimethyloctan-1-aminium bromide 6d: Yield 0.13 g (90%). ^{1}H NMR(500 MHz, $CDCl_3$, δ, ppm., *J*/Hz): 0.84 t (6H, CH_3 and C<u>H</u>$_3$$CH_2$, J_{HH} 6.3), 1.26 m (10H, CH_2), 1.39 s (18H, CH_3), 1.56 m (2H, CH_3C<u>H</u>$_2$$CH_2O$), 1.67 m (2H, CH_2), 2.99 br m (2H, CH_2CH_2NH), 3.05 d (2H, CH_2, J_{PH} 19.3), 3.21 and 3.22 two s (6H, $N(CH_3)_2$), 3.29-3.39 m (2H, CH_2), 3.59 m (2H, CH_2), 3.82 dt (2H, CH_3CH_2C<u>H</u>$_2$O, J_{HH} 7.2, J_{PH} 3.7), 5.10 br s (1H, OH), 7.04 s (2H, CH_{Ar}, J_{PH} 2.5).

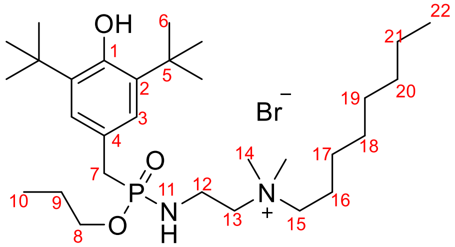

^{13}C NMR (126 MHz, $CDCl_3$, δ, ppm, *J*/Hz): 9.5 (C^{10}), 13.4 (C^{22}), 21.9 (C^9), 23.3 (C^{21}), 25.6 (C^{16}), 28.4 (C^{17}), 28.4 (C^{18-19}), 29.7 (C^6), 31.0 (C^{20}), 33.7 (C^5), 35.2 (C^7, J_{PC} 124.7), 36.1 (C^{12}), 43.2 (C^{14}), 50.9 (C^{13}), 65.3 (C^8), 122.0 (C^4), 126.1 (C^3), 135.7 (C^2), 152.1 (C^1). ^{31}P NMR (243 MHz, $CDCl_3$, δ, ppm, *J*/Hz): 31.6. Elemental analysis. Found, %: C, 59.94; H, 10.12; Br, 12.85; N, 4.63; P, 5.31. $C_{30}H_{58}N_2O_3PBr$. Calculated, %:C, 59.49; H, 9.65; Br, 13.19; N, 4.63; P, 5.11 MS (ESI-TOF), m/z (*rel. int.*, %): Found, 526.5 [M–Br]$^+$. Calculated for the bromide salt: 605.6 [M+H]$^+$.

***N*-(2-(((3,5-di-*tert*-butyl-4-hydroxybenzyl)(methoxy)phosphoryl)amino)ethyl)-*N*,*N*-dimethyldecan-1-aminium bromide 7a:** Yield 0.11 g (87%). ^{1}H NMR (400 MHz, $CDCl_3$,

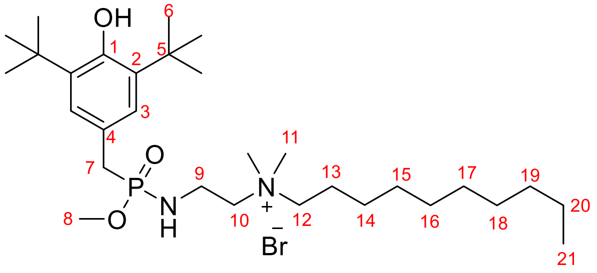

δ, ppm., *J*/Hz): 0.84 br t (3H, CH_3, J_{HH} 6.4), 1.21 m (14H, CH_2, 3H, CH_3), 1.39 s (18H, CH_3), 1.62 br m (2H, CH_2), 3.01 br d (2H, CH_2, J_{PH} 19.2), 3.10 and 3.15 two s (6H, $N(CH_3)_2$), 3.34 - 3.46 br m (2H, CH_2 and 4H, CH_2CH_2NH), 3.6 d (3H, OCH_3, J_{PH} 11.0), 5.0 br s (1H, OH), 7.09 s (2H, CH_{Ar}, J_{PH} 2.1). ^{13}C NMR (101 MHz, $CDCl_3$, δ, ppm, *J*/Hz): 14.5 (C^{21}), 23.1 (C^{20}), 23.2 (C^{13}), 26.7 (C^{14}), 29.6 (C^{15-18}), 30.9 (C^6), 32.2 (C^{19}), 34.7 (C^5), 34.9 (C^7), 35.4 (C^9), 52.0 (C^{11}), 52.2 (C^8), 65.2 (C^{12}), 66.1 (C^{10}), 122.8 (C^4), 127.2 (C^3), 136.8 (C^2), 153.2 (C^1). ^{31}P NMR(243 MHz, $CDCl_3$, δ, ppm, *J*/Hz): 32.2. Elemental analysis. Found, %: C, 59.21; H, 9.71; Br, 13.23; N, 4.54; P, 5.23. $C_{30}H_{58}N_2O_3PBr$. Calculated, %:C, 59.49; H, 9.65; Br, 13.19; N, 4.63; P, 5.11. MS (ESI-TOF), m/z (*rel. int.*, %): Found, 526.4 $[M–Br]^+$. Calculated for the bromide salt: 605.6 $[M+H]^+$.

***N*-(2-(((3,5-di-*tert*-butyl-4-hydroxybenzyl)(ethoxy)phosphoryl)amino)ethyl)-*N*,*N*-dimethyldecan-1-aminium 7b:** Yield 0.15 g (95%). IR (nujol), ν, cm $^{-1}$: 981 (P-N), 1044 (P–

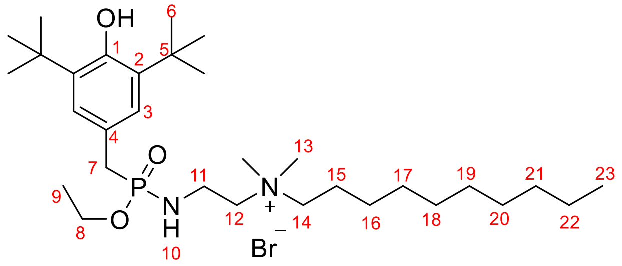

O–C_{alk}), 1323 (P=O), 1394-1412 (Calk), 1662 (C=C_{Ar}), 3632 (OH). ^{1}H NMR (400 MHz, $CDCl_3$, δ, ppm., *J*/Hz): 0.89 t (3H, CH_3, J_{HH} 6.9), 1.25 m (14H, CH_2, 3H, CH_3), 1.44 s (18H, CH_3), 1.60 br m (2H, CH_2), 2.89 br m (2H, CH_2), 2.93 m (2H, CH_2), 3.19 and 3.21 two s (6H, $N(CH_3)_2$,), 3.34 br m (2H, CH_2), 3.55 br m (2H, CH_2), 3.92-4.01 m (2H, OCH_2), 4.82 br s (1H, NH), 5.13 br s (1H, OH), 7.14 s (2H, CH_{Ar}, J_{PH} 2.2). ^{13}C NMR (101 MHz, $CDCl_3$, δ, ppm, *J*/Hz): 14.6 (C^{25}), 17.0 (C^9), 23.0 (C^{24}), 23.1 (C^{15}), 26.7 (C^{16}), 29.6 (C^{17-22}), 30.0 (C^6), 31.0 (C^{23}), 35.0 (C^5), 35.3 (C^7), 36.0 (C^{11}), 51.8 (C^{13}), 61.0 (C^8), 64.8 (C^{14}), 65.6 (C^{12}), 123.2 (C^4), 127.0 (C^3), 137.9 (C^2), 153.2 (C^1). ^{31}P NMR (243 MHz, $CDCl_3$, δ, ppm, *J*/Hz): 31.4. Elemental analysis. Found, %: C, 63.14; H, 9.02 Br, 12.61; N, 4.73; P, 4.17. $C_{31}H_{60}N_2O_3PBr$. Calculated, %:C, C, 63.58; H, 9.39; Br, 11.13; N, 3.90; P, 4.31. %:C, MS (ESI-TOF), m/z (*rel. int.*, %): Found, 540.4 $[M–Br]^+$. Calculated for the bromide salt: 620.1 $[M+H]^+$.

***N*-(2-(3,5-di-*tert*-butyl-4-hydroxybenzyl)(*iso*-propyl)phosphoryl(amino)ethyl)-*N*,*N*-dimethyldecane-1-aminium bromide 7c:** Yield 0.12 g (93%). IR, *ν*, cm $^{-1}$: 991 (P-N), 1317 (P–O–C_{alk}), 1372 (P=O), 1635 (C=C_{Ar}), 3644 (OH). ^{1}H NMR (400 MHz, $CDCl_3$, δ, ppm., *J*/Hz): 0.65 br s (3H, CH_3), 1.04 br s (14H, CH_2, 3H, CH_3), 1.20 br s (18H, CH_3), 1.67 br m (2H, CH_2), 2.55-3.30 br m (18H, CH_2), 4.35 br m (1H, CH), 4.59 br s (1H, NH) 7.13 br s (2H, CH_{Ar}). ^{13}C NMR (101 MHz, $CDCl_3$, δ, ppm, *J*/Hz): 14.6 (C^{23}), 23.1 (C^{22}), 23.4 (C^{16}), 26.7 (C^{9}), 27.0 (C^{16}), 29.6 (C^{15}), 29.9 (C^{17-20}), 30.9 (C^{6}), 32.4 (C^{21}), 34.8 (C^{5}), 35.6 (C^{7}, J_{PC} 119.8), 44.0 (C^{11}), 52.0 (C^{13}), 65.5 (C^{12}), 66.2 (C^{15}), 70.1 (C^{8}), 123.1 (C^{4}), 127.0 (C^{3}), 136.6 (C^{2}), 167.5 (C^{1}). ^{31}P NMR(243 MHz, $CDCl_3$, δ, ppm, *J*/Hz): 31.4. Elemental analysis. Found, %: C, 59.23; H, 10.24; Br, 12.95; N, 4.52; P, 4.34. $C_{32}H_{62}N_2O_3PBr$. Calculated, %:C, 60.65; H, 9.86; Br, 12.42; N, 4.42; P, 4.89. MS (ESI-TOF), m/z (*rel. int.*, %): Found, 553.4 $[M–Br]^+$. Calculated for the bromide salt: 634.2 $[M+H]^+$.

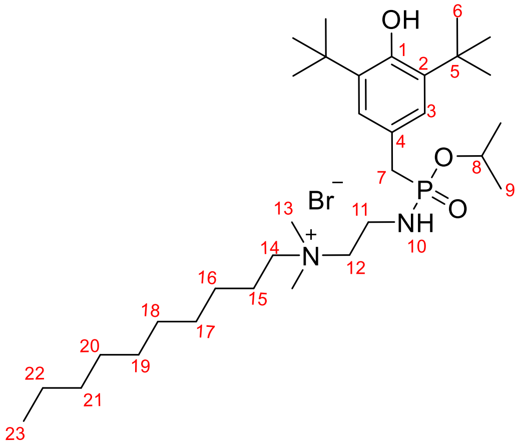

***N*-(2-(3,5-di-*tert*-butyl-4-hydroxybenzyl)(propoxyl)phosphoryl(amino)ethyl)-*N*,*N*-dimethyldecan-1-aminium bromide 7d:** Yield 0.10 g (85%). ^{1}H NMR (400 MHz, $CDCl_3$, δ, ppm., *J*/Hz): 0.80 br m (3H, CH_3, 3H, CH_3CH_2), br m (14H, CH_2), 1.43 s (18H, CH_3), 1.52 br m (2H, $CH_3CH_2CH_2O$), 1.61 (2H, CH_2), 2.82 m (2H, CH_2), 3.01 – 3.04 (2H, CH_2, 2H PCH_2), 3.09 br s and 3.13 br s (6H, $N(CH_3)_2$,), 3.31 br m (2H, CH_2), 3.48 m (2H, CH_2), 3.78 m (2H, $CH_3CH_2C\underline{H}_2O$), 4.79 br m (1H, NH or OH), 7.12 s (2H, CH_{Ar}). ^{13}C NMR (101 MHz, $CDCl_3$, δ, ppm, *J*/Hz): 9.6 (C^{10}), 13.5 (C^{24}), 22.0 (C^{9}), 23.2 (C^{23}), 25.7 (C^{16}), 28.6 (C^{17}), 28.4 (C^{18-21}), 29.7 (C^{6}), 31.0 (C^{22}), 33.7 (C^{5}), 34.6 (C^{7}), 35.7 (C^{12}), 42.9 (C^{14}), 50.9 (C^{13}), 65.8 (C^{8}), 122.0 (C^{4}), 126.0 (C^{3}), 135.5 (C^{2}), 152.0 (C^{1}). ^{31}P NMR (243 MHz, $CDCl_3$, δ, ppm, *J*/Hz): 31.9. Elemental analysis. Found, %: C, 60.59; H, 10.01; Br, 12.74; N, 4.12; P, 5.11. $C_{32}H_{62}N_2O_3PBr$. Calculated, %:C, 60.65; H, 9.86; Br, 12.61; N, 4.42; P, 4.89. MS (ESI-TOF), *m*/*z* (*rel. int.*, %): Found, 554.6 $[M–Br]^+$. Calculated for the bromide salt: 634.4 $[M+H]^+$.

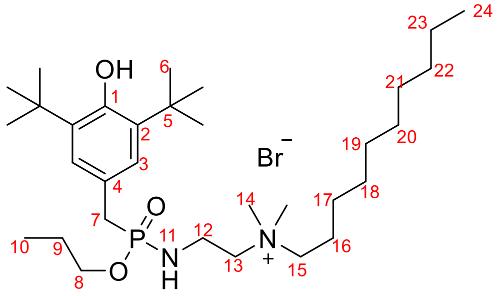

***N*-(2-(3,5-di-*tert*-butyl-4-hydroxybenzyl)(methoxy)phosphoryl(amino)propyl)-*N*,*N*-dimethyldodecan-1-aminium bromide 8a:** Yield 0.14 g (92%). ^{1}H NMR (400 MHz, CDCl$_3$, δ, ppm., *J*/Hz): 0.89 t (3H, CH$_3$, J_{HH} 8.0, 1.25 br m (16H, CH$_2$), 1.39 s (18H, CH$_3$), 1.62 br m (2H, CH$_2$), 1.79 br m (2H, CH$_2$), 2.63 m 2H, CH$_2$), 2.99 d (2H, CH$_2$, J_{PH} 19.9), 3.11, 3.16 two s (6H, N(CH$_3$)$_2$), 3.36 m (2H, CH$_2$), 3.42 br m (2H, CH$_2$), 3.65 d (6H, (OCH$_3$, J_{PH} 8.0), 5.01 br m (1H, OH), 7.15 s (2H, CH$_{Ar}$, J_{PH} 2.1). ^{13}C NMR (101 MHz, CDCl$_3$, δ, ppm, *J*/Hz): 14.6 (C^{23}), 23.1 (C^{22}), 23.5 (C^{13}), 26.8 (C^{14}), 29.9 (C$^{15-20}$), 30.9 (C^{6}), 32.4 (C^{21}), 33.3 (C^{5}), 34.5 (C^{7}), 35.4 (C^{9}), 52.0 (C^{11}), 52.3 (C^{8}), 65.4 (C^{12}), 66.3 (C^{10}), 122.8 (C^{4}), 127.2 (C^{3}), 136.7 (C^{2}), 153.2 (C^{1}). ^{31}P NMR(243 MHz, CDCl$_3$, δ, ppm, *J*/Hz): 32.7. Elemental analysis. Found, %: C, 59.99; H, 9.53; Br, 12.88; N, 4.61; P, 4.74. $C_{32}H_{62}N_2O_3PBr$. Calculated, %:C, 60.65; H, 9.86; Br, 12.61; N, 4.42; P, 4.89. MS (ESI-TOF), *m*/*z* (*rel. int.*, %): Found, 556.6 [M–Br]$^+$. Calculated for the bromide salt: 633.7 [M+H]$^+$.

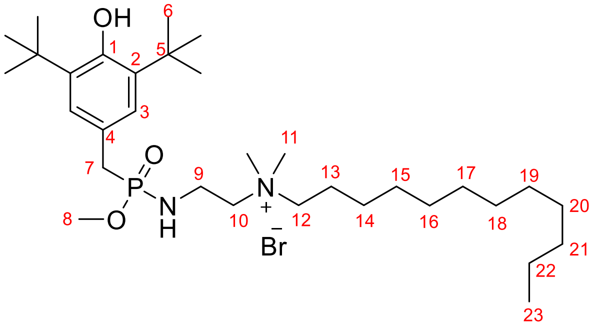

***N*-(2-(3,5-di-*tert*-butyl-4-hydroxybenzyl)(ethoxy)phosphoryl(amino)ethyl)-*N*,*N*-dimethyldodecan-1-aminium bromide 8b:** Yield 0.13 г (94%). IR, *ν*, cm^{-1}: 979 (P-N), 1071 (P–O–C$_{alk}$), 1323 (P=O), 1648 (C=C$_{Ar}$), 3645 (OH).). ^{1}H NMR (600 MHz, CDCl$_3$, δ, ppm., *J*/Hz): 0.79 t (3H, CH$_3$, J_{HH} 6.0), 1.18 m (16H, CH$_2$), 1.22 br m (3H, CH$_3$), 1.35 s (18H, CH$_3$), 1.60 br m (2H, CH$_2$), 1.77 br m (2H, CH$_2$), 2.68 br m (2H, CH$_2$), 2.97 m (2H, CH$_2$), 3.04, 3.11 two s (6H, N(CH$_3$)$_2$,), 3.27 – 3.53 br m (6H, CH$_2$), 3.93 m (2H, OCH$_2$), 4.84 br s (1H, NH or OH), 7.07 s (2H, CH$_{Ar}$). ^{13}C NMR (150 MHz, CDCl$_3$, δ, ppm, *J*/Hz): 14.5 (C^{23}), 17.0 (C^{9}), 23.0 (C^{22}), 23.3 (C^{15}), 25.7 (C^{16}), 29.6 (C$^{17-20}$), 30.9 (C^{6}), 32.1 (C^{21}), 34.9 (C^{5}), 36.2 (C^{7}), 38.0 (C^{11}), 51.6 (C^{14}), 61.3 (C^{8}), 63.0 (C^{14}), 65.1 (C^{12}), 123.1 (C^{4}), 127.0 (C^{3}), 136.6 (C^{2}), 153.1 (C^{1}). ^{31}P NMR(243 MHz, CDCl$_3$, δ, ppm, *J*/Hz): 31.3. Elemental analysis. Found, %: C, 58.98; H, 10.01; Br, 12.44; N, 4.81; P, 5.14. $C_{31}H_{60}N_2O_3PBr$. Calculated, %:C, 60.08; H, 9.76; Br, 12.89; N, 4.42; P, 5.00. MS (ESI-TOF), *m*/*z* (*rel. int.*, %): Found, 568.8 [M–Br]$^+$. Calculated for the bromide salt: 647.76 [M+H]$^+$.

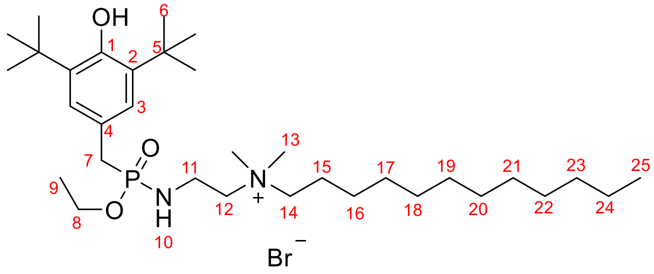

***N*-(2-(3,5-di-*tert*-butyl-4-hydroxybenzyl)(*iso*-propyl)phosphoryl(amino)ethyl)-*N*,*N*-dimethyldodecan-1-aminium bromide 8c:** Yield 0.15 g (94%). IR, ν, cm^{-1}: 979 (P-N), 1311 (P–O–C_{alk}), 1358 (P=O), 1612 (C=C_{Ar}), 3656 (OH). ^{1}H NMR (400 MHz, $CDCl_3$, δ, ppm., *J*/Hz): 0.88 t (3H, CH_3, J_{HH} 6.2), 1.21 d (3H, CH_3, J_{HH} 8.0), 1.25 m (18H, CH_2), 1.34 m (3H, CH_3), 1.43 s (18H, $(CH_3)_3$), 1.64 br m (2H, CH_2), 2.72 br m (2H, CH_2), 3.01 br m (2H, CH_2), 3.12, 3.18 two s (6H, $N(CH_3)_2$), 3.35 - 3.47 m (4H, CH_2), 4.55 br m (1H, CH), 4.71 br s (1H, NH or OH), 7.13 s (2H, CH_{Ar}). ^{13}C NMR (101 MHz, $CDCl_3$, δ, ppm, *J*/Hz): 14.4 (C^{25}), 22.9 (C^{24}), 24.4 (C^{16}), 24.8 (C^9), 28.4 (C^{17}), 29.0 (C^{18-22}), 30.8 (C^6), 32.1 (C^{23}), 34.7 (C^5), 35.5 (C^7, J_{PC} 130), 44.2 (C^{11}), 52.0 (C^{13}), 65.5 (C^{12}), 66.4 (C^{14}), 70.1 (C^8), 123.1 (C^4), 127.2 (C^3), 136.9 (C^2), 152.9 (C^1). ^{31}P NMR(243 MHz, $CDCl_3$, δ, ppm, *J*/Hz): 31.4. Elemental analysis. Found, %: C, 61.36; H, 10.14; Br, 12.23; N, 4.34; P, 5.12. $C_{34}H_{66}N_2O_3PBr$. Calculated, %:C, 61.71; H, 10.05; Br, 12.07; N, 4.23; P, 4.68. MS (ESI-TOF), *m*/*z* (*rel. int.*, %): Found, 582.4 $[M–Br]^+$. Calculated for the bromide salt: 662.5 $[M+H]^+$.

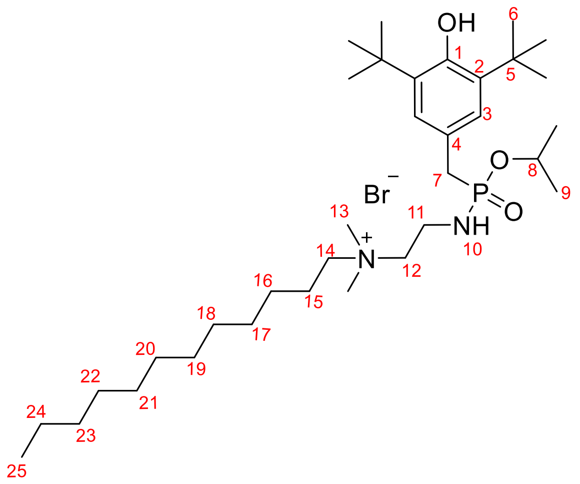

***N*-(2-(3,5-di-*tert*-butyl-4-hydroxybenzyl)(propoxyl)phosphoryl(amino)ethyl)-*N*,*N*-dimethyldodecan-1-aminium bromide 8d:** Yield 0.12 g (91%). ^{1}H NMR (400 MHz, $CDCl_3$, δ, ppm., *J*/Hz): 0.88 br t (3H, CH_3, 3H, C<u>H</u>$_3CH_2CH_2O$), 1.18 s (18H, CH_2), 1.34 s (18H, $(CH_3)_3$), 1.51 br s (2H, CH_3C<u>H</u>$_2CH_2O$), 1.68 m (2H, CH_2), 2.67 s (2H, CH_2), 3.00 br d (2H, CH_2, J_{PH} 19.0), 3.09, 3.11 two s (6H, $N(CH_3)_2$,), 3.29 br m (2H, CH_2), 3.46 br m (2H, (CH_2), 3.77 m (2H, CH_3CH_2C<u>H</u>$_2$O), 4.76 br s (1H, NH), 5.11 br s (1H, OH), 7.03 s (2H, CH_{Ar}). ^{13}C NMR (101 MHz, $CDCl_3$, δ, ppm, *J*/Hz): 9.6 (C^{10}), 13.5 (C^{26}), 23.2 (C^9), 23.3 (C^{25}), 25.8 (C^{16}), 27.6 (C^{17}), 29.05 (C^{18-23}), 29.9 (C^6), 31.4 (C^{24}), 33.7 (C^5), 34.4 (C^7, J_{PH} 125.2), 35.7 (C^{12}), 42.88 (C^{14}), 51.0 (C^{13}), 59.0 (C^8), 122.1 (C^4), 126.0 (C^3), 135.6 (C^2), 152.1 (C^1). ^{31}P NMR (243 MHz, $CDCl_3$, δ, ppm, *J*/Hz): 31.7. Elemental analysis. Found, %: C, 61.2; H, 10.04; Br, 12.05; N, 4.25; P, 4.72. $C_{34}H_{66}N_2O_3PBr$. Calculated, %:C, 61.71; H, 10.05; Br, 12.07; N, 4.23; P, 4.68. MS (ESI-TOF), *m*/*z* (*rel. int.*, %): Found, 582.4 $[M–Br]^+$. Calculated for the bromide salt: 662.2 $[M+H]^+$.

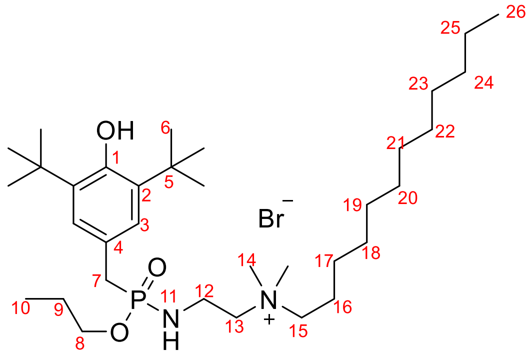

N-(2-((((3,5-di-*tret*-butyl-4-hydroxybenzyl)(methoxy)phosphoryl)amino)ethyl)-*N*,*N*-dimethyltetradecan-1-aminium bromide 9a: Yield 0.1 g (90%). ^{1}H NMR (500 MHz,

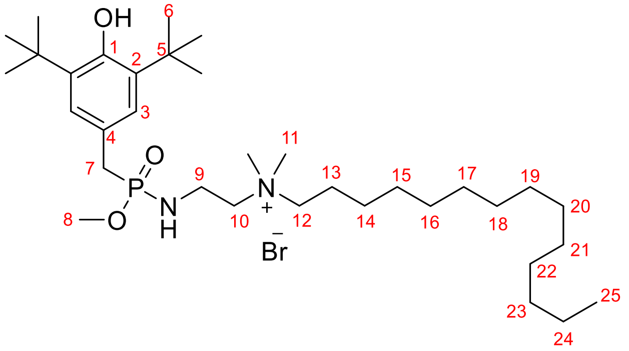

$CDCl_3$, δ, ppm., *J*/Hz): 0.75 s (3H, CH_3), 1.13 s (22H, CH_2), 1.30 s (18H, $(CH_3)_3$), 1.55 – 172 m (2H, CH_2), 2.56 br m (2H, CH_2), 2.93 m (2H, CH_2P), 3.01, 3.05 two s (6H, $N(CH_3)_2$), 3.38 – 3.48 br m (4H, CH_2), 3.50 d (6H, $(OCH_3)_2$, J_{PH} 10.0), 4.92 br s (1H, OH), 7.00 s (2H, CH_{Ar}). ^{13}C NMR (126 MHz, $CDCl_3$, δ, ppm, *J*/Hz): 13.9 (C^{25}), 22.5 (C^{24}), 22.6 (C^{13}), 26.1 (C^{14}), 29.5 (C^{15-22}), 30.3 (C^{6}), 31.7 (C^{23}), 32.7 (C^{5}), 33.7 (C^{7}, J_{PC} 126.0), 34.7 (C^{9}), 51.4 (C^{11}), 51.6 (C^{8}), 64.4 (C^{12}), 65.2 (C^{10}), 122.3 (C^{4}), 126.5 (C^{3}), 136.4 (C^{2}), 152.8 (C^{1}). ^{31}P NMR(243 MHz, $CDCl_3$, δ, ppm, *J*/Hz): 33.6. Elemental analysis. Found, %: C, 61.35; H, 10.19; Br, 11.90; N, 4.34; P, 4.51. $C_{34}H_{66}N_2O_3PBr$. Calculated, %:C, 61.71; H, 10.05; Br, 12.07; N, 4.23; P, 4.68. MS (ESI-TOF), *m/z* (*rel. int.*, %): Found, 582.3 $[M–Br]^+$. Calculated for the bromide salt: 661.8 $[M+H]^+$.

N-(2-(((3,5-di-*tert*-butyl-4-hydroxybenzyl)(ethoxy)phosphoryl)amino)ethyl)-*N*,*N*-dimethyltetradecan-1-aminium bromide 9b: Yield 0.09 g (88%). IR, *ν*, cm $^{-1}$: 984 (P-N),

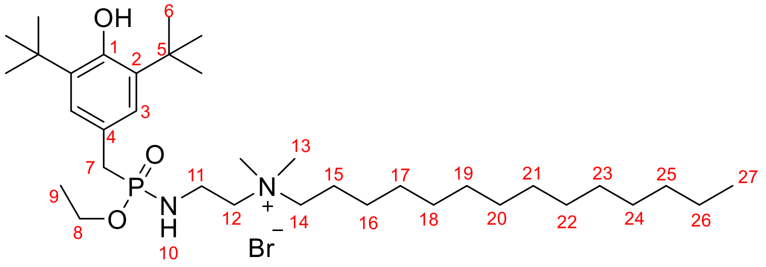

1093 (P–O–C_{alk}), 1298 (P=O), 1615 (C=C_{Ar}), 3628 (OH). ^{1}H NMR(400 MHz, $CDCl_3$, δ, ppm., *J*/Hz): 0.77 s (3H, CH_3), 1.15 - 1.22 m (20H, CH_2, 3H, CH_3), 1.33 s (18H, $(CH_3)_3$), 1.58 m (2H, CH_2), 2.56 br s (2H, CH_2), 2.94 d (2H, CH_2, J_{PH} 19.0), 3.05, 3.10 two s (6H, $N(CH_3)_2$,), 3.28 – 3.48 br m (4H, CH_2), 3.88 br m (2H, CH_2), 4.77 s (1H, NH), 5.09 s (1H, OH), 7.02 s (2H, CH_{Ar}). ^{13}C NMR (101 MHz, $CDCl_3$, δ, ppm, *J*/Hz): 14.0 (C^{27}), 16.4 (C^{9}), 22.5 (C^{26}), 22.6 (C^{15}), 26.2 (C^{16}), 29.6 (C^{17-24}), 30.3 (C^{6}), 31.8 (C^{25}), 33.9 (C^{7}), 34.9 (C^{5}), 35.7 (C^{11}), 51.5 (C^{13}), 60.7 (C^{8}), 64.86(C^{14}), 65.6 (C^{12}), 122.4 (C^{4}), 126.6 (C^{3}), 136.2 (C^{2}), 152.6 (C^{1}). ^{31}P NMR (243 MHz, $CDCl_3$, δ, ppm, *J*/Hz): 31.6. Elemental analysis. Found, %: C, 61.74; H, 10.04; Br, 11.74; N, 4.42; P, 4.61. $C_{35}H_{68}N_2O_3PBr$. Calculated, %:C, 62.20; H, 10.14; Br, 11.82; N, 4.15; P, 4.58. MS (ESI-TOF), *m/z* (*rel. int.*, %): Found, 596.6 $[M–Br]^+$. Calculated for the bromide salt: 675.8 $[M+H]^+$.

N-(2-(((3,5-di-*tert*-butyl-4-hydroxyphenyl)(*iso*-propoxy)phosphoryl)amino)ethyl)-N,N-dimethyltetradecan-1-aminium bromide 9c: Yield 0.13 g (91%). IR, ν, cm $^{-1}$: 995 (P-N), 1045 (P–O–C_{alk}), 1317 (P=O), 1634 (C=C_{Ar}), 3645 (OH). ^{1}H NMR (400 MHz, $CDCl_3$, δ, ppm., J/Hz): 0.88 t (3H, CH_3, J_{HH} 8.0), 1.21 d (3H, CH_3, J_{PH} 8.1), 1.25 m (18H, CH_2), 1.31 d (3H, CH_3, J_{PH} 8.0), 1.43 s (18H, $(CH_3)_3$), 1.67 br m (2H, CH_2), 2.67, 2.95 two m (2H, CH_2), 3.02 d (2H, CH_2, J_{PH} 20.0), 3.13, 3.19 two s (6H, $N(CH_3)_2$), 3.33 – 3.50 br m (2H, CH_2), 4.55 m (1H, CH), 7.16 s (2H, CH_{Ar}). ^{13}C NMR (101 MHz, $CDCl_3$, δ, ppm, J/Hz): 14.5 (C^{27}), 23.1 (C^{26}), 24.6 (C^{16}), 26.7 (C^{9}), 29.6 (C^{15}), 29.8 (C^{18-24}), 30.9 (C^{6}), 32.4 (C^{25}), 34.8 (C^{5}), 35.6 (C^{7}), 44.2 (C^{11}), 52.0 (C^{13}), 62.9 (C^{12}), 65.0 (C^{14}), 69.8 (C^{8}), 123.4 (C^{4}), 127.0 (C^{3}), 136.6 (C^{2}), 153.5 (C^{1}). ^{31}P NMR(243 MHz, $CDCl_3$, δ, ppm, J/Hz): 31.2. Elemental analysis. Found, %: C, 63.2; H, 10.44; Br, 11.32; N, 4.21; P, 4.23. $C_{36}H_{70}N_2O_3PBr$. Calculated, %:C, 62.28; H, 10.23; Br, 11.58; N, 4.06; P, 4.49. MS (ESI-TOF), m/z (*rel. int.*, %): Found, 610.5 $[M–Br]^+$. Calculated for the bromide salt: 690 $[M+H]^+$.

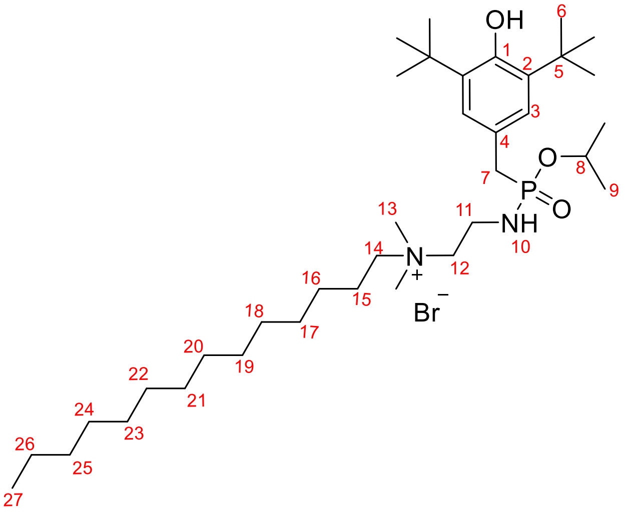

N-(2-(3,5-di-*tert*-butyl-4-hydroxybenzyl)(propoxyl)phosphoryl(amino)ethyl)-*N,N*-dimethyldecan-1-aminium bromide 9d: Yield 0.13 g (86%). ^{1}H NMR (400 MHz, $CDCl_3$, δ, ppm., J/Hz): 0.87 t (3H, CH_3, J_{HH} 7.0), 0.89 s (3H, CH_3, 3H, $CH_3(CH_2)_2O$), 1.26 s (18H, CH_2), 1.42 s (18H, CH_3), 1.50 m (2H, $CH_3C\underline{H}_2CH_2O$), 1.71 m (2H, CH_2), 2.68 m (2H, CH_2), 3.07 br d (2H, CH_2, J_{PH} 20.0), 3.00 m (2H, CH_2), 3.29 m (6H, $N(CH_3)_2$,), 3.45 br m (2H, CH_2), 3.78 m (2H, CH_2O), 4.74 br s (1H, NH), 7.03 s (2H, CH_{Ar}). ^{13}C NMR (101 MHz, $CDCl_3$, δ, ppm, J/Hz): 9.5 (C^{10}), 13.5 (C^{28}), 22.1 (C^{9}), 23.3 (C^{27}), 27.6 (C^{16}), 28.2 (C^{17}), 29.08 (C^{18-25}), 29.8 (C^{6}), 31.3 (C^{26}), 33.8 (C^{5}), 34.7 (C^{7}), 35.6 (C^{12}), 42.8 (C^{14}), 50.9 (C^{13}), 65.4 (C^{8}), 122.1 (C^{4}), 126.0 (C^{3}), 135.5 (C^{2}), 152.1 (C^{1}). ^{31}P NMR (243 MHz, $CDCl_3$, δ, ppm, J/Hz): 31.2. Elemental analysis. Found, %: C, 62.54; H, 10.12; Br, 11.62; N, 4.08; P, 4.89. $C_{36}H_{70}N_2O_3PBr$. Calculated, %:C, 62.68; H, 10.23; Br, 11.58; N,

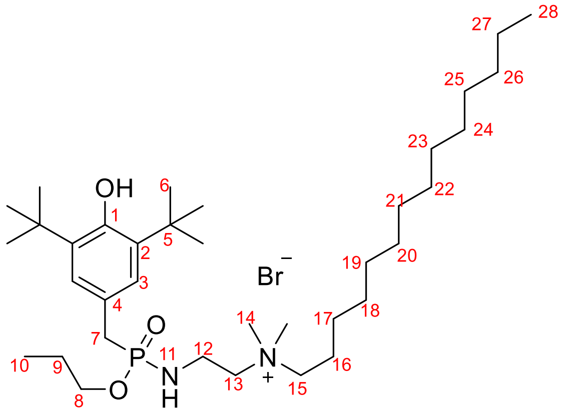

4.06; P, 4.49. MS (ESI-TOF), m/z (*rel. int.*, %): Found, 610.2 [M–Br]+. Calculated for the bromide salt: 689.84 [M+H]+.

***N*-(3-(((3,5-di-*tert*-butyl-4-hydroxybenzyl)(methoxy)phosphoryl)amino)ethyl)-*N*,*N*-dimethyhexadecan-1-aminium bromide 10a:** Yield 0.07 g (79%). ^{1}H NMR (400 MHz, CDCl$_3$, δ, ppm., *J*/Hz): 0.85 t (3H, CH$_3$, J_{HH} 6.6), 1.23 s (26H, CH$_2$), 1.40 s (18H, CH$_3$), 1.82 quint (2H, CH$_2$, J_{HH} 6.9), 2.54 m (2H, CH$_2$), 3.03 d (2H, CH$_2$, J_{PH} 18.5), 3.15, 3,18 two s (6H, N(CH$_3$)$_2$), 3.37 t (4H, CH$_2$, J_{HH} 6.9), 3.58 d (3H, OCH$_3$, J_{PH} 11.1), 5.02 br s (1H, NH), 5.16 br s (1H, OH), 7.11 s (2H, CH$_{Ar}$). ^{13}C NMR (101 MHz, CDCl$_3$, δ, ppm, *J*/Hz):14.0 (C^{27}), 22.6 (C^{26}), 23.1 (C^{13}), 26.2 (C^{14}), 29.4 (C$^{15-24}$), 30.4 (C^6), 31.9 (C^{21}), 32.7 (C^5), 36.0 (C^7), 43.2 (C^9), 51.4 (C^{11}), 51.6 (C^8), 64.9 (C^{12}), 65.7 (C^{10}), 122.3 (C^4), 126.5 (C^3), 136.2 (C^2), 152.6 (C^1). ^{31}P NMR (243 MHz, CDCl$_3$, δ, ppm, *J*/Hz): 32.7. Elemental analysis. Found, %: C, 62.44; H, 10.05; Br, 11.95; N, 4.12; P, 4.54. $C_{34}H_{66}N_2O_3PBr$. Calculated, %:C, 62.68; H, 10.32; Br, 11.58; N, 4.06; P, 4.49. MS (ESI-TOF), *m*/*z* (*rel. int.*, %): Found, 610.8 [M–Br]+. Calculated for the bromide salt: 689.9 [M+H]+.

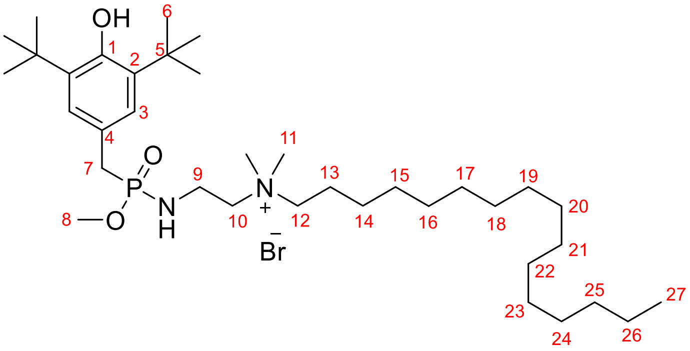

***N*-(2-(((3,5-di-*tert*-butyl-4-hydroxybenzyl)(ethoxy)phosphoryl)amino)ethyl)-*N*,*N*-dimethylhexadecan-1-aminium bromide 10b:** Yield 0.12 g (93%). IR, *ν*, cm^{-1}: 972 (P-N), 1089 (P–O–C$_{alk}$), 1341 (P=O), 1612 (C=C$_{Ar}$), 3674 (OH). ^{1}H NMR (400 MHz, CDCl$_3$, δ, ppm., *J*/Hz): 0.86 t (3H, CH$_3$, J_{HH} 8.0), 1.24 -1.25 m (26H, CH$_2$, 3H, CH$_3$), 1.42 s (18H, CH$_3$), 1.66 br m (2H, CH$_2$), 2.75 br m (2H, CH$_2$), 3.03 br m (2H, PCH$_2$), 3.10, 3.14 two s (6H, N(<u>CH$_3$</u>)$_2$), 3.38 br m (2H, CH$_2$), 3.50 br m (2H, CH$_2$), 3.97 m (2H, CH$_2$O), 4.88 br s (1H, NH or OH), 7.12 s (2H, CH$_{Ar}$). ^{13}C NMR (101 MHz, CDCl$_3$, δ, ppm, *J*/Hz): 14.0 (C^{29}), 16.4 (C^9), 22.6 (C^{28}), 22.9 (C^{15}), 26.2 (C^{16}), 29.3 (C$^{17-26}$), 30.4 (C^6), 31.8 (C^{25}), 34.2 (C^5), 35.0 (C^7), 35.7 (C^{11}), 51.4 (C^{13}), 60.8 (C^8), 64.8 (C^{14}), 65.7 (C^{12}), 122.5 (C^4), 126.7 (C^3), 136.1 (C^2), 152.6 (C^1). ^{31}P NMR(243 MHz, CDCl$_3$, δ, ppm, *J*/Hz): 31.7. Elemental analysis. Found, %: C, 63.45; H, 10.18; Br, 11.77; N, 4.23; P, 4.45. $C_{35}H_{68}N_2O_3PBr$. Calculated, %:C, 63.14; H, 10.31; Br, 11.35;

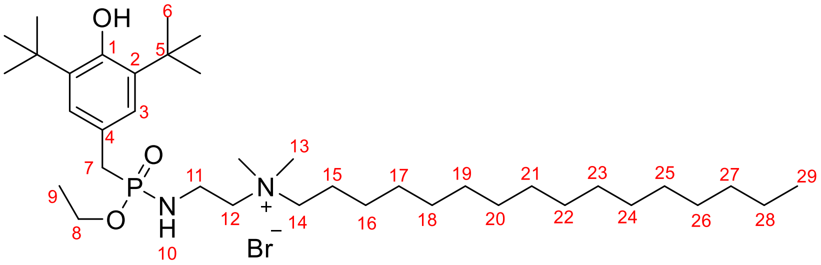

N, 3.98; P, 4.40. MS (ESI-TOF), m/z (*rel. int.*, %): Found, 624.1 $[M–Br]^+$. Calculated for the bromide salt: 703.8 $[M+H]^+$.

***N*-(2-(((3,5-di-*tert*-butyl-4-hydroxybenzyl)(*iso*-propoxy)phosphoryl)amino)ethyl)-*N*,*N*-dimethylhexadecan-1-aminium bromide 10c:** Yield 0.14 g (90%). IR, *ν*, cm^{-1}: 953 (P-N), 1031 (P–O–C_{alk}), 1288 (P=O), 1600 (C=C_{Ar}), 3590 (OH). ^1H NMR (400 MHz, $CDCl_3$, δ, ppm., *J*/Hz): 0.79 t (3H, CH_3, J_{HH} 8.0), 1.10 d (3H, CH_3, J_{HH} 6.3), 1.17 s (26H, CH_2), 1.22 d (3H, CH_3, J_{HH} 6.0), 1.35 s (18H, $(CH_3)_3$), 1.76 quint (2H, CH_2), 2.69 s (2H, CH_2), 2.99 d (2H, PCH_2, J_{PH} 19.6), 3.07, 3.12 two s (6H, $N(CH_3)_2$), 3.33 - 3.46 m (4H, CH_2), 4.46 br m (1H, CH), 4.65 br s (1H, NH), 5.06 br s (1H, OH), 7.05 s (2H, CH_{Ar}). ^{13}C NMR (101 MHz, $CDCl_3$, δ, ppm, *J*/Hz): 14.6 (C^{29}), 24.9 (C^{28}), 25.2 (C^{16}),24.6 (C^9), 26.7 (C^{15}), 29.8 (C^{18-26}), 30.9 (C^6), 32.3 (C^{27}), 34.8 (C^5), 36.9 (C^7, J_{PC} 124.8), 44.0 (C^{11}), 51.9 (C^{13}), 65.4 (C^{12}), 66.2 (C^{14}), 70.1 (C^8), 123.3 (C^4), 127.0 (C^3), 136.6 (C^2), 153.1 (C^1). ^{31}P NMR (243 MHz, $CDCl_3$, δ, ppm, *J*/Hz): 30.1. Elemental analysis. Found, %: C, 63.21; H, 9.44; Br, 11.23; N, 3.74; P, 5.32. $C_{38}H_{74}N_2O_3PBr$. Calculated, %:C, 63.58; H, 9.39; Br, 11.13; N, 3.90; P, 5.11. MS (ESI-TOF), *m/z* (*rel. int.*, %): Found, 638.1 $[M–Br]^+$. Calculated for the bromide salt: 718.1 $[M+H]^+$.

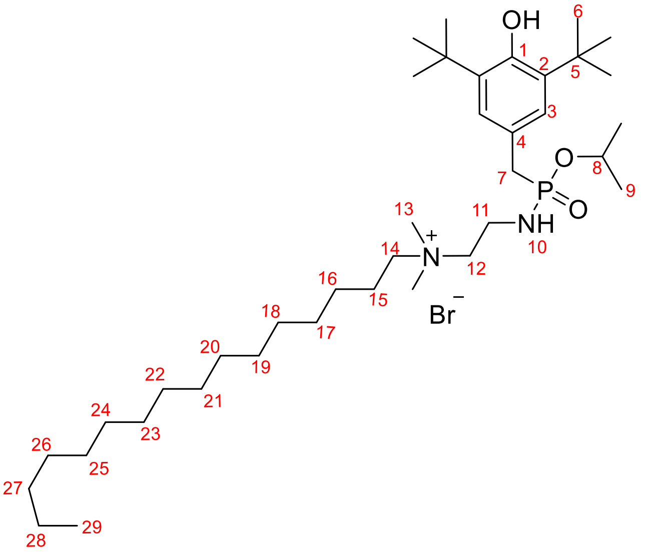

***N*-(3-(3,5-di-*tert*-butyl-4-hydroxybenzyl)(propoxy)phosphoryl(amino)ethyl)-*N*,*N*-dimethylhexadecan-1-aminium bromide 10d:** Yield 0.1 g (89%). ^1H NMR (400 MHz, acetone-d_6, δ, ppm., *J*/Hz): 0.82 br s (3H, CH_3, 3H, <u>C</u>$H_3(CH_2)_2O$), 1.20 br s (20H, CH_2), 1.37 s (18H, CH_3), 1.55 br s (2H, CH_2), 1.67 br s (2H, CH_2), 1.92 m (2H, CH_2), 2.22 br s (2H, CH_2), 2.40 br s (2H, CH_2), 2.86 br s (2H, CH_2), 3.00 br m (2H, PCH_2), 3.05 br s (6H, $N(CH_3)_2$), 3.36 br s (2H, CH_2), 3.55 m (2H, CH_2), 3.78 m (2H, OCH_2), 4.37 br s (1H, NH), 5.27 br s (1H, OH), 7.03 s (2H, CH_{Ar}). ^{13}C NMR (101 MHz, $CDCl_3$, δ, ppm, *J*/Hz): 9.6 (C^{10}), 13.6 (C^{30}), 22.1 (C^9), 23.3 (C^{29}), 25.6 (C^{16}), 27.6 (C^{17}), 28.8 (C^{18-27}), 29.9 (C^6),

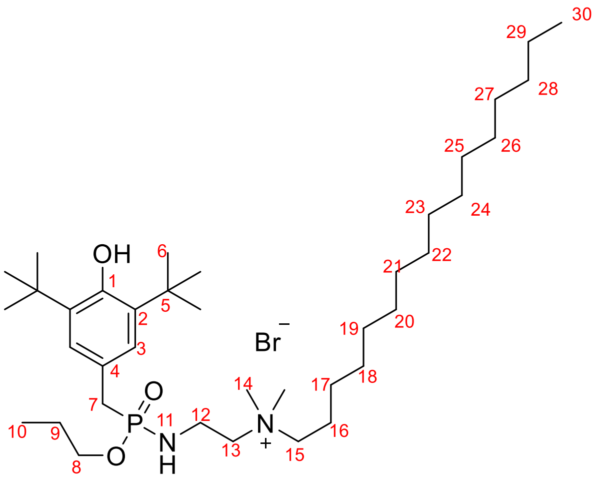

31.3 (C^{28}), 33.7 (C^{5}), 34.3 (C^{7}), 35.5 (C^{12}), 42.9 (C^{14}), 51.0 (C^{13}), 65.4 (C^{8}), 122.1 (C^{4}), 126.1 (C^{3}), 135.5 (C^{2}), 152.1 (C^{1}). ^{31}P NMR (243 MHz, $CDCl_3$, δ, ppm, *J*/Hz): 30.6. Elemental analysis. Found, %: C, 63.62; H, 10.22; Br, 11.31; N, 4.05; P, 4.52. $C_{38}H_{74}N_2O_3PBr$. Calculated, %:C, 63.58; H, 10.39; Br, 11.13; N, 3.90; P, 4.31. MS (ESI-TOF), *m*/*z* (*rel. int.*, %): Found, 638.3 [M–Br]$^+$. Calculated for the bromide salt: 717.6 [M+H]$^+$.

***N*-(3-(3,5-di-*tert*-butyl-4-hydroxybenzyl)(methoxy)phosphoryl(amino)propyl)-*N*,*N*-dimethyloctan-1-aminium bromide 11a:** Yield 0.13 g (92%). IR, ν, cm $^{-1}$: 982 (P–O–C_{alk}), 1288 (P=O), 1641 (C=C_{Ar}), 3656 (OH). ^{1}H NMR(500 MHz, $CDCl_3$, δ, ppm., *J*/Hz): 0.84 t (3H, CH_3, J_{HH} 6.0), 1.24 br m (10H, CH_2), 1.39 s (18H, CH_3), 1.69 br s (2H, CH_2), 1.95 br m (2H, CH_2), 2.65 br m (2H, CH_2), 2.96 br m (2H, CH_2), 3.06 d (2H, PCH_2, J_{PH} 19.0), 3.19, 3.20 two s (6H, $N(CH_3)_2$), 3.34 br m (2H, CH_2), 3.57 d (6H, OCH_3, J_{PH} 6.0), 4.35 br s (1H, NH), 5.14 br s (1H, OH),7.05 s (2H, CH_{Ar}). ^{13}C NMR(126 MHz, $CDCl_3$, δ, ppm, *J*/Hz): 14.0 (C^{20}), 22.5 (C^{19}), 22.7 (C^{14}), 25.2 (C^{10}), 26.2 (C^{15}), 29.1 ($C^{16,17}$), 30.4 (C^{6}), 31.5 (C^{18}), 34.1 (C^{5}), 35.1 (C^{7}, J_{PC} 126.0), 37.4 (C^{9}), 51.1 (C^{12}), 51.6 (C^{18}), 62.4 (C^{13}), 64.5 (C^{11}), 122.3 (C^{4}), 126.1 (C^{3}), 138.0 (C^{2}), 152.8 (C^{1}). ^{31}P NMR(243 MHz, $CDCl_3$, δ, ppm, *J*/Hz): 33.2. Elemental analysis. Found, %: C, 58.91; H, 9.66; Br, 13.11; N, 4.66; P, 5.31. $C_{29}H_{56}N_2O_3PBr$. Calculated, %:C, 58.87; H, 9.54; Br, 13.51; N, 4.73; P, 5.24. MS (ESI-TOF), *m*/*z* (*rel. int.*, %): Found, 512.3 [M–Br]$^+$. Calculated for the bromide salt: 591.8 [M+H]$^+$.

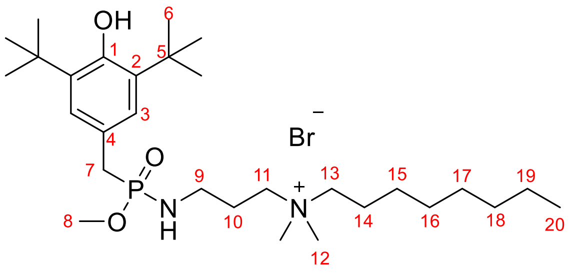

***N*-(3-(((3,5-di-*tert*-butyl-4-hydroxyphenyl)(ethoxy)phosphoryl)amino)propyl)-*N*,*N*-dimethyloctan-1-aminium bromide 11b:** Yield 0.1 g (92%). IR (nujol), *ν*, см$^{-1}$: 1160 (P–O–C_{alk}), 1316 (P=O), 1377-1459 (C_8H_{17}), 1658 (C=C_{Ar}), 3648 (OH). ^{1}H NMR (400 MHz, $CDCl_3$, δ, ppm., *J*/Hz): 0.85 m (3H, CH_3), 1.19 – 1.23 m (10H, CH_2; 3H, CH_3), 1.40 s (18H, CH_3), 1.69 br s (2H, CH_2), 1.97 br s (2H, CH_2), 2.98 br m (2H, CH_2), 3.04 d (2H, PCH_2, J_{PH} 19.5), 3.23 s (6H, $N(CH_3)_2$), 3.41 br m (2H, CH_2), 3.68 br s (2H, CH_2), 3.97 m (2H, OCH_2), 5.14 br s (1H, OH), 7.06 s (2H, CH_{Ar}). ^{13}C NMR (101 MHz, $CDCl_3$, δ, ppm, *J*/Hz): 14.5 (C^{22}), 16.9 (C^{9}), 23.0 (C^{23}), 23.3 (C^{16}), 25.7 (C^{12}), 26.8 (C^{17}), 29.5 (C^{18-19}, J_{PC} 12.1 Гц), 30.9 (C^{6}), 34.8 (C^{20}), 34.9 (C^{5}), 35.5 (C^{7}, J_{PC} 124.1), 38.0 (C^{11}), 51.6 (C^{14}), 61.3 (C^{8}), 63.0 (C^{13}), 65.2 (C^{13}), 123.1 (C^{4}, J_{PC} 8.6), 127.0 (C^{3}), 136.6 (C^{2}), 153.1 (C^{1}). ^{31}P NMR (243 MHz, $CDCl_3$, δ, ppm, *J*/Hz): 31.7. Elemental

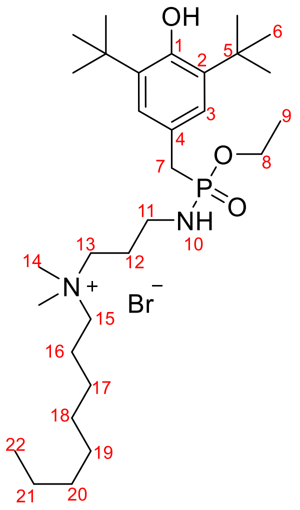

analysis. Found, %: C, 59.77; H, 10.01; Br, 12.81; N, 4.72; P, 5.54. $C_{30}H_{58}N_2O_3PBr$. Calculated, %:C, 59.49; H, 9.65; Br, 13.19; N, 4.63; P, 5.11. MS (ESI-TOF), *m/z* (*rel. int.*, %): Found, 525.8 $[M–Br]^+$. Calculated for the bromide salt: 605.69 $[M+H]^+$.

***N*-(3-(((3,5-di-*tert*-butyl-4-hydroxybenzyl)(*iso*-propoxy)phosphoryl)amino)propyl)-*N*,*N*-dimethyloctan-1-aminium bromide 11c:** Yield 0.14 g (95%). IR, ν, cm^{-1}: 993 (P-N), 1112 (P–O–C_{alk}), 1319 (P=O), 1632 (C=C_{Ar}), 3644 (OH). ^{1}H NMR (500 MHz, $CDCl_3$, δ, ppm., *J*/Hz): 0.87 t (3H, CH_3, J_{HH} 6.2), 1.16 d (3H, CH_3, J_{HH} 6.0), 1.25 m (10H, CH_2), 1.27 br s (3H, CH_3), 1.41 s (18H, $(CH_3)_3$), 1.70 br m (2H, CH_2), 1.96 br m (2H, CH_2), 2.97 br m (2H, CH_2), 3.04 d (2H, PCH_2, J_{PH} 19.4), 3.23 s (6H, N($\underline{CH_3}$)$_2$), 3.41 br m (4H, $(CH_2)_2$), 3.61 br m (2H, CH_2), 4.38 m (1H, OCH), 4.55 br s (1H, NH), 7.07 s (2H, CH_{Ar}). ^{13}C NMR (101 MHz, $CDCl_3$, δ, ppm, *J*/Hz): 14.0 (C^{22}), 22.5 (C^{21}), 22.7 (C^{16}), 24.4 (C^9, J_{PC} 4.1), 26.2 (C^{17}), 29.1 (C^{18-19}), 30.4 (C^6), 31.6 (C^{20}), 34.3 (C^5), 35.6 (C^7, J_{PC} 124.5), 37.5 (C^{11}), 51.2 (C^{14}), 62.3 (C^{13}), 64.5 (C^{15}), 69.3 (C^8), 122.9 (C^4), 126.5 (C^3), 136.0 (C^2), 152.5 (C^1). ^{31}P NMR (243 MHz, $CDCl_3$, δ, ppm, *J*/Hz): 30.6. Elemental analysis. Found, %: C, 60.1; H, 9.75; Br, 12.83; N, 4.66; P, 5.34. $C_{31}H_{60}N_2O_3PBr$. Calculated, %:C, 60.08; H, 9.76; Br, 12.89; N, 4.52; P, 5.00. MS (ESI-TOF), *m/z* (*rel. int.*, %): Found, 540.8 $[M–Br]^+$. Calculated for the bromide salt: 619.6 $[M+H]^+$.

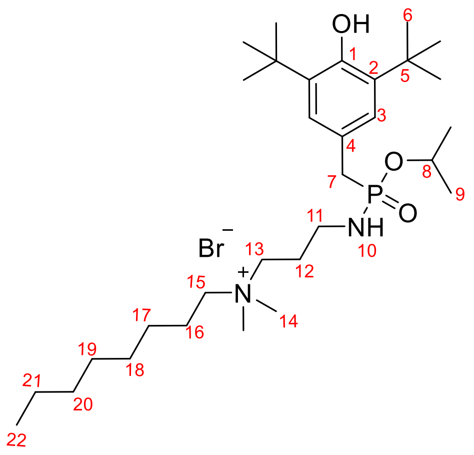

***N*-(3-(((3,5-di-*tert*-butyl-4-hydroxybenzyl)(propoxy)phosphoryl)amino)propyl)-*N*,*N*-dimethyloctan-1-aminium bromide 11d:** Yield 0.11 g (88%). ^{1}H NMR (400 MHz, $CDCl_3$, δ, ppm., *J*/Hz): 0.84 t (3H, CH_3; 3H, CH_3 J_{HH} 8.0), 1.23 m (10H, CH_2), 1.39 s (18H, $(CH_3)_3$), 1.56 m (2H, CH_2), 1.68 m (2H, CH_2), 1.93 m (2H, CH_2), 2.98 m (2H, CH_2), 3.03 d (2H, PCH_2, J_{PH} 20.2), 3.20, 3.21 two s (6H, $N(CH_3)_2$), 3.36 br m (2H, CH_2), 3.60 m (2H, CH_2), 3.82 m (2H, OCH_2), 7.05 s (2H, CH_{Ar}). ^{13}C NMR (101 MHz, $CDCl_3$, δ, ppm, *J*/Hz): 10.1 (C^{10}), 14.0 (C^{23}), 22.5 (C^{22}), 22.7 (C^{17}), 23.8 (C^9), 25.9 (C^{13}), 26.2 (C^{18}), 29.3 (C^{19-20}), 30.3 (C^6), 31.7 (C^{21}), 34.2 (C^5), 35.5 (C^7 J_{PC} 124.0), 37.4 (C^{12}), 51.2 (C^{15}), 62.3 (C^{14}), 64.5 (C^{16}), 66.2 (C^8), 122.7 (C^4), 126.4 (C^3), 136.1 (C^2), 152.5 (C^1). ^{31}P NMR (243 MHz, $CDCl_3$, δ, ppm, *J*/Hz): 31.2. Elemental analysis. Found, %: C, 59.94; H, 10.12; Br, 12.85; N, 4.63; P, 5.31. $C_{31}H_{60}N_2O_3PBr$. Calculated, %:C, 60.08; H, 9.76; Br, 12.89; N, 4.52; P, 5.00. MS (ESI-

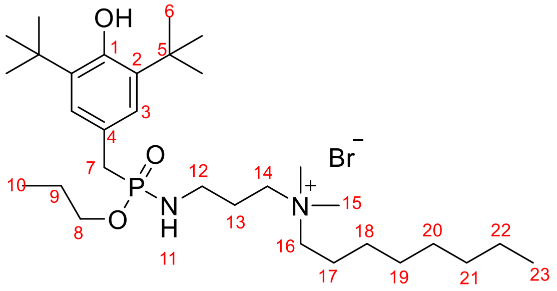

TOF), *m/z* (*rel. int.*, %): Found, 540.1 $[M–Br]^+$. Calculated for the bromide salt: 620.1 $[M+H]^+$.

***N*-(3-(3,5-di-*tert*-butyl-4-hydroxybenzyl)(methoxy)phosphoryl(amino)propyl)-*N*,*N*-dimethyldecan-1-aminium bromide 12a:** Yield 0.13 g (92%). IR, ν, cm^{-1}: 982 (P–O–C_{alk}), 1288 (P=O), 1641 (C=C_{Ar}), 3656 (OH). ^{1}H NMR (400 MHz, $CDCl_3$, δ, ppm., *J*/Hz): 0.91 t (3H, CH_3; J_{HH} 8.0), 1.32 br m (14H, CH_2), 1.44 s (18H, $(CH_3)_3$), 1.77 br m (2H, CH_2), 1.90 br m (2H, CH_2), 2.98 br m (2H, CH_2), 3.09, 3.10 s (6H, $N(CH_3)_2$), 3.14 d (2H, PCH_2, J_{PH} 19.0), 3.36 br m (4H, CH_2), 3.68 d (3H, OCH_3, J_{PH} 8.0), 7.13 s (2H, CH_{Ar}, J_{PH} 2.1). ^{13}C NMR (101 MHz, *Methanol-d*$_4$, δ, ppm, *J*/Hz): 13.1 (C^{22}), 22.2 (C^{21}), 22.3 (C^{14}), 25.0 (C^{10}), 26.0 (C^{15}), 29.2 (C^{16-19}), 29.5 (C^6), 31.6 (C^{20}), 33.4 (C^5), 34.3 (C^7, J_{PC} 126.0), 37.3 (C^9), 50.1 (C^{12}), 50.6 (C^8), 61.8 (C^{13}), 64.3 (C^{11}), 122.3 (C^4), 126.1 (C^3), 138.0 (C^2), 152.8 (C^1). ^{31}P NMR (243 MHz, $CDCl_3$, δ, ppm, *J*/Hz): 33.6. Elemental analysis. Found, %: C, 60.11; H, 9.88; Br, 12.21; N, 4.47; P, 4.95. $C_{31}H_{60}N_2O_3PBr$. Calculated, %:C, 60.08; H, 9.76; Br, 12.89; N, 4.52; P, 5.00. MS (ESI-TOF), *m/z* (*rel. int.*, %): Found, 539.8 $[M–Br]^+$. Calculated for the bromide salt: 620.1 $[M+H]^+$.

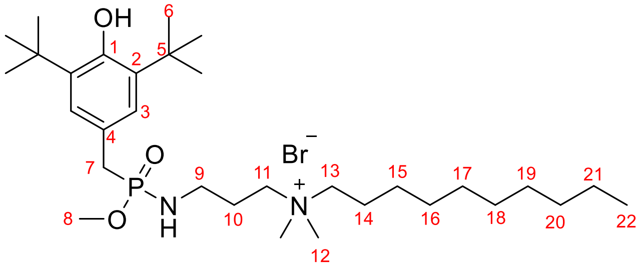

***N*-(3-(3,5-Di-*tert*-butyl-4-hydroxybenzyl)(ethoxy)phosphoryl(amino)propyl)-*N*,*N*-dimethyldecan-1-aminium bromide 12b:** Yield 0.2 g (97%). IR (nujol), ν, cm^{-1}: 1042 (P–O–C_{alk}), 1318 (P=O), 1391-1467 ($C_{10}H_{21}$), 1630 (C=C_{Ar}), 3645 (OH). ^{1}H NMR(400 MHz, $CDCl_3$, δ, ppm., *J*/Hz): 0.82 br t (3H, CH_3, J_{HH} 6.1), 1.20 – 1.23 br m (14H, CH_2; 3H, CH_3), 1.37 s (18H, CH_3), 1.66 br m (2H, CH_2), 1.92 br m (2H, CH_2), 2.78 br m (2H, CH_2), 3.04 br d (2H, CH_2, J_{PH} 19.0), 3.19 s (6H, $N(CH_3)_2$,), 3.37 br m (2H, CH_2), 3.61 br m (2H, CH_2), 3.97 m (2H, OCH_2), 4.09 br s (1H, NH), 7.04 s (2H, CH_{Ar}). ^{13}C NMR (101 MHz, $CDCl_3$, δ, ppm, *J*/Hz): 16.9 (C^{24}), 17.0 (C^9, J_{PC} 6.1), 23.0 (C^{23}), 23.3 (C^{16}), 25.7 (C^{12}), 26.7 (C^{17}), 29.6 (C^{18-21}), 30.9 (C^6), 34.8 (C^{22}), 35.0 (C^5), 35.6 (C^7, J_{PC} 123.8), 37.9 (C^{11}), 51.6 (C^{14}), 61.1 (C^8), 62.9 (C^{13}), 65.1 (C^{13}), 123.3 (C^4), 126.9 (C^3), 136.6 (C^2), 153.0 (C^1). ^{31}P NMR (243 MHz, $CDCl_3$, δ, ppm, *J*/Hz): 31.4. Elemental analysis. Found, %: C, 59.94; H, 10.12; Br, 12.74; N, 4.48; P, 4.93. $C_{32}H_{62}N_2O_3PBr$. Calculated, %:C, 60.65; H, 9.86; Br, 12.61; N, 4.42; P, 4.89. MS (ESI-

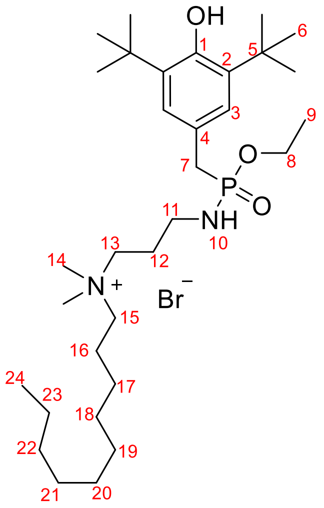

TOF), *m/z* (*rel. int.*, %): Found, 553.8 [M–Br]$^+$. Calculated for the bromide salt: 633.7 [M+H]$^+$.

***N*-(3-(((3,5-di-*tert*-butyl-4-hydroxybenzyl)(*iso*-propoxy)phosphoryl)amino)propyl)-*N*,*N*-dimethylhexadecan-1-aminium bromide 12c:** Yield 0.12 g (81%). IR, ν, cm^{-1}: 993 (P-N), 1111 (P–O–C_{alk}), 1318 (P=O), 1633 (C=C_{Ar}), 3645 (OH). ^{1}H NMR (400 MHz, *Methanol-d$_4$*, δ, ppm, *J*/Hz): 0.91 br s (3H, CH_3), 1.26 br m (3H, CH_3), 1.32 br s (14H, CH_2; 3H CH_3), 1.45 s (18H, $(CH_3)_3$), 1.80 br m (2H, CH_2), 1.92 br m (2H, CH_2), 2.98 br m (2H, CH_2), 3.06 br d (2H, CH_2, J_{PH} 19.2), 3.11 s (6H, $N(CH_3)_2$), 3.39 br m (4H, CH_2), 4.61 br m (1H, CH), 7.13 s (2H, CH_{Ar}). ^{13}C NMR (101 MHz, *Methanol-d$_4$*, δ, ppm, *J*/Hz): 13.9 (C^{24}), 22.9 (C^{23}), 24.0 (C^9), 25.5 (C^{12}), 25.6 (C^{16}), 29.8 (C^{18-21}), 30.3 (C^6), 32.2 (C^{22}), 34.7 (C^5), 36.3 (C^7, J_{PC} 124.8), 37.9 (C^{11}), 50.8 (C^{14}), 62.4 (C^{13}), 64.8 (C^{15}), 69.8 (C^8), 123.3 (C^4), 126.7 (C^3), 138.4 (C^2), 153.3 (C^1). ^{31}P NMR (243 MHz, $CDCl_3$, δ, ppm, *J*/Hz): 33.0. Elemental analysis. Found, %: C, 61.19; H, 9.96; Br, 12.34; N, 4.32; P, 4.78. $C_{33}H_{64}N_2O_3PBr$. Calculated, %:C, 61.23; H, 9.71; Br, 13.11; N, 4.41; P, 4.91. MS (ESI-TOF), *m/z* (*rel. int.*, %): Found, 586.8 [M–Br]$^+$. Calculated for the bromide salt: 647.69 [M+H]$^+$.

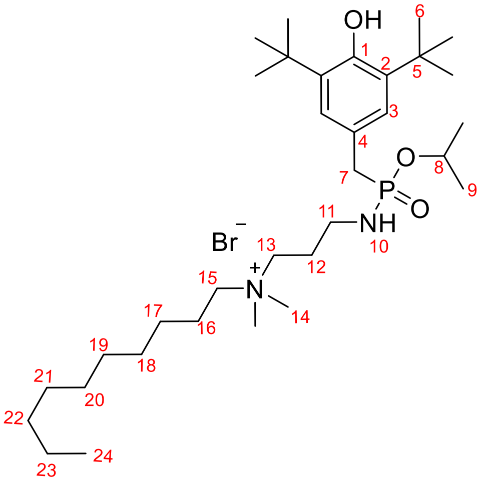

***N*-(3-(3,5-di-*tert*-butyl-4-hydroxybenzyl)(propoxy)phosphoryl(amino)propyl)-*N*,*N*-dimethyldecan-1-aminium bromide 12d:** Yield 0.13 g (98%). ^{1}H NMR (500 MHz, $CDCl_3$, δ, ppm., *J*/Hz): 0.83 m (6H, CH_3), 1.21 m (14H, CH_2), 1.39 s (18H, $(CH_3)_3$), 1.55 m (2H, CH_2), 1.67 m (2H, CH_2), 1.95 m (2H, CH_2), 2.98 br m (2H, CH_2), 3.02 d (2H, CH_2, J_{PH} 20.2), 3.1906 d (6H, $N(CH_3)_2$, J_{HH} 6.8), 3.36 br m (2H, CH_2), 3.59 br m (2H, CH_2), 3.80 m (2H, OCH_2), 7.04 s (2H, CH_{Ar}). ^{13}C NMR (126 MHz, $CDCl_3$, δ, ppm, *J*/Hz): 10.1 (C^{10}), 14.0 (C^{25}), 22.5 (C^{24}), 22.7 (C^{16}), 23.8 (C^9), 25.9 (C^{12}), 26.7 (C^{17}), 29.3 (C^{18-22}), 30.3 (C^6), 31.7 (C^{23}), 34.2 (C^5), 35.5 (C^7), 37.4 (C^{11}), 51.1 (C^{14}), 62.3 (C^{13}), 64.5 (C^{15}), 66.2 (C^8), 122.7 (C^4), 126.4 (C^3), 136.1 (C^2), 152.5 (C^1). ^{31}P NMR (243 MHz, $CDCl_3$, δ, ppm, *J*/Hz): 32.6. Elemental analysis. Found, %: C, 60.5; H, 9.71; Br, 12.26; N, 4.74; P, 5.54. $C_{33}H_{64}N_2O_3PBr$. Calculated, %:C, 61.19; H, 9.96; Br, 12.34; N, 4.32; P, 4.78. MS (ESI-TOF), m/z (*rel. int.*, %): Found, 567.8 [M–Br]$^+$. Calculated for the bromide salt: 647.3 [M+H]$^+$.

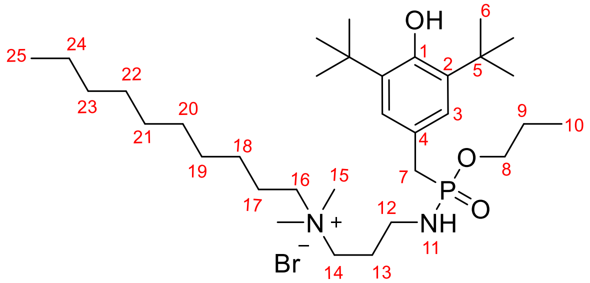

_N_-(3,5-(3,5-di-_tert_-butyl-4-hydroxybenzyl)(methoxy)phosphoryl(amino)propyl)-_N_,_N_-dimethyldodecan-1-aminium bromide 13a: Yield 0.12 g (89%). IR, ν, см$^{-1}$: 1112 (P–O–C$_{alk}$), 1295 (P=O), 1644 (C=C$_{Ar}$), 3634 (OH). ^{1}H NMR (400 MHz, CDCl$_3$, δ, ppm., *J*/Hz): 0.86 t (3H, CH$_3$, J_{HH} 6.6), 1.23 m (18H, CH$_2$), 1.40 s (18H, (CH$_3$)$_3$), 1.68 br m (2H, CH$_2$), 1.96 br m (2H, CH$_2$), 2.99 br m (2H, CH$_2$), 3.06 d (2H, CH$_2$ J_{PH} 19.6), 3.20 s (6H, N(CH$_3$)$_2$), 3.32 -3.38 br m (2H, CH$_2$), 3.60 d (3H, OCH$_3$, J_{PH} 12.0), 7.06 s (2H, CH$_{Ar}$). ^{13}C NMR (101 MHz, CDCl$_3$, δ, ppm, *J*/Hz): 14.5 (C^{24}), 23.1 (C^{23}), 23.3 (C^{10}), 25.7 (C^{15}), 26.8 (C^{14}), 29.9 (C$^{16-21}$), 30.9 (C^6), 32.3 (C^{22}), 34.5 (C^5), 35.7 (C^7, J_{PC} 124.8), 38.0 (C^9), 51.7 (C^{12}), 52.1 (C^8), 63.0 (C^{13}), 65.1 (C^{11}), 122.4 (C^4), 128.1 (C^3), 135.8 (C^2), 153.1 (C^1). ^{31}P NMR(243 MHz, CDCl$_3$, δ, ppm, *J*/Hz): 33.6. Elemental analysis. Found, %: C, 62.14; H, 10.11; Br, 12.01; N, 4.34; P, 4.22. $C_{33}H_{64}N_2O_3PBr$. Calculated, %:C, 61.19; H, 10.21; Br, 11.86; N, 4.22; P, 4.44. MS (ESI-TOF), *m/z* (*rel. int.*, %): Found, 568.4 [M–Br]$^+$. Calculated for the bromide salt: 648.3 [M+H]$^+$.

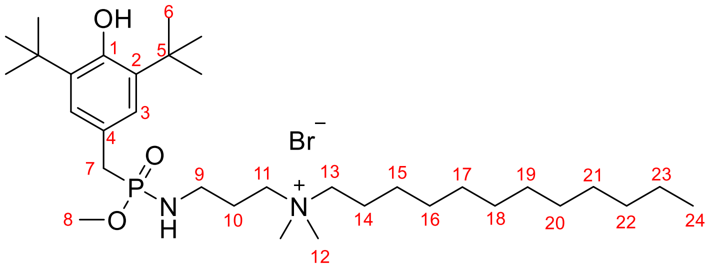

_N_-(3-(3,5-Di-_tert_-butyl-4-hydroxybenzyl)(ethoxy)phosphoryl(amino)propyl)-_N_,_N_-dimethyldodecan-1-aminium bromide 13b: Yield 0.09 g (89%). IR (nujol), ν, см$^{-1}$: 951 (P-N), 1043 (P–O–C$_{alk}$), 1318 (P=O), 1391-1467 ($C_{12}H_{25}$), 1634 (C=C$_{Ar}$), 3645 (OH). ^{1}H NMR (600 MHz, DMSO-d$_6$, δ, ppm, *J*/Hz): 0.76 t (3H, CH$_3$, J_{HH} 7.0), 1.11 t (3H, CH$_3$, J_{HH} 6.0), 1.14 m (18H, CH$_2$), 1.31 s (18H, (CH$_3$)$_3$), 1.60 br m (2H, CH$_2$), 1.85 br m (2H, CH$_2$), 2.93 br m (2H, CH$_2$), 2.97 d (2H, CH$_2$, J_{PH} 19.7), 3.11, 3.12 two s (6H, N(CH$_3$)$_2$,), 3.28 m (2H, CH$_2$), 3.53 m (2H, CH$_2$), 3.84 m (2H, OCH$_2$), 4.13 br m (1H, NH), 5.06 s (1H, OH), 6.97 s (2H, CH$_{Ar}$). ^{13}C NMR (150 MHz, CDCl$_3$, δ, ppm, *J*/Hz): 14.5 (C^{26}), 16.9 (C^9), 23.0 (C^{25}), 23.2 (C^{16}), 25.6 (C^{12}), 25.7 (C^{17}), 29.6 (C$^{18-23}$), 30.8 (C^6), 32.3 (C^{24}), 34.7 (C^5), 36.2 (C^7, J_{PC} 123.6), 37.9 (C^{11}), 51.5 (C^{14}), 61.0 (C^8), 62.8 (C^{13}), 65.0 (C^{15}), 123.3 (C^4), 126.9 (C^3), 136.6 (C^2), 153.0 (C^1). ^{31}P NMR(243 MHz, CDCl$_3$, δ, ppm, *J*/Hz): 31.3. Elemental analysis. Found, %: C, 61.74; H, 10.01; Br, 12.11; N, 4.25; P, 4.62. $C_{34}H_{66}N_2O_3PBr$. Calculated, %:C, 61.71; H, 10.05; Br, 12.07; N, 4.23; P, 4.68. MS (ESI-TOF), *m/z* (*rel. int.*, %): Found, 582.3 [M–Br]$^+$. Calculated for the bromide salt: 662.3 [M+H]$^+$

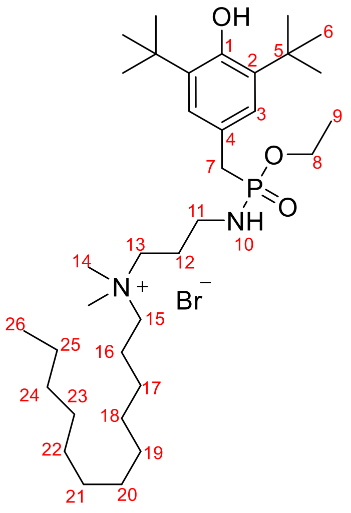

***N*-(3-(3,5-Di-*tret*-butyl-4-hydroxybenzyl)(*iso*-propoxy)phosphoryl(amino)propyl)-*N*,*N*-dimethyldecan-1-aminium bromide 13c:** Yield 0.13 g (90%). IR, *ν*, см$^{-1}$: 997 (P-N), 1116 (P–O–C$_{alk}$), 1321 (P=O), 1644 (C=C$_{Ar}$), 3640 (OH). ^{1}H NMR (500 MHz, CDCl$_3$, δ, ppm, *J*/Hz): 0.86 br t (3H, CH$_3$, J_{HH} 5.9), 1.14 d (3H, CH$_3$, J_{HH} 5.0), 1.23 m (12H, CH$_2$, 3H, CH$_3$), 1.40 s (18H, (CH$_3$)$_3$), 1.69 br m (2H, CH$_2$), 1.94 br m (2H, CH$_2$), 2.98 br m (2H, CH$_2$), 3.02 br d (2H, PCH$_2$, J_{PH} 18.0), 3.22 br s (6H, N(CH$_3$)$_2$), 3.36 br m (2H, CH$_2$), 3.62 br m (2H, CH$_2$), 4.51 m (1H, OCH), 7.06 c (2H, CH$_{Ar}$). ^{13}C NMR (126 MHz, CDCl$_3$, δ, ppm, *J*/Hz): 14.0 (C^{26}), 22.6 (C^{25}), 22.7 (C^{16}), 24.1 (C^{9}), 25.2 (C^{12}), 26.2 (C^{17}), 29.5 (C$^{18-23}$), 30.3 (C^{6}), 31.8 (C^{24}), 34.3 (C^{5}), 36.1 (C^{7}, J_{PC} 123.2), 37.5 (C^{11}), 51.2 (C^{14}), 62.3 (C^{13}), 64.5 (C^{15}), 69.4 (C^{8}), 122.8 (C^{4}), 126.5 (C^{3}), 136.0 (C^{2}), 152.5 (C^{1}). ^{31}P NMR (243 MHz, CDCl$_3$, δ, ppm, *J*/Hz): 31.3. Elemental analysis. Found, %: C, 62.10; H, 10.05; Br, 11.12; N, 4.21; P, 4.74. $C_{35}H_{68}N_2O_3PBr$. Calculated, %:C, 62.20; H, 10.14; Br, 11.82; N, 4.15; P, 4.58. MS (ESI-TOF), m/z (*rel. int.*, %): Found, 582.1 [M–Br]$^+$. Calculated for the bromide salt: 675.8 [M+H]$^+$.

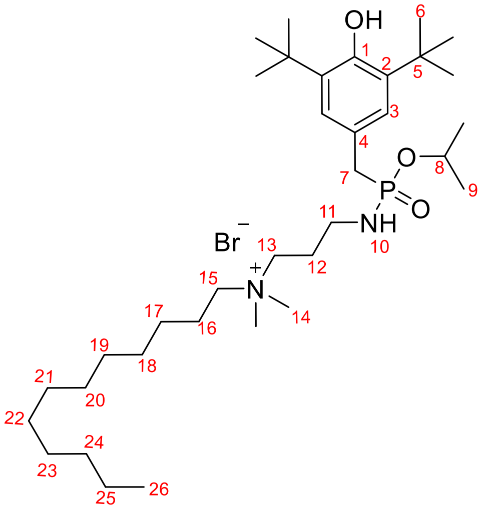

***N*-(3-(3,5-di-*tert*-butyl-4-hydroxybenzyl)(propoxy)phosphoryl(amino)propyl)-*N*,*N*-dimethyldodecane-1-aminium bromide 13d:** Yield 0.13 g (98%). ^{1}H NMR (400 MHz, CDCl$_3$, δ, ppm., *J*/Hz): 0.84 t (3H, CH$_3$; 3H, CH$_3$, J_{HH} 6.0), 1.27 – 1.31 m (18H, CH$_2$), 1.39 s (18H, (CH$_3$)$_3$), 1.56 m (2H, CH$_2$), 1.68 m (2H, CH$_2$), 1.96 m (2H, CH$_2$), 2.98 m (2H, CH$_2$), 3.03 d (2H, CH$_2$, J_{PH} 19.5), 3.20 s (6H, N(CH$_3$)$_2$), 3.37 m (2H, CH$_2$), 3.59 m (2H, CH$_2$), 3.82 m (2H, OCH$_2$), 7.05 s (2H, CH$_{Ar}$). ^{13}C NMR (101 MHz, CDCl$_3$, δ, ppm, *J*/Hz): 10.1 (C^{10}), 14.0 (C^{27}), 22.5 (C^{26}), 22.7 (C^{16}), 23.8 (C^{9}), 25.9 (C^{12}), 26.7 (C^{17}), 29.3 (C$^{19-24}$), 30.3 (C^{6}), 31.7 (C^{25}), 34.2 (C^{5}), 35.5 (C^{7}), 37.4 (C^{11}), 51.1 (C^{14}), 62.3 (C^{13}), 64.5 (C^{15}), 66.2 (C^{8}), 122.7 (C^{4}), 126.4 (C^{3}), 136.1 (C^{2}), 152.5 (C^{1}). ^{31}P NMR (243 MHz, CDCl$_3$, δ, ppm, *J*/Hz): 31.2. Elemental analysis. Found, %: C, 62.30; H, 9.91; Br, 11.94; N, 4.33; P, 4.12. $C_{35}H_{68}N_2O_3PBr$. Calculated, %:C, 62.20; H, 10.14; Br, 11.82; N, 4.15; P, 4.58. MS (ESI-TOF), *m/z* (*rel. int.*, %): Found, 595.4 [M–Br]$^+$. Calculated for the bromide salt: 675.8 [M+H]$^+$.

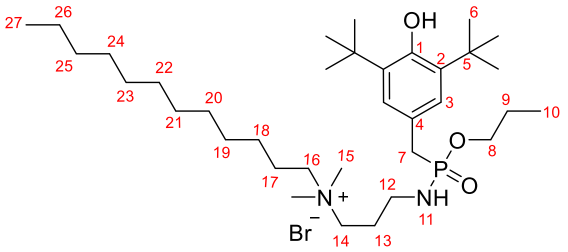

***N*-(3-((((3,5-di-*tert*-butyl-4-hydroxybenzyl)(methoxy)phosphoryl)amino)propyl)-*N*,*N*-dimethyltetradecan-1-aminium bromide 14a:** Yield 0.14 g (95%). IR, ν, cм$^{-1}$: 1078 (P–O–C$_{alk}$), 1304 (P=O), 1595 (C=C$_{Ar}$), 3628 (OH). ^{1}H NMR (400 MHz, CDCl$_3$, δ, ppm., *J*/Hz): 0.86 t (3H, CH$_3$, J_{HH} 6.0), 1.23 m (22H, CH$_2$), 1.40 s (18H, (CH$_3$)$_3$), 1.68 br m (2H, CH$_2$), 1.97 br m (2H, CH$_2$), 2.98 br m (2H, CH$_2$), 3.04 br d (2H, PCH$_2$, J_{PH} 19.0), 3.20 s (6H, N(CH$_3$)$_2$), 3.31 br m (2H, (CH$_2$), 3.50 br m (2H, CH$_2$), 3.60 d (6H, OCH$_3$, J_{PH} 8.0), 4.35 br s (1H, NH), 5.15 br s (1H, OH), 7.06 s (2H, CH$_{Ar}$). ^{13}C NMR (101 MHz, CDCl$_3$, δ, ppm, *J*/Hz): 14.3 (C^{26}), 22.7 (C^{25}), 25.2 (C^{10}), 26.3 (C^{14}), 29.2 (C^{15}), 29.3 (C$^{16\text{-}23}$), 29.4 (C^6), 32.4 (C^{24}), 34.1 (C^5), 35.1 (C^7), 37.4 (C^9), 51.8 (C^{12}), 52.3 (C^8), 62.4 (C^{13}), 64.6 (C^{11}), 122.5 (C^4), 126.4 (C^3), 136.1 (C^2), 152.5 (C^1). ^{31}P NMR (243 MHz, CDCl$_3$, δ, ppm, *J*/Hz): 33.6. Elemental analysis. Found, %: C, 62.14; H, 10.11; Br, 12.01; N, 4.34; P, 4.22. $C_{35}H_{68}N_2O_3PBr$. Calculated, %:C, 62.20; H, 10.14; Br, 11.85; N, 4.12; P, 4.58. MS (ESI-TOF), *m*/*z* (*rel. int.*, %): Found, 595.9 [M–Br]$^+$. Calculated for the bromide salt: 675.8 [M+H]$^+$.

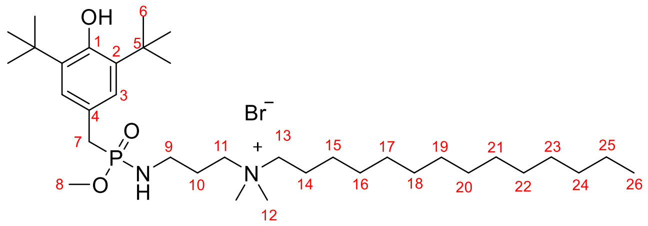

***N*-(3-(((3,5-di-*tert*-butyl-4-hydroxybenzyl)(ethoxy)phosphoryl)amino)propyl)-*N*,*N*-dimethyltetradecan-1-aminium bromide 14b:** Yield 0.12 g (93%). IR (nujol), ν, cм$^{-1}$: 934 (P-N), 1011 (P–O–C$_{alk}$), 1312 (P=O), 1615 (C=C$_{Ar}$), 3633 (OH). ^{1}H NMR (600 MHz, CDCl$_3$, δ, ppm., *J*/Hz): 0.85 t (3H, CH$_3$, J_{HH} 7.0), 1.17 t (3H, CH$_3$, J_{HH} 7.0), 1.22 m (22H, CH$_2$), 1.39 s (18H, (CH$_3$)$_3$), 1.66 br m (2H, CH$_2$), 1.93 br m (2H, CH$_2$), 2.97 br m (2H, CH$_2$), 3.04 d (2H, CH$_2$, J_{PH} 20.4), 3.21, 3.22 two s (6H, N(CH$_3$)$_2$), 3.36 br m (2H, CH$_2$), 3.65 br m (2H, CH$_2$), 3.91 dq (2H, OCH$_2$, J_{HH} 7.3, J_{PH} 2.0), 4.10 br s (1H, NH), 5.09 s (1H, OH), 7.05 s (2H, CH$_{Ar}$). ^{13}C NMR (101 MHz, CDCl$_3$, δ, ppm, *J*/Hz): 14.0 (C^{28}), 16.4 (C^9, J_{PC} 6.6), 22.6 (C^{27}), 22.7 (C^{16}), 25.2 (C^{12}), 26.2 (C^{17}), 29.2 (C$^{18\text{-}25}$), 30.3 (C^6), 32.8 (C^{26}), 33.9 (C^5), 35.6 (C^7, J_{PC} 123.8), 37.4 (C^{11}), 51.0 (C^{14}), 60.6 (C^8), 62.4 (C^{13}), 64.6 (C^{15}), 122.9 (C^4), 126.4 (C^3), 136.0 (C^2), 152.5 (C^1). ^{31}P NMR (243 MHz, CDCl$_3$, δ, ppm, *J*/Hz): 31.3. Elemental analysis. Found, %: C, 62.31; H, 10.17; Br, 11.44; N, 4.08; P, 4.53. $C_{36}H_{70}N_2O_3PBr$. Calculated, %:C, 62.68; H, 10.23; Br, 11.58; N, 4.06; P, 4.49. MS (ESI-TOF), *m*/*z* (*rel. int.*, %): Found, 610.8 [M–Br]$^+$. Calculated for the bromide salt: 690.4 [M+H]$^+$.

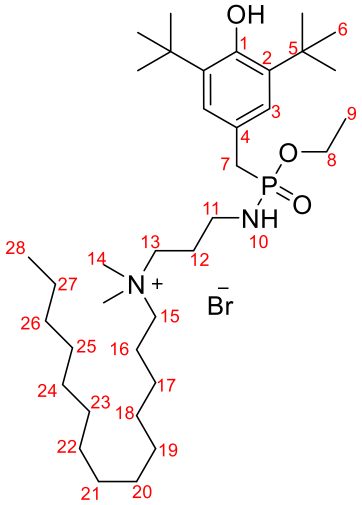

N-(3-(((3,5-di-*tert*-butyl-4-hydroxybenzyl)(*iso*-propoxy)phosphoryl)amino)propyl)-

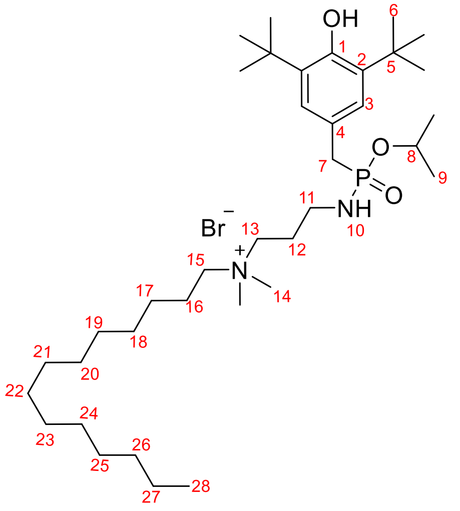

***N,N*-dimethyltetradecan-1-amonium bromide 14c:** Yield 0.13 g (92%). IR , *ν*, см$^{-1}$: 1000 (P-N), 1124 (P–O–C_{alk}), 1320 (P=O), 1645 (C=C_{Ar}), 3631 (OH). ^{1}H NMR (500 MHz, $CDCl_3$, δ, ppm, *J*/Hz): 0.7 br s (3H, CH_3), 1.09 - 1.20 br s (22H, CH_2, 6H, CH_3), 1.30 s (18H, $(CH_3)_3$), 1.54 br s (2H, CH_2), 1.80 br m (2H, CH_2), 2.89 – 3.02 br m (4H, CH_2, PCH_2), 3.06 br s (6H, $N(CH_3)_2$), 3.25 br m (2H, CH_2), 3.44 m (2H, CH_2), 4.38 (1H, OCH), 6.92 s (2H, CH_{Ar}). ^{13}C NMR (126 MHz, $CDCl_3$, δ, ppm, *J*/Hz): 14.4 (C^{28}), 22.6 (C^{27}), 23.0 (C^9), 25.2 (C^{12}), 25.6 (C^{16}), 26.6 (C^{16}), 29.5 (C^{18-25}), 30.3 (C^6), 31.8 (C^{24}), 34.7 (C^5), 36.2 (C^7, J_{PC} 126), 37.9 (C^{11}), 51.5 (C^{14}), 62.6 (C^{13}), 64.8 (C^{15}), 69.5 (C^8), 123.4 (C^4), 126.8 (C^3), 136.7 (C^2), 152.9 (C^1) ^{31}P NMR (243 MHz, $CDCl_3$, δ, ppm, *J*/Hz): 31.0. Elemental analysis. Found, %: C, 63.21; H, 10.21; Br, 11.24; N, 4.44; P, 5.54. $C_{37}H_{72}N_2O_3PBr$. Calculated, %:C, 63.14; H, 10.31; Br, 11.35; N, 3.98; P, 4.40. MS (ESI-TOF), *m*/*z* (*rel. int.*, %): Found, 624.5 $[M–Br]^+$. Calculated for the bromide salt: 703 $[M+H]^+$.

N-(3-(((3,5-di-tert-butyl-4-hydroxybenzyl)(propooxy)phosphoryl)amino)propyl)-N,N-dimethyltetradecan-1-aminium bromide 14d: Yield 0.09 g (84%). ^{1}H NMR (500 MHz,

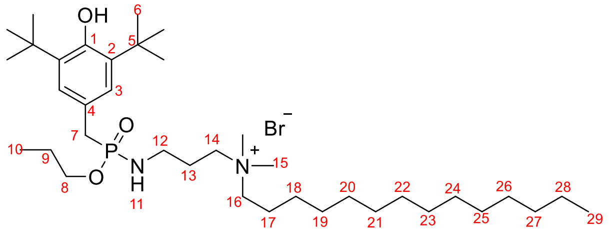

$CDCl_3$, δ, ppm., *J*/Hz): 0.82 br s (3H, CH_3; 3H, CH_3), 1.19 m (22H, CH_2), 1.36 s (18H, $(CH_3)_3$), 1.53 m (2H, CH_2), 1.65 m (2H, 3H, CH_2), 1.91 m (2H, CH_2), 2.96 m (2H, CH_2), 3.01 br d (2H, CH_2, J_{PH} 19.2), 3.18 s (6H, $N(CH_3)_2$), 3.33 br m (2H, (CH_2), 3.58 br m (2H, CH_2), 3.80 m (2H, OCH_2), 4.17 br s (1H, NH), 5.13 s (1H, OH), 7.02 s (2H, CH_{Ar}). ^{13}C NMR (126 MHz, $CDCl_3$, δ, ppm, *J*/Hz): 10.2 (C^{10}), 14.1 (C^{29}), 22.6 (C^{28}), 22.8 (C^9), 23.8 (C^{13}), 26.4 (C^{18}), 29.8 (C^{17-26}), 30.3 (C^6), 31.8 (C^{27}), 34.8 (C^5), 35.5 (C^7, J_{PC} 124.1), 37.4 (C^{12}), 51.1 (C^{14}), 62.3 (C^{13}), 64.6 (C^{15}), 66.4 (C^8), 122.8 (C^4), 126.4 (C^3), 136.0 (C^2), 152.5 (C^1). ^{31}P NMR (243 MHz, $CDCl_3$, δ, ppm, *J*/Hz): 31.3. Elemental analysis. Found, %: C, 63.21; H, 10.14; Br, 11.54; N, 3.76; P, 4.81. $C_{37}H_{72}N_2O_3PBr$. Calculated, %:C, 63.14 H, 10.31; Br, 11.35; N, 3.98; P, 4.40. MS (ESI-TOF), *m*/*z* (*rel. int.*, %): Found, 624.1 $[M–Br]^+$. Calculated for the bromide salt: 704.2 $[M+H]^+$.

N-(3-(((3,5-di-*tert*-butyl-4-hydroxybenzyl)(methoxy)phosphoryl)amino)propyl)-N,N-dimethylhexadecan-1- aminium bromide 15a: Yield 0.05 g (79%). IR, ν, см$^{-1}$: 980 (P-N), 1041 (P–O–C$_{alk}$), 1318 (P=O), 1635 (C=C$_{Ar}$), 3644 (OH). ^{1}H NMR (500 MHz, CDCl$_3$, δ, ppm, *J*/Hz): 0.79 t (3H, CH$_3$, J_{HH} 6.5), 1.19 br m (26H, CH$_2$), 1.34 s (18H, (CH$_3$)$_3$), 1.63 br m (2H, CH$_2$), 1.89 br m (2H, CH$_2$), 2.93 br m (2H, CH$_2$), 3.02 d (2H, CH$_2$, J_{PH} 19.3), 3.15 s (6H, N(CH$_3$)$_2$), 3.31 br m (2H, CH$_2$), 3.52 br m (2H, CH$_2$), 3.54 d (3H, OCH$_3$), 4.40 br s (1H, NH), 5.11 br s (1H, OH), 7.01 s (2H, CH$_{Ar}$). ^{13}C NMR (101 MHz, CDCl$_3$, δ, ppm, *J*/Hz): 14.0 (C^{28}), 22.5 (C^{27}), 22.7 (C^{14}), 26.2 (C^{10}), 28.0 (C^{15}), 29.5 (C$^{16-25}$), 30.3 (C^6), 31.8 (C^{26}), 34.0 (C^5), 35.0 (C^7), 37.4 (C^9), 51.1 (C^{12}), 51.6 (C^8), 62.3 (C^{13}), 64.4 (C^{11}), 122.6 (C^4), 126.4 (C^3), 136.2 (C^2), 152.5 (C^1). ^{31}P NMR (243 MHz, CDCl$_3$, δ, ppm, *J*/Hz): 33.5. Elemental analysis. Found, %: C, 63.11; H, 10.21; Br, 11.11; N, 4.08; P, 4.45. $C_{37}H_{72}N_2O_3PBr$. Calculated, %:C, 63.14; H, 10.31; Br, 11.35; N, 3.98; P, 4.40. MS (ESI-TOF), *m/z* (*rel. int.*, %): Found, 624.3 [M–Br]$^+$. Calculated for the bromide salt: 703.4 [M+H]$^+$.

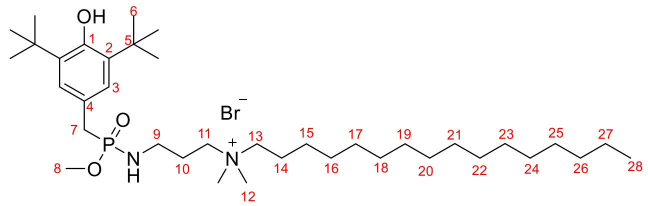

N-(3-(((3,5-di-*tert*-butyl-4-hydroxybenzyl)(ethoxy)phosphoryl)amino)propyl)-N,N-dimethylhexadecan-1- aminium bromide 15b: Yield 0.11 g (89%). IR (nujol), ν, см$^{-1}$: 977 (P-N), 1014 (P–O–C$_{alk}$), 1321 (P=O), 1617 (C=C$_{Ar}$), 3633 (OH). ^{1}H NMR (400 MHz, CDCl$_3$, δ, ppm, *J*/Hz): 0.85 m (3H, CH$_3$,), 1.23 s (26H, CH$_2$, 3H, CH$_3$), 1.39 s (18H, (CH$_3$)$_3$), 1.58 br m (2H, CH$_2$), 1.94 br m (2H, CH$_2$), 2.92 br m (2H, CH$_2$), 3.07 br m (2H, CH$_2$), 3.20 br s (6H, N(CH$_3$)$_2$), 3.35 br m (2H, CH$_2$), 3.57 br m (2H, CH$_2$), 3.93 m (2H, OCH$_2$), 7.05 s (2H, CH$_{Ar}$). ^{13}C NMR (101 MHz, CDCl$_3$, δ, ppm, *J*/Hz): 14.5 (C^{30}), 16.9 (C^9, J_{PC} 6.7), 23.1 (C^{29}), 23.4 (C^{16}), 25.7 (C^{12}), 26.8 (C^{17}), 29.8 (C$^{18-27}$), 30.9 (C^6), 32.4 (C^{28}), 33.3 (C^5), 35.5 (C^7, J_{PC} 123.7), 37.9 (C^{11}), 51.7 (C^{14}), 61.3 (C^8), 62.8 (C^{13}), 65.0 (C^{15}), 123.3 (C^4), 126.9 (C^3), 136.6 (C^2), 153.0 (C^1). ^{31}P NMR (243 MHz, CDCl$_3$, δ, ppm, *J*/Hz): 31.8. Elemental analysis. Found, %: C, 63.23; H, 9.41; Br, 11.23; N, 4.12; P, 4.89. $C_{38}H_{74}N_2O_3PBr$. Calculated, %:C, C, 63.58; H, 9.39; Br, 11.13; N, 3.90; P, 4.31. MS (ESI-TOF), *m/z* (*rel. int.*, %): Found, 638.1 [M–Br]$^+$. Calculated for the bromide salt: 718.1 [M+H]$^+$.

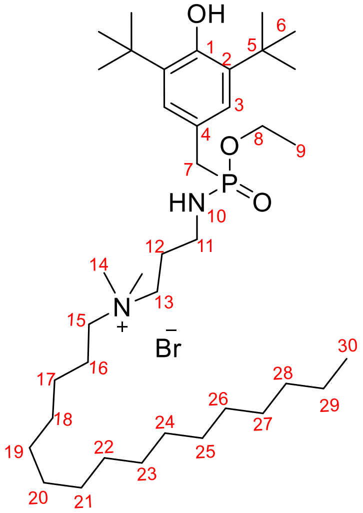

***N*-(3-(3,5-di-*tert*-butyl-4-hydroxybenzyl)(*iso*-propyl)phosphoryl(amino)propyl)-*N,N*-dimethyldodecan-1-aminium bromide 15c:** Yield 0.15gr (89%). IR, *ν*, см$^{-1}$: 1000 (P-N), 1124 (P–O–C$_{alk}$), 1320 (P=O), 1645 (C=C$_{Ar}$), 3631 (OH). ^{1}H NMR (400 MHz, CDCl$_3$, δ, ppm, *J*/Hz): 0.85 m (3H, CH$_3$), 1.12 d (3H, CH$_3$, J_{HH} 5.0), 1.22 s (26H, CH$_2$), 1.24 br s (3H, CH$_3$), 1.38 s (18H, (CH$_3$)$_3$), 1.68 br m (2H, CH$_2$), 1.92 br m (2H, CH$_2$), 2.72, 2.92 two br s (2H, CH$_2$), 3.04 br d (2H, PCH$_2$, J_{PH} 20.0), 3.20 s (6H, N(CH$_3$)$_2$), 3.35 br m (2H, CH$_2$), 3.59 br m (2H, CH$_2$), 4.50 br m (1H, OCH), 7.05 s (2H, CH$_{Ar}$). ^{13}C NMR (101 MHz, CDCl$_3$, δ, ppm, *J*/Hz): 14.5 (C^{30}), 23.1 (C^{29}), 23.3 (C^{16}), 24.6 (C^9), 24.9 (C^{12}), 28.6 (C^{17}), 30.3 (C$^{18-27}$), 30.9 (C^6), 32.3 (C^{28}), 34.8 (C^5), 35.5 (C^7, J_{PC} 124.4), 38.0 (C^{11}), 51.7 (C^{14}), 62.8 (C^{13}), 65.0 (C^{15}), 69.8 (C^8), 123.4 (C^4), 127.0 (C^3), 136.5 (C^2), 153.0 (C^1). ^{31}P NMR (243 MHz, CDCl$_3$, δ, ppm, *J*/Hz): 30.6. Elemental analysis. Found, %: C, 64.21; H, 10.35; Br, 11.01; N, 3.79; P, 4.21. $C_{39}H_{76}N_2O_3PBr$. Calculated, %:C, 64.00; H, 10.47; Br, 10.92; N, 3.83; P, 4.23. MS (ESI-TOF), *m*/*z* (*rel. int.*, %): Found, 653.4 [M–Br]$^+$. Calculated for the bromide salt: 731,93 [M+H]$^+$.

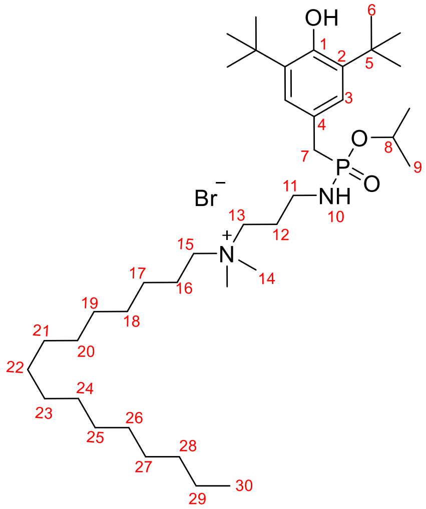

***N*-(3-(((3,5-di-*tert*-butyl-4-hydroxybenzyl)(propoxy)phosphoryl)amino)propyl)-*N,N*-dimethylhexadecan-1-aminium bromide 15d:** Yield 0.1 g (95%). IR, *ν*, см$^{-1}$: 1044 (P–O–C$_{alk}$), 1124 (C–O–C), 1305 (P=O), 1598 (C=C$_{Ar}$), 3628 (OH). ^{1}H NMR (500 MHz, CDCl$_3$ and DMSOd$_6$, δ, ppm, *J*/Hz): 0.80 t (3H, CH$_3$; 3H, CH$_3$), 1.19 s (26H, CH$_2$), 1.34 s (18H, (CH$_3$)$_3$), 1.52 m (2H, CH$_2$), 1.63 m (2H, CH$_2$) ,1.80 m (2H, CH$_2$), 2.88 m (2H, CH$_2$), 2.95 d (2H, PCH$_2$, J_{PH} 19.5), 3.02 s (6H, N(CH$_3$)$_2$), 3.23 br m (2H, CH$_2$), 3.35 m (2H, CH$_2$), 3.77 m (2H, OCH$_2$), 4.55 s (1H, NH), 6.97 s (2H, CH$_{Ar}$)). ^{13}C NMR (126 MHz, CDCl$_3$+DMSOd$_6$, δ, ppm, *J*/Hz): 10.3 (C^{10}), 14.2 (C^{31}), 22.4 (C^{30}), 22.5 (C^{16}), 23.8 (C^9), 25.1 (C^{12}), 26.3 (C^{17}), 29.5 (C$^{18-28}$), 30.6 (C^6), 31.7 (C^{29}), 33.5 (C^7), 34.6 (C^5), 37.5 (C^{11}), 50.7 (C^{14}), 61.7 (C^{13}), 64.1 (C^{15}), 65.5 (C^8), 123.5 (C^4), 123.5 (C^3), 138.4 (C^2), 152.6 (C^1). ^{31}P NMR (243 MHz, Methanole-d$_4$, δ, ppm, *J*/Hz): 31.3. Elemental analysis. Found %: C, 63.97; H, 10.34; Br, 11.05; N, 3.44; P, 4.54. $C_{39}H_{76}N_2O_3PBr$. Calculated, %:C, 64.00; H, 10.47; Br, 10.92; N, 3.83; P, 4.23. MS (ESI-

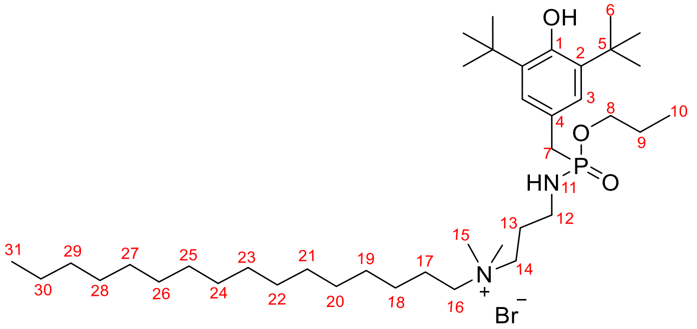

TOF), m/z (*rel. int.*, %): Found, 652.4 [M–Br]$^+$. Calculated for the bromide salt: 732.1 [M+H]$^+$.

The X-ray diffraction data

The high-resolution X-ray diffraction data for the single crystals were collected on a Bruker AXS D8 Quest diffractometer at 150(2) K using Mo Kα radiation (λ = 0.71073 Å). Data collection was performed according to recommended strategies employing an ω/φ-scan mode. The programs used: APEX3 for data collection, SAINT for data reduction, SADABS and TWINABS for multi-scan absorption correction, SHELXT for structure solution, SHELXL for structure refinement by full-matrix least-squares against F^2 [3,4]. CCDC 2474807 contain the supplementary crystallographic data for this paper. Crystallographic data for structures reported in this paper have been deposited with the Cambridge Crystallographic Data Center.

Crystal data for **5b** $C_{22}H_{41}N_2O_3P$ (M = 412.54 g mol^{-1}), monoclinic, space group $P2_1/n$ at 150(2) K: a = 11.3463(12) Å, b = 19.353(2) Å and c = 11.6092(12) Å, β = 95.019(3)°, V = 2539.4(5) Å^3, Z = 4, d_{calc} = 1.076 g cm^{-3}, μ(MoKα)) = 0.130 mm^{-1}, F(000) = 904. A total of 75845 reflections were collected (12302 independent reflections and 7917 independent reflections with $I \geq 2(\sigma)$), GOOF 1.018, final R indices [$I \geq 2(\sigma)$]: R_1 = 0.0844, wR_2 = 0.1601, R indices (all data): R_1 = 0.1414, wR_2 = 0.1916.

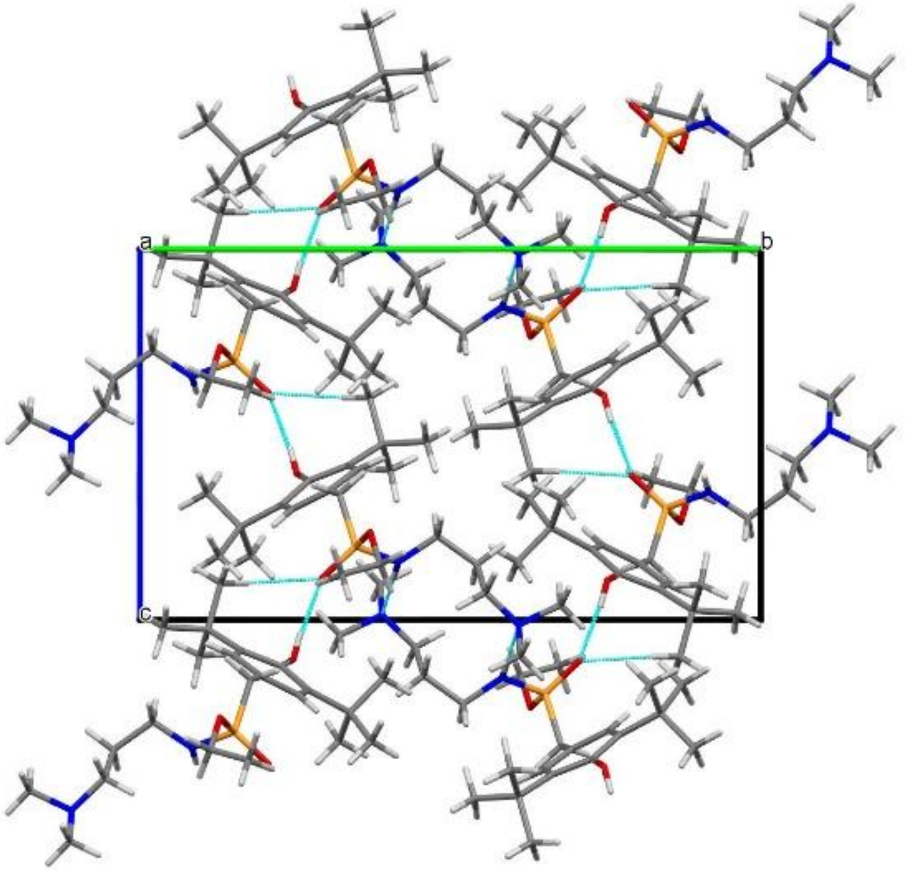

Figure S1. Fragments of crystal packing in of compound **5b** in crystal. Labels a, b, and c denote the unit cell axes. Ellipsoids are shown with 50% probability.

Table S1. Hygrogen bonds in crystals of investigated compounds **5b**

H-bond	D – H, Å	H...A, Å	D...A, Å	D - H...A, ˚
N16–H16…N20	0.88(3)	2.23(3)	2.952(6)	139(2)
C10–H10C…O3	0.98(2)	2.33(2)	2.949(9)	120(2)
C11–H11…O1	0.98(2)	2.35(2)	2.259(7)	154(2)
C13–H13…O3	0.98(2)	2.28(2)	2.950(7)	124(2)
C15–h15C…O3	0.98(2)	2.46(2)	3.091(7)	122(2)
C19–H19B…N16	0.99(2)	2.62(2)	3.016(6)	104(2)

NMR spectra of compounds

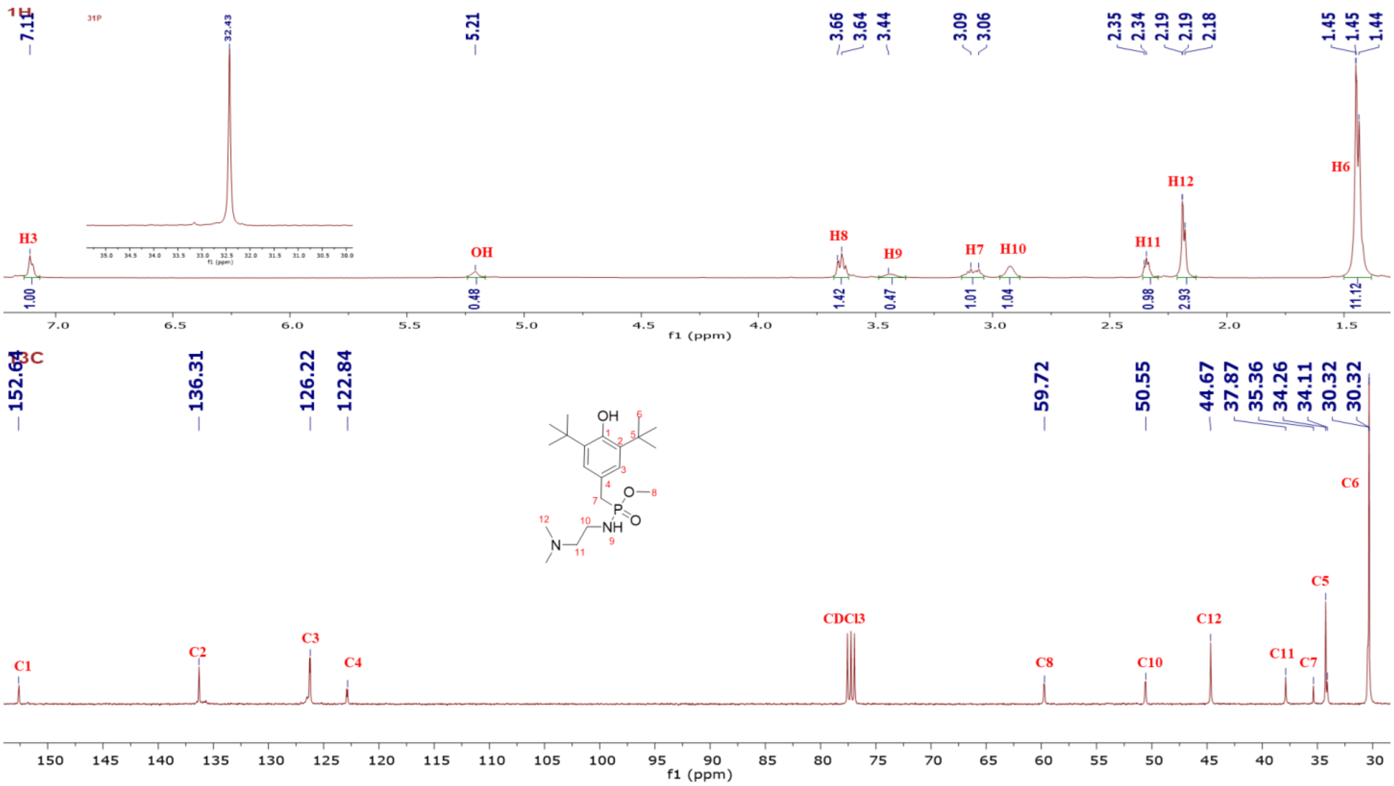

Figure S2. ^{1}H-, ^{13}C, ^{31}P- NMR of compound **4a**

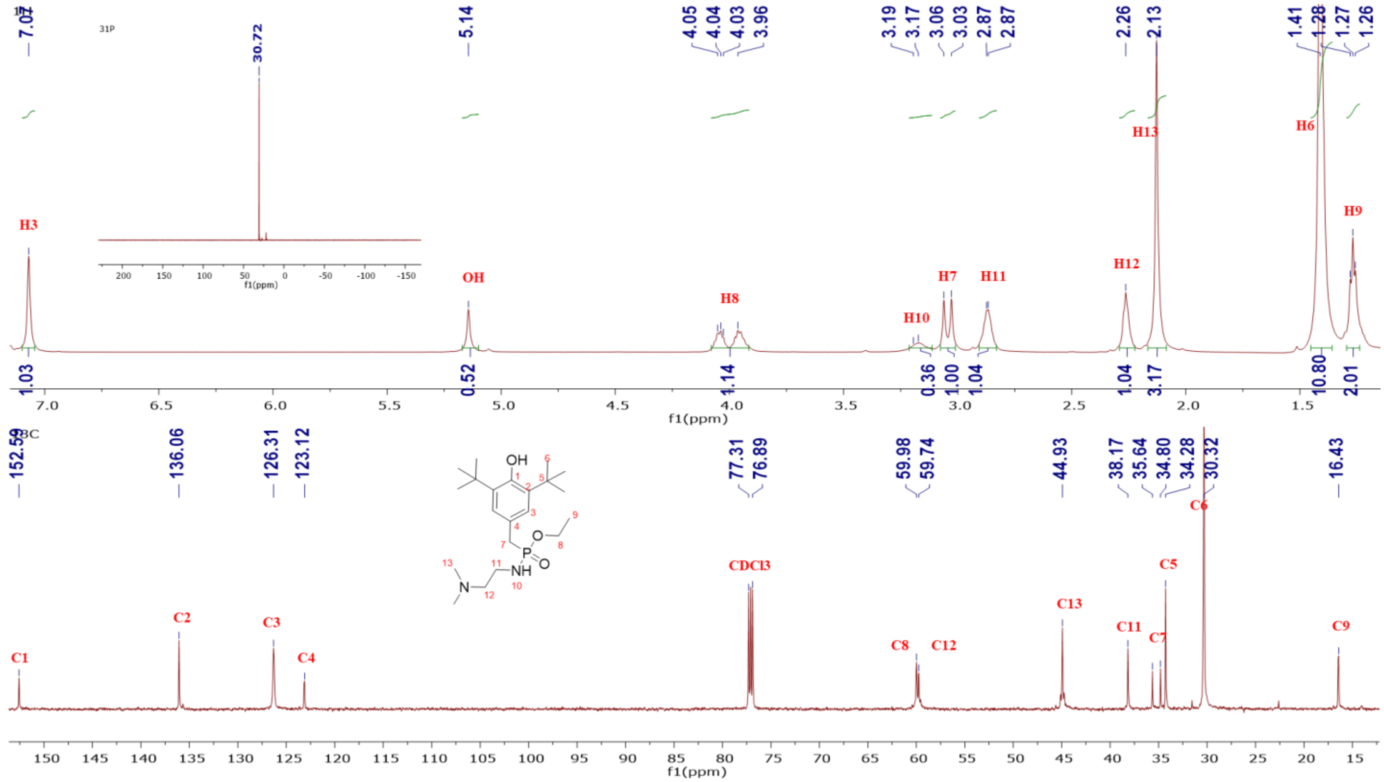

Figure S3. ^{1}H-, ^{13}C, ^{31}P- NMR of compound **4b**

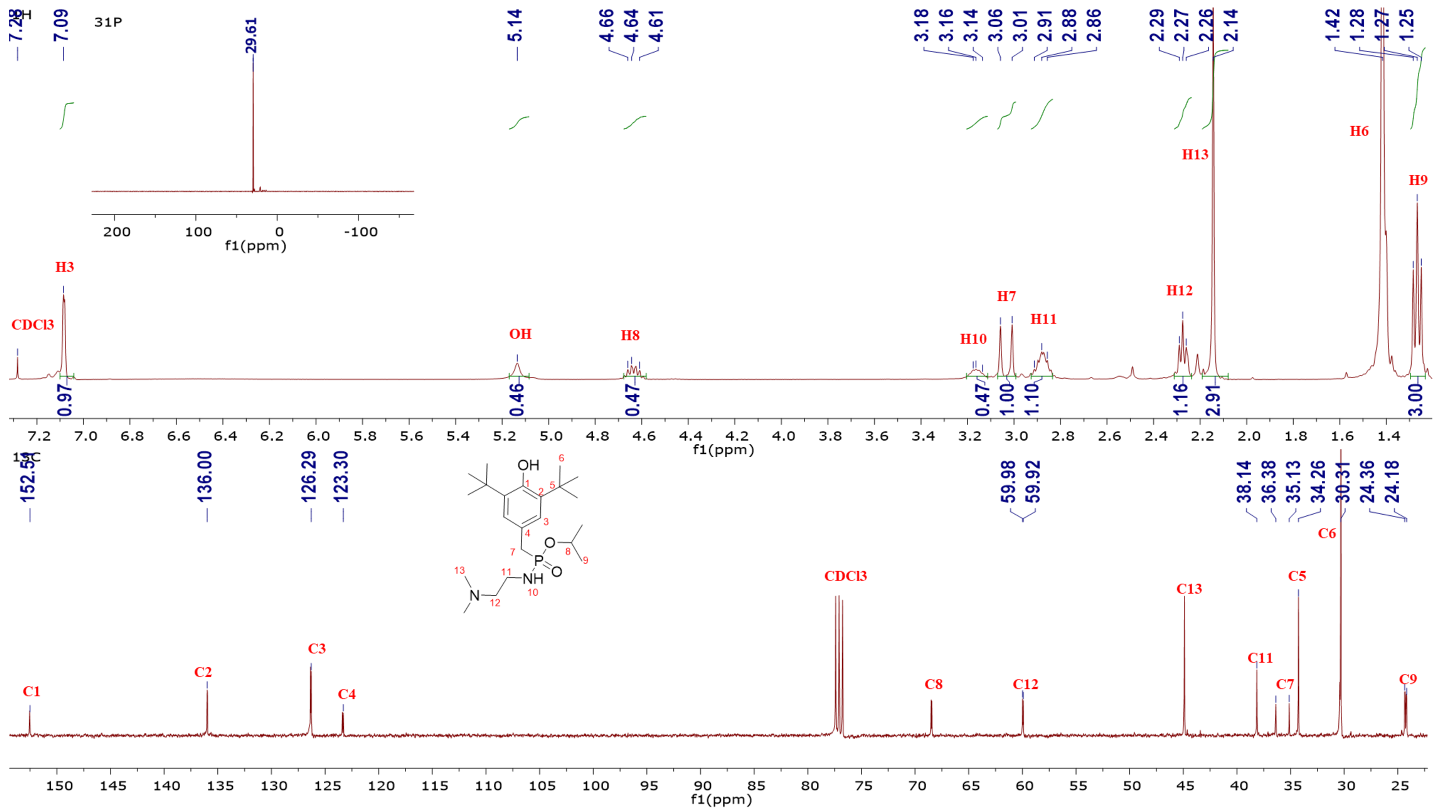

Figure S4. ^{1}H-, ^{13}C, ^{31}P- NMR of compound **4c**

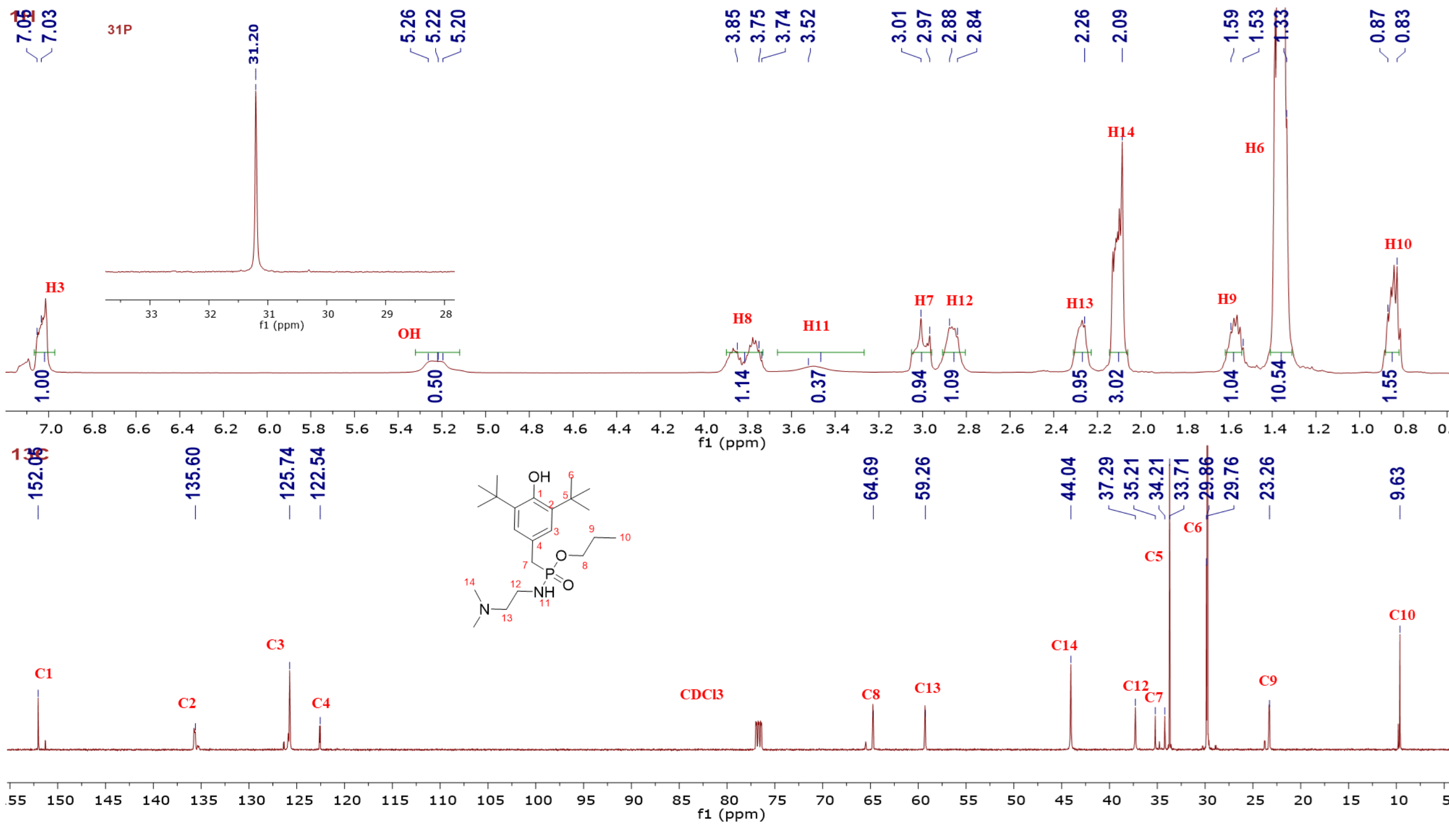

Figure S5. ^{1}H-, ^{13}C, ^{31}P- NMR of compound **4d**

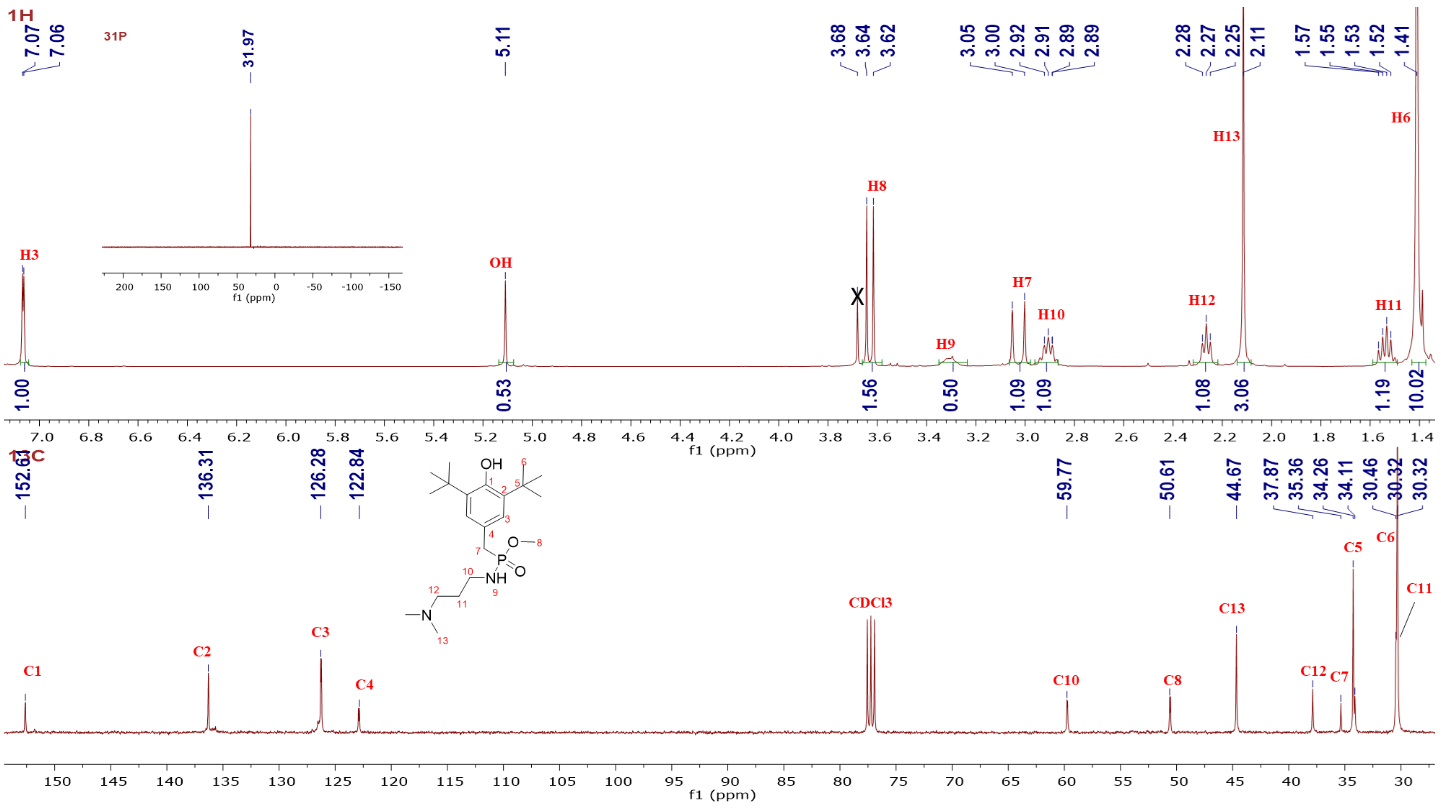

Figure S6. ^{1}H-, ^{13}C, ^{31}P- NMR of compound **5a**

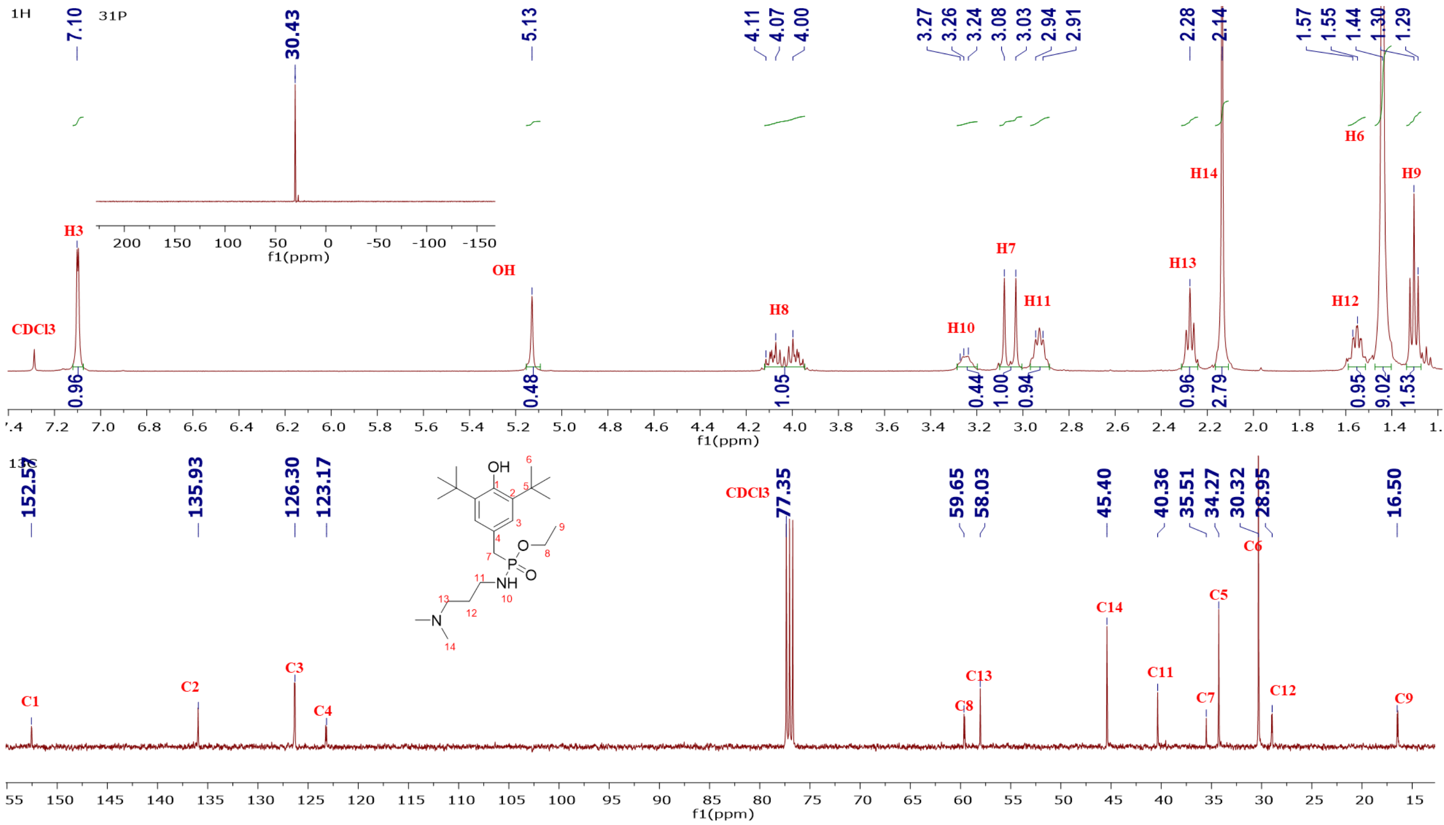

Figure S7. ^{1}H-, ^{13}C, ^{31}P- NMR of compound **5b**

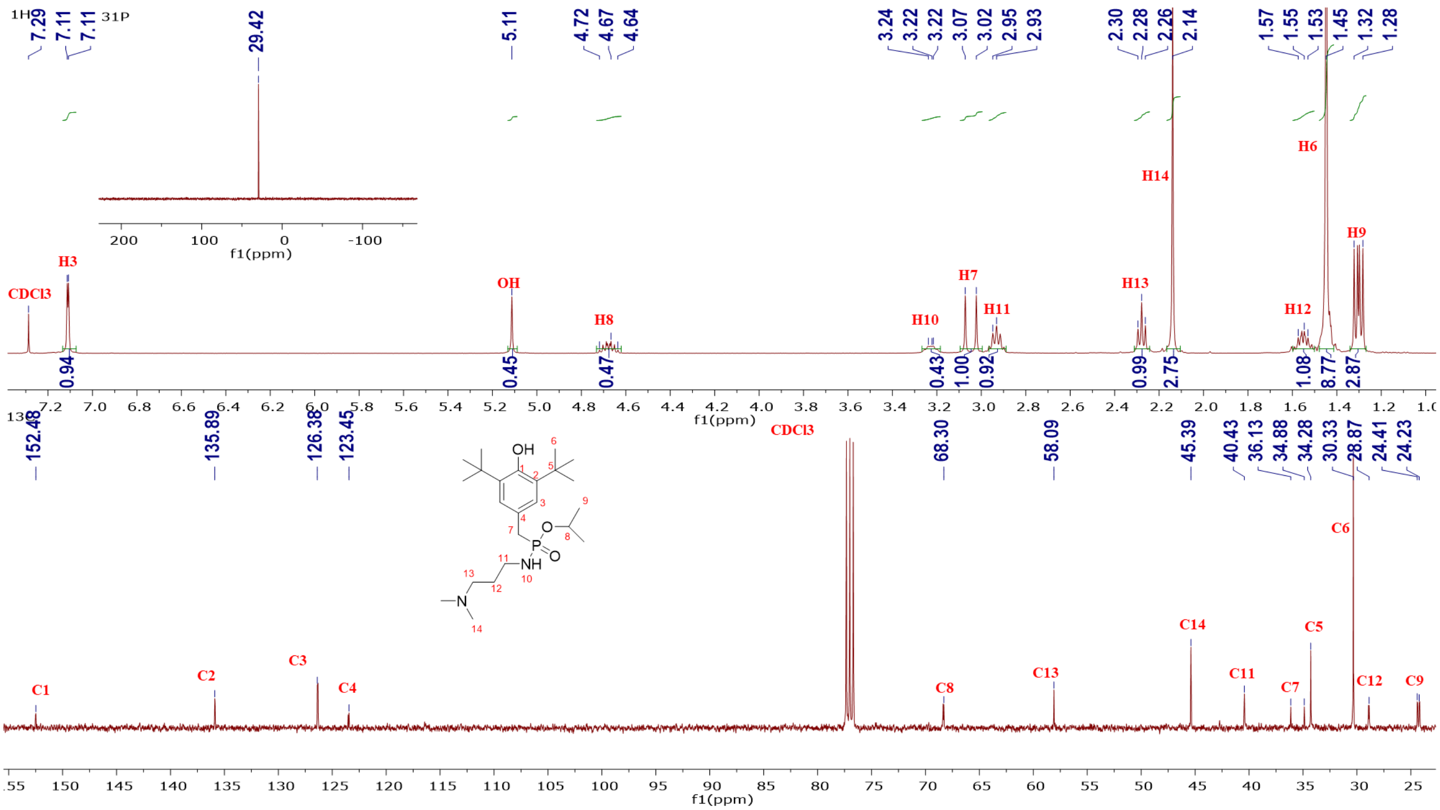

Figure S8. ^{1}H-, ^{13}C, ^{31}P- NMR of compound **5c**

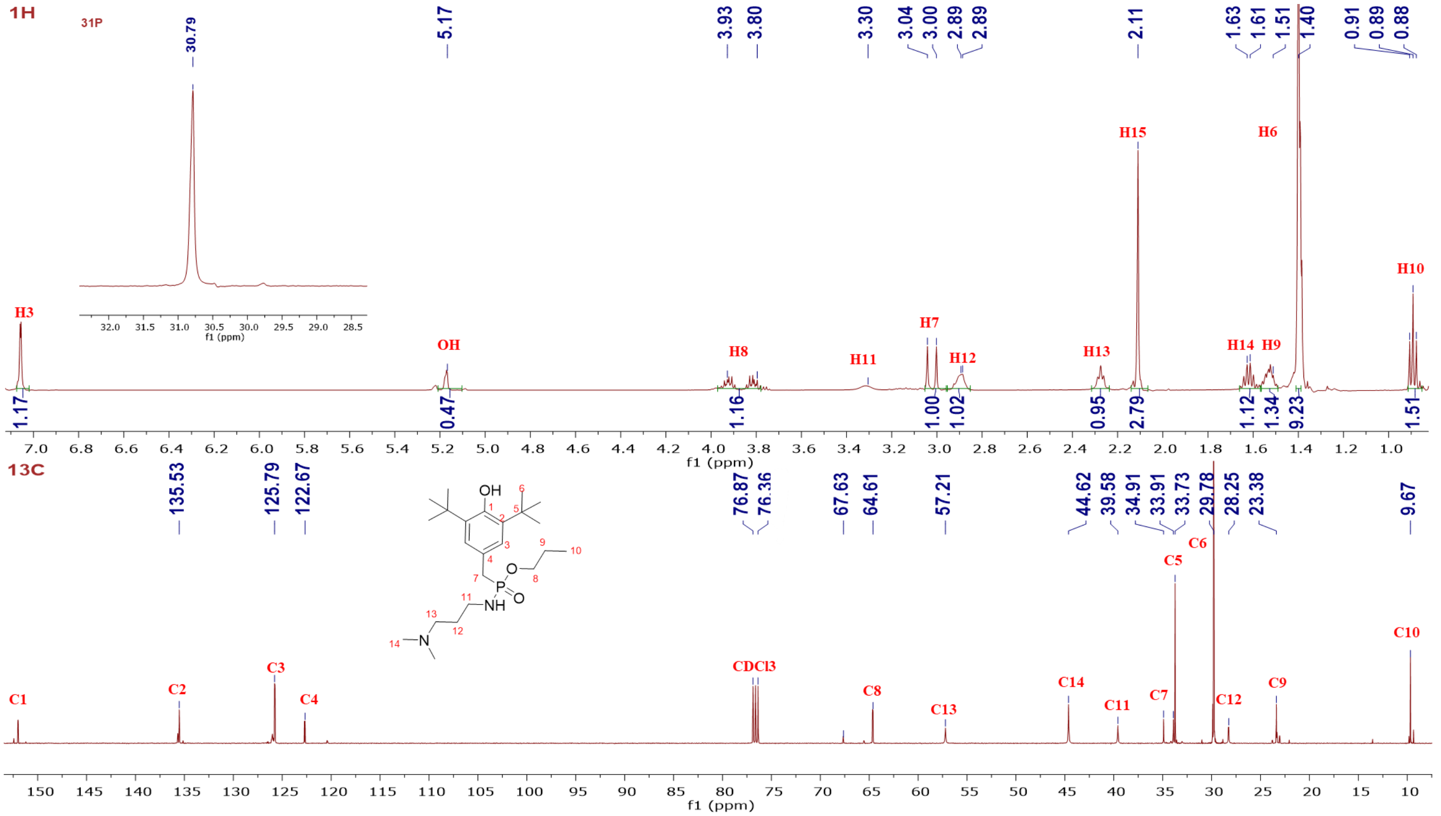

Figure S9. ^{1}H-, ^{13}C, ^{31}P- NMR of compound **5d**

2D correlation NMR experiments ^{1}H-^{1}H COSY, ^{1}H-^{13}C HSQC, ^{1}H-^{13}C HMBC of compounds 5d

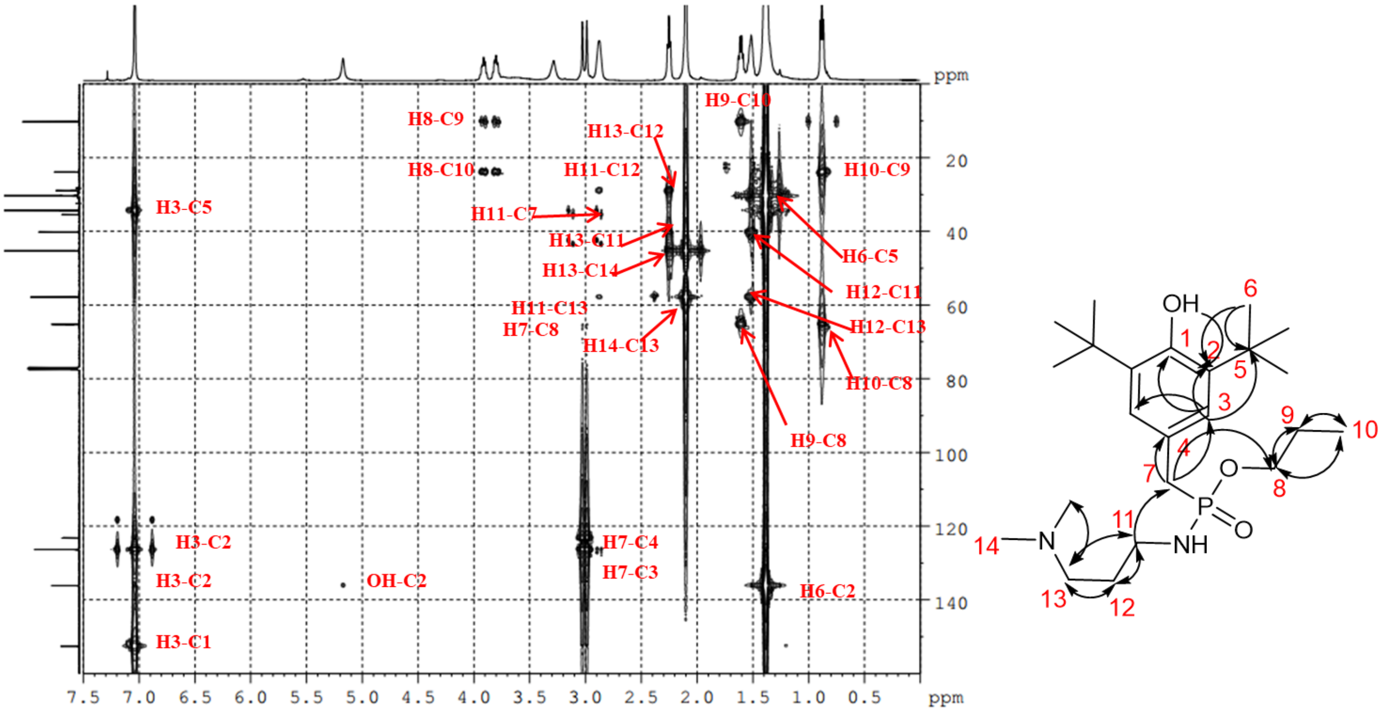

Figure S10. ^{1}H- ^{13}C HMBC correlations

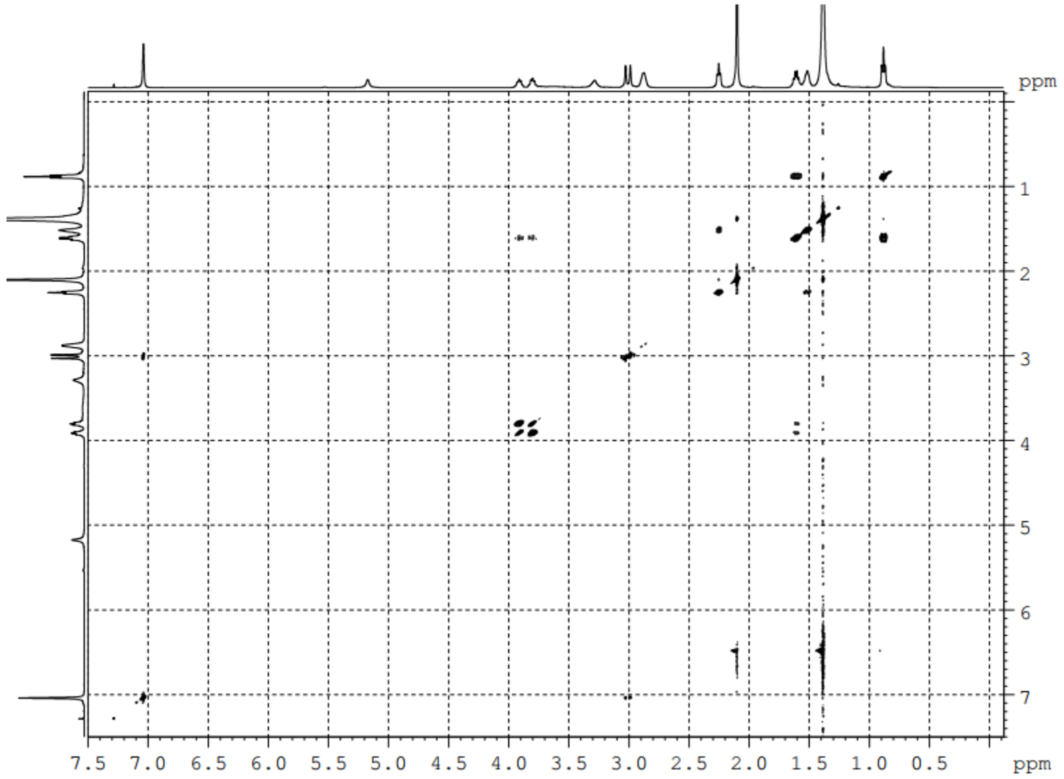

Figure S11. ^{1}H- ^{1}H COSY correlations

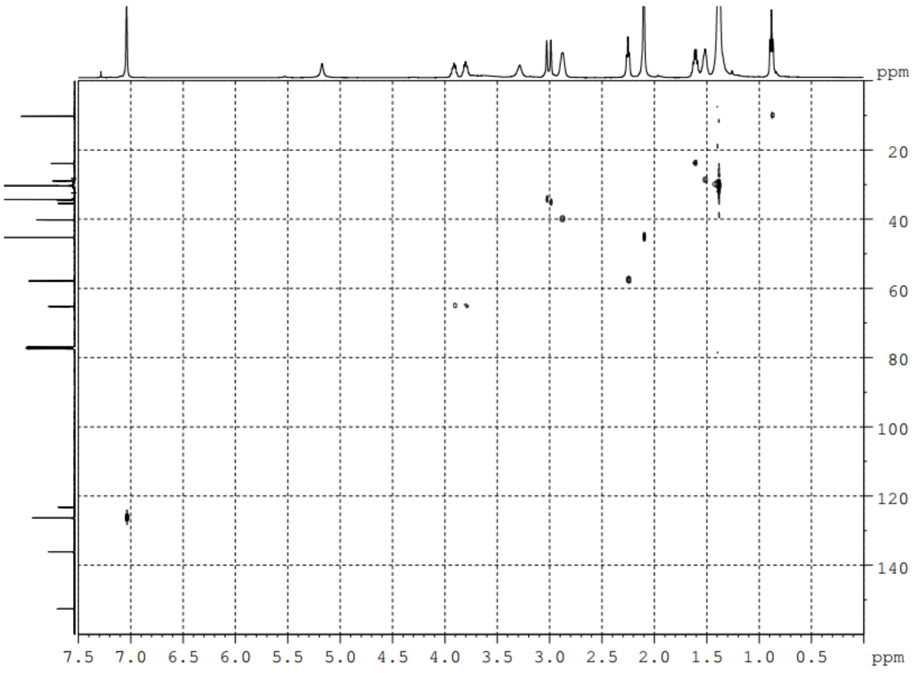

Figure S12. ^{1}H- ^{13}C HSQC correlations

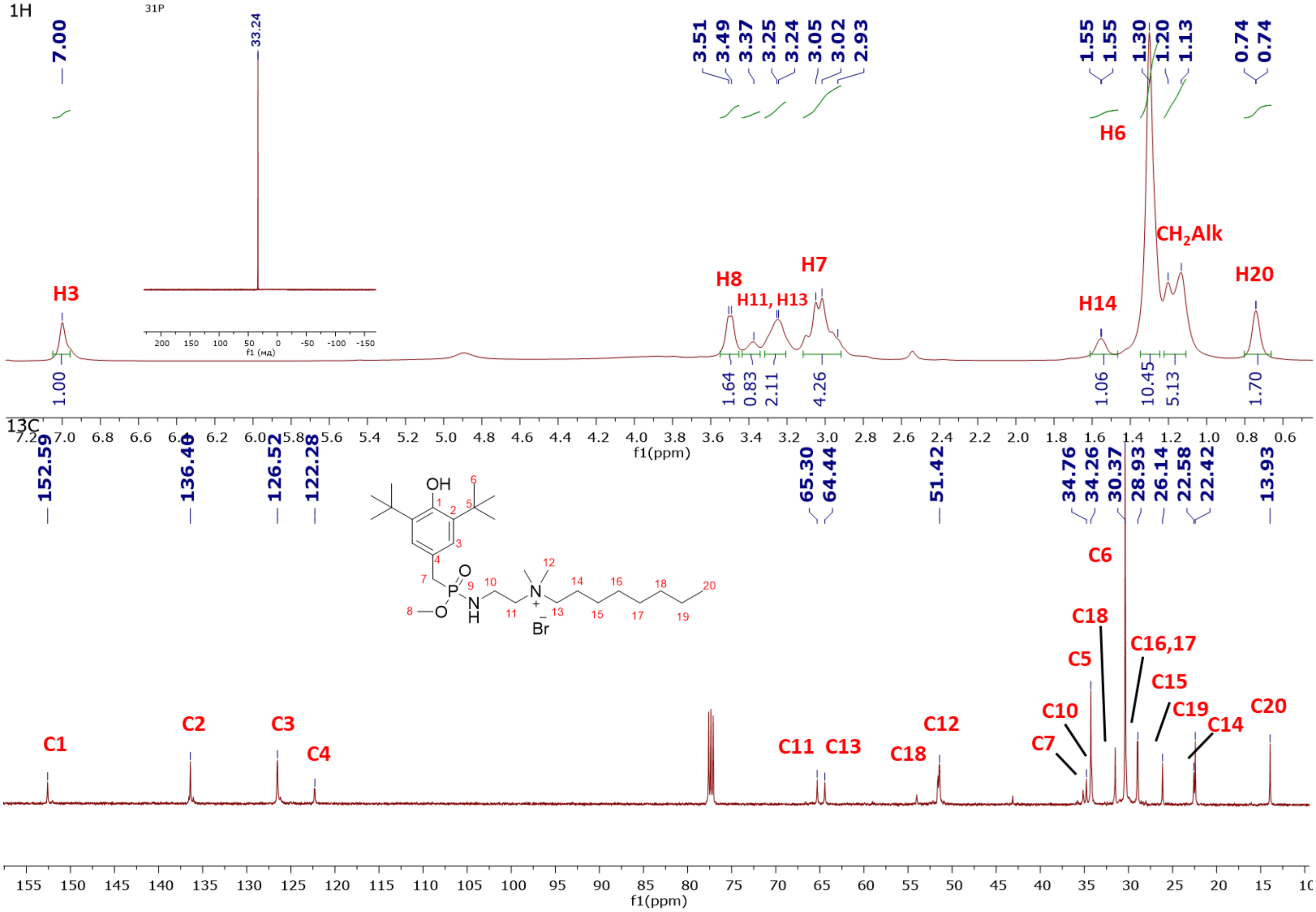

Figure S13. ^{1}H-, ^{13}C, ^{31}P- NMR of compound **6a**

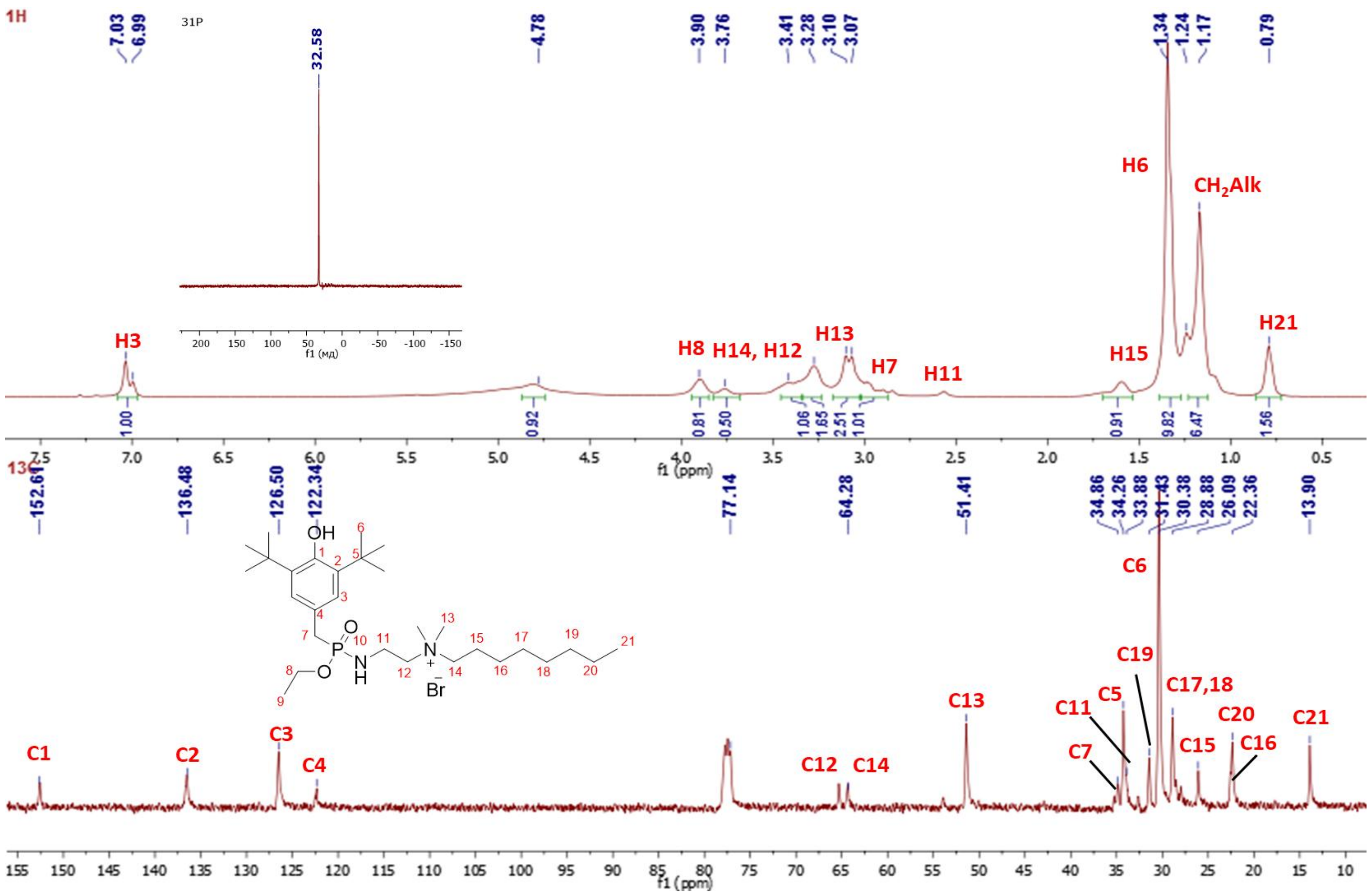

Figure S14. ¹H-, ¹³C, ³¹P- NMR of compound **6b**

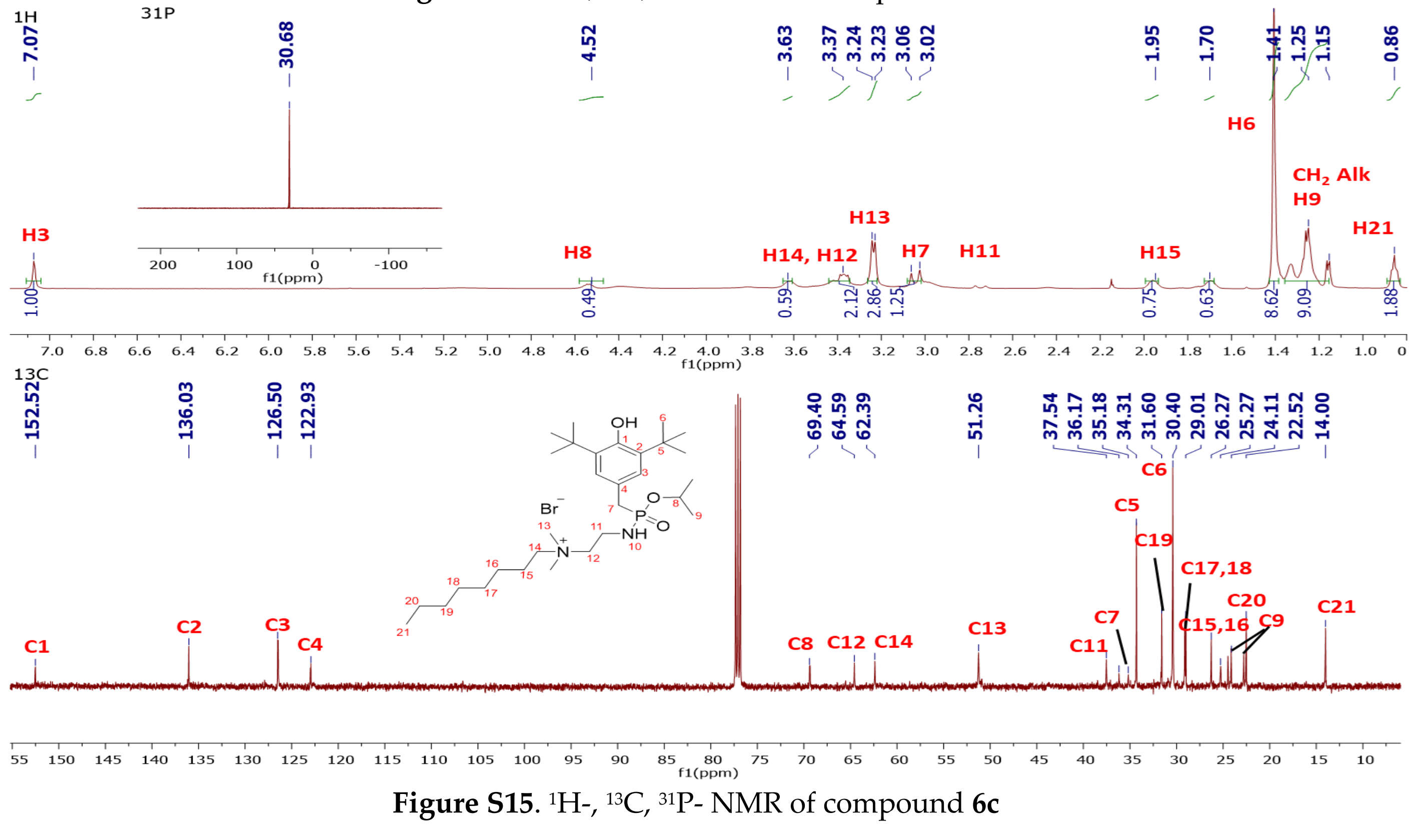

Figure S15. ¹H-, ¹³C, ³¹P- NMR of compound **6c**

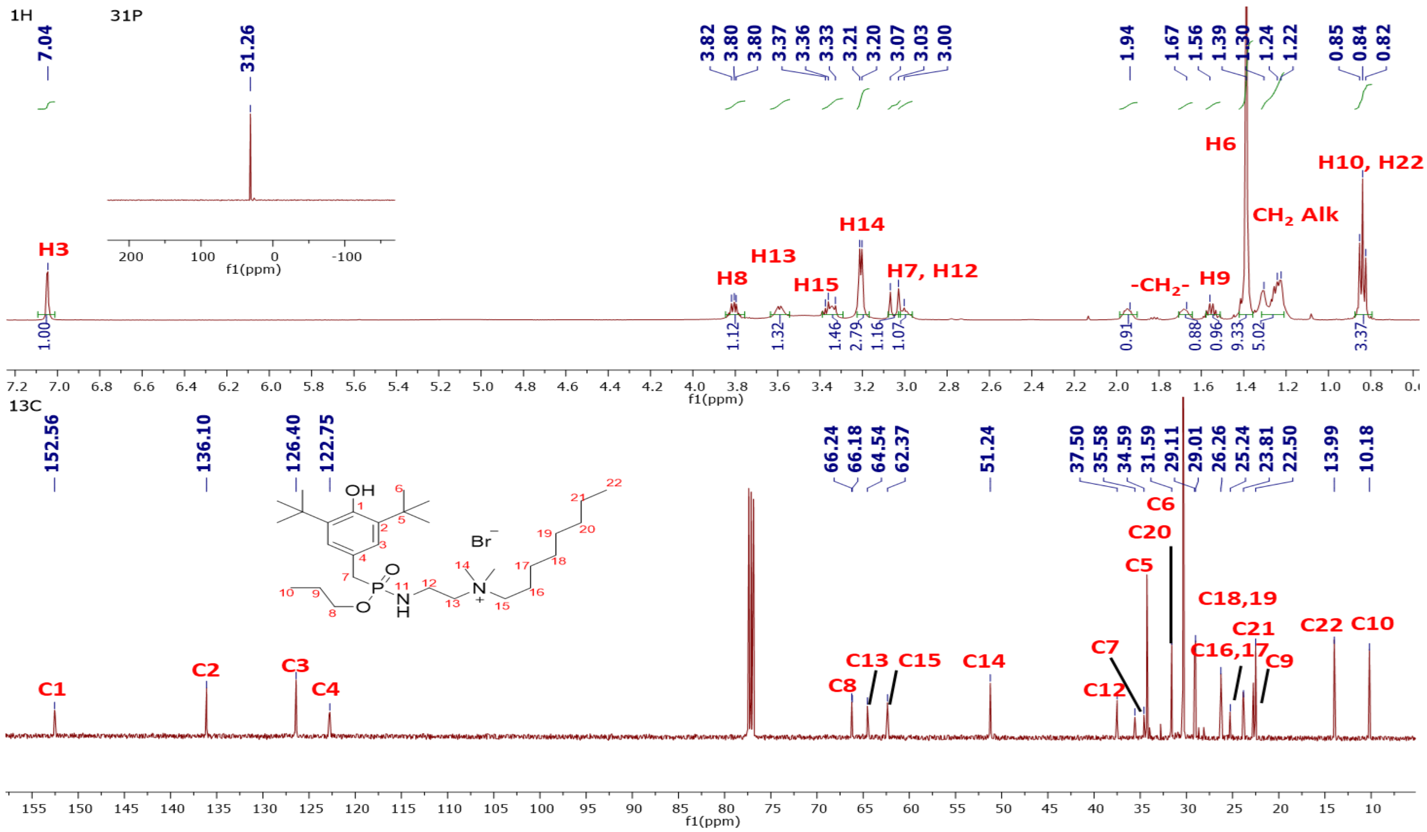

Figure S16. ^{1}H-, ^{13}C, ^{31}P- NMR of compound **6d**

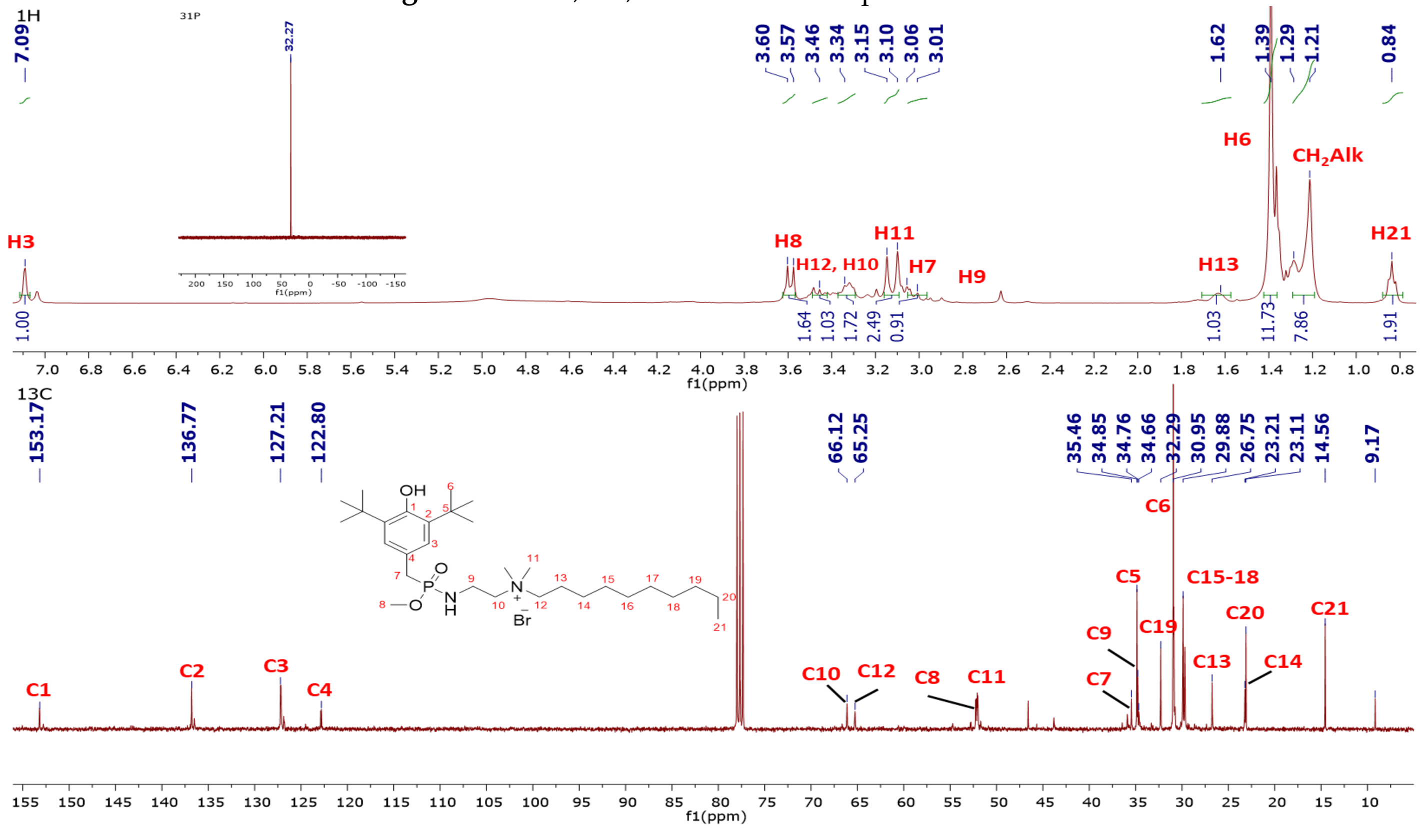

Figure S17. ^{1}H-, ^{13}C, ^{31}P- NMR of compound **7a**

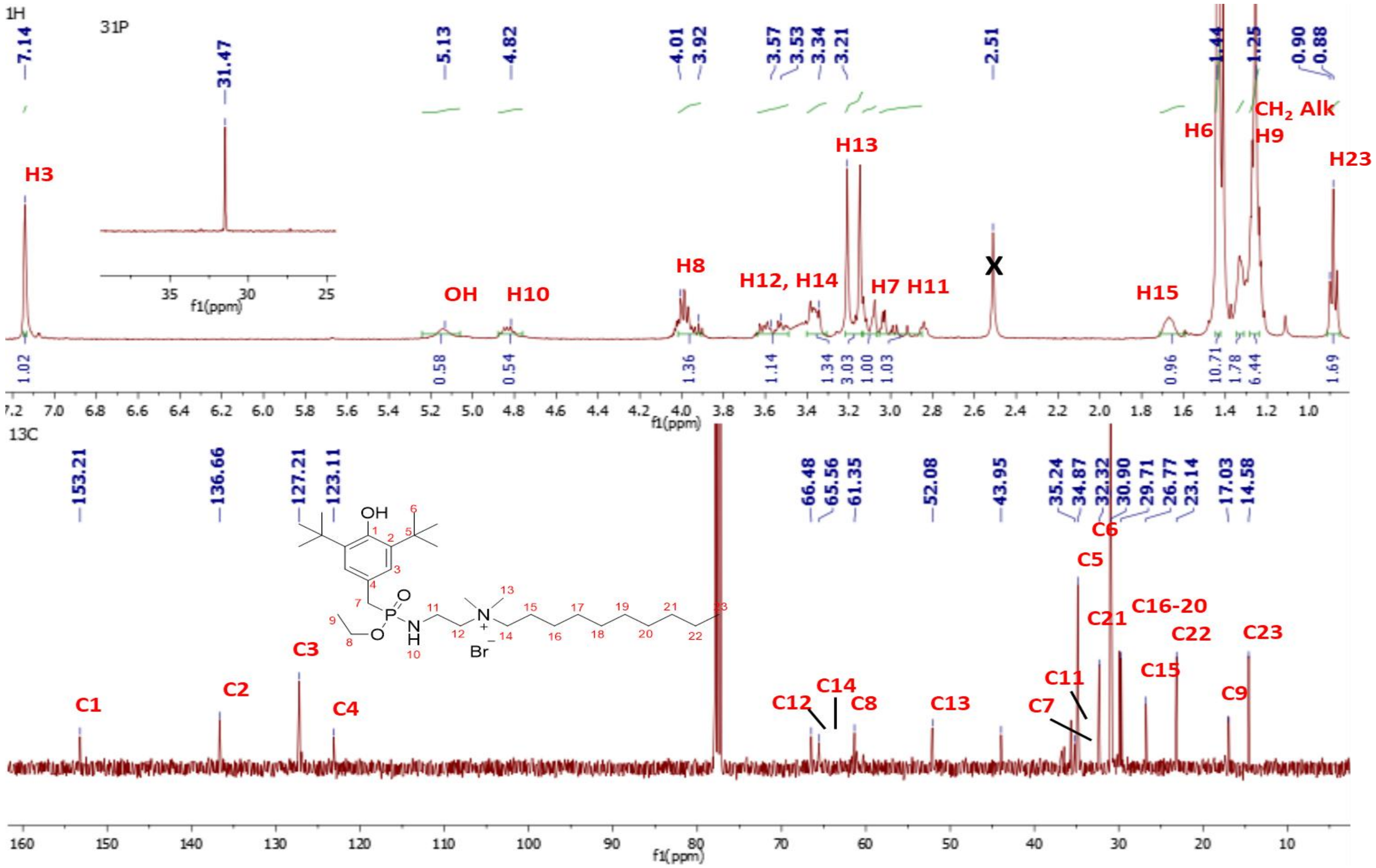

Figure S18. ^{1}H-, ^{13}C, ^{31}P- NMR of compound **7b**

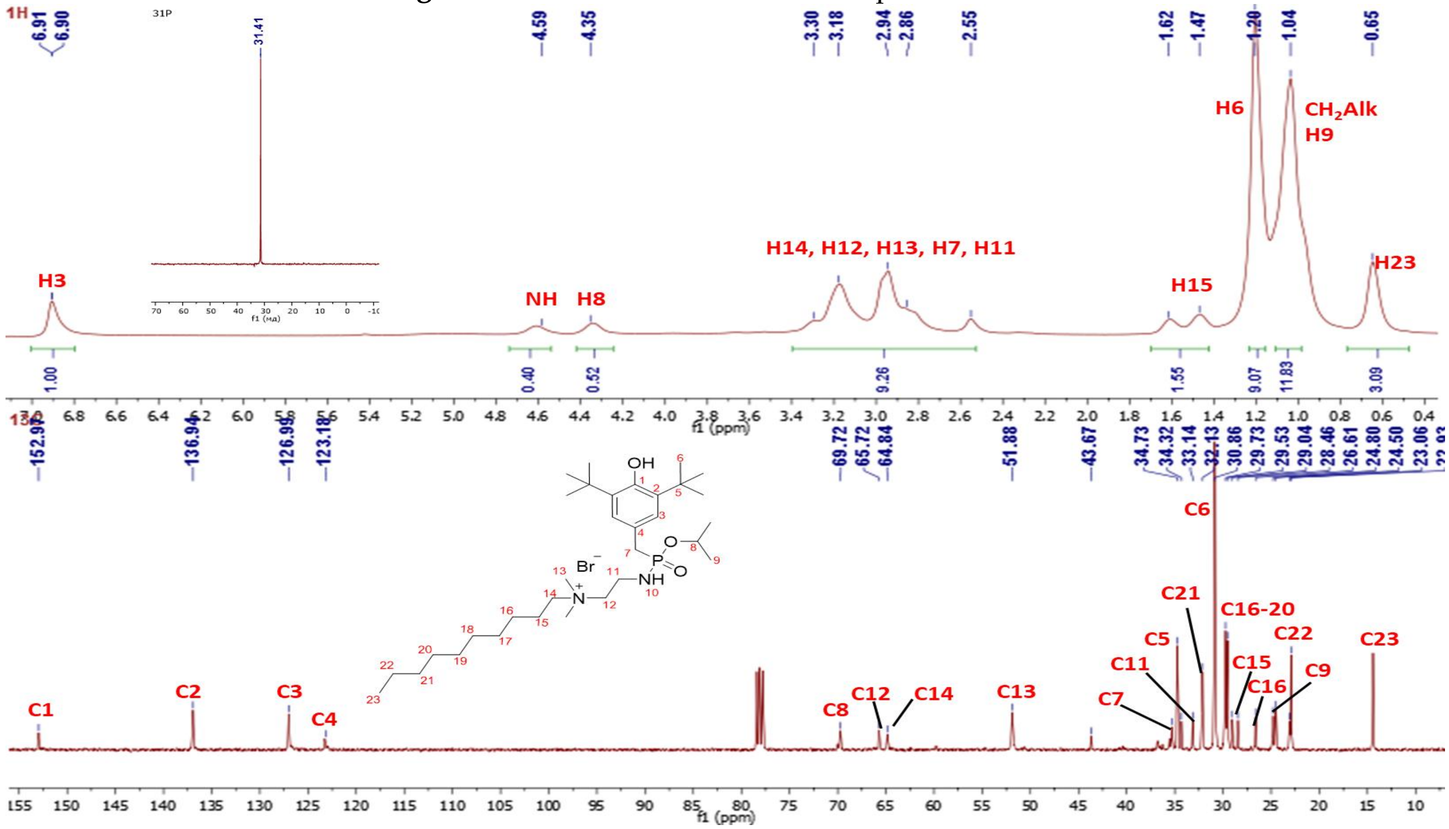

Figure S19. ^{1}H-, ^{13}C, ^{31}P- NMR of compound **7c**

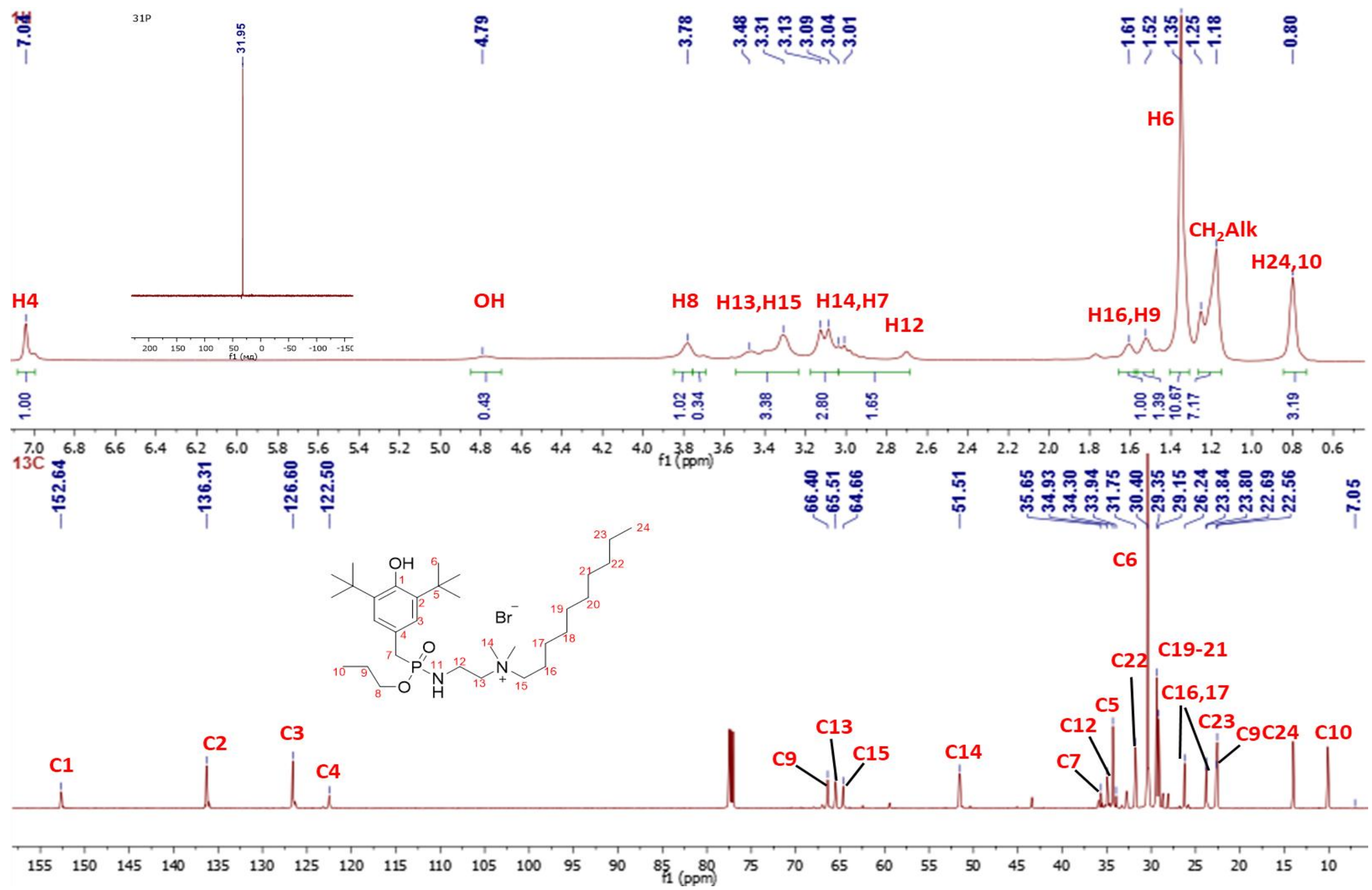

Figure S20. ^{1}H-, ^{13}C, ^{31}P- NMR of compound **7d**

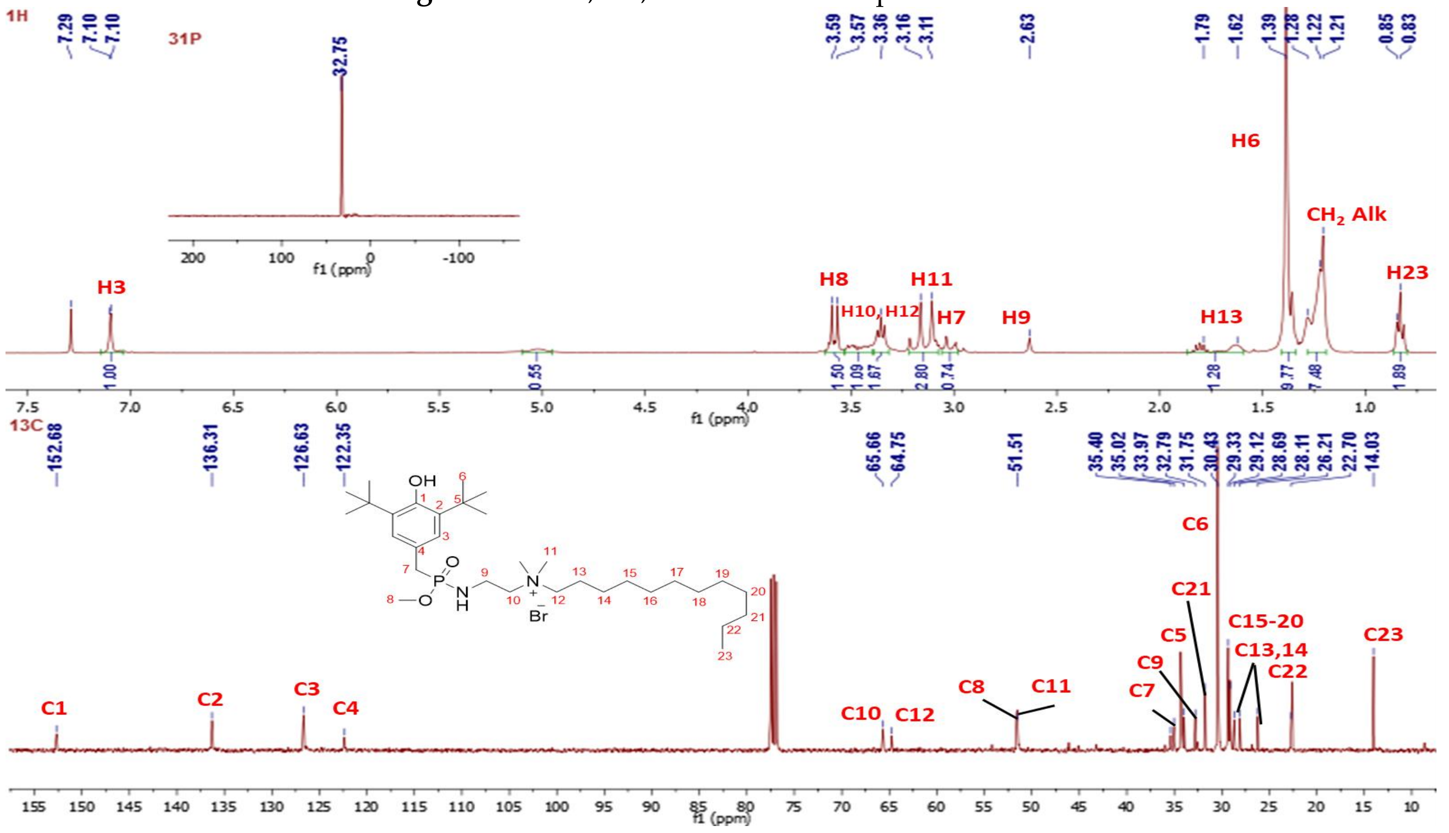

Figure S21. ^{1}H-, ^{13}C, ^{31}P- NMR of compound **8a**

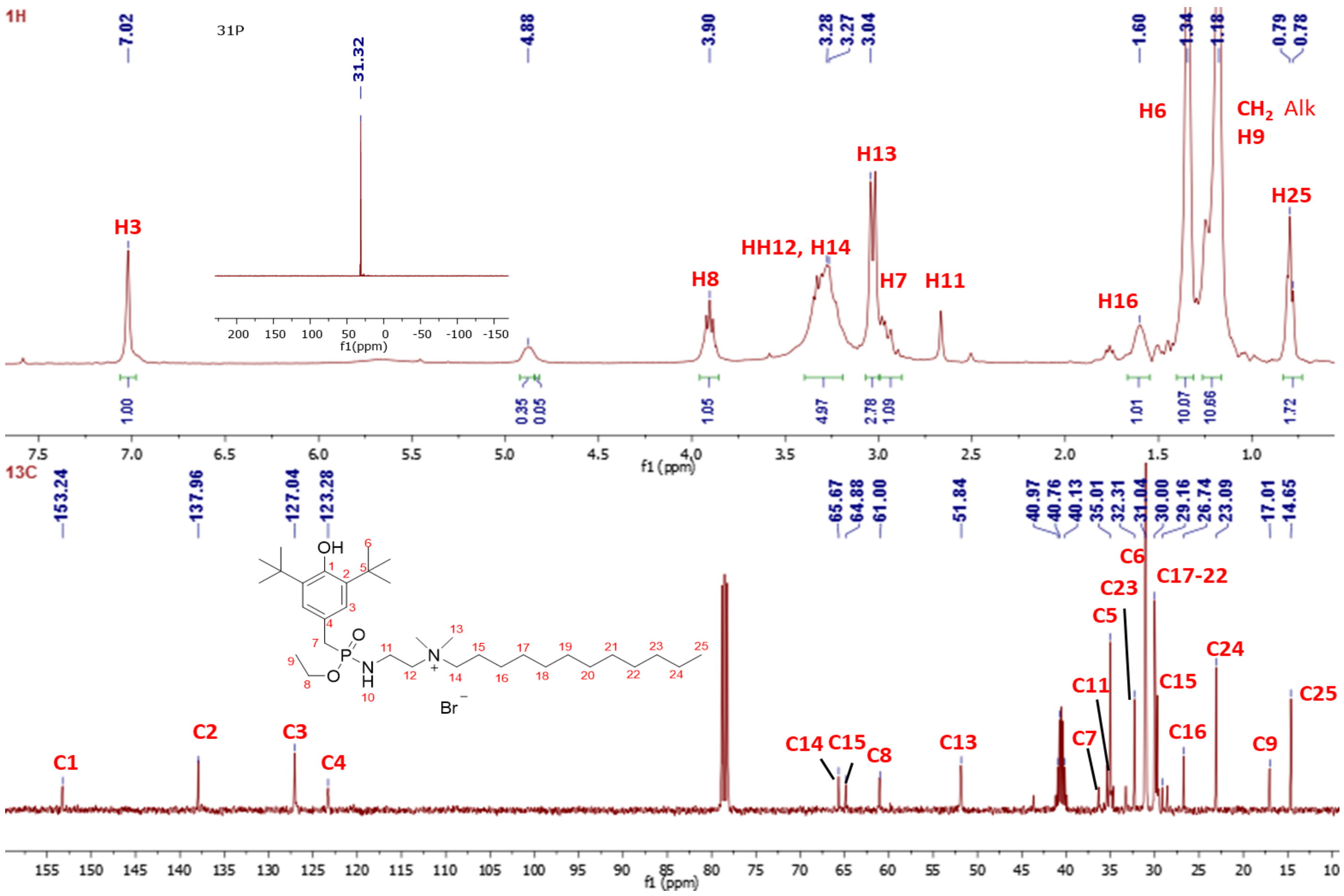

Figure S22. ^{1}H-, ^{13}C, ^{31}P- NMR of compound **8b**

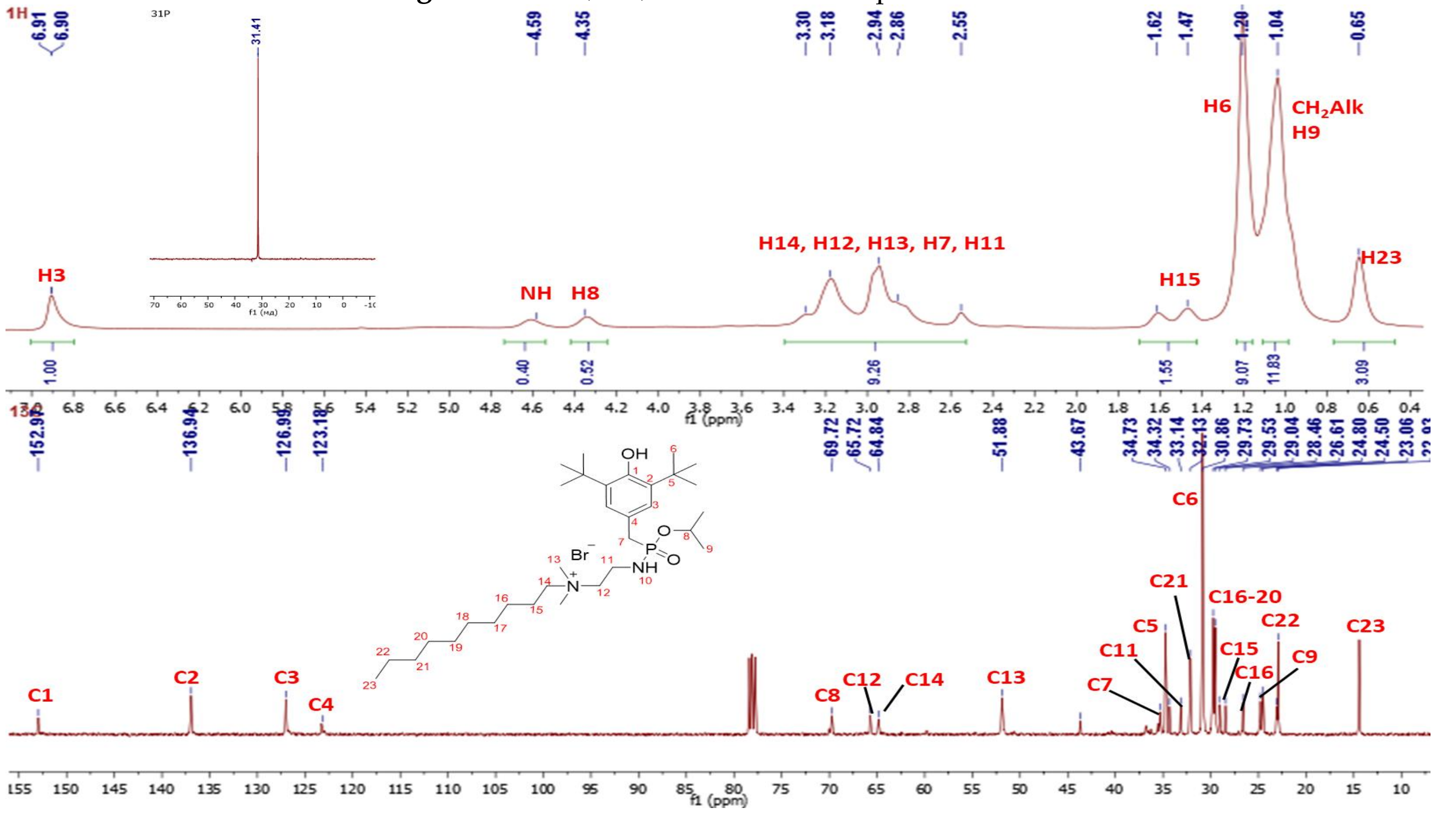

Figure S23. ^{1}H-, ^{13}C, ^{31}P- NMR of compound **8c**

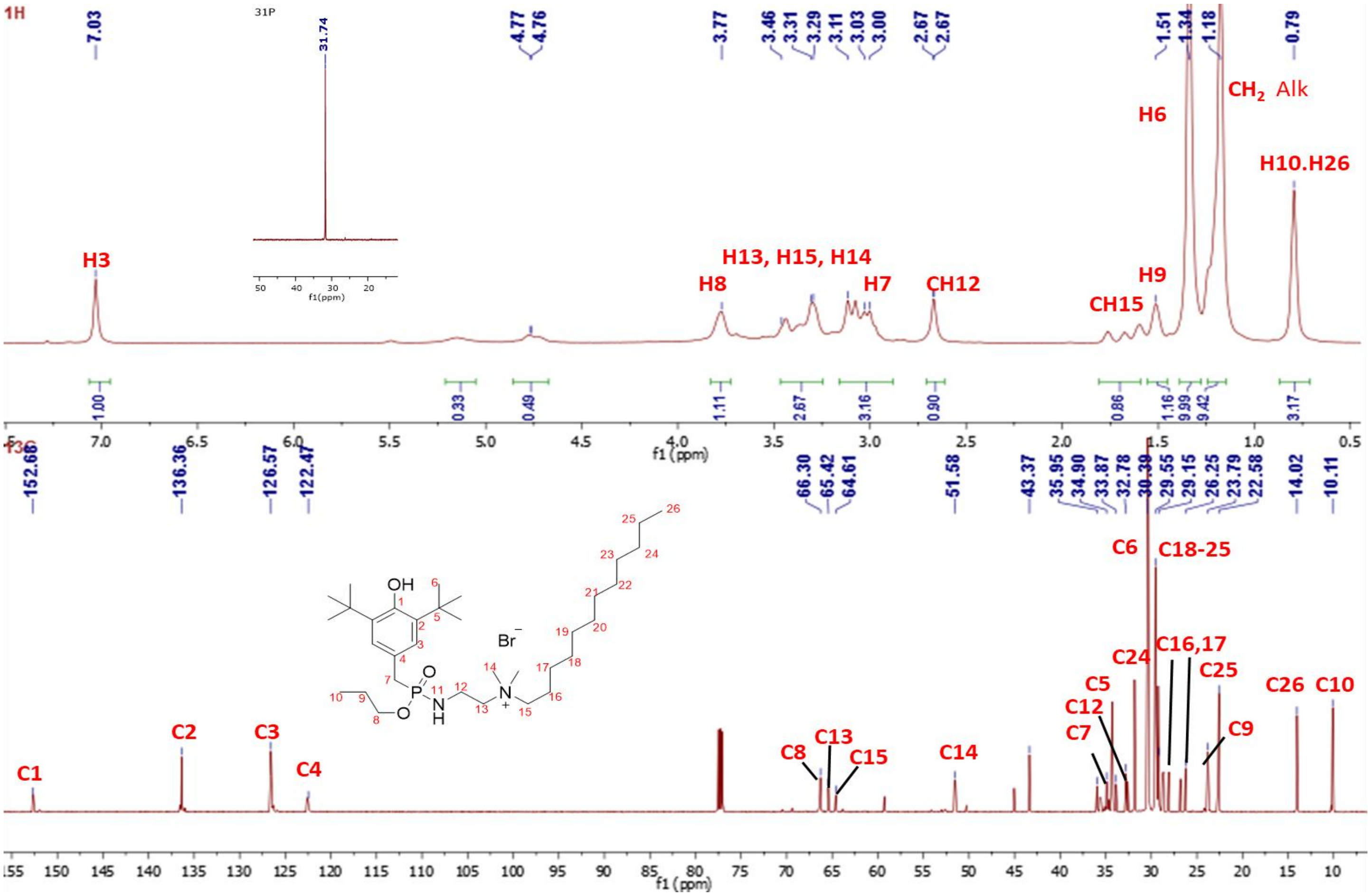

Figure S24. ^{1}H-, ^{13}C, ^{31}P- NMR of compound **8d**

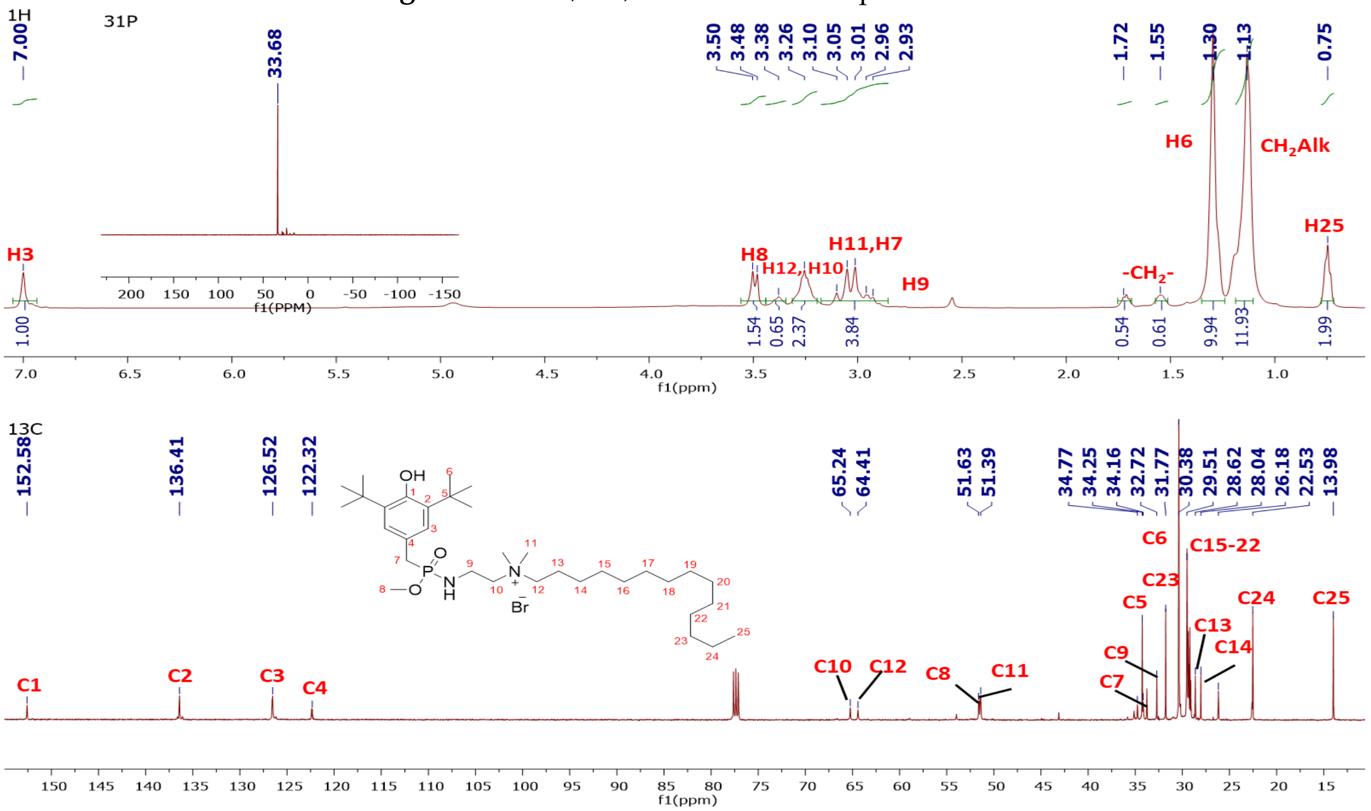

Figure S25. ^{1}H-, ^{13}C, ^{31}P- NMR of compound **9a**

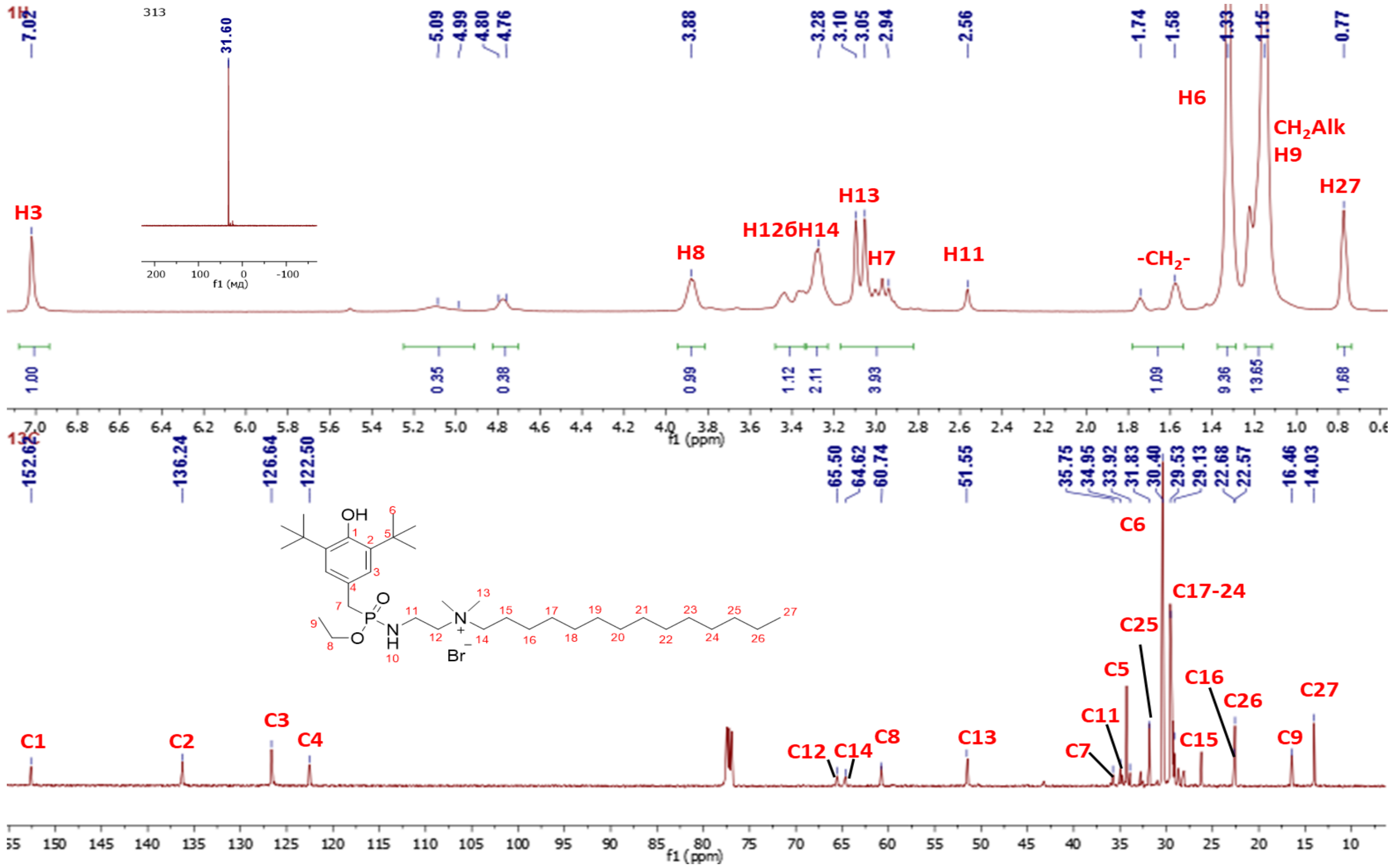

Figure S26. ^{1}H-, ^{13}C, ^{31}P- NMR of compound **9b**

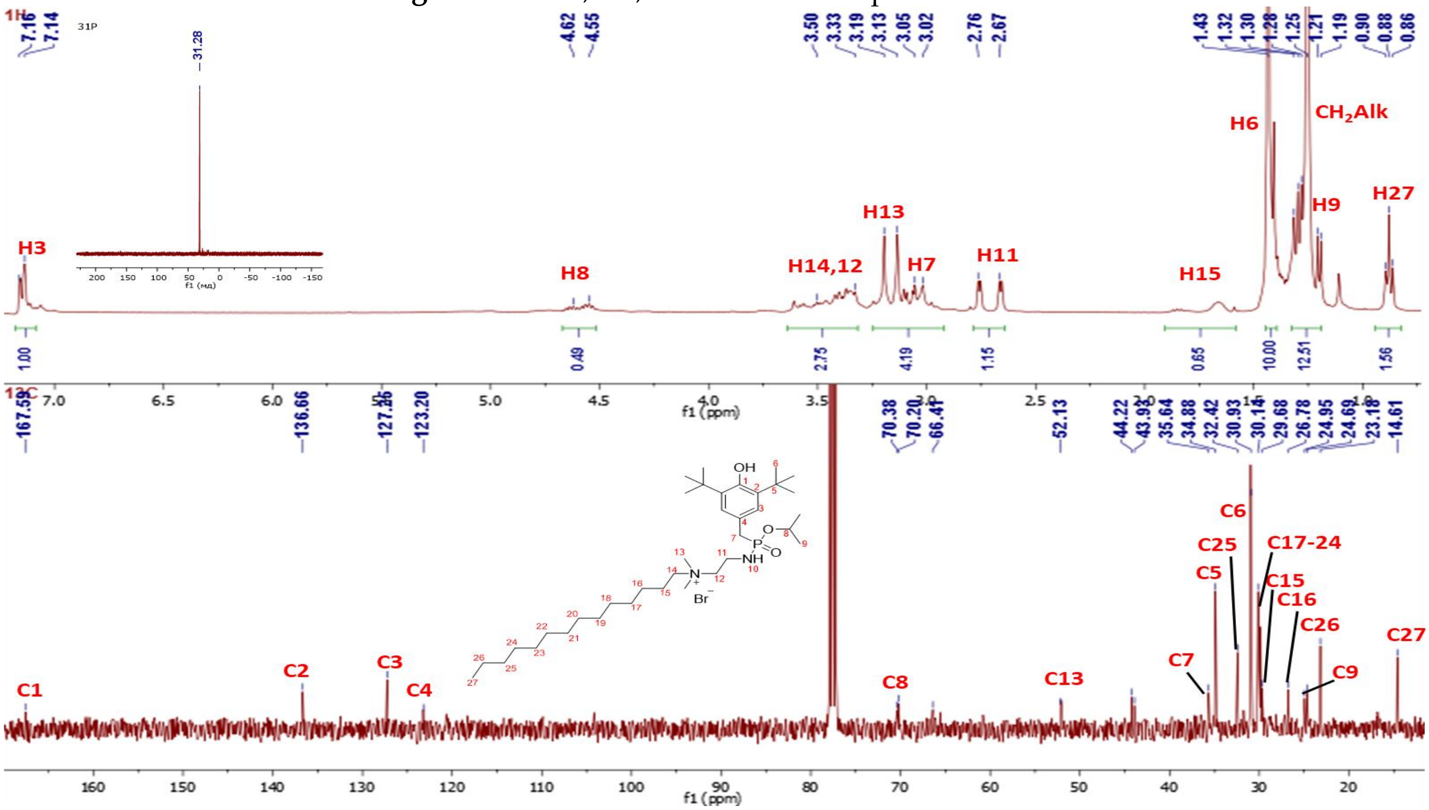

Figure S27. ^{1}H-, ^{13}C, ^{31}P- NMR of compound **9c**

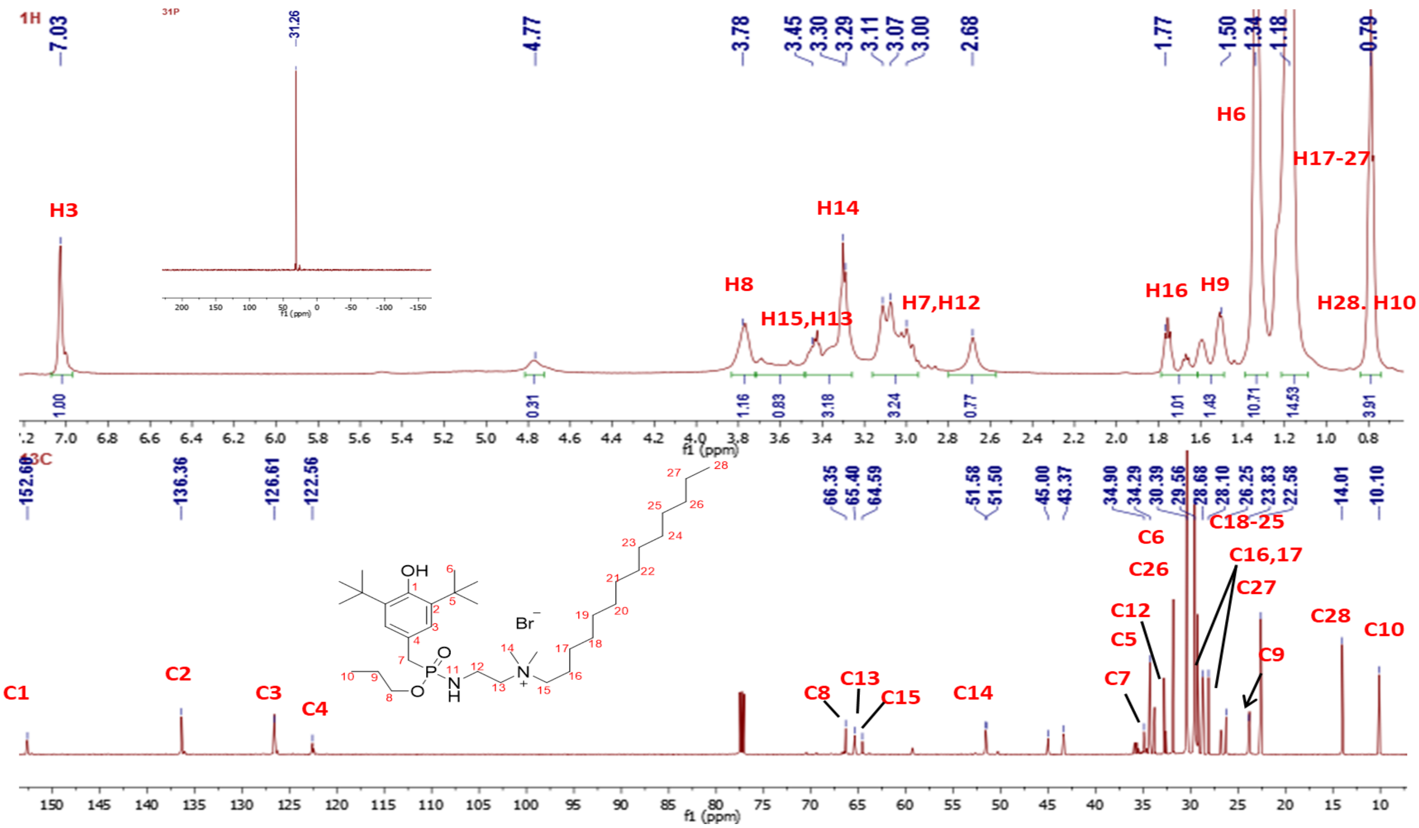

Figure S28. ^{1}H-, ^{13}C, ^{31}P- NMR of compound **9d**

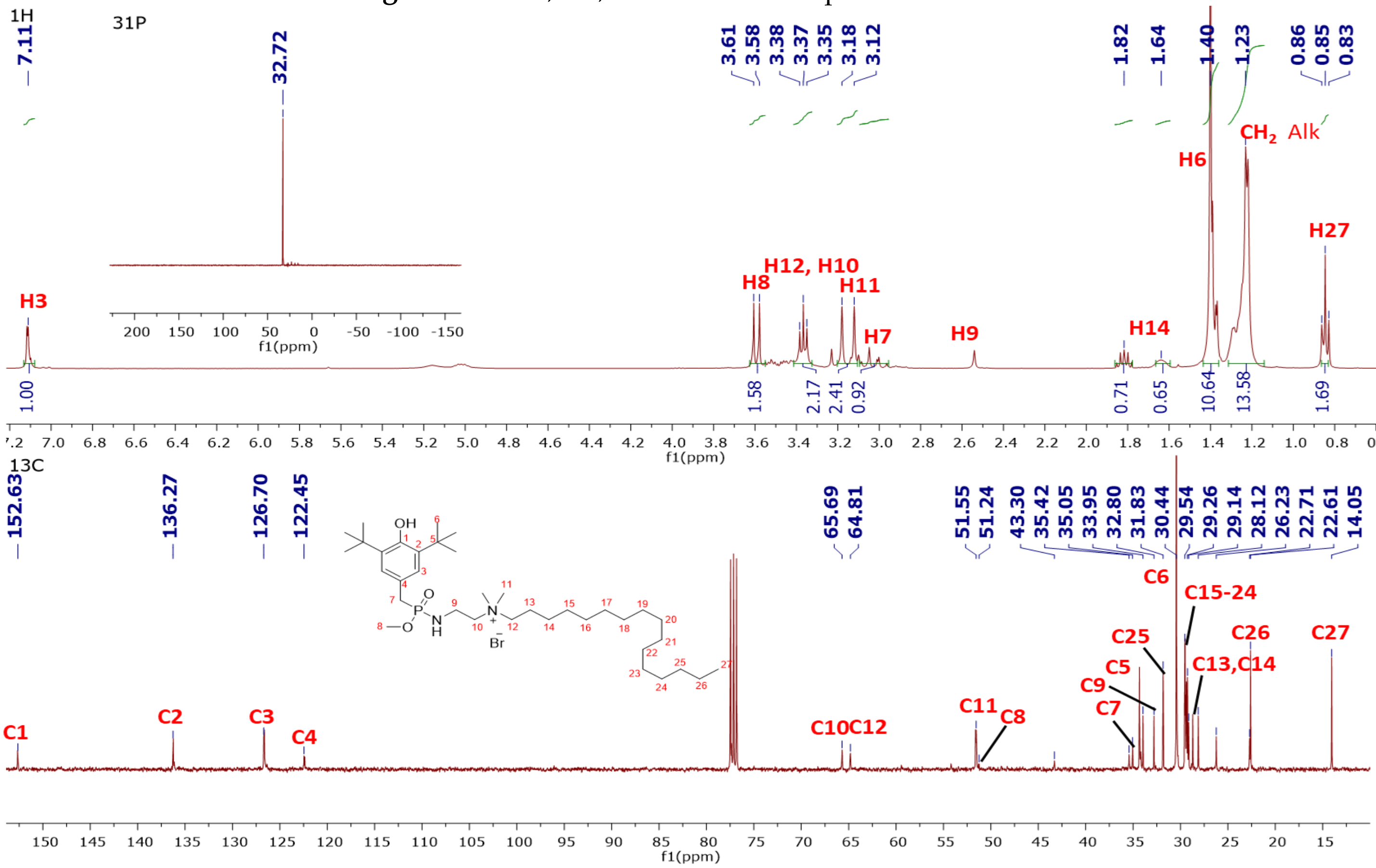

Figure S29. ^{1}H-, ^{13}C, ^{31}P- NMR of compound **10a**

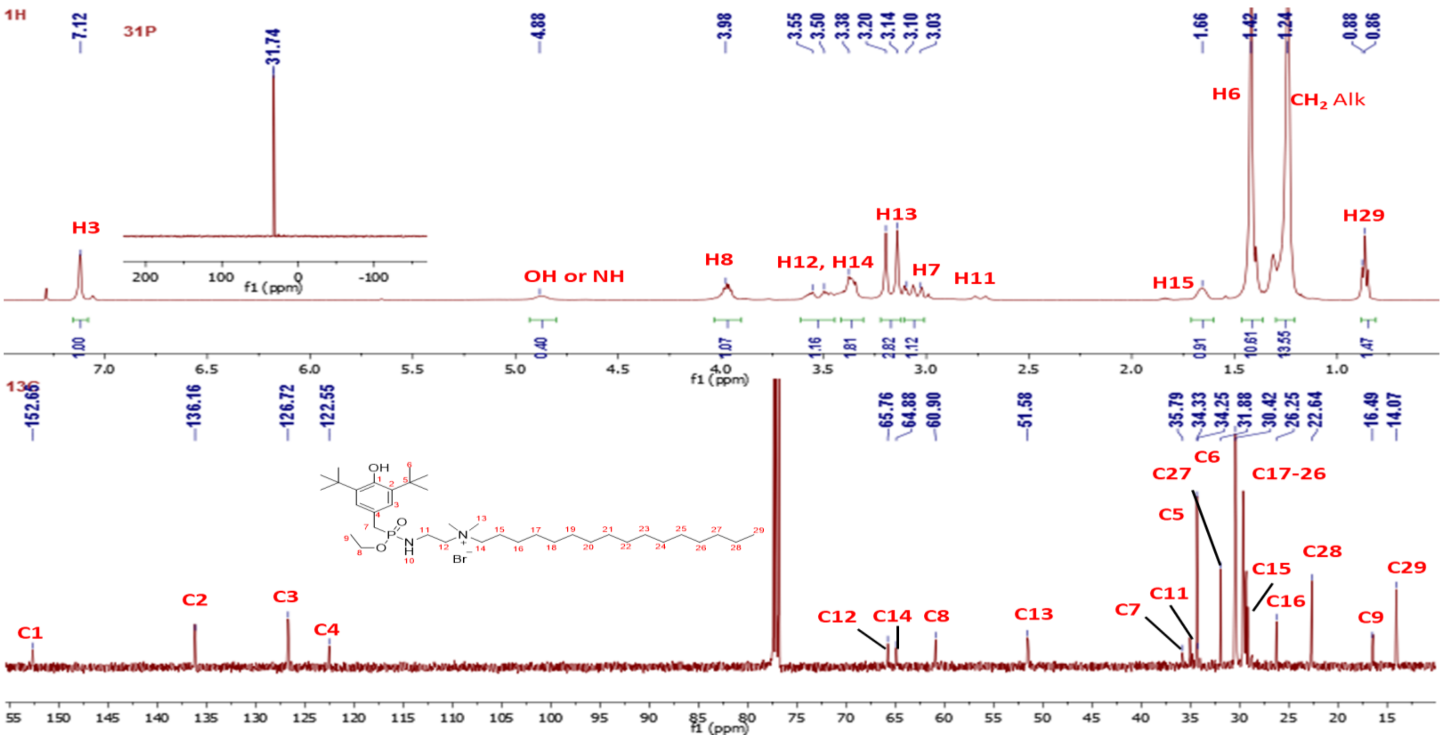

Figure S30. ^{1}H-, ^{13}C, ^{31}P- NMR of compound **10b**

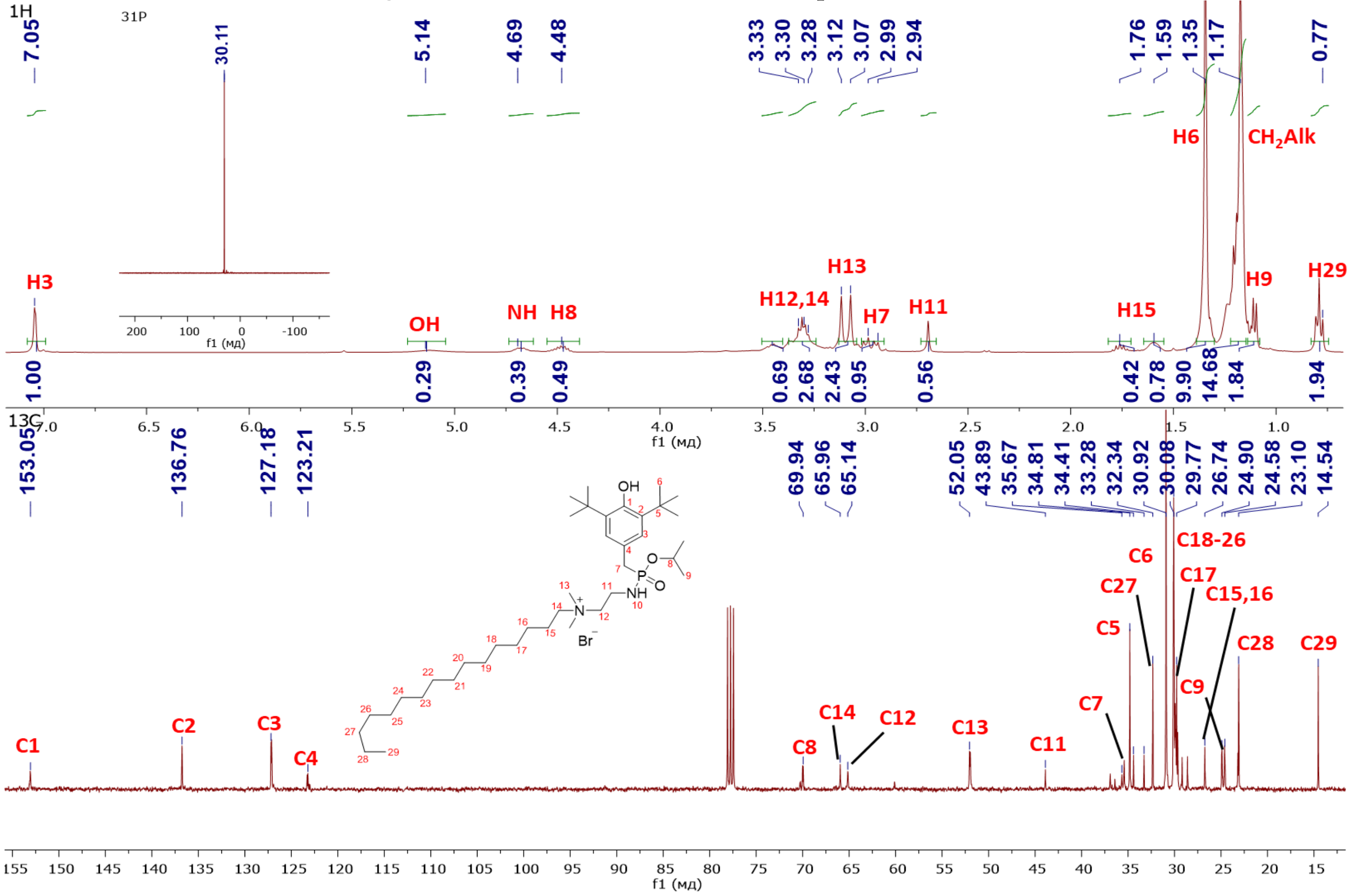

Figure S31. ^{1}H-, ^{13}C, ^{31}P- NMR of compound **10c**

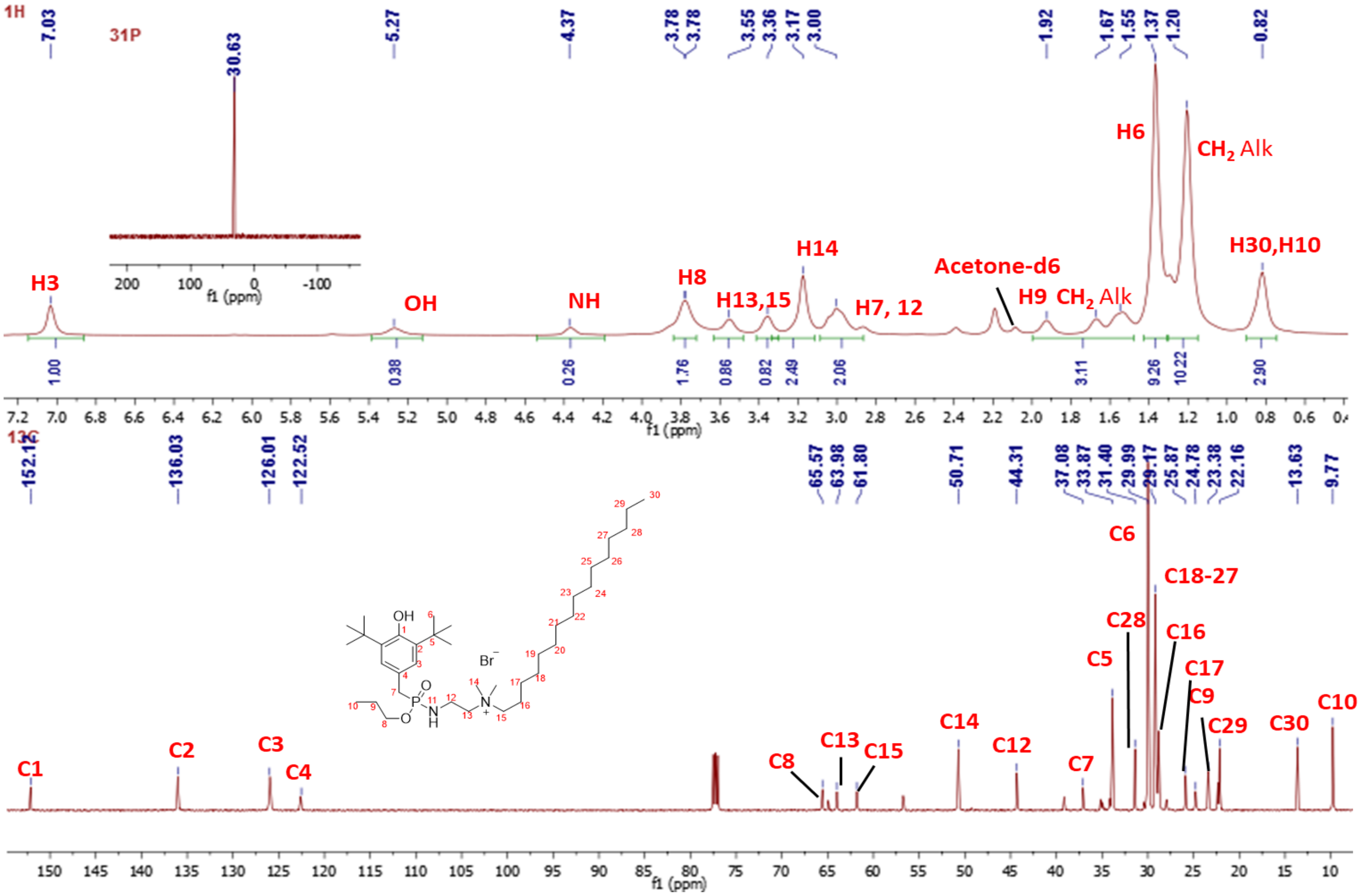

Figure S32. ^{1}H-, ^{13}C, ^{31}P- NMR of compound **10d**

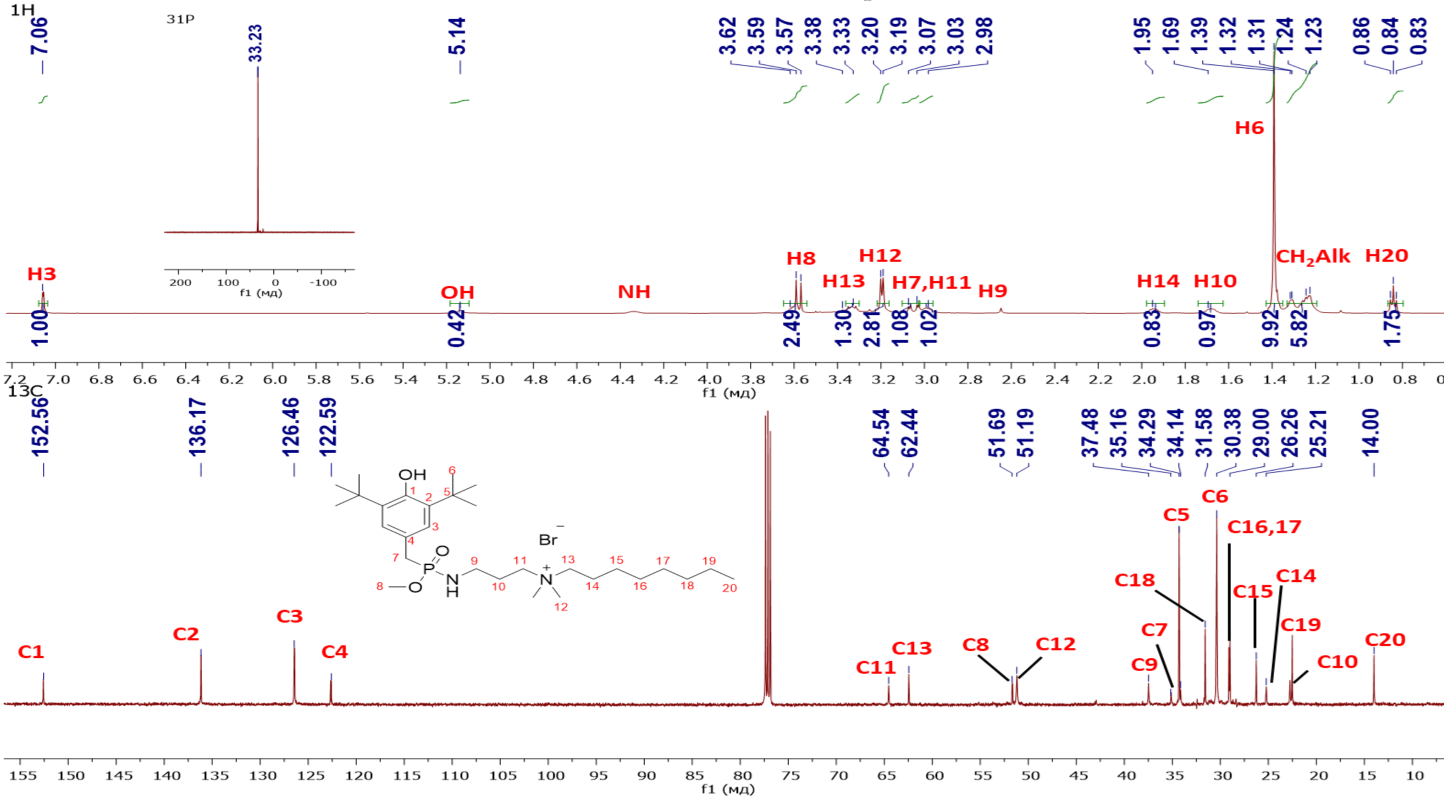

Figure S33. ^{1}H-, ^{13}C, ^{31}P- NMR of compound **11a**

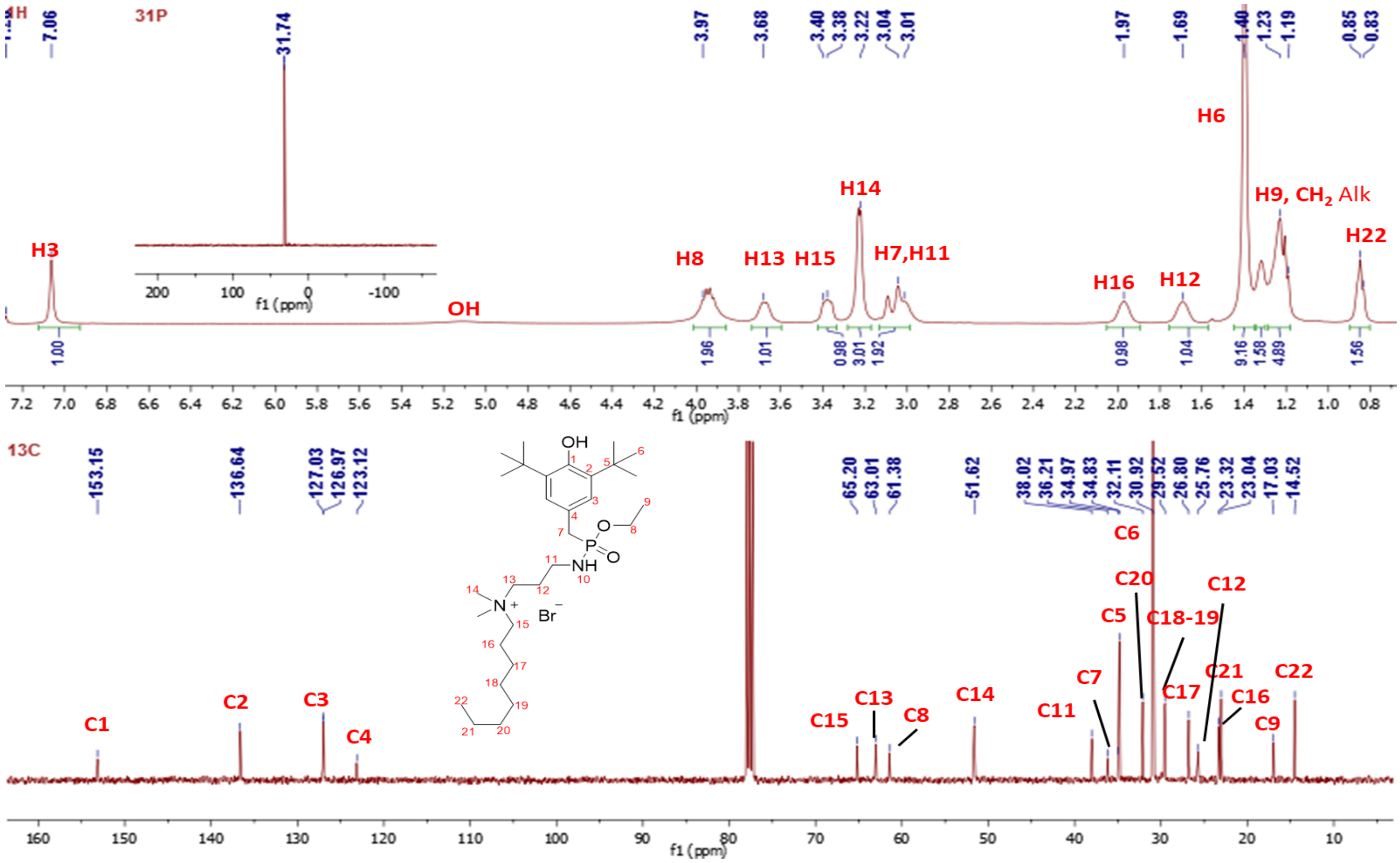

Figure S34. 1H-, 13C, 31P- NMR of compound **11b**

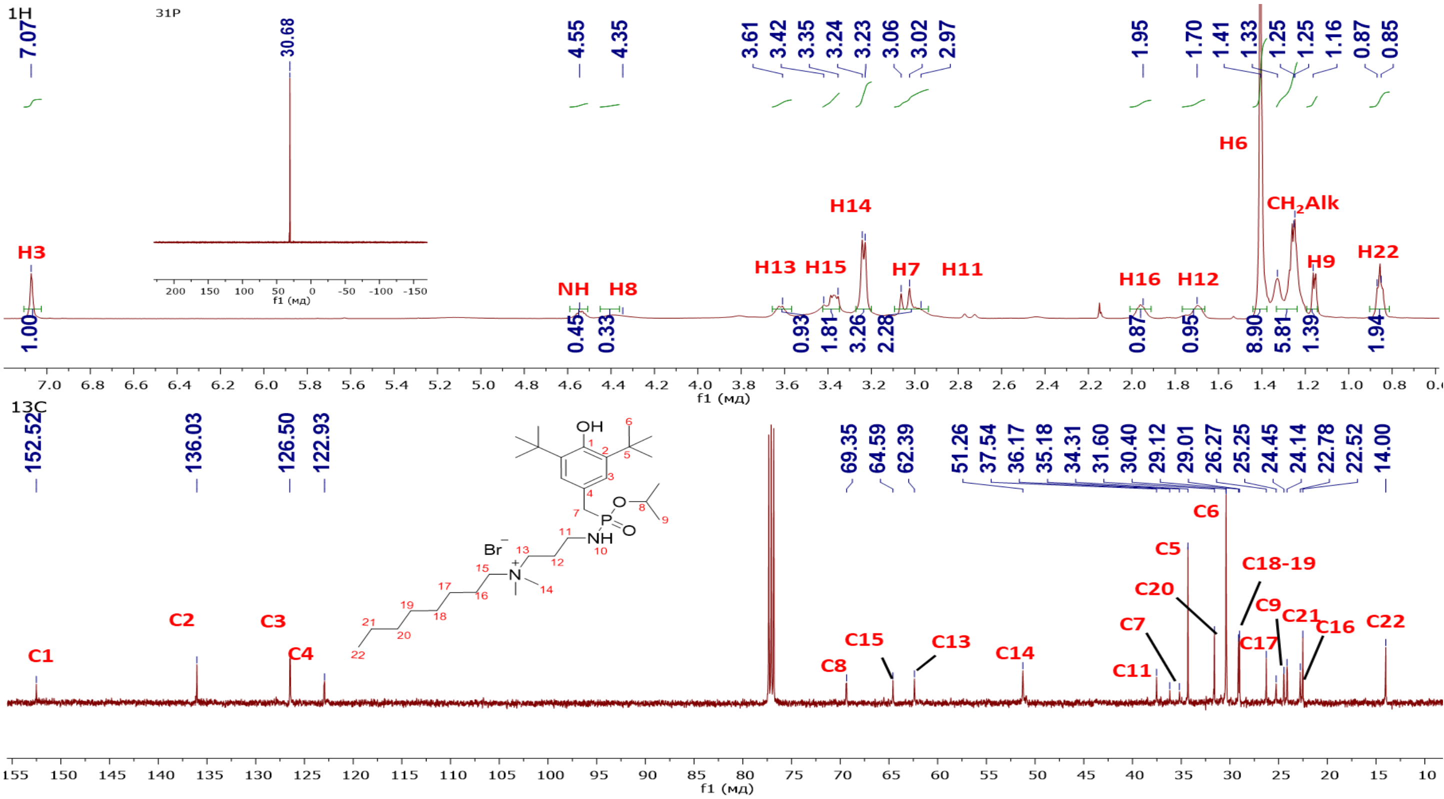

Figure S35. 1H-, 13C, 31P- NMR of compound **11c**

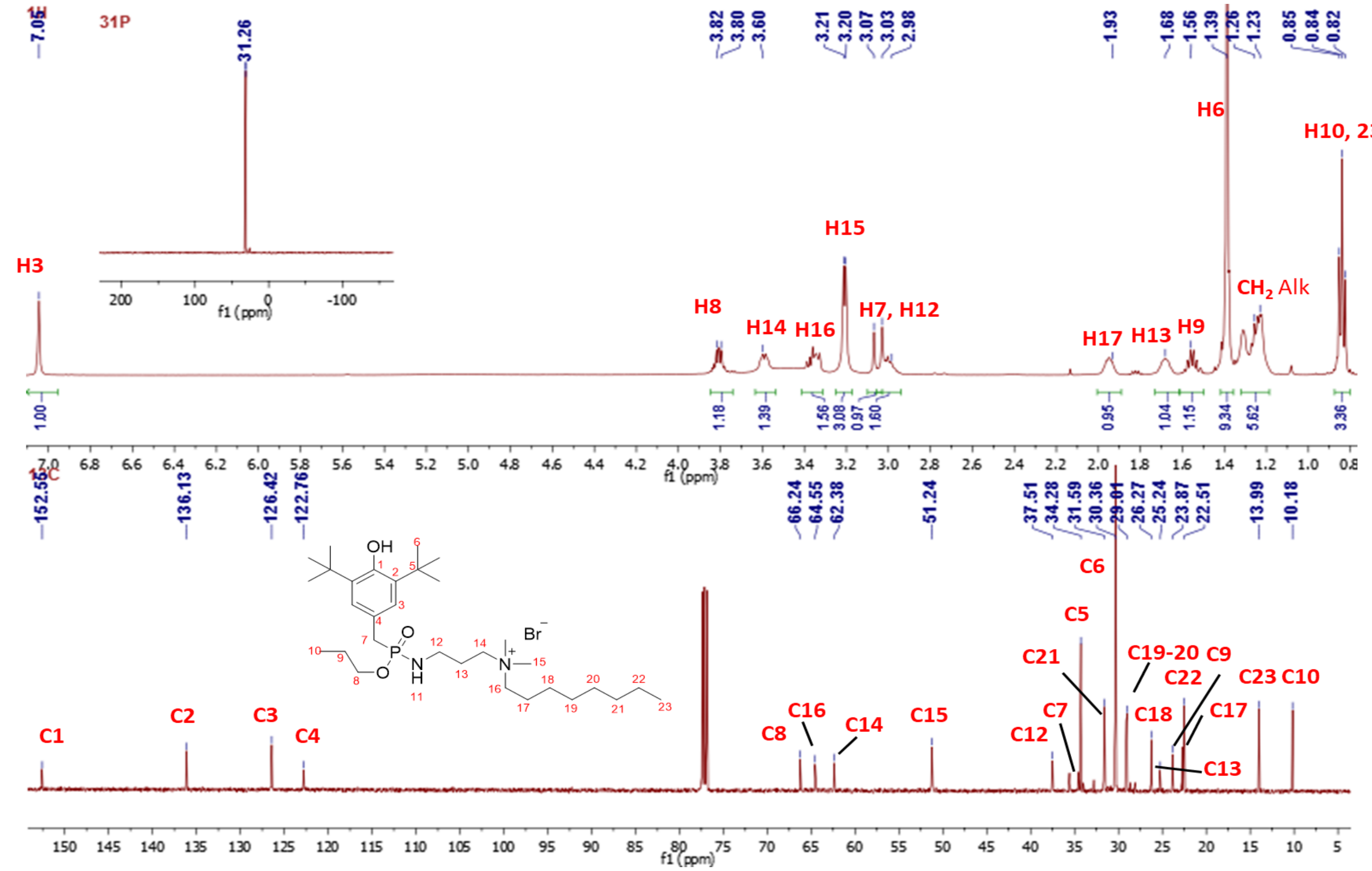

Figure S36. ¹H-, ¹³C, ³¹P- NMR of compound **11d**

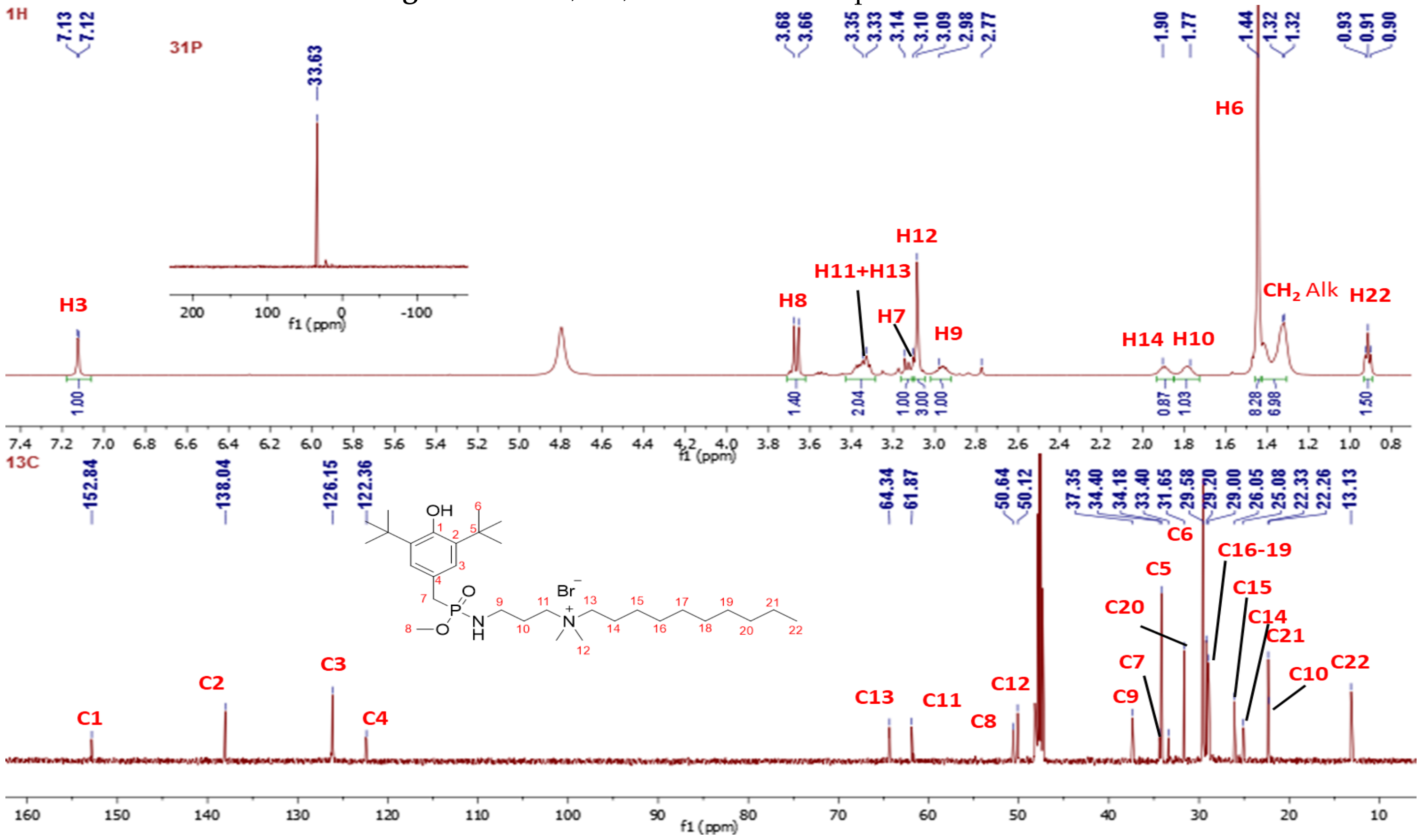

Figure S37. ¹H-, ¹³C, ³¹P- NMR of compound **12a**

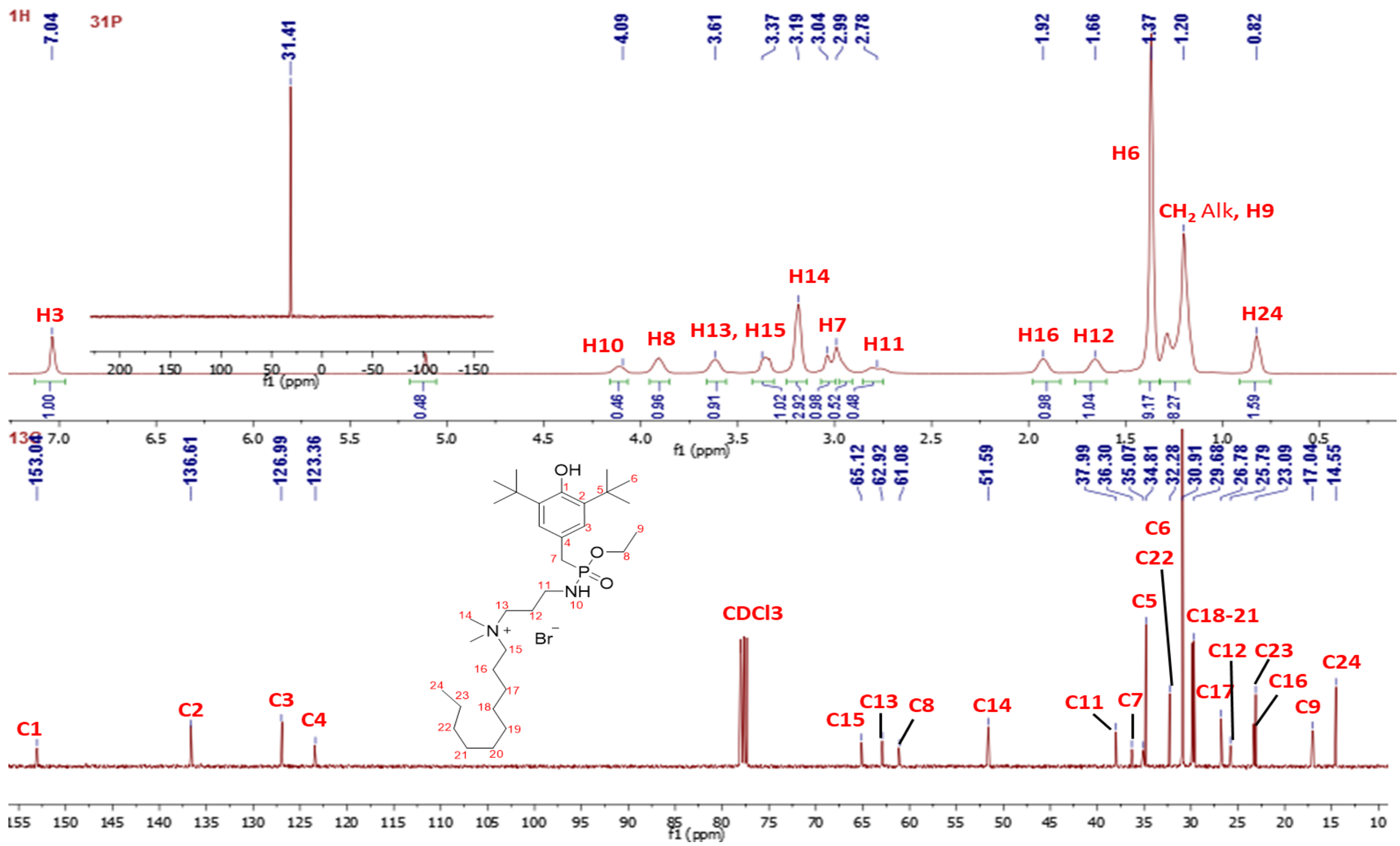

Figure S38. ^{1}H-, ^{13}C, ^{31}P- NMR of compound **12b**

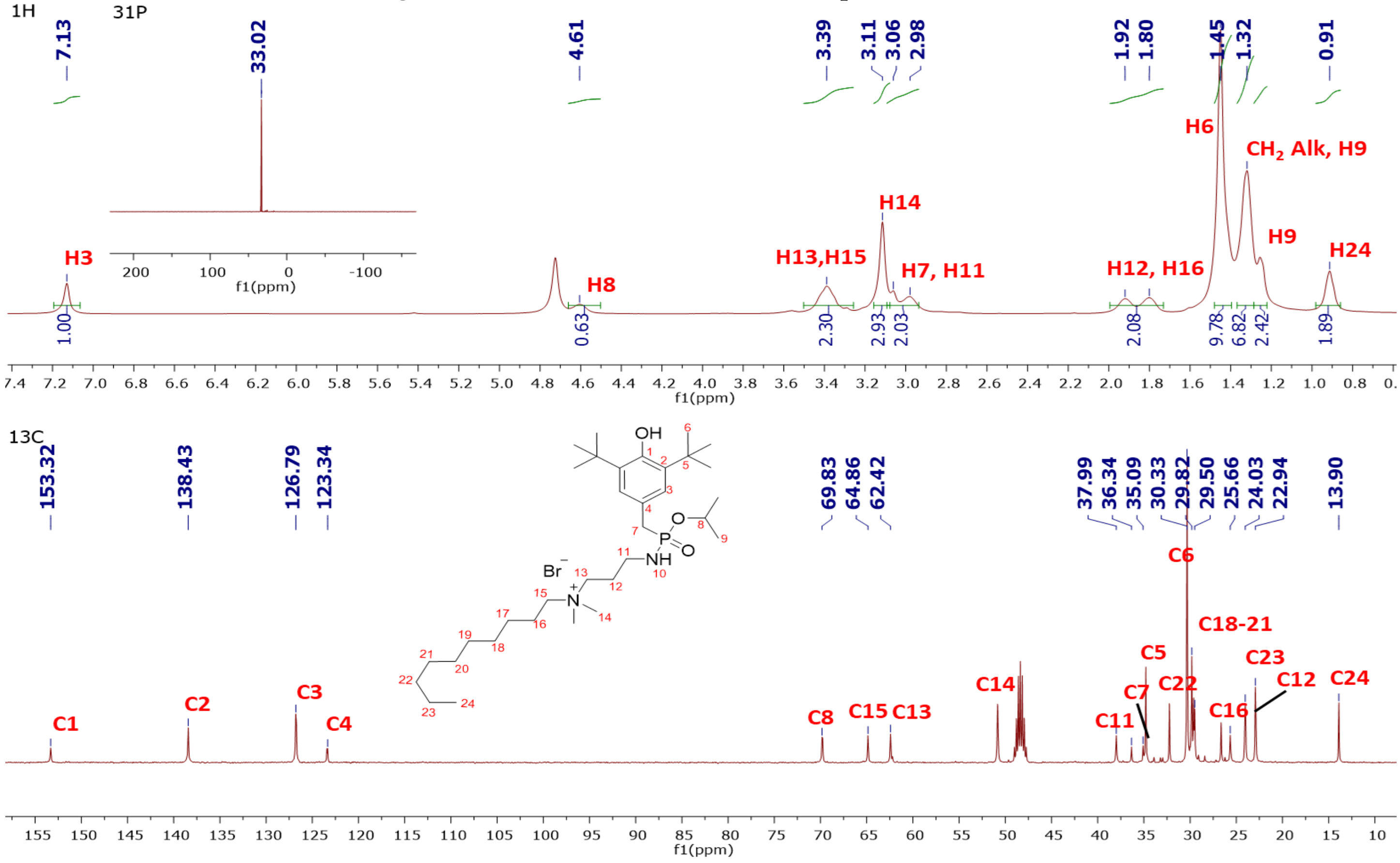

Figure S39. ^{1}H-, ^{13}C, ^{31}P- NMR of compound **12c**

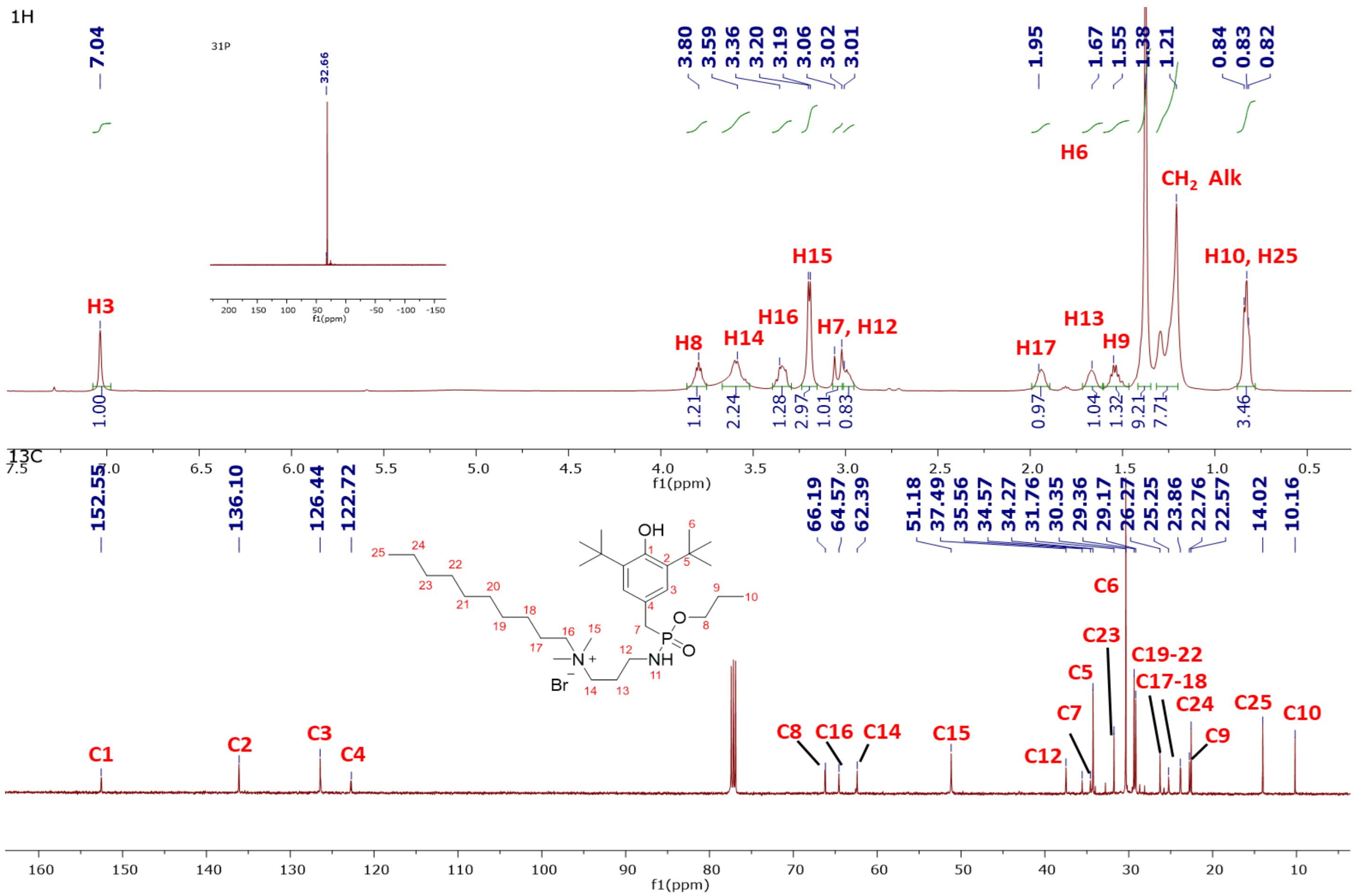

Figure S40. ^{1}H-, ^{13}C, ^{31}P- NMR of compound **12d**

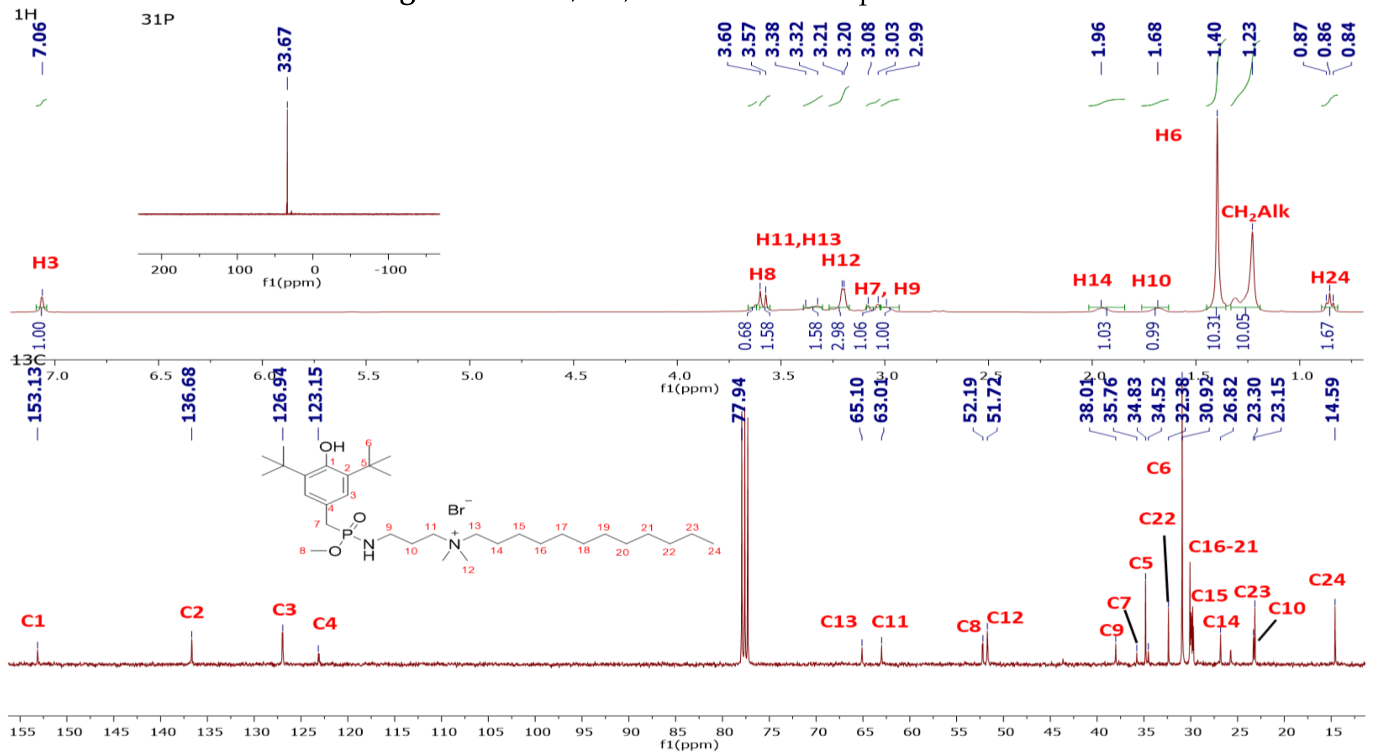

Figure S41. ^{1}H-, ^{13}C, ^{31}P- NMR of compound **13a**

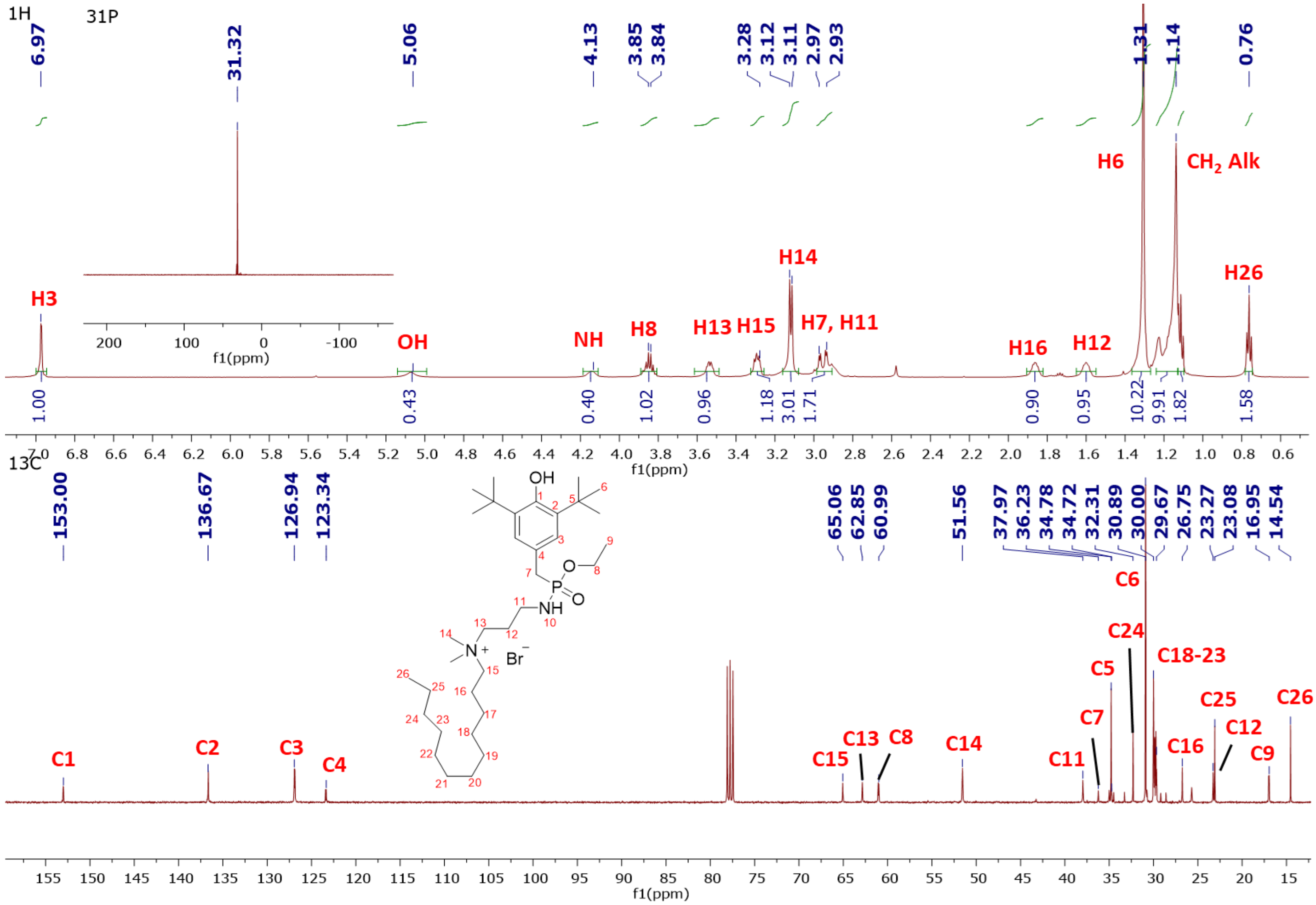

Figure S42. ^{1}H-, ^{13}C, ^{31}P- NMR of compound **13b**

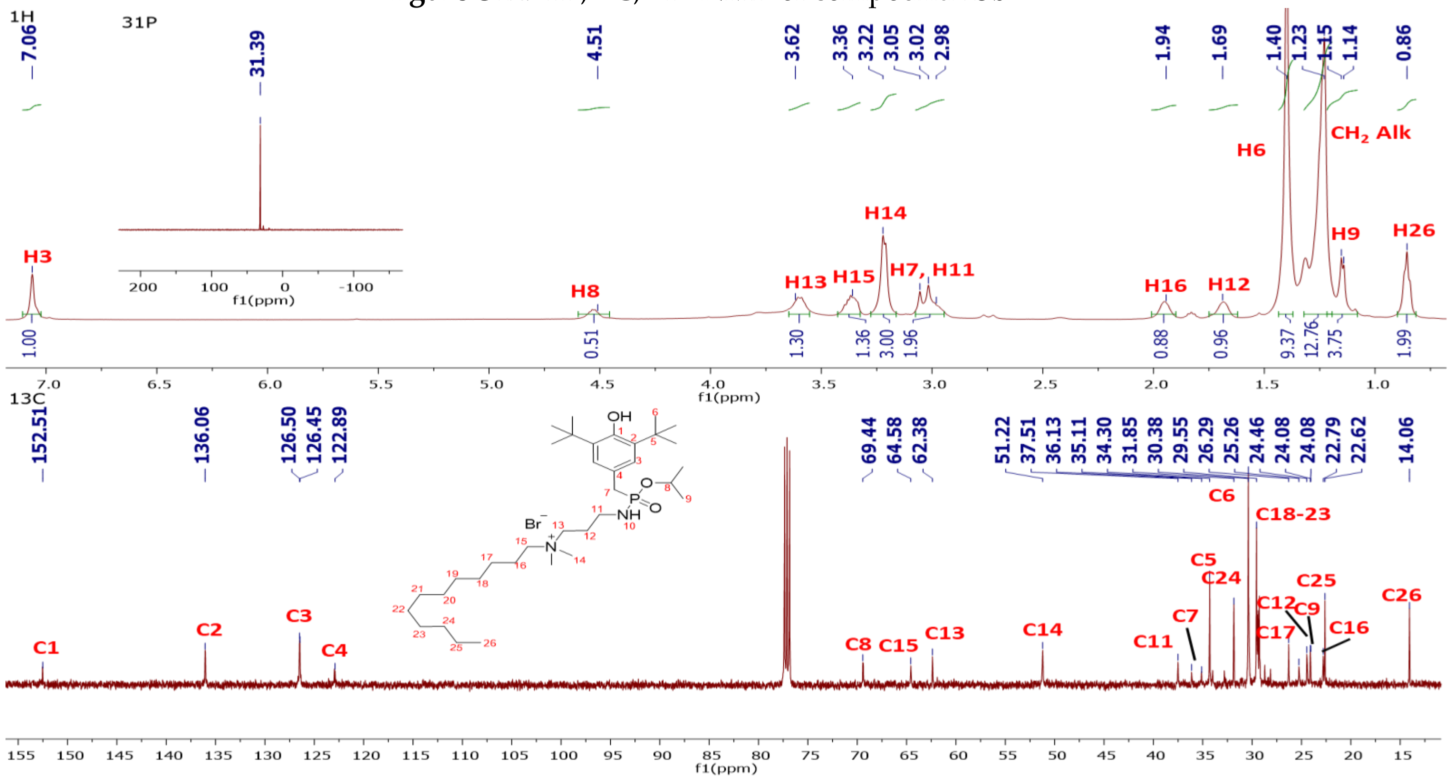

Figure S43. ^{1}H-, ^{13}C, ^{31}P- NMR of compound **13c**

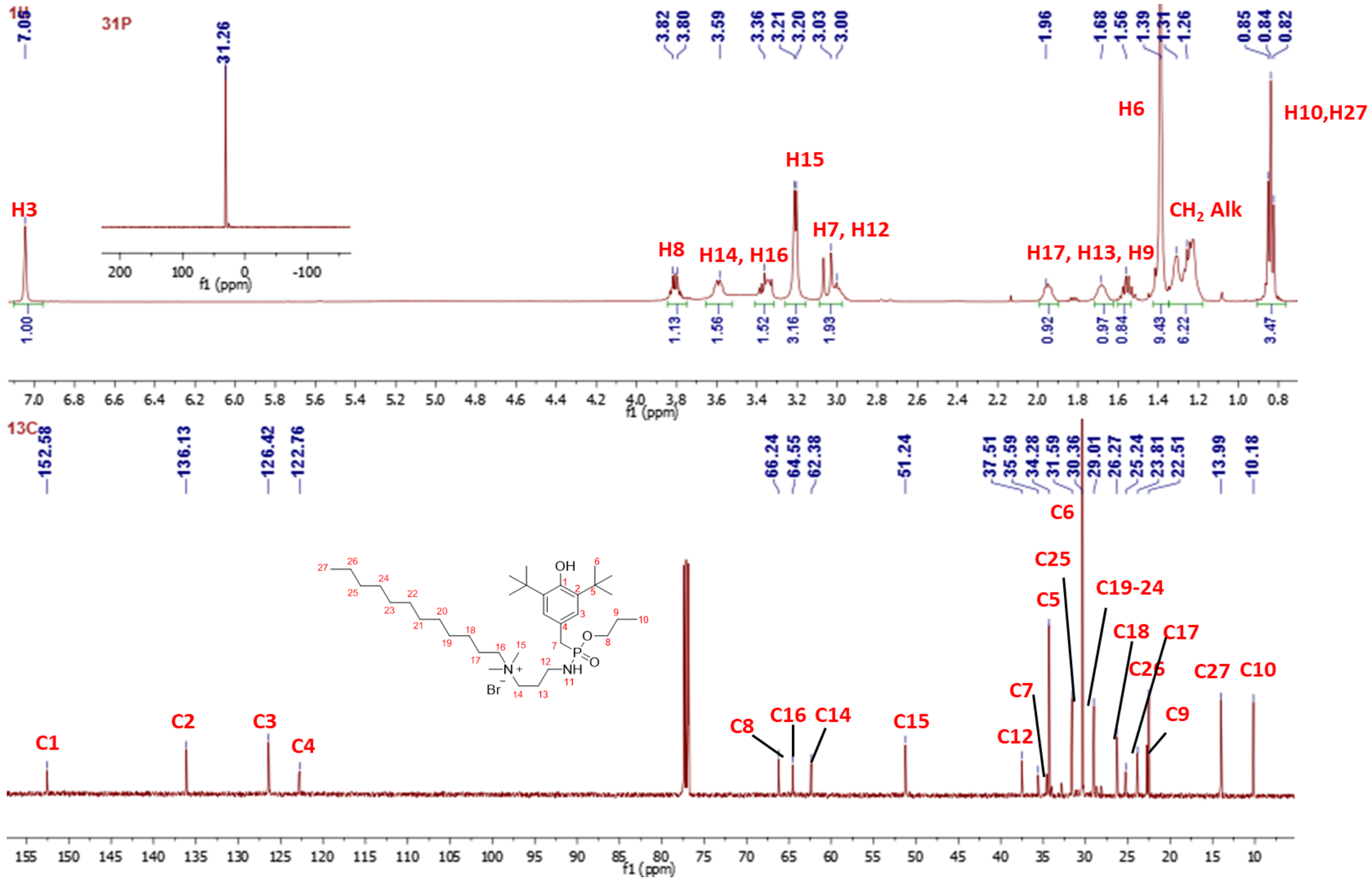

Figure S44. ^{1}H-, ^{13}C, ^{31}P- NMR of compound **13d**

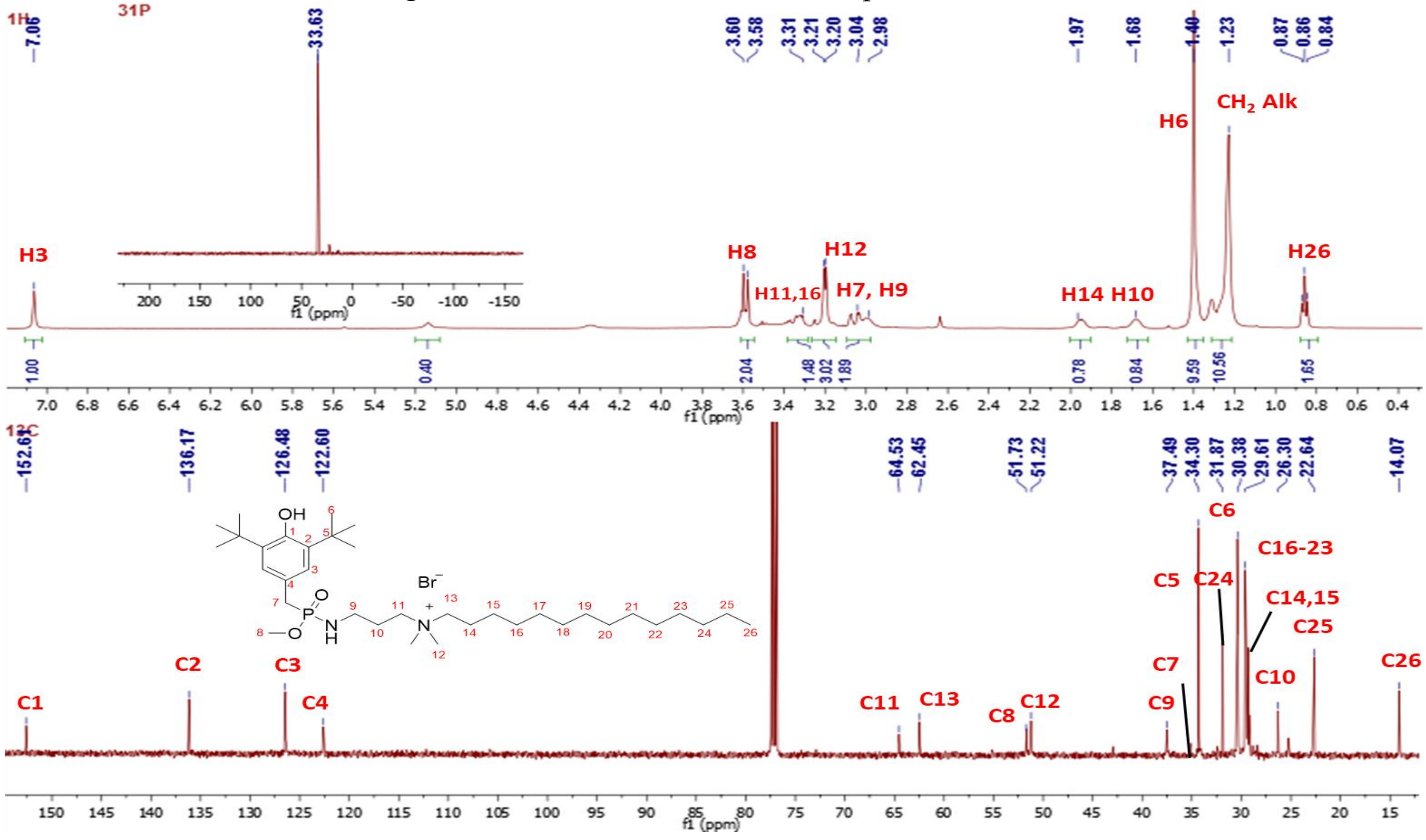

Figure S45. ^{1}H-, ^{13}C, ^{31}P- NMR of compound **14a**

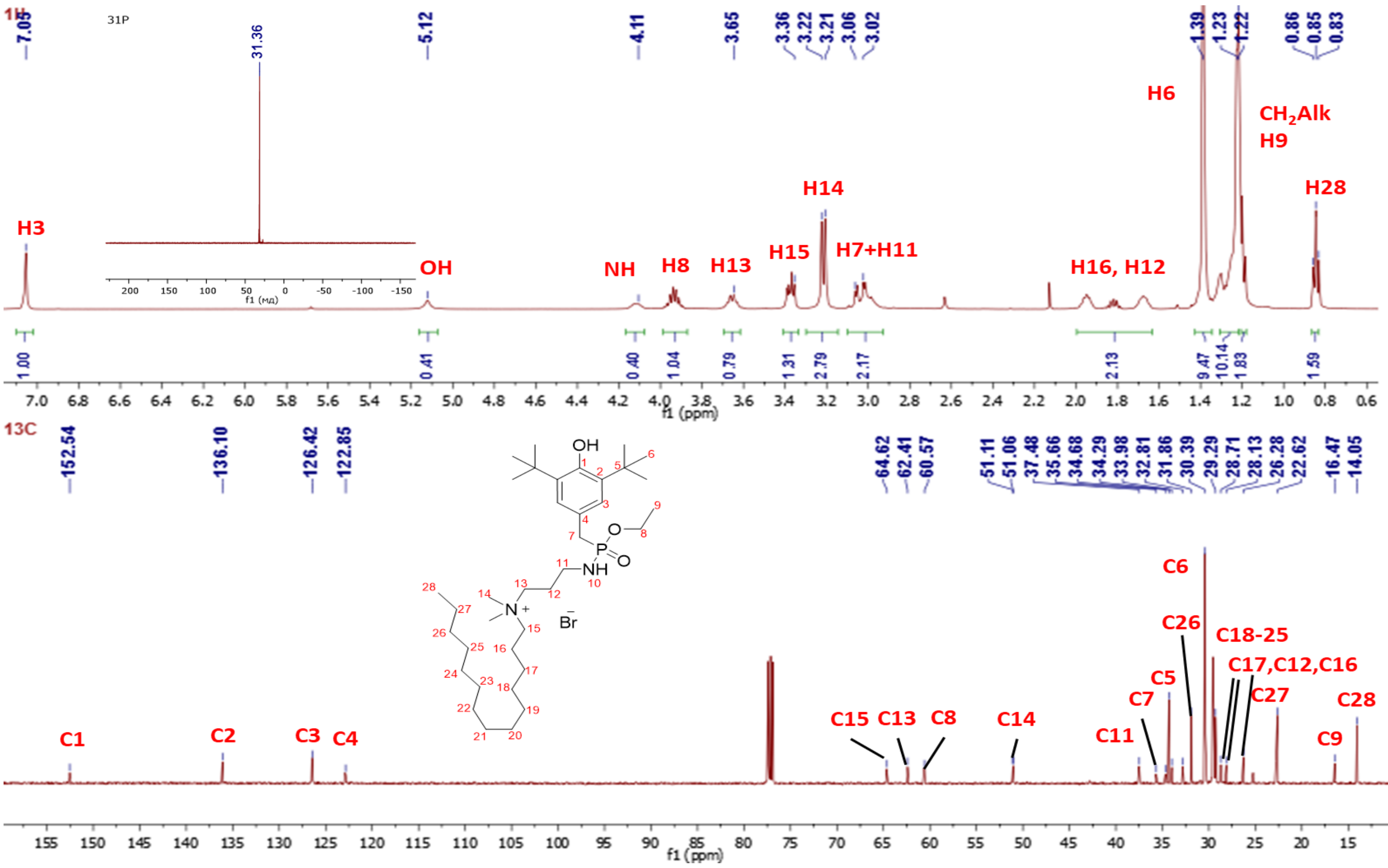

Figure S46. ^{1}H-, ^{13}C, ^{31}P- NMR of compound **14b**

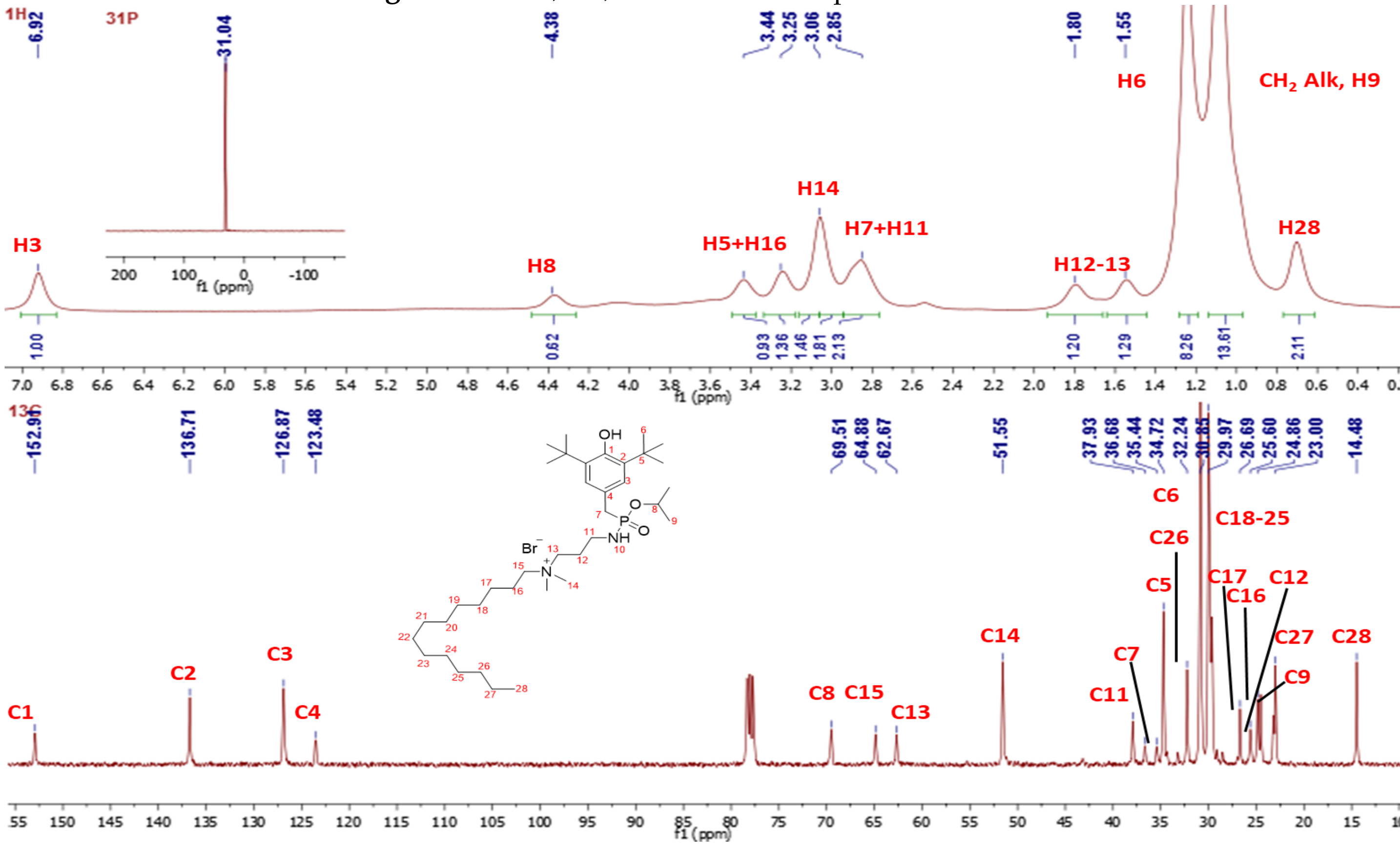

Figure S47. ^{1}H-, ^{13}C, ^{31}P- NMR of compound **14c**

2D correlation NMR experiments ^{1}H-^{1}H COSY, ^{1}H-^{13}C HSQC, ^{1}H-^{13}C HMBC of compound 14c

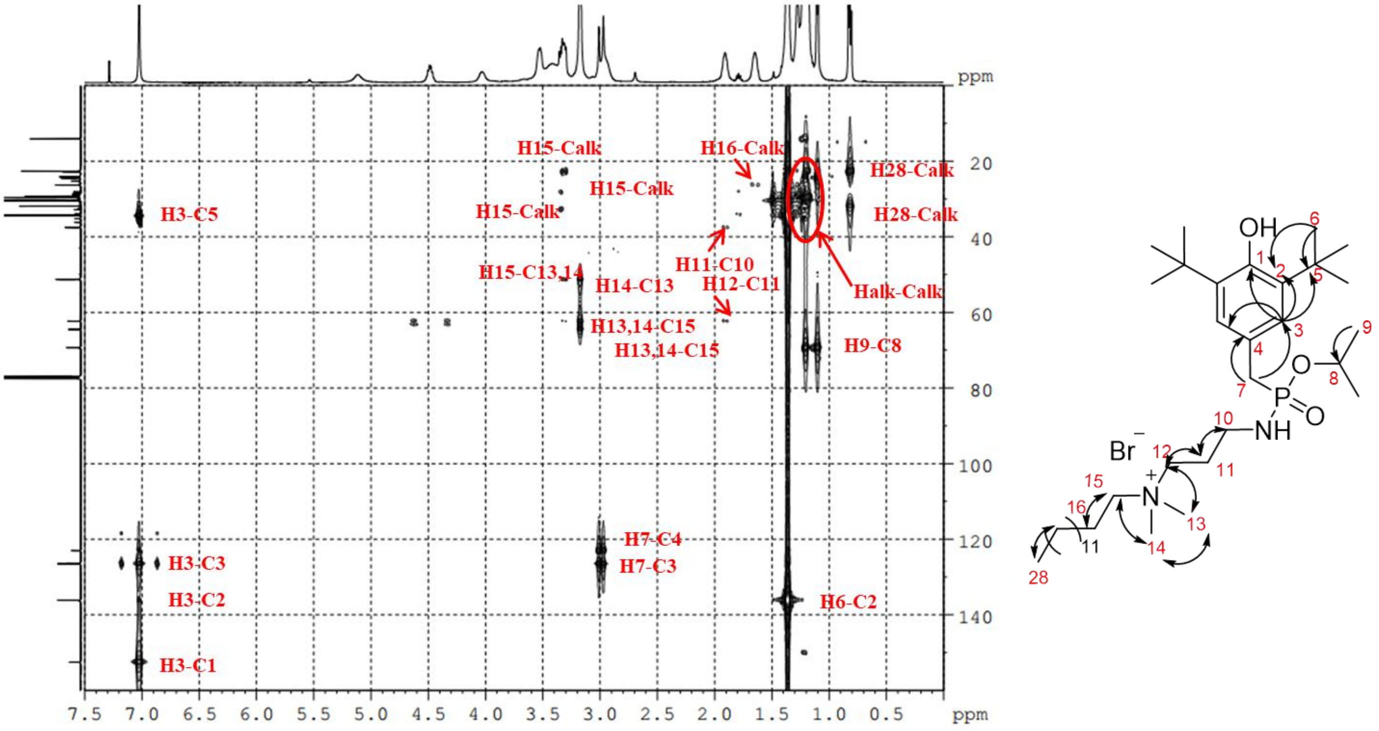

Figure S48. ^{1}H- ^{13}C HMBC correlations

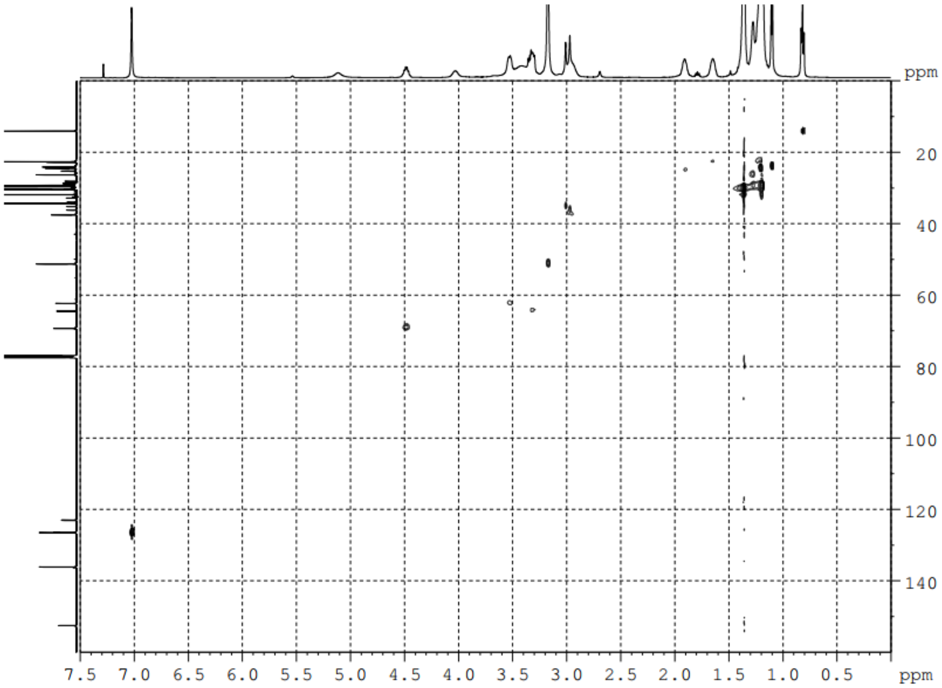

Figure S49. ^{1}H- ^{13}C HSQC correlations

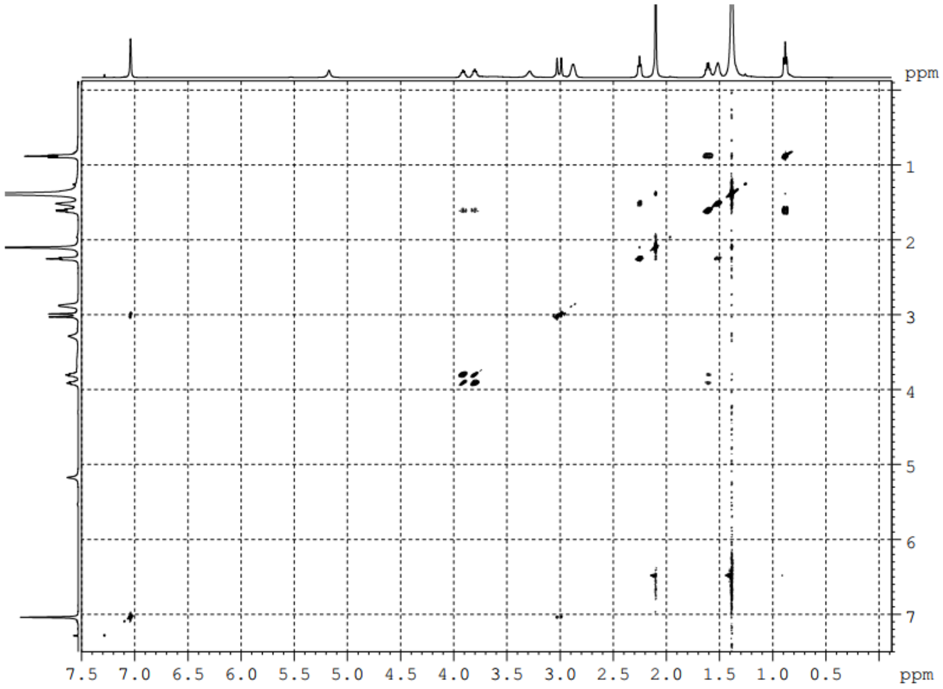

Figure S50. ^1H- ^1H COSY correlations

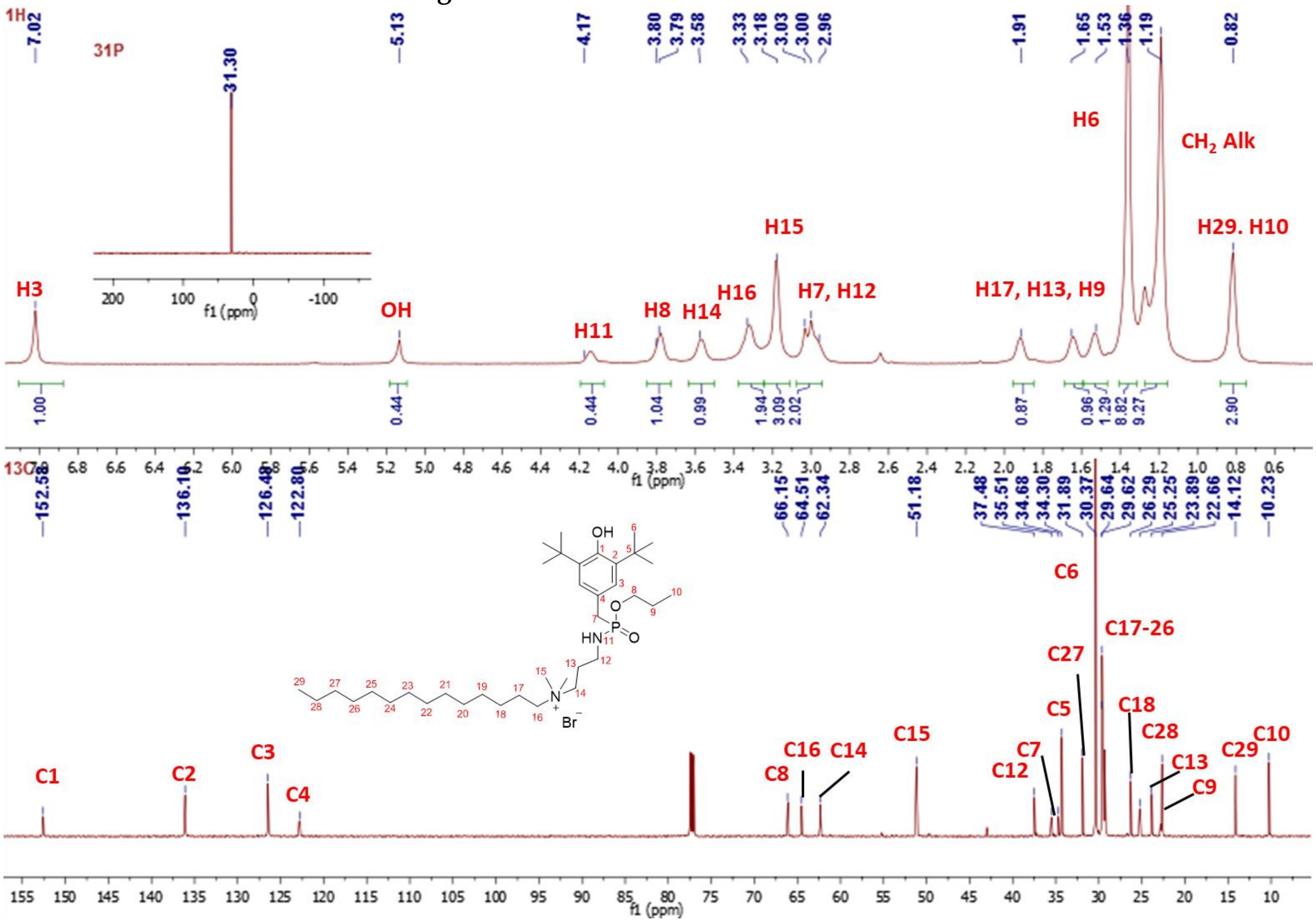

Figure S51. ^1H-, ^{13}C, ^{31}P- NMR of compound **14d**

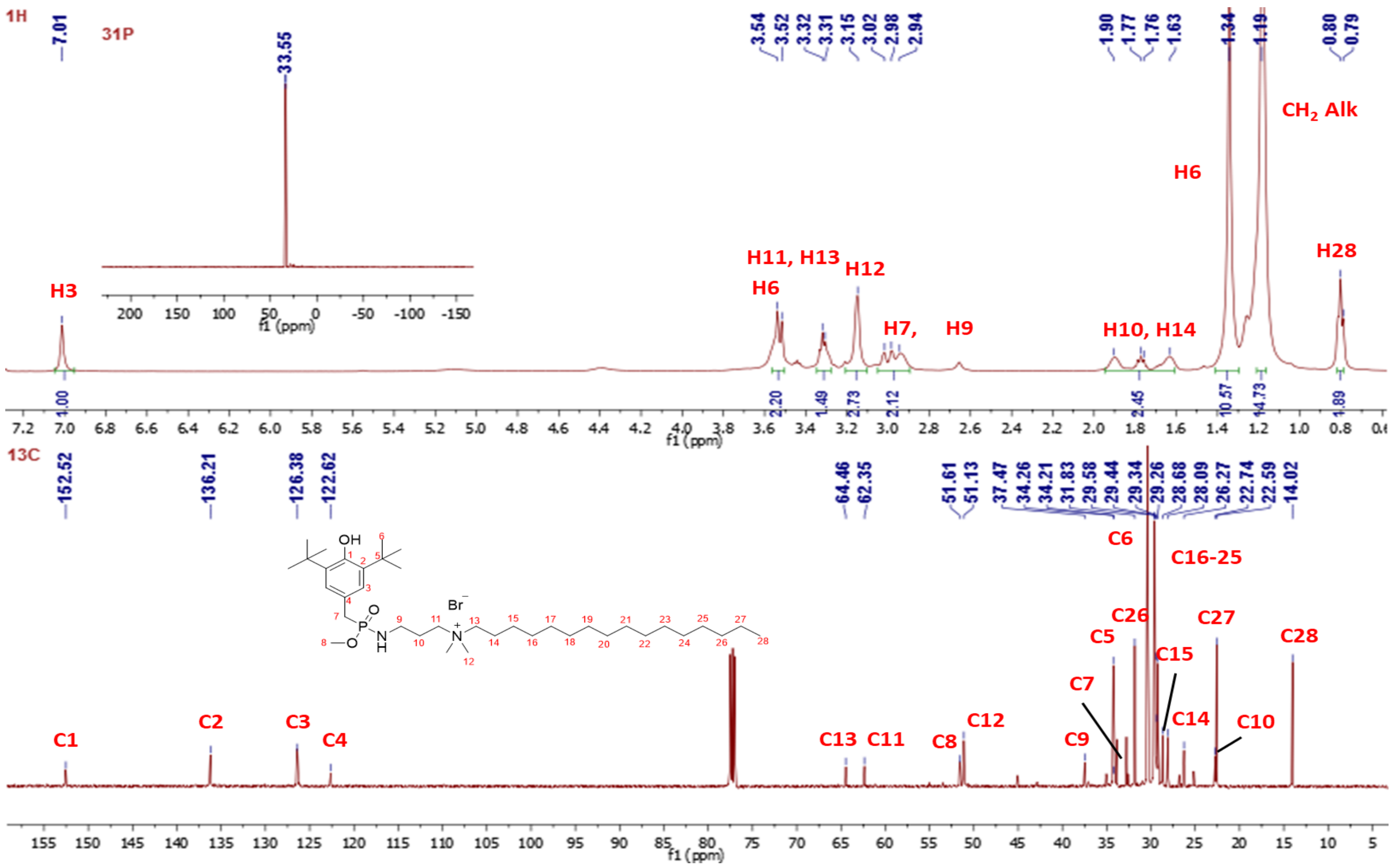

Figure S52. ^{1}H-, ^{13}C, ^{31}P- NMR of compound **15a**

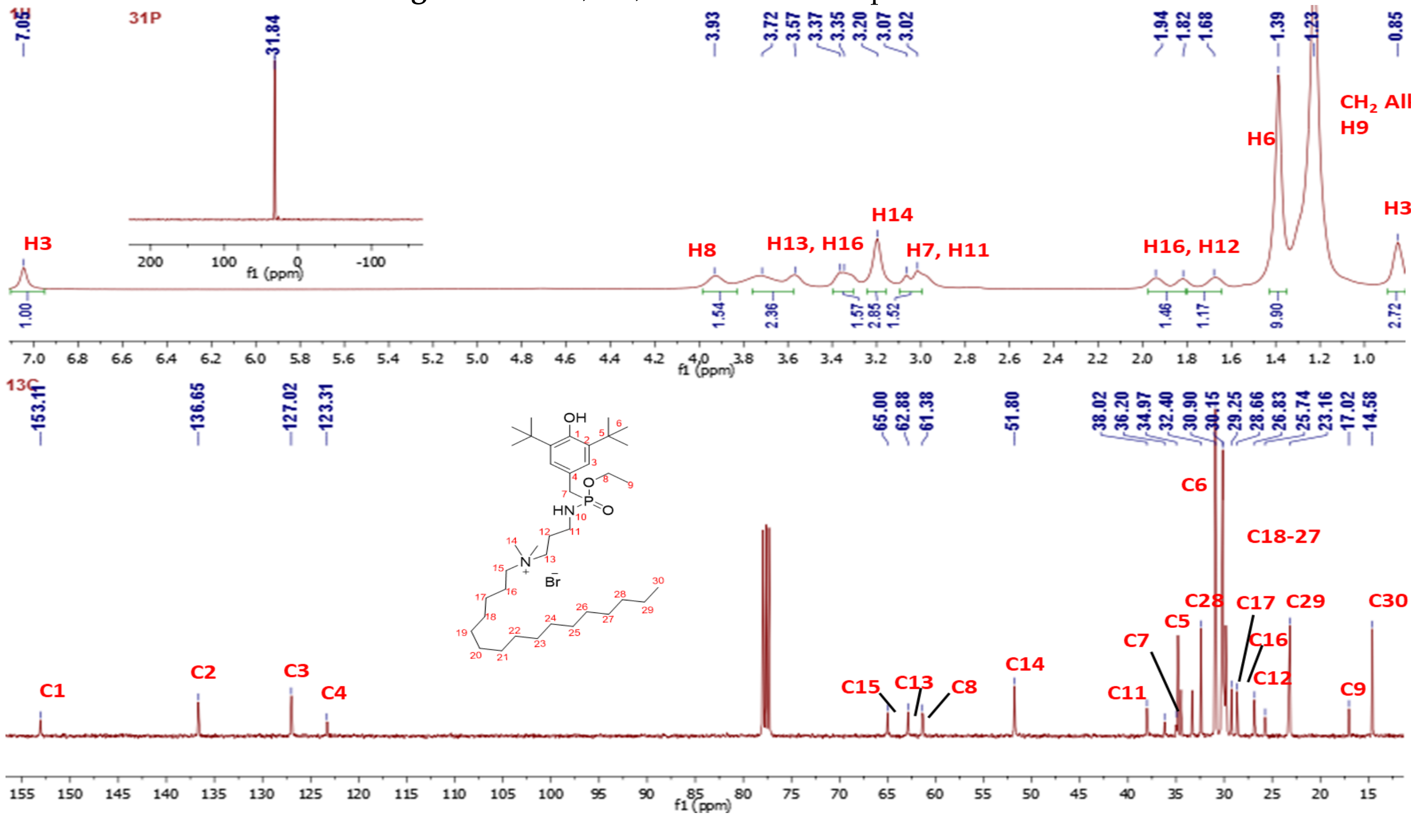

Figure S53. ^{1}H-, ^{13}C, ^{31}P- NMR of compound **15b**

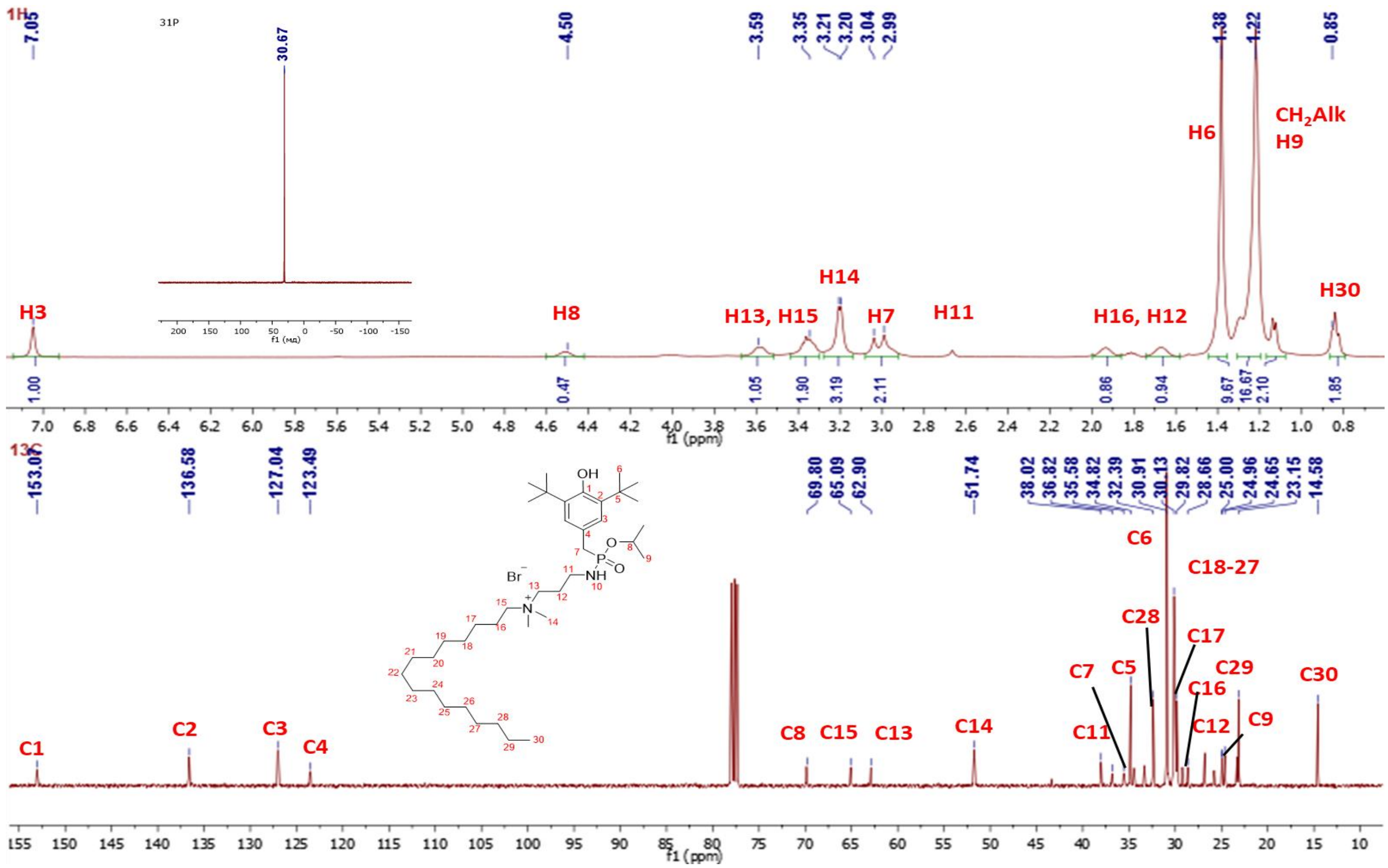

Figure S54. ^{1}H-, ^{13}C, ^{31}P- NMR of compound **15c**

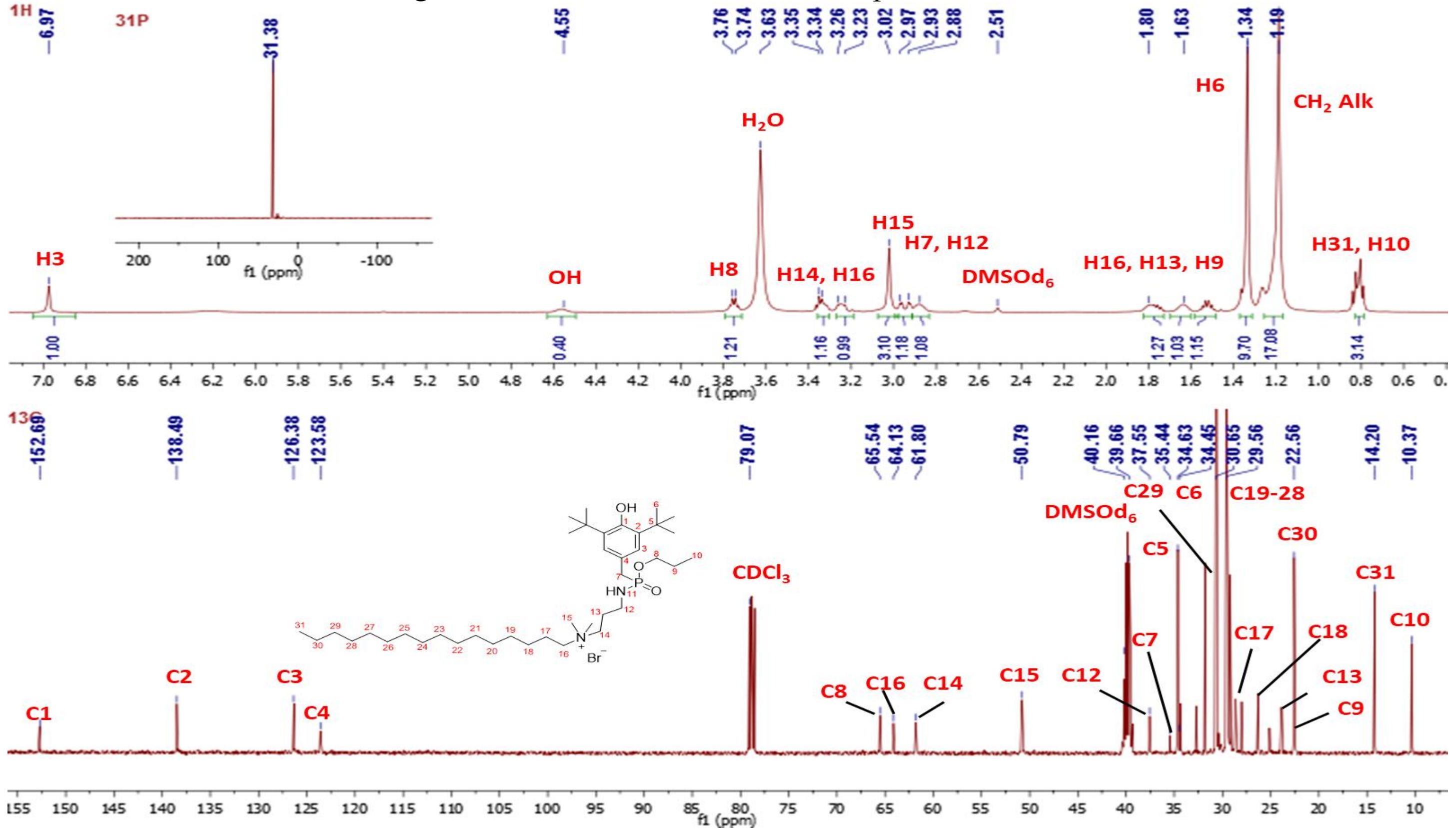

Figure S55. ^{1}H-, ^{13}C, ^{31}P- NMR of compound **15d**

Stability of compounds 8c, 12c, and 13c in biological media

The stability of selected compounds (**8c**, **12c** and **13c**) was evaluated in model media: Mueller-Hinton broth (for **12c**, **13c**), cell culture medium (for **12c**), and a solution of bovine serum albumin (BSA) (for **8c**). The experiment followed a protocol adapted from published procedures [5,6].

Sample preparation. Each compound (5.0 mg) was dissolved in a mixture of DMSO-d_6 (0.050 mL) and D_2O (0.15 mL). A control ^{31}P NMR spectrum was recorded at 37 °C. Then, 0.30 mL of the corresponding medium was added.

Incubation and NMR measurements. After addition of the medium, the sample was incubated at 37 °C. ^{31}P NMR spectra were recorded at 1 h, 12 h, and 24 h after the addition of the medium.

Results. For all tested compounds and media, no degradation was observed at any time point. The ^{31}P NMR spectra (control, 1 h, 12 h, 24 h) are presented below (Figures S55-S58).

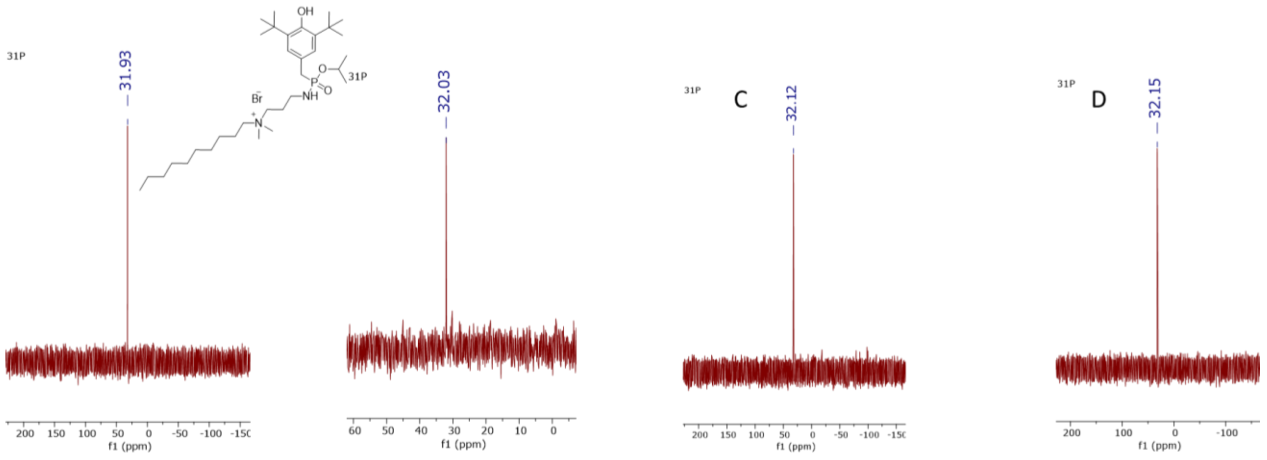

Figure S56. ^{31}P NMR spectra of compound **12c** in Mueller-Hinton broth after incubation at 37 °C. **A**– control (before incubation); **B** – after 1 h; **C**– after 12 h; **D** – after 24 h.

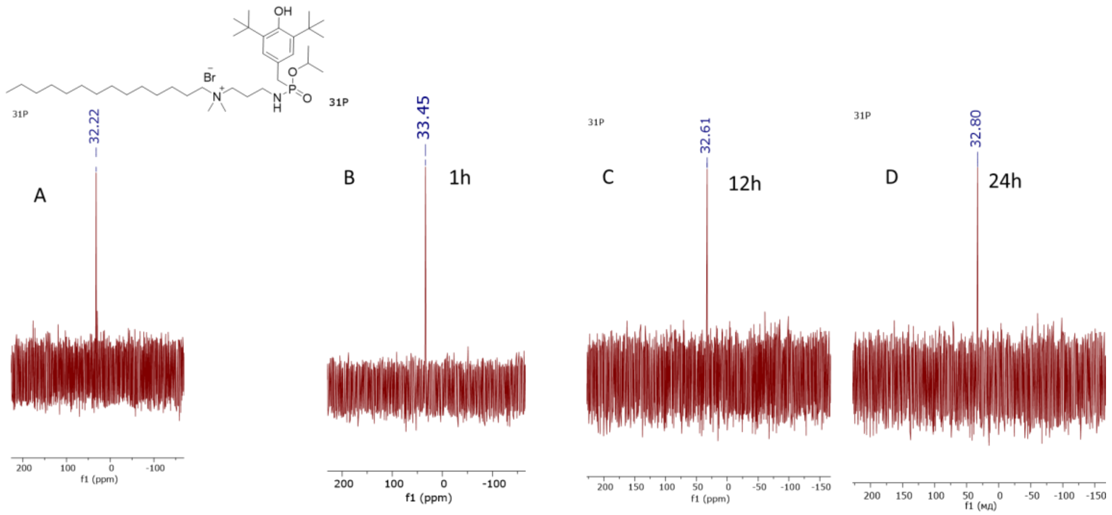

Figure S57. ^{31}P NMR spectra of compound **13c** in Mueller-Hinton broth after incubation at 37 °C. **A** – *control;* **B** *– after 1 h;* **C** *– after 12 h*, **D** *– after 24 h.*

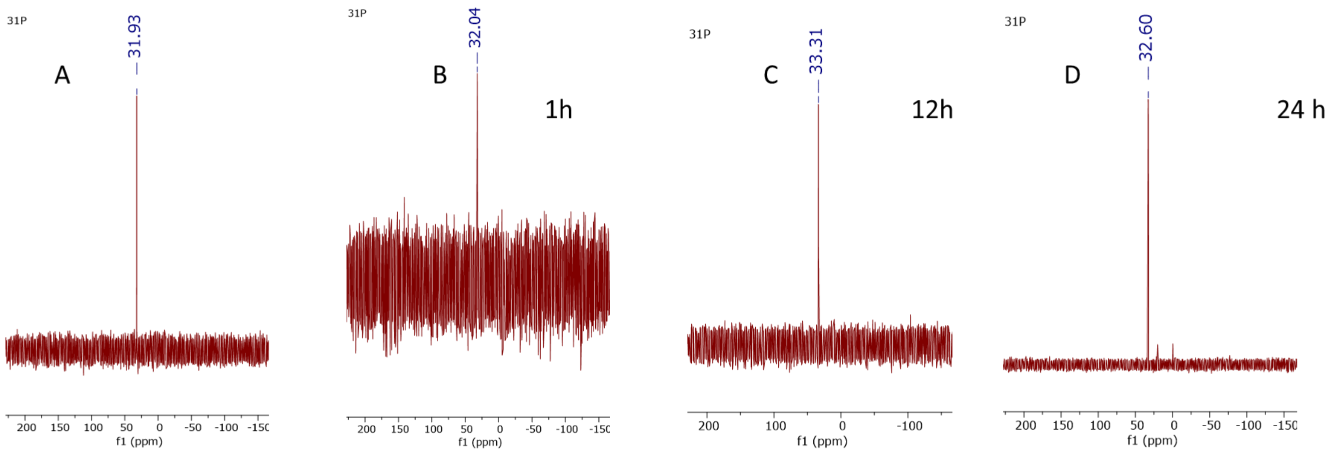

Figure S58. ^{31}P NMR spectra of compound **12c** after incubation in cell culture medium. A – reference signal (control); B – after 1 h; C – after 12 h; D – after 24 h.

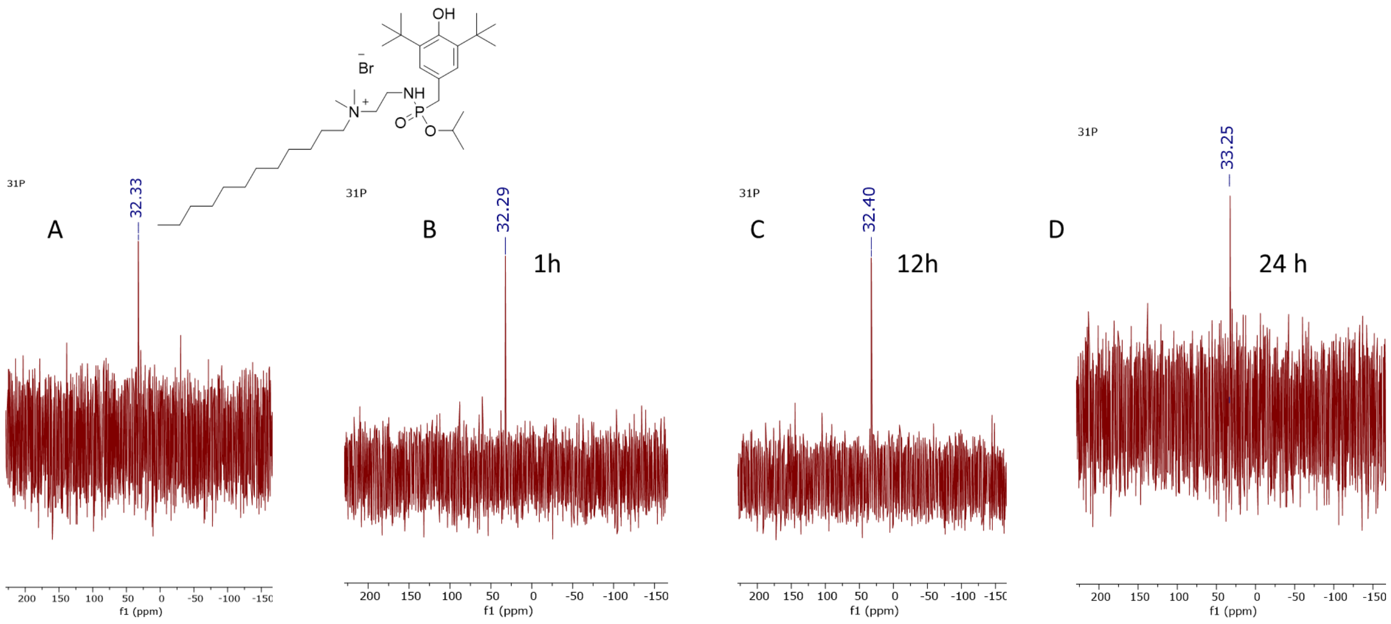

Figure S59. ^{31}P NMR spectra of compound **8c** after incubation in bovine serum albumin (BSA). **A** – reference signal (control); **B**– after 1 h; **C**– after 12 h; **D** – after 24 h.

Cytotoxicity data

Table S2 Dose-response curves for the cytotoxic activity of selected phosphonamidates against Chang Liver cells. Curves were generated using the online IC50 calculator (Quest Graph™, AAT Bioquest, Inc.)

7a	8a	9a
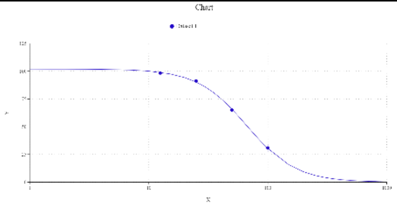	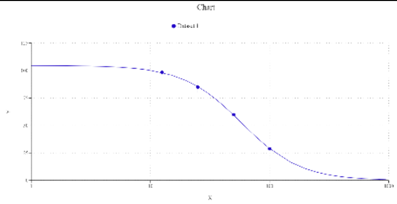	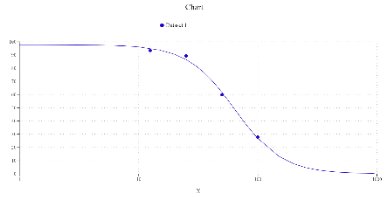
13a	14a	7b
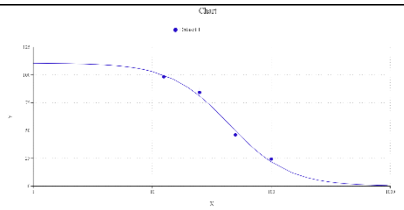	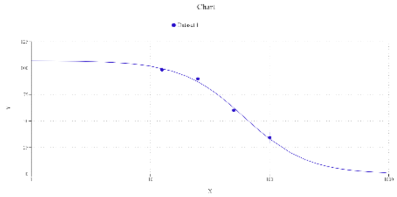	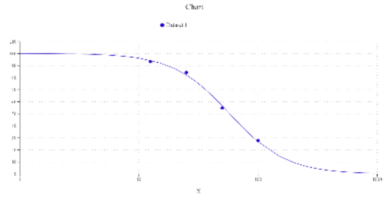
9b	12b	13b
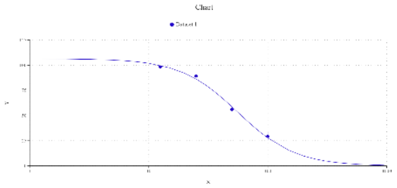	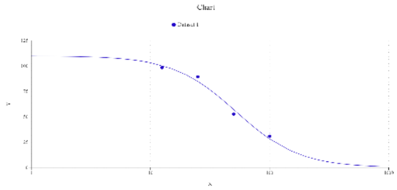	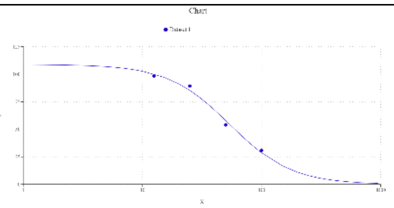
7c	8c	9c
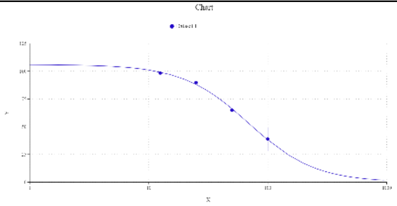	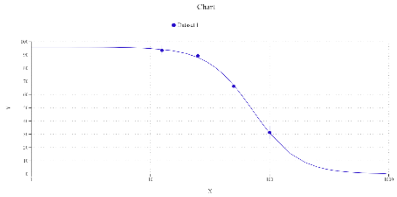	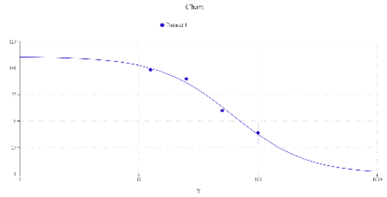
13c	14c	15c

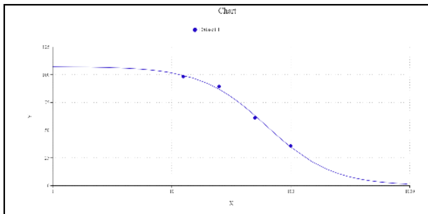	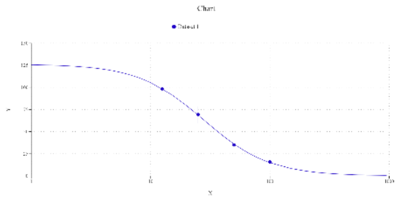	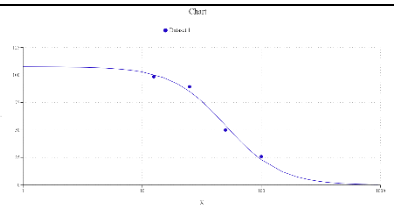
12d	13d	14d
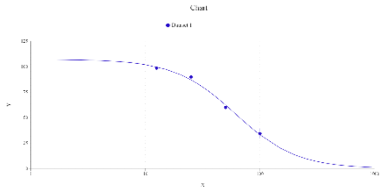	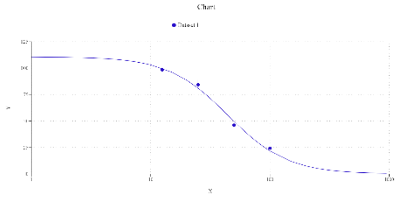	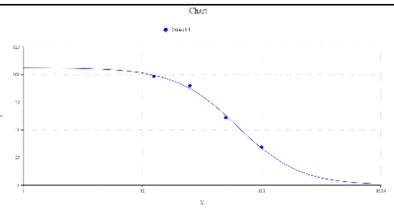
12a	8b	14b
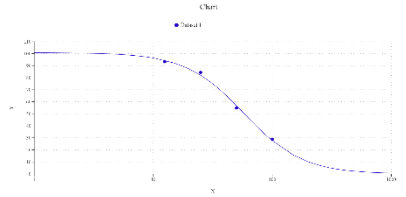	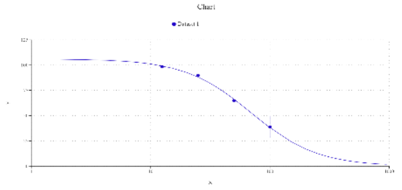	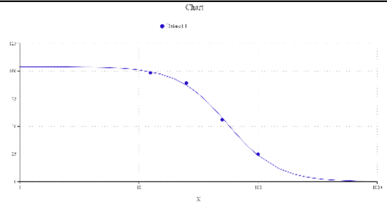
12c	7d	15d
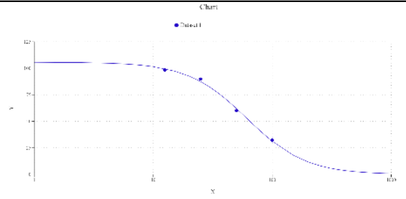	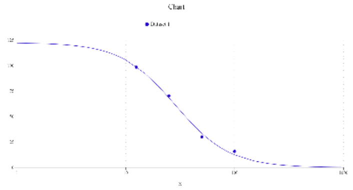	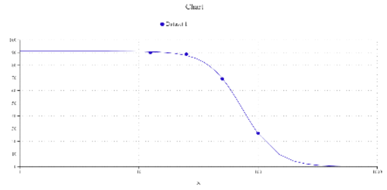
CTAB		
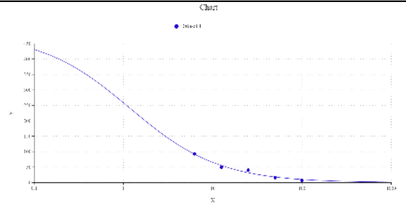		

References

1. Gibadullina, E.; Nguyen, T.T.; Strelnik, A.; Sapunova, A.; Voloshina, A.; Sudakov, I.; Vyshtakalyuk, A.; Voronina, J.; Pudovik, M.; Burilov, A. New 2,6-Diaminopyridines Containing a Sterically Hindered Benzylphosphonate Moiety in the Aromatic Core as Potential Antioxidant and Anti-Cancer Drugs. *Eur J Med Chem* **2019**, *184,* 111735. doi:10.1016/j.ejmech.2019.111735.

2. Gibadullina, E.M.; Shaekhov, T.R.; Badrtdinov, A.K.; Burilov, A.R. α-Phosphorylated 2,6-Di-*tert*-Butyl-4-Methylidene-2,5-Cyclohexadienones in the Reactions with Meta-Phenylenediamine. *Russian Chemical Bulletin* **2014**, *63,* 1455–1456 . doi:10.1007/s11172-014-0619-2.
3. Sheldrick, G.M. SHELXTL, version 6.12; Structure Determination Software Suite; Bruker AXS: Madison, WI, USA, 2000.
4. Sheldrick, G.M. Crystal structure refinement with SHELXL. Acta Crystallogr. Sect. C Struct. Chem. 2015, 71, 3–8. https://doi.org/10.1107/S2053229614024218.Bruker. APEX3 Crystallography Software Suite, Bruker AXS, Inc., Madison, WI, USA. **2016**.
5. Kadri, H.; Taher, T.E.; Xu, Q.; Sharif, M.; et al. Aryloxy Diester Phosphonamidate Prodrugs of Phosphoantigens (ProPAgens) as Potent Activators of Vγ9/Vδ2 T-Cell Immune Responses. *J. Med. Chem.* **2020**,*63*, 11258–11270. doi:10.1021/acs.jmedchem.0c01232.
6. Slusarczyk, M.; Lopez, M.H.; Balzarini, J.; Mason, M.; et al. Application of ProTide Technology to Gemcitabine: A Successful Approach to Overcome the Key Cancer Resistance Mechanisms Leads to a New Agent (NUC-1031) in Clinical Development. *J. Med. Chem.* **2014**, *57*, 1531–1542. doi:10.1021/jm401853a.
